# Autocatalytic Reaction between HCN and Cysteamine Produces Hydrophobic, Catalytic, Liquid Compartments

**DOI:** 10.1021/jacs.5c09581

**Published:** 2025-09-03

**Authors:** Alexander I. Novichkov, Yael Diskin-Posner, Linda J. W. Shimon, Gregory Leitus, Christoph Flamm, Sergey N. Semenov

**Affiliations:** † Department of Molecular Chemistry and Materials Science, 34976 Weizmann Institute of Science, Herzl Street 234, Rehovot 7610001, Israel; ‡ Department of Chemical Research Support, 34976 Weizmann Institute of Science, Herzl Street 234, Rehovot 7610001, Israel; § Department of Theoretical Chemistry, University of Vienna, Währinger Strasse 17, Vienna 1090, Austria

## Abstract

Although HCN has been explored extensively as a precursor in the prebiotic synthesis of biological molecules, macroscopic system-level phenomena, originating from reactions of HCN, such as autocatalysis, oscillations, pattern formation, and phase separation have attracted less attention. Autocatalysis and phase separation are particularly interesting in the context of the origin of life because they are sources of self-replication and compartmentalization. In this work, we investigate the reaction between HCN and cysteamine in water, which exhibits both sigmoidal reaction kinetics and the formation of a distinct liquid phase. We studied the origin of the sigmoidal kinetics using NMR spectroscopy and other techniques, investigated the chemical composition of the products using single-crystal X-ray diffraction and mass spectrometry, and probed the absorption of inert additives into the second liquid phase. Our studies show that the sigmoidal kinetics arise from an autocatalytic feedback loop driven by both an increase in pH and the catalytic nature of the newly formed phase itself. Product analysis revealed co-oligomers with a backbone derived from HCN and branches from cysteamine. This composition suggests that co-oligomerization with thiols provides a route to tractable oligomers, mitigating the formation of insoluble HCN polymers. Furthermore, this second liquid phase effectively sequesters hydrophobic molecules like benzene, demonstrating its capacity to act as a primitive compartment. The phenomena that we observed may provide some insight into prebiotic chemical networks and early-stage chemical evolution.

## Graphical abstract

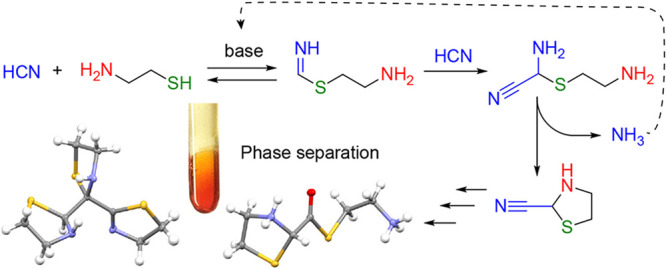

## Introduction

The origin of life on Earth remains one of the most challenging and multidisciplinary problems in modern science. The majority of this work focuses on the prebiotic synthesis of specific biochemical molecules and the development of conditions for the chemical replication of RNA.^1–5^ Remarkable work in prebiotic chemistry has demonstrated that nucleotides,^6–10^ amino acids,^11,12^ nucleic acids,^13–17^ peptides,^18–21^ sugars,^22–25^ and central metabolites^26–29^ could form under prebiotically plausible conditions.

The synthesis of complex molecules is not the sole characteristic of life. Equally important is its ability to utilize energy to replicate itself and navigate its environment.^30–33^ Simplistically speaking, the machinery of life operates on the chemical potential of the food it consumes. In this context, it is reasonable to pose the following questions: How do metastable prebiotic mixtures discharge their chemical potential? And how do these mixtures evolve when reactants are constantly supplied?

Naturally, system-level phenomena, such as network autocatalysis and phase separation,^34–37^ would play an important role in answering these questions. Autocatalysis represents a kinetically very stable state,^34,38^ a kind of kinetic trap where complex systems subjected to perturbation would fall if they could reach this state. On the other hand, liquid–liquid phase separation leads to compartmentalization,^36,37,39–42^ greatly expanding the space for available chemistry and thus increasing the chances of finding a more stable kinetic state. Moreover, the effects of autocatalysis and compartmentalization can be synergistic. First, compartments can retain autocatalytic species, thereby further accelerating their production. Second, compartments may also trap reactive intermediates (e.g., free radicals), enabling otherwise improbable reaction pathways, some of which can become autocatalytic. Third, autocatalysis and compartmentalization together can exert combined selective pressures, ensuring that only species capable of catalyzing their own production and remaining within compartments survive. Finally, if autocatalysis develops into self-replication, compartmentalization can mitigate effects of parasitic cross-catalysis and thereby facilitate evolution.^38^ However, system-level phenomena have often been overlooked in studies of simple prebiotic chemistry.^33^

Perhaps the most studied molecule in the context of the origin of life is hydrogen cyanide (HCN).^1,43–49^ This molecule has multiple reactivity modes and a high thermodynamic driving force for its reactions. HCN can act as an electrophile, yield the nucleophilic cyanide ion (CN^–^), and undergo oxidation and reduction. It is available through astrochemistry and geochemistry.^50–52^ The polymerization of HCN was recognized as a possible source of prebiotic molecules as early as the 1960s, when Oro found adenine in the products of HCN polymerization.^43–46^ Following this discovery, a large amount of work was dedicated to investigating HCN polymerization.^53–55^ Products of this reaction, some of which were insoluble oligomers, were studied in detail.^48,54,55^ On the other hand, macroscopic system-level phenomena, originating from reactions of HCN, such as autocatalysis, oscillations, pattern formation, and phase separation have attracted much less attention. In the example of autocatalysis, it is known that liquid HCN can undergo explosive polymerization and that heterogeneous polymerization of NH_4_CN at high temperature has some autocatalytic character.^49,56,57^ However, the contribution of the autocatalytic pathway to the overall HCN transformation in these reactions, a key measure of autocatalytic efficiency according to Kiedrowski,^34,58^ is limited, as indicated by the poorly resolved lag and exponential phases.^49,56,57^

The combination of high thermodynamic potential for reactivity and kinetic inertness of nitrile group motivated us to look into reactivity of HCN in the presence of thiols, which are known to activate nitriles. Surprisingly little is known about HCN reactions with thiols. It has been shown that thiophenol and its disulfide catalyze formation of diaminomaleonitrile from HCN in situ generated from acetone cyanohydrin in dimethylformamide (DMF),^59^ while the reactivity of HCN with organic thiols in water remains largely unexplored.

In this work, we investigated an aqueous reaction between HCN and cysteamine–the simplest stable aminothiol, chosen for its rich reactivity and recently discussed prebiotic relevance as a mediator for peptide ligation and a precursor of Coenzyme A.^60–62^ Our investigation into the reaction’s kinetics, mechanism, and products revealed three features of significant prebiotic interest: (i) autocatalysis, (ii) liquid–liquid phase separation, and (iii) the formation of tractable oligomers instead of insoluble polymers. Moreover, the products of the reaction can generate useful functions such as compartmentalization and catalysis.

## Results and Discussion

### Autocatalytic Nature of the Reaction between HCN and Cysteamine

In our investigation of the HCN plus cysteamine hydrochloride (MEA·HCl) system, we noticed a lag phase in the onset of the reaction. When continuously observed by an automatic photoacquisition system, the reaction between HCN (1M) and MEA·HCl (1M) displayed no visible changes during the first 12 h after which it gradually changed from light yellow to red-brown (Figure 1A). Moreover, the color change was accompanied by the formation of a second liquid phase. The formation of this phase started with a cloudy appearance of the solution, and then small droplets formed and merged into larger ones at the bottom of the vial (Figure 1A). The second liquid was more viscous and more intensely colored than the water solution around it. Importantly, we used HCN solution prepared from pure HCN liquid to eliminate variability in the initial pH arising from imprecise ratios of KCN and mineral acids, as well as to avoid interference from byproduct salts. For safety protocols regarding the preparation and handling of HCN, see the Supporting Information Section 1.

**Figure 1:**
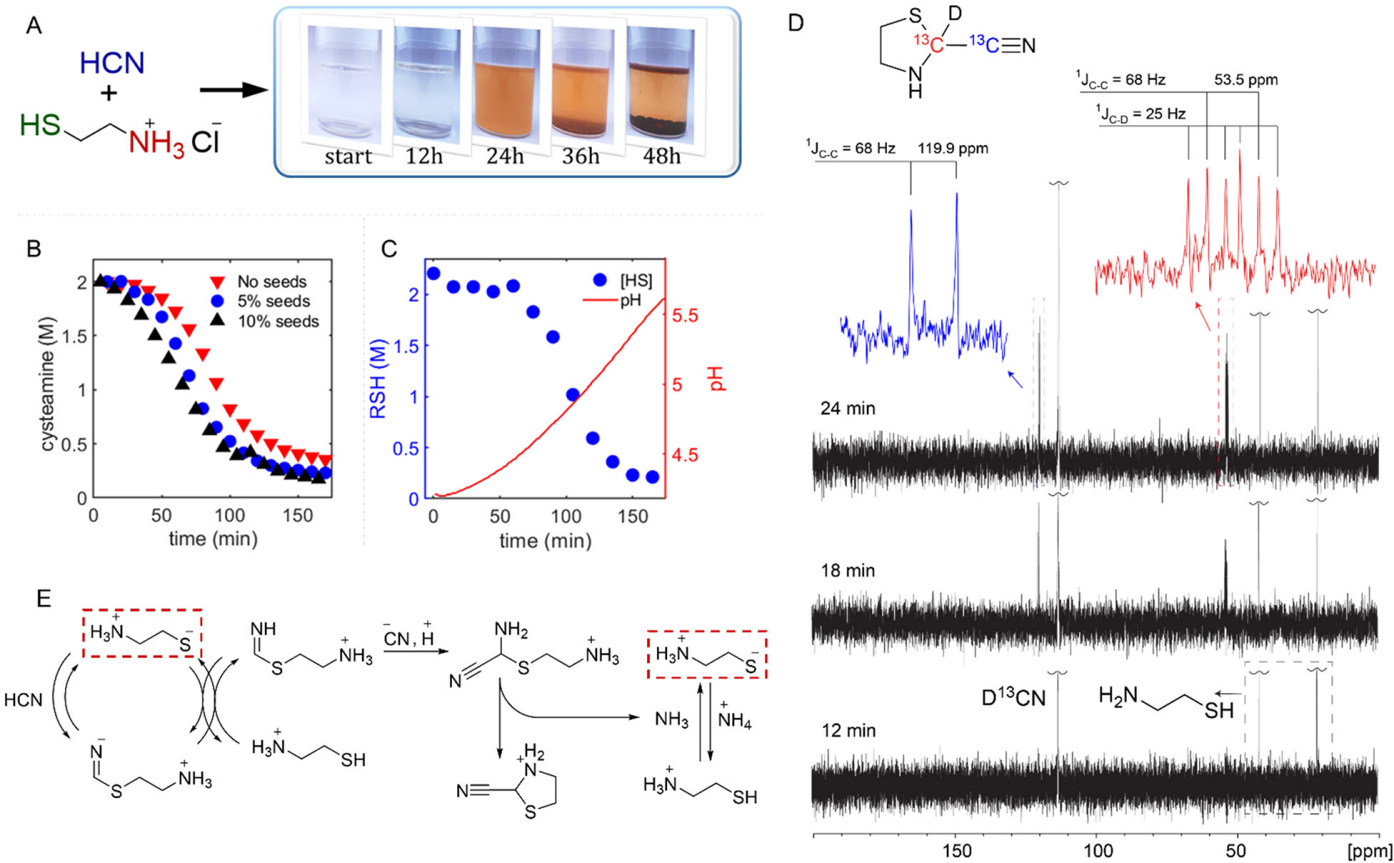
Autocatalysis in the reaction between HCN and cysteamine. (A) Visual monitoring of the aqueous reaction between HCN (1M) and cysteamine hydrochloride (MEA·HCl) (1M) at 25 °C (pH 4.3 from reactants). (B) Standard addition experiments. MEA·HCl concentration was determined by ^1^H NMR using *t*-BuOH as an internal standard. Reaction conditions: D_2_O, HCN (2M), MEA·HCl (2M), 40 °C. For seeding, we used the products of the same reaction after 8 h when it is nearly complete. (C) Monitoring of the integral concentration of thiol (SH) functionality and pH during the reaction between HCN and MEA·HCl. Reaction conditions: H_2_O, HCN (2M), MEA·HCl (2M), 40 °C. Thiol concentration was measured at 15 min intervals by Ellman’s test (see Supporting Information Section 6a). For the same reaction mixture, pH was monitored by a pH-electrode in real-time. (D) ^13^C NMR studies (DEPTQ, inverted for clarity) of the initial stages of the reaction between K^13^CN (1M) and MEA·HCl (1M) in the presence of KH_2_PO_4_ (1M) at 25 °C in D_2_O. (E) Plausible pathway leading to pH-driven amplification. Dashed squares highlight thiolate species whose concentration increases during this amplification cycle.

The system’s nonlinear visual changes, including color shifts and the formation of a second liquid phase, suggested sigmoidal kinetics in the reaction and the possibility of autocatalysis. To confirm the sigmoidal kinetics, we monitored the concentration of cysteamine over the course of the reaction by integrating the signals of cysteamine’s CH_2_ groups in ^1^H NMR spectra. For practical convenience, we accelerated the reaction by increasing the reactants’ concentrations from 1 to 2 M and raising the temperature from 25 to 40 °C. The experiment demonstrated that the reaction indeed has a sigmoidal kinetic profile (Figure 1B red triangles) with about 40 min lag phase followed by exponential and saturation phases.

Sigmoidal kinetics is an indication but not proof of autocatalysis. To validate our hypothesis about the autocatalytic nature of this reaction, we conducted experiments involving the addition of reaction products. If a reaction is autocatalytic, then the addition of its products at the beginning of the experiment should shorten or eliminate the lag phase. Therefore, we conducted experiments in which we added 5 or 10% (relative to the amount of reactants) of the mixture of products and monitored the progress of the reaction by ^1^H NMR (Figure 1B blue circles and black triangles). The addition of 5% of products shortened the lag phase to 25 min, while the addition of 10% of the products almost eliminated it. This seeding effect underscores the reaction’s autocatalytic nature. Nevertheless, even with 10% seeding, the lag phase does not disappear completely, and the reaction profile remains smooth. Similar observations have been reported for the Soai reaction, where doping with a transient hemiacetalate catalyst shifts the reaction traces in time but does not fully eliminate the lag phase.^63^

Next, we investigated the possible causes of the autocatalytic behavior observed in this reaction. The hydrolysis and polymerization of HCN release ammonia and other nitrogen bases.^48^ At the same time, the most reactive nucleophilic center in cysteamine is the thiolate (S^–^), the deprotonated form of the thiol (SH) group. Therefore, we hypothesized that an increase in pH during the reaction could be a source of autocatalysis. An increase in pH would increase the concentration of thiolate through deprotonation of the thiol group. This, in turn, would accelerate thiolate attack on HCN, releasing more ammonia and further raising the pH.

To test this hypothesis, we conducted a series of experiments. First, we simultaneously monitored the concentration of cysteamine (and all other SH-containing species) and pH in this reaction (Figure 1C and Supporting Information Section 6a). Expectedly, the cumulative concentration of SH groups (as determined by Ellman’s test) followed sigmoidal kinetics, which was similar to the kinetics of cysteamine consumption determined by ^1^H NMR. During the same experiment, pH increased from 4.2 to 5.6. Second, we initiated the reaction by adding 5 mol % KOH (Supporting Information Section 3i), which deprotonates approximately 5% of MEA·HCl. The reaction started instantly, confirming that basification alone is sufficient to initiate the process. Third, we reacted 1 M HCN with three compounds: (i) 1 M MEA·HCl, (ii) 1 M ethanolamine hydrochloride, and (iii) 1 M sodium 2-mercaptoethanesulfonate (MESNA) thiol (Supporting Information Section 2f). All reactions exhibited a delayed phase after mixing, as indicated by a lack of color change. The MESNA reaction commenced first, after approximately 1 h. MEA·HCl reacted completely within 1 day, while no reaction was detected for ethanolamine even after 4 days. This experiment confirms that the presence of an amine group alone is insufficient to drive autocatalysis and highlights the central role of the thiol group in the reaction.

To better understand the initiation of the reaction between HCN and MEA·HCl on a molecular level, we conducted ^13^C NMR studies of the reaction between D^13^CN (generated in situ from K^13^CN and KH_2_PO_4_) and MEA·HCl in D_2_O (Figure 1D). For the first 12 min, only signals corresponding to the starting materials were detected. At 18 min, two new signals appeared in the spectrum: a doublet at 119.9 ppm (*J* = 68 Hz) and a signal at 53.5 ppm, which presented as a doublet (*J* = 68 Hz) of 1:1:1 triplets (*J* = 25 Hz). Notably, these two signals grew synchronously over the subsequent 6 min, indicating they belong to a single, predominant product formed in the early stages. The structure consistent with this spectrum is 2-cyanothiazolidine. Specifically, the signal at 53.5 ppm exhibits splitting characteristic of coupling to both the nitrile carbon (^1^ *J*_C–C_ = 68 Hz, causing the doublet) and a single deuterium atom (^1^ *J*_C‑D_ = 25 Hz, causing the 1:1:1 triplet). The 68 Hz coupling constant is typical for a one-bond C–C coupling. Concurrently, the signal at 119.9 ppm is a doublet with the same 68 Hz splitting, confirming that the carbon atoms corresponding to these two signals are directly bonded. Furthermore, the chemical shift of 53.5 ppm is consistent with a thiazolidine ring carbon (specifically C2), while 119.9 ppm is characteristic of a nitrile carbon. These combined NMR data strongly support the formation of ^13^C- and deuterium-labeled 2-cyanothiazolidine as the initial major product. The detection of 2-cyanothiazolidine at early reaction stages allows us to propose an amplification cycle based on ammonia release and the resulting increase in basicity (Figure 1E). This mechanism illustrates that while the formation of 2-cyanothiazolidine itself does not directly consume or produce protons, the concomitant release of ammonia increases the pH of the solution. This, in turn, promotes the formation of the cysteamine thiolate, which is required to initiate the reaction with HCN, thereby creating a positive feedback loop that accelerates product formation. Interestingly, the proposed facilitation of C–C bond formation by amidine intermediates corroborates with proposed mechanism for thiophenol catalyzed formation of diaminomaleonitrile where similar steps are involved.^59^

Moreover, the nitrile in 2-cyanothiazolidine is likely more reactive than that in HCN, as evidenced by the lack of its long-term accumulation in the reaction mixture, observed via ^13^C NMR (Supporting Information Section 3h). We hypothesized that this enhanced reactivity could complement the pH-based amplification mechanism by releasing additional ammonia and thereby further increasing the pH. To test this hypothesis, we investigated whether malononitrile, a molecule also expected to have more reactive nitriles than HCN, would initiate the reaction. Indeed, the addition of just 5 mol % of malononitrile at the start of the reaction shortened the lag phase from 300 to 90 min compared to the control experiment (see Supporting Information Section 2g).

To confirm that a bulk pH change can fully explain the kinetics of the system, we conducted two additional experiments. First, we performed ^1^H NMR kinetic experiment for the reaction of HCN (2M) and MEA·HCl (2M) in the presence of 1 M phosphate buffer pH 6.5 (Supporting Information Section 3j), which is one pH unit above the values observed during the exponential phase (Figure 1C). The reaction started almost immediately, although a small lag can be noticed. Second, we performed the reaction of HCN (1M) and MEA·HCl (1M) in a 1 M acetate buffer pH 5 (Supporting Information Section 2e), corresponding to the pH at the midpoint of the exponential phase according to Figure 1C. No signatures of autocatalysis were observed under these conditions. The solution gradually, mildly darkened without phase separation. Considering these results and the fact that the pH variation during the exponential phase was less than one unit (Figure 1C), we conclude that pH is the major contributor to the autocatalysis in this system, but contributions from other mechanisms cannot be ruled out.

One of these mechanisms could be the catalytic activity of the second liquid phase formed in this reaction. To test this hypothesis, we conducted the reaction between HCN and MEA·HCl in the presence of neutral surfactant molecules that can suppress the formation of the second liquid phase by suppressing nucleation and growth of this phase. We used isopentenyl alcohol (3-methylbut-2-en-1-ol) as a weak and Triton X-100 as a strong neutral surfactant. Isopentenyl alcohol (100 mM) increased the lag phase from 550 to 770 min, while in the presence of Triton (∼100 mM) the reaction had not started for at least 1500 min (Figure 2A). We also conducted ^1^H NMR experiment, which showed that smaller (∼15 mM) concentrations of Triton still increased the lag phase from 40 to 180 min (Figure 2C) compared to the experiment under identical conditions without surfactants (Figure 1B). Interestingly, NMR signals of Triton gradually disappeared during the lag phase indirectly indicating incorporation of Triton into structures (e.g., surfactant-stabilized droplets) that are too large to give sharp NMR signals. Overall, these experiments indicate that the second liquid phase is critical for the exponential acceleration of the reaction.

**Figure 2:**
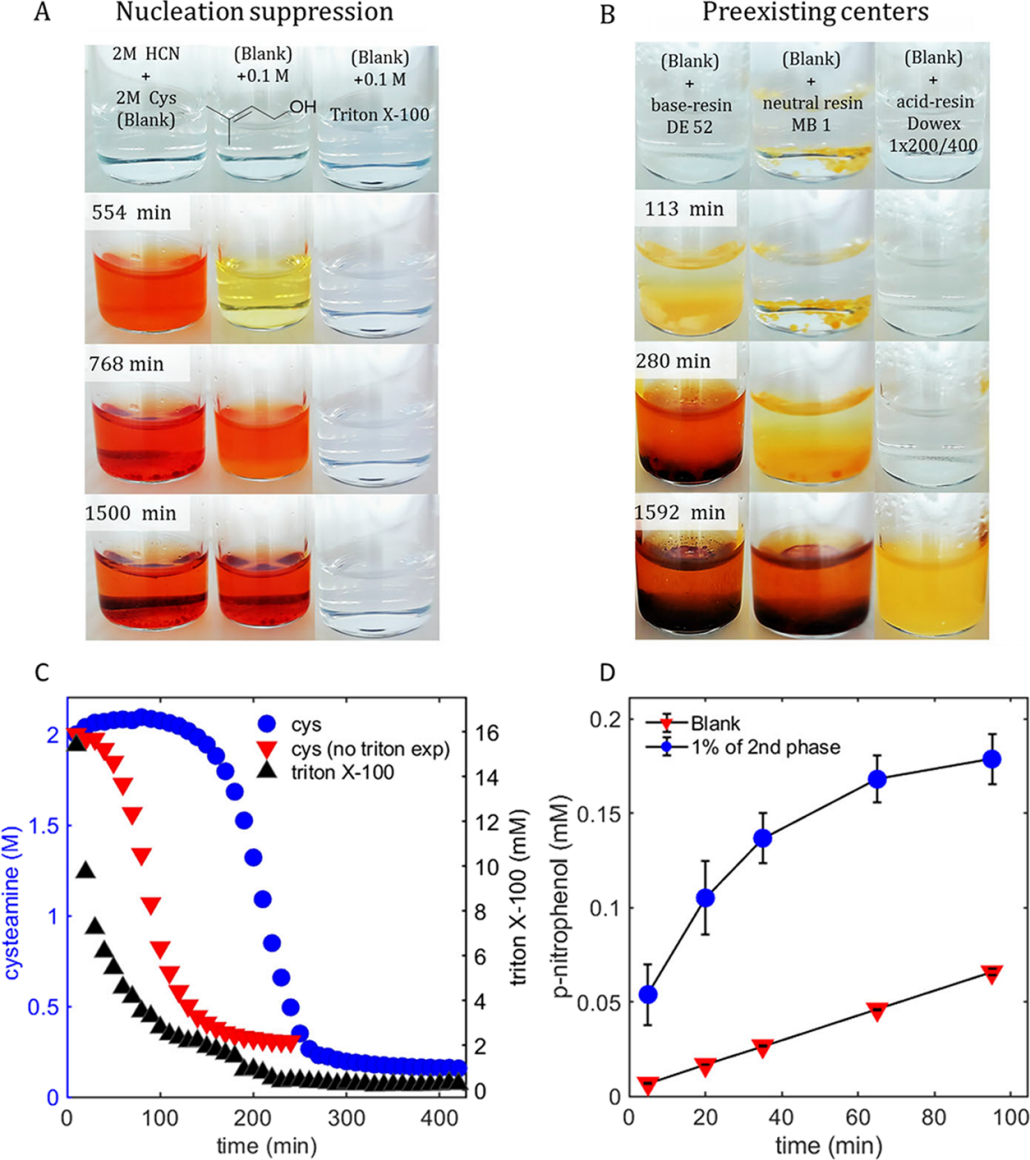
Investigation of the role of the second liquid phase in the autocatalysis in HCN-MEA·HCl reaction. (A) Investigation of the phase separation suppression by surfactants. Visual progression of the reaction between 2 M HCN and 2 M MEA·HCl in the absence of additives (left) and in the presence of 0.1 M isopentenyl alcohol (middle) and 0.1 M Triton X-100 (right). (B) Investigation of the influence of pre-existing nucleation centers on the reaction. Visual progression of the reaction between 2 M HCN and 2 M MEA·HCl in the presence of three ion-exchange resins: basic DE-52 (left), neutral MB-1 (middle), and acidic Dowex 1 × 200/400 (right). (C) The ^1^H NMR kinetic study of the reaction between 2 M HCN and 2 M MEA·HCl at 40 °C in the presence of Triton X-100 (15 mM) (blue line – cysteamine; black line – Triton) demonstrates a significant delay in the autocatalytic process compared to the experiment conducted in the absence of Triton (red line – cysteamine). (D) Comparison of the hydrolysis rate of *p*-nitrophenol acetate in pH 7 phosphate buffer (200 mM) with and without the second liquid phase (1 vol %). Error bars indicate the standard deviation based on three independent experiments.

In the next series of experiments, we tested whether the lag phase could be shortened by promoting nucleation and growth of the second liquid phase. We conducted the reaction between HCN and MEA·HCl on top of the beads of ion-exchange resins (Figure 2B). We chose three resins – DE-52, MB-1, and Dowex 1 – because of the different acidity of their surface groups. When suspended in water, DE-52 produced basic, MB-1 neutral, and Dowex 1 acidic solutions. The basic resin accelerated the reaction strongly with the lag phase being shortened from 550 to 115 min. Interestingly, the neutral resin also accelerated the reaction with the lag phase being shortened from 550 to 280 min. Finally, the acidic resin slowed the reaction, which started only at about 1600 min. These experiments pointed toward the importance of two factors for the initiation and progress of autocatalysis: (i) the presence of nucleation points for the second liquid phase, and (ii) the basicity of the solution. Thus, neutral resin provided nucleation points but no pH increase and accelerated the reaction less than the basic resin that provided both nucleation points and pH increase.

In addition, we investigated whether supramolecular aggregation precedes liquid–liquid phase separation by monitoring the reaction between HCN and MEA·HCl at 25 °C using dynamic light scattering (DLS) (Supporting Information Section 7a). The measurements revealed the appearance of species with a size distribution peaking at approximately 1 nm, around 30 min prior to the onset of phase separation. However, this size is too small to support the formation of organized supramolecular assemblies, such as micelles or supramolecular polymers. Independent observations by optical microscopy (Supporting Information Section 7b) showed that the newly formed droplets of the second liquid phase were clear and did not contain visible solid particles.

To test whether the second liquid phase contains components acting as basic catalysts, we examined its effect on the rate of hydrolysis of *p*-nitrophenol acetate (Figure 2D and Supporting Information Section 6b). In these experiments, we added ∼100 mg of the second liquid phase to 10 mL of the solution of *p*-nitrophenol acetate (0.4 mM) in phosphate buffer pH 7. The experiments demonstrated a 5× increase in the initial rate of hydrolysis of *p*-nitrophenol acetate by the second liquid phase. Overall, the experiment shown in Figure 2 demonstrated that the second liquid phase plays an important role in the autocatalytic acceleration of the reaction between HCN and MEA·HCl. To better understand the reasons for the catalytic activity of the second liquid phase, we studied the molecular composition of products of the reaction between HCN and MEA·HCl in detail.

### Reaction Network Originating from HCN Cysteamine Reaction

Analyzing the reaction products presents significant challenges, with mass spectrometry revealing more than 30 distinct compounds (Table 1). Examination of extracts from both the second liquid phase and the aqueous solutions above it showed no substantial differences in the detected products. This observation suggests that, in the early stages of the reaction, most transformations take place in the solution phase. Nevertheless, after a day or more, the second phase becomes almost black, indicating ongoing polymerization processes.

**Table 1:** Summary of the Mass Spectrometry Studies of the Reaction of MEA·HCl with Natural Isotope Distribution, ^13^C Enriched, and ^15^N Enriched KCN in Phosphate Buffer^a^

mass	OF	number of atoms from HCN		cysteamine fragments	+H_2_O	–NH_3_	red/Ox balance
		n(C)	n(N)				–H_2_/+H_2_
87	9	1	0	1	no	1	0
89	9	1	0	1	no	1	+2[H]
102	10	1	1	1	no	no	–2[H]
104	15	1	1	1	no	no	0
105	8	1	0	1	yes	1	0
132	7	2	1	1	yes	1	0
141	6	3	2	1	no	1	0
164	15	1	0	2	no	1	0
172	13	2	0	2	no	2	–2[H]
174	28	2	0	2	no	2	0
179	9	1	1	2	no	no	–2[H]
191	15	2	1	2	no	1	0
201	**31**	3	1	2	no	2	0
203	24	3	1	2	no	2	+2[H]
211	**46**	4	1	2	no	3	0
213	23	4	1	2	no	3	+2[H]
226	28	4	2	2	no	2	–2[H]
228	7	4	2	2	no	2	0
243	10	4	3	2	no	1	–2[H]
261	7	3	0	3	no	3	0
298	**43**	5	1	3	no	4	0
300	**43**	5	1	3	no	4	+2[H]
315	21	5	2	3	no	3	0
317	23	5	2	3	no	3	+2[H]
325	15	6	2	3	no	4	0
327	12	6	2	3	no	4	+2[H]
424	12	8	2	4	no	6	+2[H]
426	12	8	2	4	no	6	+4[H]

a OF stands for observation frequency

Given the complexity of the chemical space formed by HCN and cysteamine, and the difficulty of distinguishing some compounds within this space by NMR, we initially studied the system by single-crystal X-ray diffraction. Although this approach does not provide a representative quantitative analysis of the mixture’s composition, it offers an unambiguous collection of molecular structures for compounds definitively present in the mixture, which can serve as starting points for deciphering the entire reaction network (Figure 3). We used four methods to process the reaction mixture for crystallization: (i) spontaneous crystallization from the reaction conducted in the presence of isopentenyl alcohol, (ii) separation of the second liquid phase from the solution, followed by its dissolution in perchloric acid and crystallization as perchlorate salts, (iii) acylation of the reaction mixture with benzoyl chloride, followed by chromatographic separation on a silica gel column and crystallization of the separated benzoyl derivatives, (iv) bocylation of the reaction mixture with Boc_2_O, followed by chromatographic separation on a silica gel column, removal of Boc group by TFA, and crystallization of the TFA salts (Supporting Information Section 4).

**Figure 3:**
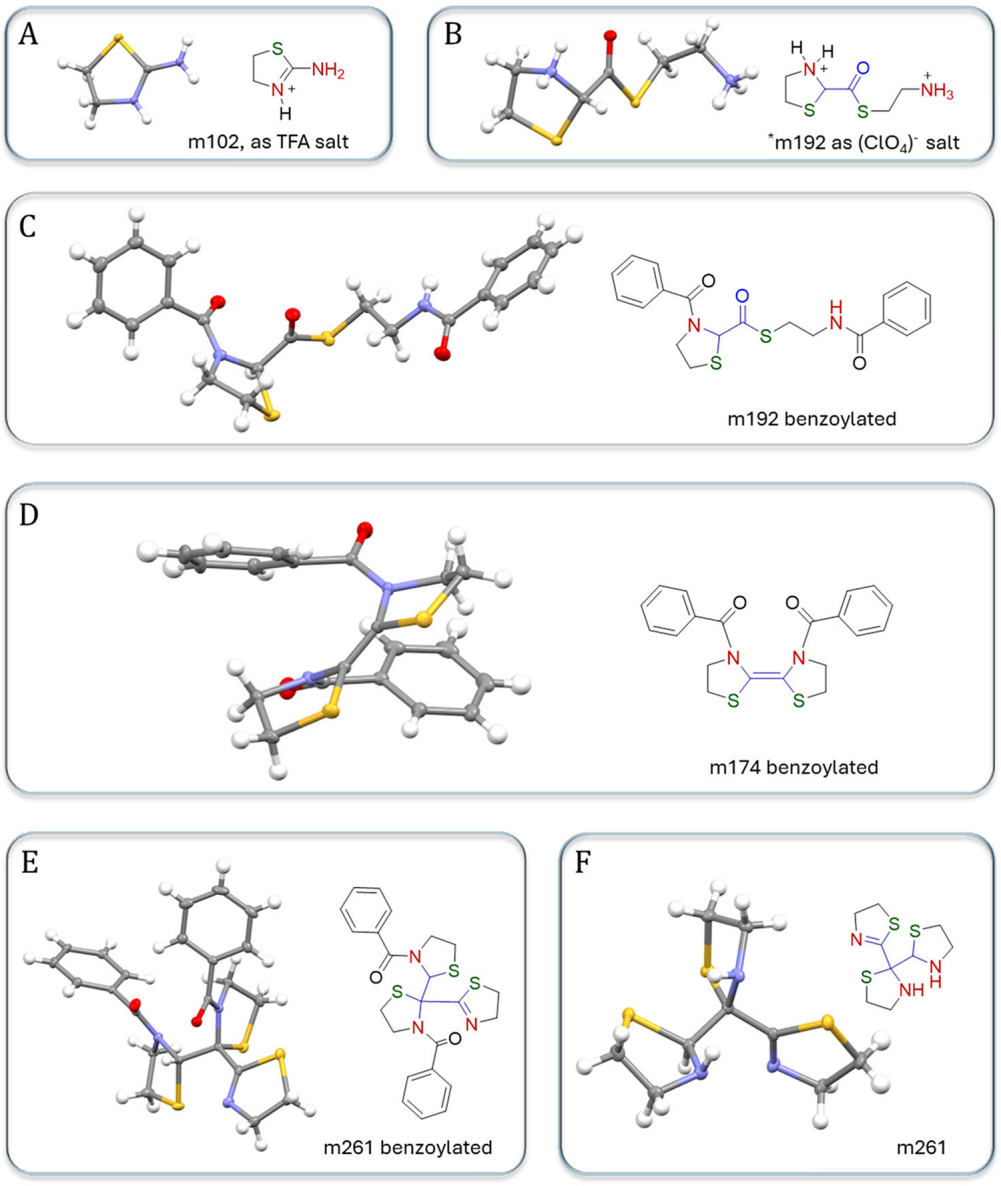
Single-crystal X-ray diffraction analysis of compounds. (A) Structure of m102 as a TFA salt. TFA^–^ counterion is omitted for clarity. (B) Structure of m192 as a perchlorate salt. ClO_4_^–^ counterion is omitted for clarity. (C–E) Structures of the benzoyl derivatives of m192, m174, and m261, respectively. (F) Structure of m261. All structures were drawn with 50% probability ellipsoids.

The lightest component detected by X-ray was 4,5-dihydrothiazol-2-amine (m102) as a TFA salt (Figure 3A). This compound most likely forms from cystamine (cysteamine disulfide) through the heterolytic cleavage of the disulfide bond with cyanide anion, followed by the intramolecular cyclization of 2-aminoethylthiocyanate. The thioester derivative (m192) was detected as both a perchlorate salt and a benzoyl derivative (Figure 3B,C), suggesting its abundance in the reaction mixture. This molecule is a derivative of HCN dimer; there are several possible pathways for its formation, all involving C–C bond formation between two carbons from HCN molecules and the addition of two cysteamine molecules (Figure 4). Interestingly, this molecule is essentially a thioester of an amino acid, which is a frequently discussed class of molecules in the context of the origin of life.^64,65^

**Figure 4:**
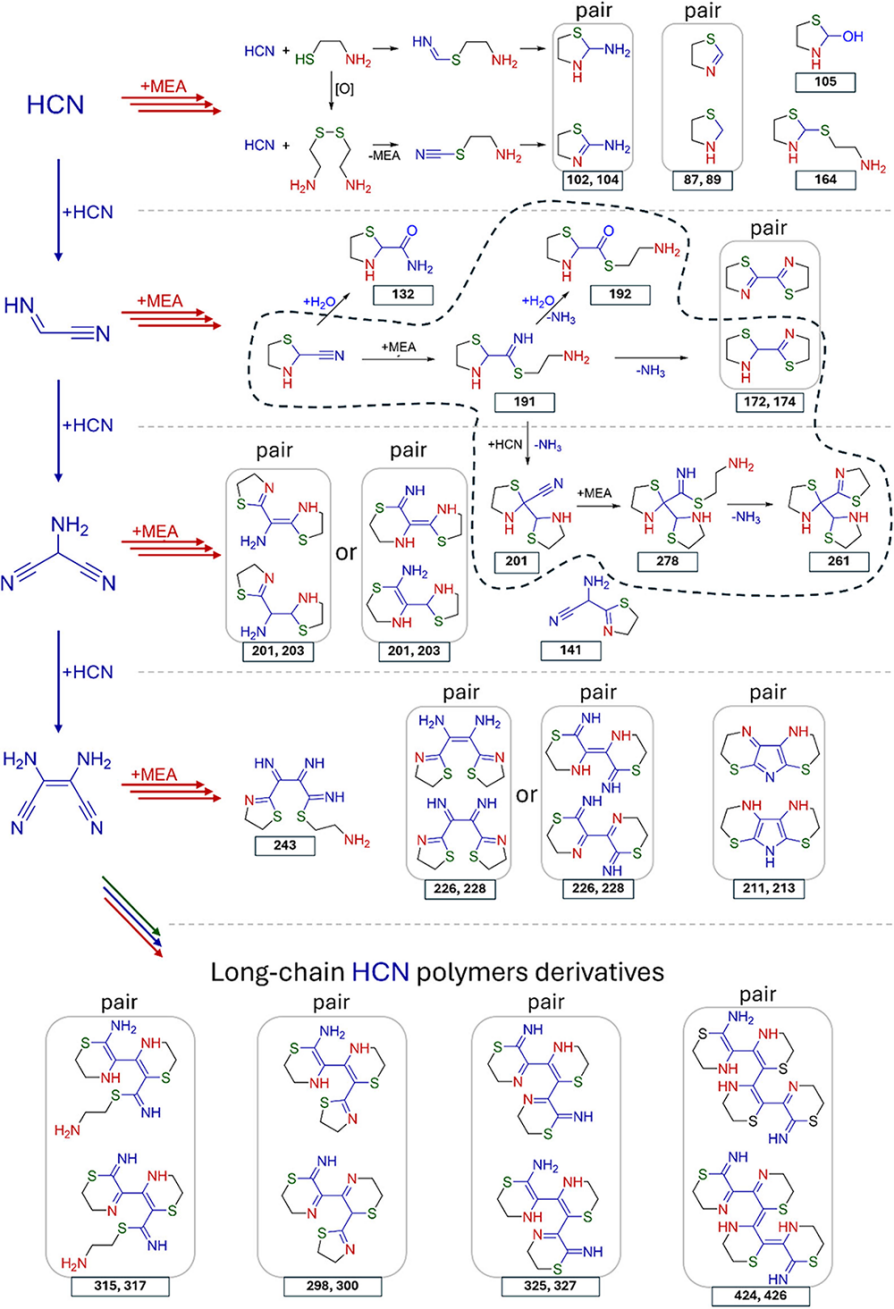
Proposed fragmented chemical reaction network originating from the reaction of HCN with cysteamine. Because of the inability to fully reconstruct the reaction network from our data, we have organized the proposed structures into rows based on the length of the carbon chain derived from HCN. Each row contains compounds that can be viewed as products of the addition of cysteamine to the corresponding HCN oligomer. However, we acknowledge that the actual sequence of HCN and cysteamine fragment additions can vary significantly between compounds. The part of the reaction network that is more reliably deduced based on the combination of X-ray, NMR, and MS data is highlighted by dashed line. Additionally, since many compounds appear as pairs in the MS spectra, these pairs have been highlighted in the scheme. All compounds are represented in their electrically neutral forms.

Another detected molecule containing two carbon atoms from HCN is the (Z)-4,4′,5,5′-tetrahydro-3H,3′H-2,2′-bithiazolylidene (m174), which crystallized as a benzoyl derivative (Figure 3D). NMR studies (Supporting Information Section 3g) indicate that, in solution, this molecule preferentially exists in its tautomeric form 2-(thiazolidin-2-yl)-4,5-dihydrothiazole. The pathway for m174 formation is likely similar to that of m192 but includes an extra cyclization step (Figure 4).

The only component of the reaction mixture with three carbon atoms from HCN characterized by X-ray was 2-(4,5-dihydrothiazol-2-yl)-2,2′-bithiazolidine (m261). This compound crystallized as both a neutral amine and a benzoyl derivative (Figure 3E,F), potentially indicating its abundance in the system. The compound can be viewed as a product of the addition of three cysteamine molecules to the HCN trimer. Interestingly, the structures m192, m174, and m261 can all be obtained from 2-cyanothiazolidine, whose presence was proposed based on NMR studies of the reaction’s early stages.

Based on the identified structures, the following key motifs can be proposed for this reaction: (i) cyanide attack on a molecule, forming a new nitrile group, (ii) thiolate attack on the resulting nitrile group, forming a thioamidine, and (iii) subsequent cyclization or hydrolysis, accompanied by ammonia release (Figure 4). The release of ammonia drives the pH toward more basic conditions. Ammonia can also be detected by the color change of wet pH paper exposed to the gases above the reaction mixture in a closed vial.

The availability of labeled cyanide sources (K^13^CN, KC^15^N) makes their use, in combination with natural isotope abundance cyanide, a powerful method for searching and identifying compounds in this reaction by mass spectrometry (MS) and NMR. ^13^C NMR spectra of the reaction mixture and of the second liquid phase obtained from K^13^CN indicate that major reaction products are derivatives of HCN dimer and large oligomers, because of dominant doublet signals in the low-field region (Supporting Information Section 3h). However, exact structural assignments and identification of minor reaction products remain challenging based on these data alone. Therefore, we concentrated our efforts on MS analysis (Supporting Information Section 5). To avoid synthesis of HCN from costly K^13^CN and KC^15^N for a series of experiments for MS analysis, we generated HCN in situ by combining equimolar quantities of KCN and an acid (HCl or KH_2_PO_4_) in each experiment. Control experiments showed that the MS spectra of reaction mixtures obtained from preprepared HCN and in situ generated HCN were very similar.

By recording MS spectra for reaction mixtures obtained using regular KCN, K^13^CN, and KC^15^N, we were able to determine not only the molecular weight of a compound associated with a specific MS peak, but also the number of carbon and nitrogen atoms originating from HCN. Specifically, the mass shift (in atomic mass units, au) of a peak in the mixture from the K^13^CN reaction compared to its position in the spectrum of regular KCN reaction gives the number of carbon atoms from HCN. Analogously, the shift in peak positions between spectra from regular KCN and KC^15^N gives the number of nitrogen atoms from HCN. Moreover, for reasonably small compounds (∼below 500 au), the number of cysteamine fragments can be calculated accurately.

To fully explore the chemical space of this reaction, we conducted experiments at four conditions differing by pH and the KCN/cysteamine ratio: (i) KCN (1M), MEA·HCl (1M), HCl (0.5M), pH 8.3 (ii) KCN (1M), MEA·HCl (0.5M), pH 9.4, (iii) KCN (1M), MEA·HCl (1M), KH_2_PO_4_ (2M), pH 6.7, (iv) KCN (1M), MEA·HCl (0.5M), KH_2_PO_4_ (2M), pH 6.75. No systematic variation of the MS signals across these experimental conditions was observed. Therefore, we combined all MS data, calculated the observation frequency of each compound (i.e., how often a component appeared across all experiments), and focused on compounds that were detected with sufficient frequency (Table 1).

A complex analysis of the MS data, combined with information from X-ray crystallography, ^13^C NMR, and computational screening of the chemical space (Supporting Information Section 8), allowed us to propose possible structures for the compounds listed in Table 1 (Figure 4). The compounds are organized according to the length of the carbon backbone from HCN. The exact pathways by which the proposed compounds are formed cannot be identified, but we propose that in most cases it likely involve sequential additions of HCN and cysteamine in varying orders. Moreover, the closure of 5- and 6-membered rings, imine hydrolysis, and some redox processes are likely necessary to account for the formation of all compounds within this network.

There are three aspects of the reaction network from Figure 4 that are worth discussing. First, we observe several precursors to the compounds identified by X-ray. For example, m192 is formed through the hydrolysis of m191, and m261 is formed by the closure of a 5-member ring in m278. The transformations associated with ammonia release during ring closure are evident in time-resolved experiments, where aliquots were taken at 30, 60, 120, and 300 min, as well as 1–2 days after the start of the reaction. In these experiments, the heavier signals (by 17 au) decrease over time, while the corresponding lighter signals increase (Supporting Information Section 5b). Second, although many of the observed masses can be explained by structures containing only 5-membered rings derived from cysteamine, some higher masses, particularly those above 300 au, require the incorporation of 6-membered rings into the structures. Third, MS data showed that many compounds in the system appeared in pairs with a mass difference of two units, which remained consistent in the ^13^C and ^15^N experiments, indicating that the compounds differ by two hydrogen atoms. Such pairs include m87/m89, m102/m104, m172/m174, m201/m203, m211/m213, m226/m228, m298/m300, m315/m317, m325/m327, m424/m426.

Except for the m102/m104 pair, where two plausible pathways can be easily drawn, the sources of the other pairs remain speculative. Direct two-electron proton-coupled oxidation or reduction reactions that would result in a ±2 au mass difference seem unlikely within the chemistry of this reaction network. Nevertheless, some pathways could potentially lead to compounds that are formally products of two-electron reductions or oxidations within the family of derivatives of HCN oligomers. For example, 2-aminoethylthiocyanate, which forms in a straightforward reaction between cystamine and cyanide,^66^ might react with a second cyanide before cyclizing to form m102. This reaction could give rise to a cyanogen family, with 2 fewer hydrogens than the HCN oligomer family, potentially explaining the formation of what appear to be formally oxidized products (e.g., m172). On the other hand, decarboxylation reactions could give rise to formally reduced products. For instance, the decarboxylation of the acid whose amide appears as m132 would result in m87.

Next, we computationally investigated whether a combination of reactions–including the additions of HCN and cysteamine, cyclizations, hydrolysis, as well as decarboxylation and cyanide addition to 2-aminoethylthiocyanate – could plausibly account for the formation of all proposed products. In analogy to retrosynthetic analysis, a predefined set of reaction operations was iteratively applied to the target compounds, to deconstruct them into their tentative building blocks (HCN, cysteamine, water). In the resulting reaction network, a stoichiometrically balanced pathway between the target compound and the building blocks, that minimizes the number of reactions, was determined via integer linear programming techniques, to corroborate a chemically plausible pathway in synthetic direction (Supporting Information Section 8).^48^ This analysis showed that all structures up to m327 can be constructed using the proposed set of reactions.

The ability to isolate crystalline m261 in its neutral form allowed us to test initiation of this autocatalytic reaction network with one of its specific products instead of using a product mixture. We conducted an experiment reacting HCN (∼1M) with MEA·HCl (2M) in the presence and absence of seed crystals of m261 (∼1.5 mol %). Due to the low solubility of m261, a suspension of ground crystals was used to seed the reaction. As expected from the basic amine nature of m261, ^1^H NMR kinetic monitoring showed a shorter lag phase in the seeded experiment, though the effect was moderate due to the limited amount of dissolved material (Figure 5A). More surprisingly, the seed crystals influenced the final product composition. This was immediately evident visually; the seeded reaction produced a large quantity of crystalline m261, in stark contrast to the dark-yellow, liquid second phase formed in the unseeded control experiment (Figure 5B).

**Figure 5:**
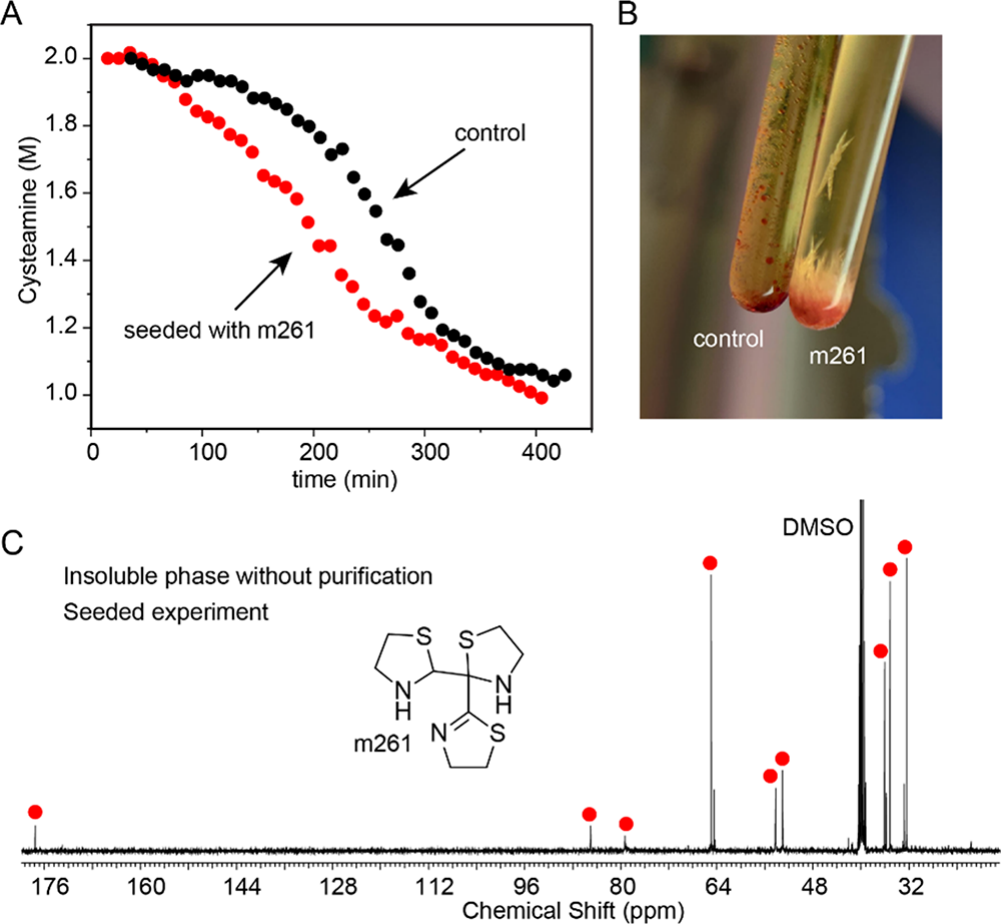
Seeding of the reaction between HCN (∼1M) and MEA·HCl (2M) with m261. (A) ^1^H NMR kinetic plot showing changes in cysteamine concentration in seeded and control experiments. Cysteamine concentration was calculated using *tert*-butanol internal standard. (B) Visual difference between seeded and control experiments after reaction completion. (C) ^13^C NMR spectrum of the unpurified precipitate in the seeded experiment. Red circles indicate signals belonging to m261. For ^1^H spectra and comparison with the control experiment, see Supporting Information Section 3k.

To quantify this difference, we separated the water-insoluble phases and analyzed them by NMR in DMSO-d^6^. The analysis revealed that the solid precipitate from the seeded experiment was approximately 80% pure m261, whereas the second liquid phase from the control experiment contained only about 30% m261 (Figure 5C and Supporting Information Section 3k). Furthermore, analysis against an internal standard and similar composition of the remaining aqueous phases in both experiments indicated a larger absolute amount of m261 formed in the seeded experiment (Supporting Information Section 3k). Such templating would be expected in a network of dynamic, reversible reactions where crystallization simply shifts the equilibrium. However, its occurrence is not obvious in a system like this, where many steps appear irreversible and overall transformation has large thermodynamic driving force. Therefore, the ability to direct this complex reaction network toward a specific product is an interesting question for future investigations.

In summary of this section, the reaction between HCN and MEA·HCl yields a wide variety of compounds. Some of these compounds, such as the amino acid thioester m192, could serve as precursors for functional polymers. Most of the identified compounds contain secondary or tertiary amines–for example, compound m261 contains both types. The localized concentration of these amines within the second liquid phase likely enhances its catalytic activity relative to the bulk solution and increases the local pH (within the phase and its immediate vicinity), thus explaining this phase’s role in the previously discussed autocatalysis. Moreover, the results indicate that crystalline m261 can direct the reaction network toward its own formation.

### Second Liquid Phase as a Compartment

The phase separation process in the HCN/MEA·HCl reaction concentrates hydrophobic products into the second liquid phase. We hypothesized that this phase could also absorb hydrophobic molecules from the solution that are not part of the HCN/MEA·HCl reaction, thereby acting as a compartment that could potentially facilitate new chemistry.

To investigate the potential for incorporating molecules into the second phase, we selected several compounds with varying LogP values, a measure of the distribution of a substance between water and lipid phases. The selected molecules were benzene (LogP: 2.13), cyclohexanol (LogP: 1.23), isopentenyl alcohol (LogP: 1.1) and adenine (LogP: −1.15). We conducted ^1^H NMR kinetic studies of the reaction between HCN (2M) and MEA·HCl (2M) in the presence of various amounts of these compounds. Since the second liquid phase precipitates at the bottom of the NMR tube, a decrease in the ^1^H NMR signal of the inert molecule indicates its absorption by the second liquid phase.

Benzene stood out from the other tested molecules, because it combines high lipophilicity (LogP: 2.13) and water solubility (∼2 g/L) sufficient to clearly detect its sharp signal from six identical protons. In our experiments, benzene’s concentration in the aqueous phase dropped more than 4-fold during the formation of the second liquid phase (Figure 6A). Simultaneously with the decrease in intensity of the main benzene’s signal, a broadened twin signal appeared in the upfield region of the NMR spectrum (Figure 6B). This new signal likely corresponds to benzene within the droplets of the second liquid phase before their sedimentation at the bottom of the tube, outside of the detection region.

**Figure 6:**
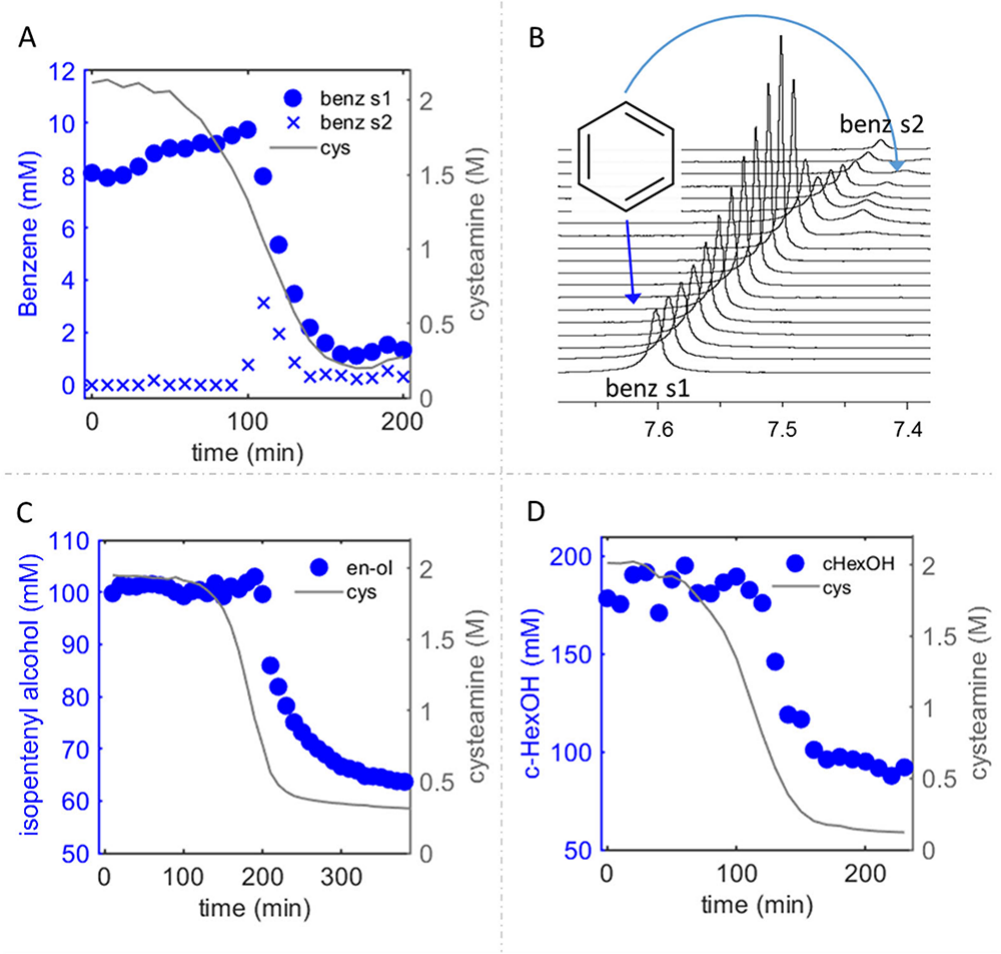
Droplets of the second liquid phase as hydrophobic compartments. (A) ^1^H NMR kinetic plot showing changes in concentrations of cysteamine and benzene in the reaction of HCN (2M) and MEA·HCl (2M) in the presence of the saturated solution of benzene (about 0.2 wt %). Benzene was monitored in both the solution phase (benz s1) and the second liquid phase (benz s2). (B) Time evolution of the benzene signal in ^1^H NMR experiment from panel A. Appearance of the satellite signal (benz s2), which we assign to benzene in the second liquid phase, is clearly visible here. (C) ^1^H NMR kinetic plot showing changes in concentrations of cysteamine and isopentenyl alcohol (enol) in the reaction of HCN (2M) and MEA·HCl (2M) in the presence of isopentenyl alcohol (100 mM). (D) ^1^H NMR kinetic plot showing changes in concentrations of cysteamine and cyclohexanol (c-HexOH) in the reaction of HCN (2M) and MEA·HCl (2M) in the presence of cyclohexanol (200 mM).

Isopentenyl alcohol and cyclohexanol, both moderately lipophilic, also demonstrated a significant shift into the second liquid phase. The more lipophilic cyclohexanol (LogP 1.23) migrated about 50% (Figure 6D), while the less lipophilic isopentenyl alcohol (LogP 1.1) migrated about 35% (Figure 6C). The changes in the signals of the alcohols lagged behind the changes in the cysteamine signal, likely due to the delay between the consumption of cysteamine in reactions and the onset of the phase separation. In addition, the previously observed delay in the onset of autocatalysis in the presence of isopentenyl alcohol (see Figure 2A) was confirmed. Specifically, the lag phase for reactions in the presence of benzene and cyclohexanol was about 70 min, whereas it extended to approximately 150 min with isopentenyl alcohol.

Incorporation of the alcohols into the second liquid phase changed its consistency and color, turning it from red to yellow-orange and reducing its viscosity. The lowered viscosity might enhance the second liquid phase’s capacity to act as a solvent for other reactions. In the case of the incorporation of isopentenyl alcohol, the surface of the second liquid phase became a site for the growth of crystals of compound m261, providing the most direct example of the structure of molecules within the second phase.

Adenine, being a much more hydrophilic molecule (LogP = −1.15), did not show any significant change in its concentration in water during the formation of the second phase (Supporting Information Section 3f). This observation, alongside the findings from the other tested compounds, supports the expected trend that the degree of incorporation into the second liquid phase is proportional to the compounds’ LogP values.

## Conclusions

This research demonstrates that HCN can autocatalytically co-oligomerize with cysteamine, leading to the formation of liquid droplets capable of compartmentalizing moderately hydrophobic molecules. The autocatalysis observed in this process is generally nonspecific and is driven by an increase in the basicity and nucleophilicity of the reaction mixture, particularly by an increase in concentration of thiolate species. Interestingly, the reaction starts with the formation of derivatives of the HCN dimer instead of the expected dominance of formic acid derivatives at the initial stages. These findings indicate that thiols induce C–C bond formation between HCN-derived fragments. Additionally, preliminary results indicate that this reaction network can be directed toward the formation of specific products using crystalline seeds.

It is useful to discuss these results in the context of the questions posed in the introduction: How do metastable prebiotic mixtures release their chemical potential? And how do these mixtures evolve when additional reactants are supplied? The metastability of prebiotic (or other) mixtures of reactants often arises from the absence of certain reactivity modes. For instance, in the system presented in this study, there is a lack of strong nucleophiles in a weakly acidic environment. The reaction generates nitrogen bases, which in turn produce a highly nucleophilic thiolate that drives the autocatalysis. A parallel example can be found in the formose reaction, where the absence of α-CH in formaldehyde prevents polycondensation until glycolaldehyde is formed.^22,23^ Another interesting parallel with the formose reaction is the existence of a bottleneck at the stage of coupling of two C1 building blocks (CH_2_O or HCN), after which further oligomerization proceeds more readily in both cases.

When considering typical prebiotic building blocks, many exhibit predominantly electrophilic behavior in the weakly acidic conditions of early Earth.^67^ In contrast, prebiotic synthesis is often favored in basic environments.^5^ Hence, we can argue that the formation of local autocatalytic environments through basification mechanisms–like the one observed in this study–could have occurred under prebiotic conditions. Additionally, we can speculate that the system might exhibit more complex behaviors, such as bistability and oscillations. This could occur if some amine-inactivating reactions, including ester hydrolysis and aminolysis, are introduced into the medium in flow conditions.

A second key aspect of the studied reaction is the formation of a liquid phase capable of concentrating certain hydrophobic molecules, some having low water solubility. Importantly, the formation of a distinct liquid phase through the oligomerization of small, fully water-soluble molecules is a relatively rare phenomenon that has attracted the interest of researchers in the earliest studies on the origin of life.^68^ Perhaps the best-known example of this phenomenon is the reaction between formaldehyde and ammonium thiocyanate.^68,69^ Such two-phase systems can bring together otherwise incompatible reactants and thereby significantly expand the repertoire of potential chemical reactions. Interestingly, many cofactors (such as flavin, thiamine, and heme) have reactive components that are poorly soluble in their pure form.^70^ In modern cells, they are typically modified with phosphate or ribophosphate groups, which enhance their solubility. However, during the early stages of chemical evolution, such complex chemistry was not yet available. The formation of compartments capable of solubilizing cofactor-like molecules and catalysts could significantly expand the chemistry of prebiotic reaction networks and may represent a crucial factor in the evolution of prebiotic mixtures when a continuous supply of reactants is present.

Another notable observation is that the copolymerization of HCN with cysteamine largely prevents the formation of insoluble black precipitates, instead producing medium-sized heterocyclic compounds. It is plausible that the copolymerization of high-energy prebiotic building blocks (such as HCN and cyanogen) with other simple molecules could provide a pathway to generate complex, nonpolymeric structures. However, further investigation is needed to determine the generality of this phenomenon.

## Supplementary Material



## Notes

Detailed procedures for HCN synthesis, kinetics experiments, NMR, and MS analysis (PDF)
