# Supplementary material for: Autocatalytic Reaction between HCN and Cysteamine Produces Hydrophobic, Catalytic, Liquid Compartments
Source: J Am Chem Soc. 2025 Sep 3;147(37):33711–22. doi: 10.1021/jacs.5c09581 (PMC12447481; doi:10.1021/jacs.5c09581)
Supplement: Supplementary file 1 [file ja5c09581_si_001.pdf]

Supporting information for:

Autocatalytic Reaction between HCN and Cysteamine Produces Hydrophobic, Catalytic, Liquid Compartments

Alexander I. Novichkov,[a] Yael Diskin-Posner,[b] Linda J. W. Shimon,[b] Gregory Leitus,[b] Christoph Flamm,[c] and Sergey N. Semenov[a]*

[a] Department of Molecular Chemistry and Materials Science, Weizmann Institute of Science, Herzl Street, Rehovot 7610001, Israel.

[b] Department of Chemical Research Support, Weizmann Institute of Science, Herzl Street 234, Rehovot 7610001, Israel.

[c] Department of Theoretical Chemistry, University of Vienna, Währinger Strasse 17, Vienna 1090, Austria.

*Correspondence to: sergey.semenov@weizmann.ac.il

Table of Contents

Materials and Methods 4

1. Synthesis and handling precautions for hydrogen cyanide (HCN) 5

   a) General safety precautions for handling hydrogen cyanide 5

   b) Synthesis of anhydrous hydrogen cyanide 6

   C) Purity, storage, and handling notes 10

2. Experimental setup and protocols for HCN – cysteamine reaction with visual control 11

   a) General protocol and visual monitoring of HCN reactions 11

   b) 1M HCN + 1M cysteamine*HCl, 25°C General Experiment (Fig. 1A) 11

   c) 2M HCN + 2M cysteamine*HCl, 25°C experiment (Fig. 2A) 11

   d) 2M HCN + 2M cysteamine*HCl, 25°C experiment (Fig. 2B) 12

   e) Visual monitoring of HCN-cysteamine reaction in buffered solutions at various pH values 12

   f) Comparative visual monitoring of HCN reactions with cysteamine and its analogs 14

   g) Visual monitoring of the HCN-cysteamine reaction in the presence of malononitrile 16

3. NMR Experiments 17

   a) Standard Addition Experiment (Fig. 1B), 2M HCN + 2M cysteamine*HCl, 40°C 18

   b) NMR experiment with benzene (Fig. 4A, B), 2M HCN + 2M cysteamine*HCl, 40°C 20

   c) NMR experiment with triton X-100 (Fig. 2 C), 2M HCN + 2M cysteamine*HCl, 40°C 21

d) NMR experiment with isopentenyl alcohol (Fig. 4 C), 2M HCN + 2M cysteamine*HCl, 40°C 22

e) NMR experiment with cyclohexanol (figure 4 D), 2M HCN + 2M cysteamine*HCl, 40°C 23

f) NMR experiment with adenine, 2M HCN + 2M cysteamine*HCl, 40°C 24

g) NMR studies of m174 26

h) NMR experiment $K^{13}CN$ with cysteamine under ^{13}C NMR control 29

i) ^{1}H NMR kinetic study of HCN- cysteamine reaction initiated by KOH 32

j) ^{1}H NMR kinetic study of HCN-cysteamine reaction in phosphate buffer (pH 6.5) 33

k) Seeding experiments with m261 35

4. Isolation and structural analysis of products by X-ray crystallography 41

a) Single-crystal X-ray diffraction analysis of compounds. 41

b) Formation of m261 in experiment with isopentenyl alcohol 44

c) Isolation m192 using $HClO_4$ treatment of the second liquid phase 44

d) Benzoylation of the second liquid phase: formation and isolation of m192benzoylated, m174benzoylated, and m261benzoylated. 45

e) Boc protection, chromatography, and deprotection of the second phase to isolate m102 46

5. Mass spectrometry analysis of HCN-cysteamine reactions: techniques and methods 47

a) Selected experiments with isotopically labeled cyanides 47

b) Signal recognition and triplet identification 48

c) Mathematical framework for data analysis 56

d) Data processing and analysis using MATLAB and IVO algorithm 58

6. UV-Vis experiments 64

a) **Hydrolysis rate of PNPA in the presence of the second liquid phase**......... 64

b) Continuous pH monitoring and thiol concentration measurement in HCN-cysteamine reaction.. 66

7. DLS and microscopy characterization of the second liquid phase 68

a) DLS studies of the formation of the second liquid phase. 68

b) Light microscopy studies of the second liquid phase. 69

8. Computational Deconstruction 71

9. Appendix S1: Raw NMR integral data for kinetic experiments 86

10. Appendix S2: Matlab scripts and framework for mass spectrometry data analysis and compound identification.. 92

1. Core principles of the analytical approach... 92

2. Workflow for data analysis 93

3. MATLAB Scripts.. 95

4. Note on workflow optimization 101

10. Appendix S3: Computational deconstruction pathways for selected compounds 102

Materials and Methods

Benzoyl chloride (PhCOCl), di-tert-butyl dicarbonate (Boc_2O), pyridine (Py), Triton X-100, cyclohexanol, benzene, potassium cyanide (KCN), cysteamine hydrochloride (MEA·HCl), monopotassium phosphate (KH_2PO_4), dipotassium phosphate (K_2HPO_4), and hydrochloric acid (HCl) were purchased from Sigma-Aldrich, Acros Organics, Alfa Aesar, and Merck. Solvents, including dichloromethane (DCM), methanol, ethanol, n-hexane, ethyl acetate, acetone, and anhydrous acetonitrile, were purchased from Sigma-Aldrich and Acros Organics.

Deuterium oxide (D_2O) and all other NMR solvents were purchased from Tzamal d-chem. Potassium-^{13}C cyanide ($K^{13}CN$, 99% enrichment) and potassium-^{15}N cyanide ($KC^{15}N$, 99% enrichment) were obtained from Cambridge Isotope Laboratories, Inc. All chemicals, including solvents, were used without further purification. LC-MS grade water was used for all reactions conducted in water. Hydrogen cyanide (HCN) was prepared according to a literature procedure.[1]

NMR spectra were measured on a Bruker AVANCE III-300 spectrometer at 300 MHz for ^{1}H and 73.7 MHz for ^{13}C{1H}, on a Bruker AVANCE III-400 spectrometer at 400 MHz for ^{1}H and 100.6 MHz for ^{13}C{1H}, and on a Bruker AVANCE III HD-500 spectrometer at 500 MHz for ^{1}H and 125.8 MHz for ^{13}C{1H}. Chemical shifts for ^{1}H is given in ppm relative to TMS.

The absorbance was measured on a Cary 60 UV-VIS spectrometer, manufactured by Agilent Technologies.

A Metrohm 913 pH meter was used for pH measurements, calibrated according to the standard procedure prior to use.

The chromatographic separation and mass analysis were performed on a Waters Acquity liquid chromatography system equipped with a PDA Detector (210 to 700 nm) and a

[1] Vogel, A. I., *Vogel's Textbook of Practical Organic Chemistry*, 5th ed., Furniss, B. S., et al. (Eds.), Longman Scientific & Technical; Wiley, 1989, p 439.

Waters QDa mass detector with an electrospray ionization (ESI) and a mass range of 85–1250 m/z. For experiments involving chromatographic separation, a Jupiter 5 µm C4 300 Å LC Column (50 x 4.6 mm) was used.

1. Synthesis and handling precautions for hydrogen cyanide (HCN)

a) General safety precautions for handling hydrogen cyanide

!Extreme Hazard Warning!: Hydrogen cyanide is an extremely toxic and volatile liquid (b.p. 26 °C) that is rapidly fatal by inhalation, ingestion, and skin absorption. All operations involving HCN must be meticulously planned and performed exclusively in a certified, high-performance chemical fume hood.

Engineering controls and monitoring: All work with HCN was conducted in a designated laboratory area equipped with two personal HCN gas detectors (UNI MP100, mPower Electronics (Shanghai) Co., Ltd., with an HCN sensor range of 0–100 ppm). One detector was worn by the operator on the lab coat, and a second stationary detector was placed adjacent to the fume hood where the synthesis and handling procedures were carried out to monitor ambient HCN levels. Anhydrous HCN was stored at -20 °C in a sealed Schlenk flask. This freezer was equipped with an independent defrost alarm system monitored by the Weizmann Institute's Safety Unit, which was fully informed prior to the commencement of any work involving HCN.

Administrative controls and personal protective equipment (PPE): All procedures involving HCN were performed ensuring that a second qualified person was present in the laboratory and aware of the ongoing work, allowing for immediate assistance in case of an emergency. Thorough planning and proper workspace organization within the fume hood are critical. It is advisable to place balances and other necessary equipment directly inside the fume hood during work with HCN. Given HCN's ability to readily penetrate many common glove materials, including standard nitrile gloves which offer only limited

protection, careful selection of appropriate chemical-resistant gloves is crucial. Thicker butyl rubber gloves or specialized laminate gloves are generally recommended.

b) Synthesis of anhydrous hydrogen cyanide

Anhydrous HCN was prepared based on the procedure described by Vogel with several important modifications implemented to enhance operational safety, control, and the efficiency of HCN collection. The primary motivation for these modifications was to address the practical limitations of certain apparatus described in the reference literature, which could be cumbersome and potentially lead to inefficient HCN evolution or losses. Our redesigned setup aimed to ensure a controlled, unidirectional flow of HCN from the generation flask directly to the collection trap under a gentle argon stream. A photograph of the custom-built apparatus with key components annotated is shown in Figure 1.1.

Procedure: The synthesis was typically performed using solid potassium cyanide (KCN, 50.0 g, 0.768 mol) as the HCN precursor. Sulfuric acid (125 mL of a 50% v/v aqueous solution, prepared by carefully adding concentrated H_2SO_4 (98%,) to deionized water with cooling) was used for the liberation of HCN, providing a ~1.5x molar excess of H_2SO_4 (~1.1-1.2 mol).

The reaction was carried out in a 250 mL three-neck round-bottom flask equipped with a magnetic stirrer, a pressure-equalizing dropping funnel for H_2SO_4 addition, and an argon inlet/gas outlet adapter. Solid KCN was placed in the flask, chosen for its advantage over aqueous cyanide solutions by providing a higher reactant concentration, thus simplifying HCN evolution and minimizing the need for distillation from a dilute aqueous phase. The flask was immersed in a water bath maintained at approximately 50 °C. A continuous gentle stream of dry argon (flow rate adjusted to approx. 1 bubble every 1-2 seconds through the downstream scrubbers) was passed through the flask throughout the reaction. This argon flow was crucial for maintaining a steady, unidirectional transport of HCN gas, preventing pressure fluctuations and potential suck-back of scrubber solutions, and ensuring efficient entrainment of the generated HCN. Sulfuric acid was added dropwise from the funnel to the stirred KCN over a period of 2.5-3 hours, controlling the addition

rate to avoid excessive foaming or temperature increase due to the initial exothermic reaction upon contact with solid KCN. While minor charring within the reaction flask was occasionally observed under these conditions, the collected HCN was found to be of high purity after cryogenic trapping.

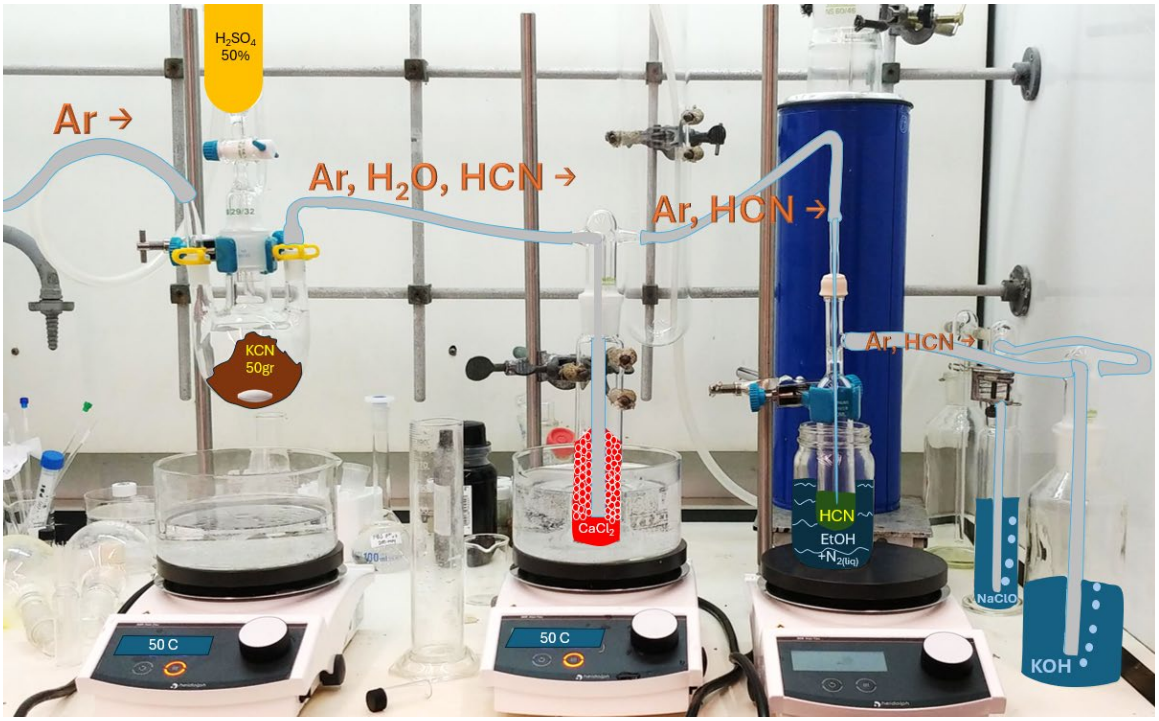

Figure 1.1 Custom-built apparatus for the laboratory synthesis of anhydrous HCN, showing the reaction flask, drying tube, cryogenic collection trap, and neutralization scrubbers.

The effluent gas stream (Ar, HCN, and water vapor) from the reactor was passed through a trap packed with anhydrous calcium chloride ($CaCl_2$) granules. This drying tube was also maintained at approximately 50 °C in a water bath. This temperature ensures efficient HCN volatilization from the reactor and prevents its premature condensation within the trap, while facilitating moisture removal.

The dried gas stream (Ar + HCN) was then directed into a specialized collection flask (e.g., a heavy-walled test tube or Schlenk flask, typically 50 mL capacity, see example in Figure 1.2) fitted with a septum. The gas inlet was achieved using a modified Pasteur pipette with a narrowed tip (drawn out carefully, ensuring the opening was not excessively constricted) that penetrated the septum and extended into the cold zone of the flask

(optimally to about mid-depth, as shown schematically in Figure 1.2). The flask outlet, via a side arm, was connected to the neutralization scrubbers.

The choice of collection temperature involves practical trade-offs. While collecting HCN as a liquid (e.g., using an ice-salt bath at -5 to -10 °C) minimizes the risk of the inlet tube being blocked by solid HCN, it leads to higher vapor pressure and thus greater product loss into the effluent gas stream. Conversely, cryogenic trapping (e.g., using an ethanol/liquid nitrogen bath at approx. -78 °C or lower) ensures very efficient condensation and collection due to the low vapor pressure of solid HCN, and the bath temperature is relatively easy to maintain. However, this method requires careful monitoring of the gas flow and the state of the inlet pipette tip to prevent blockage by solid HCN accumulating near the cold inlet.

Anhydrous HCN condensed efficiently as a colourless solid on the cold walls of the flask immersed in the ethanol/liquid nitrogen bath. The typical yield of anhydrous HCN collected per synthesis run from 50 g of KCN was estimated to be 20-25 mL (approximately 14-17 g, representing a 65-85% yield based on KCN).

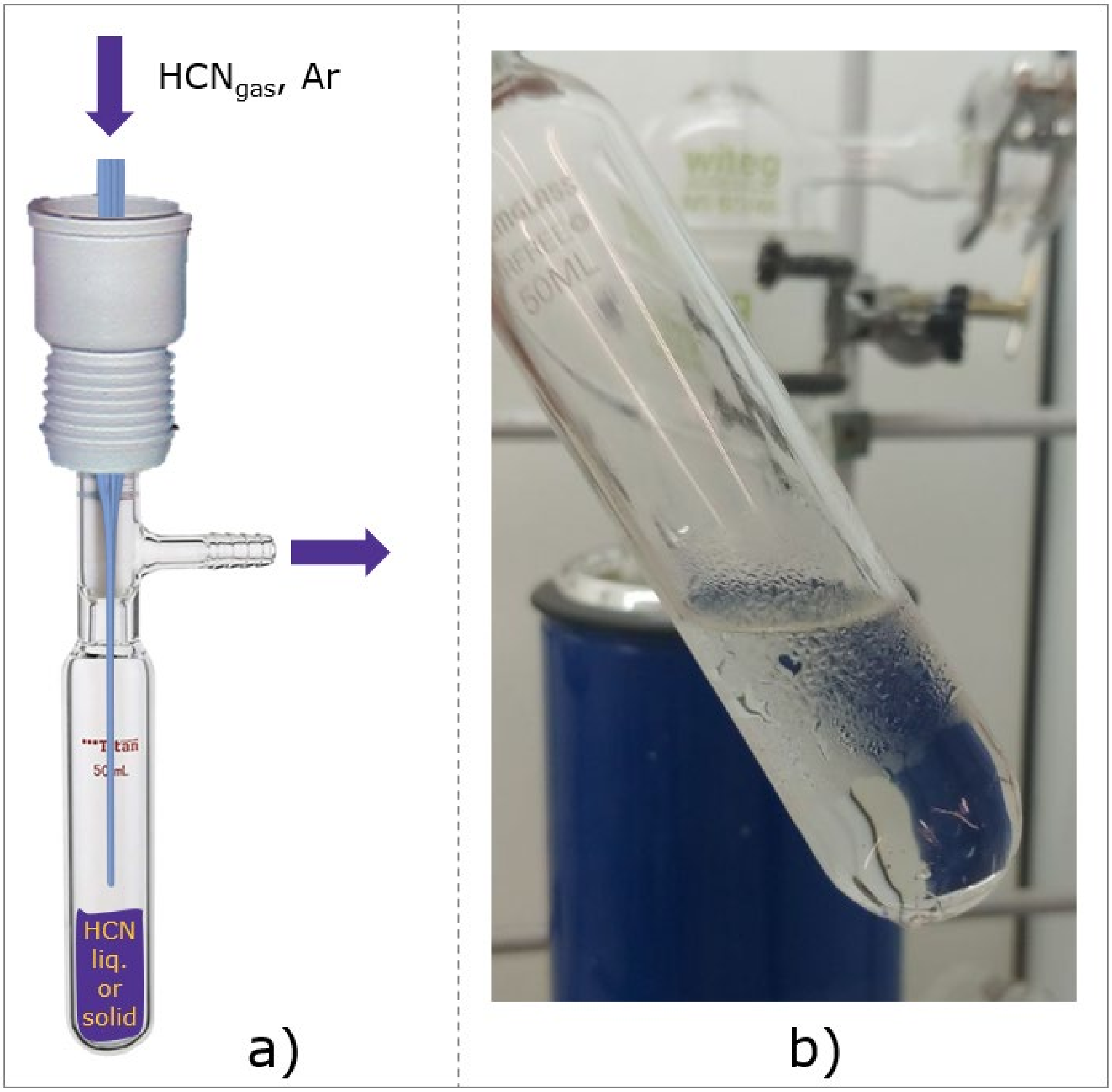

Figure 1.2 Collection of anhydrous hydrogen cyanide. (a) Schematic diagram of the HCN collection assembly. The dried gas stream (HCN and Ar) enters through a modified Pasteur pipette with a narrowed tip, which penetrates a septum sealing the collection flask (e.g., a 50 mL heavy-walled tube or Schlenk flask). HCN condenses as a liquid or solid on the cold walls of the flask, which is immersed in a cryogenic bath. The effluent gas is directed to neutralization scrubbers via the side arm. **(b)** Photograph of liquefied anhydrous HCN (approximately 20-25 mL) collected in a 50 mL tube from a typical synthesis run (starting from 50 g KCN), shown after thawing the cryogenically trapped solid.

The non-condensable gases exiting the cryogenic trap were passed sequentially through two safety scrubbing bottles to rigorously remove any residual HCN and acidic vapors. The first trap contained an aqueous solution of potassium hydroxide (KOH, ~10% w/v or

~2 M), followed by a second trap containing an aqueous solution of sodium hypochlorite (NaClO, ~5% active chlorine).

C) Purity, storage, and handling notes

The anhydrous HCN collected via cryogenic trapping typically appeared as elongated colorless crystals when frozen, condensing into a clear, colorless liquid upon thawing (Figure 1.2 b). The material obtained directly from the trap was generally used for subsequent experiments without further purification.

For storage, the anhydrous HCN was kept in a sealed Schlenk flask at -20 °C in the designated, monitored freezer (see Section 1.1). Storage under an inert atmosphere overlay (e.g., argon) was found to be unnecessary for maintaining stability under these conditions for periods up to one year.

It is advisable to carefully consider the setup geometry and materials used for connecting tubing. While glass tubing is preferred where possible, if flexible tubing (e.g., silicone or Tygon) is used, configurations where liquid HCN might pool or condense should be avoided. On one occasion, such pooling in a section of Tygon tubing led to leaching of impurities, which manifested as turbidity upon dissolving the collected HCN in water. In that specific instance, the contaminated batch was purified by standard vacuum transfer into a clean, pre-cooled Schlenk flask prior to use. Solutions prepared from routinely synthesized HCN were clear.

2. Experimental setup and protocols for HCN – cysteamine reaction with visual control

a) General protocol and visual monitoring of HCN reactions

The standard experiment follows these steps: Freshly prepared, cooled hydrogen cyanide solution is added to cysteamine, after which the vial is placed in front of a camera, and photos are taken at specific intervals for subsequent analysis. In cases where multiple experiments were conducted in parallel, the same reaction mixture was distributed across vials. A Linux-based single-board computer, Raspberry Pi 3b, and a Logitech camera (Logitech Brio Ultra HD Pro Business Webcam 960) were used to capture the photos. The images were collected using a script.

b) 1M HCN + 1M cysteamine*HCl, 25°C General Experiment (Fig. 1A)

For this experiment, liquid HCN was added to pre-cooled water, followed by dilution to achieve a 1M concentration of HCN. A measured amount of cysteamine hydrochloride was then added just before the start of the experiment to achieve a final concentration of 1M for both reactants. The vial was then placed at room temperature in front of a camera, and photos were taken at specific intervals for subsequent analysis.

c) 2M HCN + 2M cysteamine*HCl, 25°C experiment (Fig. 2A)

The reaction was conducted according to the standard protocol. The concentrations of cysteamine and hydrogen cyanide were increased to 2M to reduce the experiment duration. Three vials were each filled with 5 mL of the cysteamine and hydrogen cyanide mixture. To one vial, 0.5 mmol of isopentenyl alcohol (3-methylbut-2-en-1-ol) (51 µL) was added. Another vial received 0.5 mmol of Triton X-100 (325 mg). The third vial was used as a standard for subsequent comparative analysis.

d) 2M HCN + 2M cysteamine*HCl, 25°C experiment (Fig. 2B)

In this experiment, 5 mL of the cysteamine and hydrogen cyanide solution was combined with the following amounts of ion-exchange resins: (a) 10 mg of DE 52 (base resin), (b) 10 mg of MB-1 (neutral), and (c) 10 mg of Dowex 1x200/400 (acid). A blank sample was also included in this experiment and exhibited similar time-dependent changes as observed in the previous experiment (Fig. 2A).

e) Visual monitoring of HCN-cysteamine reaction in buffered solutions at various pH values

To assess the influence of initial pH on the visual progression of the reaction, experiments were conducted in four different buffer systems. The reaction mixtures, containing HCN generated *in situ* from KCN, cysteamine hydrochloride, and the respective buffer components, were prepared as follows to ensure precise initial pH control. This method, where HCN is generated directly in the buffered cysteamine solution, was chosen for enhanced safety in preparation and greater accuracy in achieving the target pH values compared to mixing pre-formed buffers with separately prepared HCN solutions.

Preparation of Reaction Mixtures: For each of the four conditions, cysteamine hydrochloride (1.136 g, 10 mmol) and potassium cyanide (KCN, 650 mg, 10 mmol) were placed in a vial. Approximately 5 mL of deionized water was added, followed by the respective acid for the buffer system:

For acetate buffers: Acetic acid (572 µL, 0.60 g, 10 mmol) was added. The pH of the resulting solution (initially ~6.4) was then carefully adjusted downwards to the target pH of 5.0 or 4.0 using a 5 M HCl solution, with continuous monitoring via a calibrated pH electrode.

For phosphate buffers: Phosphoric acid (890 µL of a 70% H_3PO_4 solution, corresponding to ~10 mmol H_3PO_4) was added. The pH was then adjusted upwards from the initial acidic value to the target pH of 6.5 or 7.5 using a 4 M KOH solution, with continuous monitoring via a calibrated pH electrode.

All solutions were prepared sequentially, typically starting from the lowest target pH to the highest for each buffer type. After the final pH adjustment, each solution was diluted with deionized water to a final volume of 10 mL. This resulted in reaction mixtures containing 1 M HCN (from KCN), 1 M cysteamine hydrochloride, and 1 M of the principal buffer species (acetate or phosphate) at the desired initial pH. The vials were maintained at 25 °C and visually monitored. Photographic records and, for the pH 5.0 solution, colorimetric analysis were performed at selected intervals

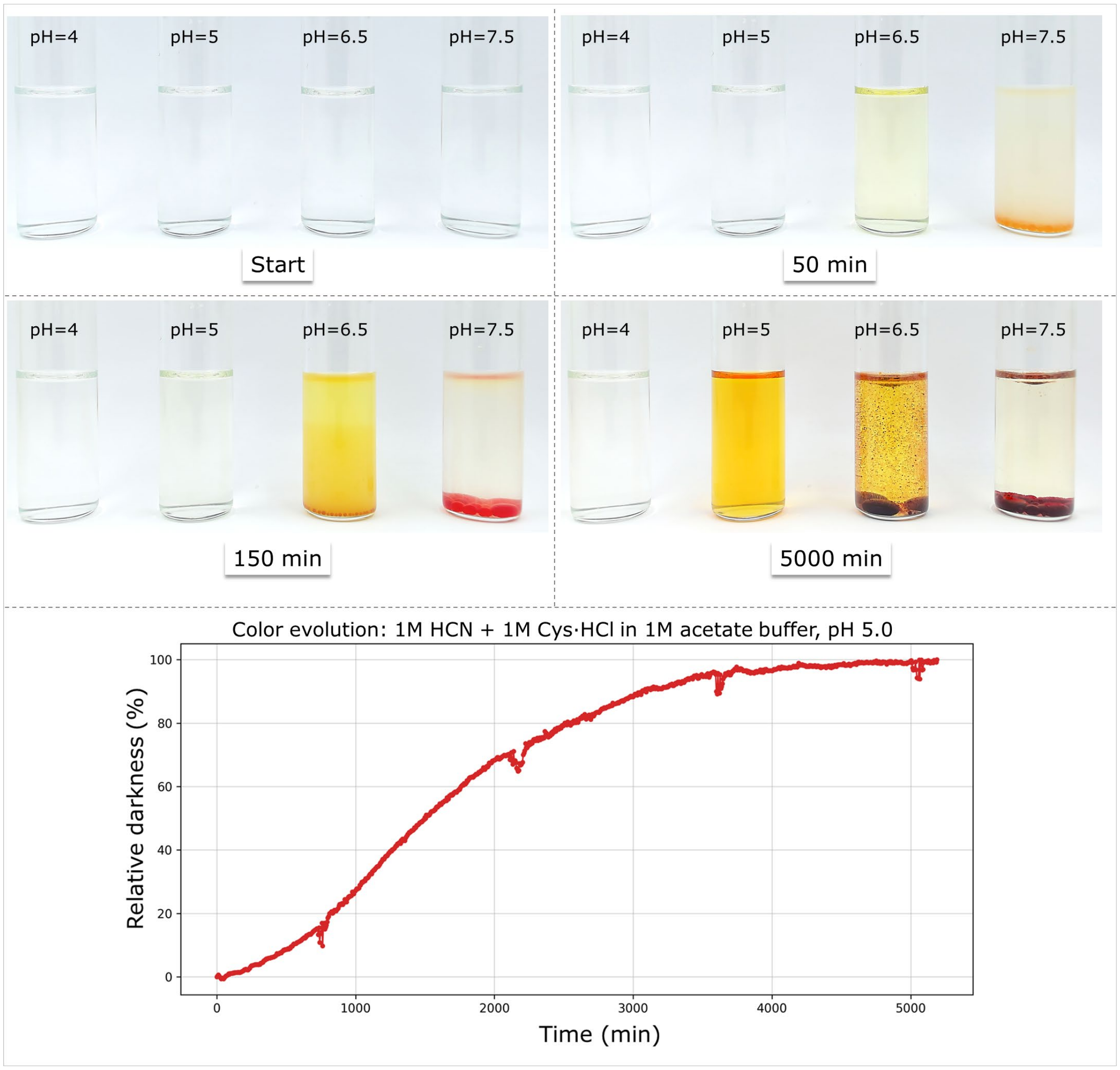

Figure 2.1. Visual progression and colorimetric analysis of the reaction between HCN (1 M) and cysteamine hydrochloride (1 M) in 1 M buffered solutions at different initial pH values. Top panels: Vials containing the reaction mixtures at pH 4.0 (acetate), pH 5.0 (acetate), pH 6.5 (phosphate), and pH 7.5 (phosphate) (left to right in each time series) at t = 0, 50 min, 150 min, and

5000 min. Bottom panel: Quantitative analysis of the relative darkness (%) over time for the reaction mixture at pH 5.0 (1 M acetate buffer), monitored by image analysis. Spikes in the graph are artifacts due to reflections from ambient sunlight.

As shown in Figure 2.1:

- At pH 4.0 (acetate buffer), no significant colour change was observed even after 5000 min, indicating negligible reaction.

- At pH 5.0 (acetate buffer), a gradual and mild darkening of the solution occurred over an extended period, as quantified by the increase in relative darkness (Figure 2.1, bottom panel). However, no rapid autocatalytic behaviour or phase separation was observed.

- At pH 6.5 (phosphate buffer), the reaction proceeded noticeably faster, with significant colour change and turbidity appearing by 50-150 min, leading to the formation of the second liquid phase.

- At pH 7.5 (phosphate buffer), the reaction was very rapid, with intense colour development and phase separation evident within 50 minutes.

These observations qualitatively demonstrate the strong pH-dependence of the reaction, with significantly accelerated product formation and phase separation at higher pH values. The reaction at pH 5.0 proceeds slowly and does not exhibit the clear autocatalytic signatures observed at neutral or mildly basic pH or in unbuffered solutions.

f) Comparative visual monitoring of HCN reactions with cysteamine and its analogs

To elucidate the relative contributions of the thiol and amine functionalities to the reaction with HCN, comparative visual experiments were performed. Equimolar mixtures of HCN (2 M) and one of the following compounds (2 M) – cysteamine hydrochloride,

ethanolamine hydrochloride, or sodium 2-mercaptoethanesulfonate (MESNA) – were prepared in water.

Specifically, to 4 mmol of each respective activator (cysteamine hydrochloride: 454 mg; ethanolamine hydrochloride: 390 mg; MESNA: 657 mg) was added an aqueous stock solution of HCN (4 M) and deionized water to achieve final concentrations of 2 M for both HCN and the activator in a total volume of 2 mL per vial. The vials were maintained at 25 °C and visually monitored, with photographic records taken at selected intervals (Figure 2.2).

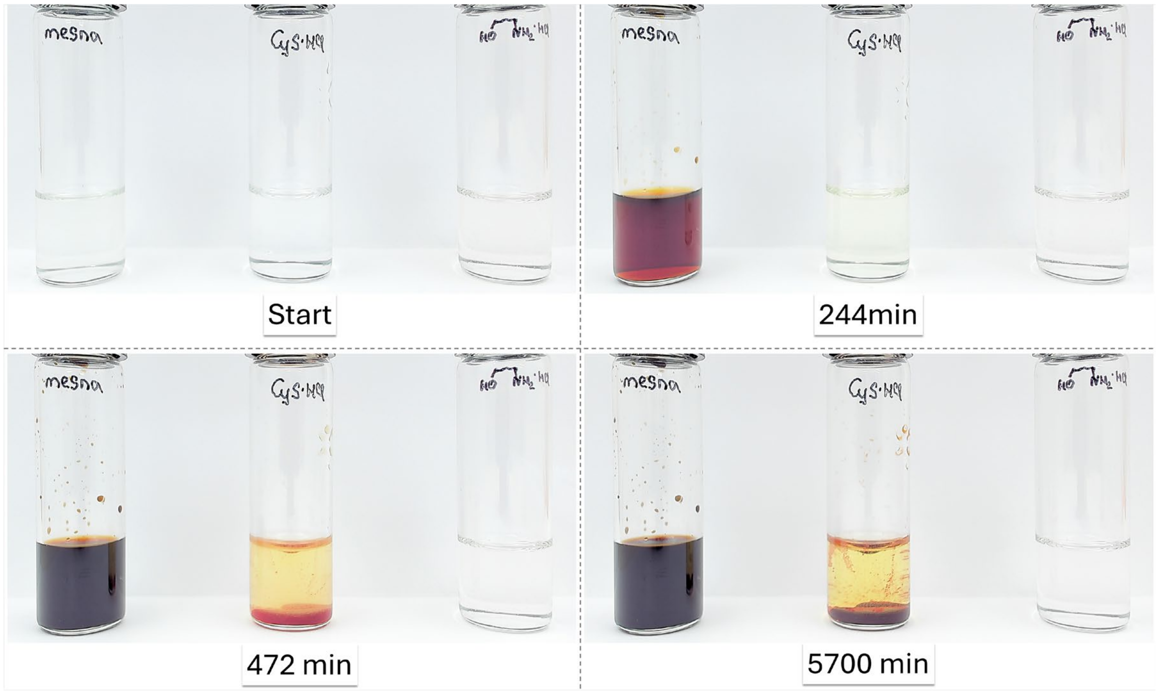

Figure 2.2. Comparative visual monitoring of reactions between HCN (2 M) and cysteamine hydrochloride or its analogs (2 M) at 25 °C. Vials (left to right in each panel) contain reaction mixtures with: MESNA, cysteamine hydrochloride (MEA·HCl; Cys·HCl on the photo), and ethanolamine hydrochloride. Time points shown are: Start (0 min), 244 min (~4 h), 472 min (~8 h), and 5700 min (~95 h).

The visual progression of these reactions is shown in Figure 2.2.

- Initially (Start), all solutions were clear and colorless.

- The reaction with **MESNA** (thiol group only) exhibited the first significant color change (to dark red/brown) after approximately **4 hours** (e.g., 244 min point in Figure 2.2).

- The reaction with **cysteamine hydrochloride** (thiol and amine groups) showed noticeable color development (yellow to orange) and turbidity indicative of reaction onset after approximately **8 hours** (e.g., 472 min point), eventually forming a deeply colored two-phase system.

- The reaction with **ethanolamine hydrochloride** (amine group only) displayed no significant visual changes (remaining clear and colorless) even after extended observation periods (e.g., 5700 min / ~95 hours, and confirmed up to 4 days in separate monitoring).

These comparative experiments visually demonstrate that the thiol group (present in MESNA and cysteamine) plays a critical role in initiating a rapid reaction with HCN under these conditions, while the amine group alone (in ethanolamine) is insufficient to promote a similar transformation.

g) Visual monitoring of the HCN-cysteamine reaction in the presence of malononitrile

To assess whether other nitriles could initiate and accelerate the reaction between HCN and cysteamine, a comparative visual experiment was performed. A reaction mixture was prepared by dissolving cysteamine hydrochloride (682 mg, 6 mmol) in freshly prepared 2 M aqueous HCN (3 ml, 6 mmol). The resulting homogeneous solution was immediately divided equally into two separate vials. Malononitrile (CH2(CN)2; 9.9 mg, 0.15 mmol, corresponding to 5 mol% relative to cysteamine) was added to one vial, while the second vial served as an additive-free control. Both vials were placed side-by-side at 25 °C and visually monitored (Figure 2.3).

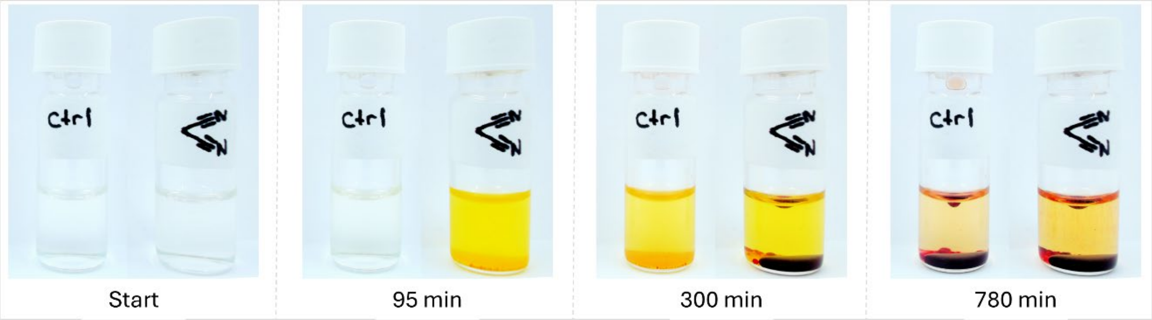

Figure 2.3. Visual monitoring of the HCN-cysteamine reaction initiated by malononitrile. Comparative visual progression of the reaction between HCN (2 M) and cysteamine hydrochloride (2 M) at 25 °C. **Left vial:** Control experiment without additives. **Right vial:** Experiment initiated with 5 mol% of malononitrile. The addition of malononitrile significantly shortens the reaction's lag phase, with noticeable color development and subsequent phase separation appearing at approximately 95 minutes, while the control sample remains unchanged over the same period.

3. NMR Experiments

The experiments were conducted using internal standards such as acetate or t-BuOH. The obtained spectra were integrated by regions using ACD/NMR Processor Academic edition, ACD Labs Release 12.00. Quantitative analysis of ^{1}H NMR spectra for kinetic experiments was performed by integrating characteristic signals of the reactants and internal standard (if used). The initial concentration of cysteamine hydrochloride was accurately known from its weighed mass. For experiments where other compounds (e.g., benzene, Triton X-100, isopentenyl alcohol, cyclohexanol, adenine) were monitored alongside cysteamine, their concentrations were determined by normalizing their respective signal integrals to the integral of a known number of cysteamine protons (the -CH_2S- triplets), assuming the initial cysteamine concentration as a primary reference. This approach was chosen because cysteamine hydrochloride was the main solid reactant weighed with high precision. Detailed tables of integral values for each kinetic experiment are compiled in **Appendix S1.**

a) Standard Addition Experiment (Fig. 1B), 2M HCN + 2M cysteamine*HCl, 40°C

For this experiment, liquid HCN was added to a pre-cooled D_2O, followed by dilution to achieve a 2M concentration of HCN. A measured amount of cysteamine hydrochloride was then added just before the start of the experiment to achieve a final concentration of 2M for both reactants. Simultaneously, a portion of this solution was kept at 40°C in a sealed vial for 8 hours to create a "seed" solution. 5% of the aged solution from the vial was then added to a freshly prepared 2M HCN and 2M cysteamine solution, while another solution was prepared for seed aging. After 8 hours, the experiment was repeated with a 10% seed solution.

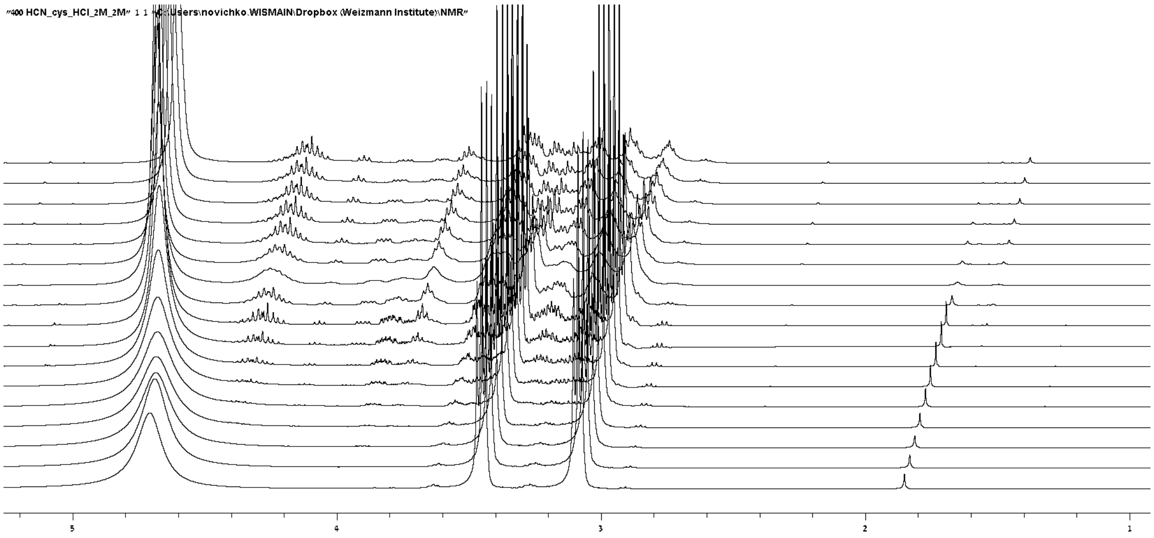

Figure 3.1. NMR spectra of the standard addition experiment (no seeds), 2M HCN + 2M cysteamine•HCl, at 40°C, with 10-minute intervals. The characteristic triplets of cysteamine between 3 and 3.5 ppm were used for integration and for constructing the kinetic concentration curve of cysteamine (Figure 1B).

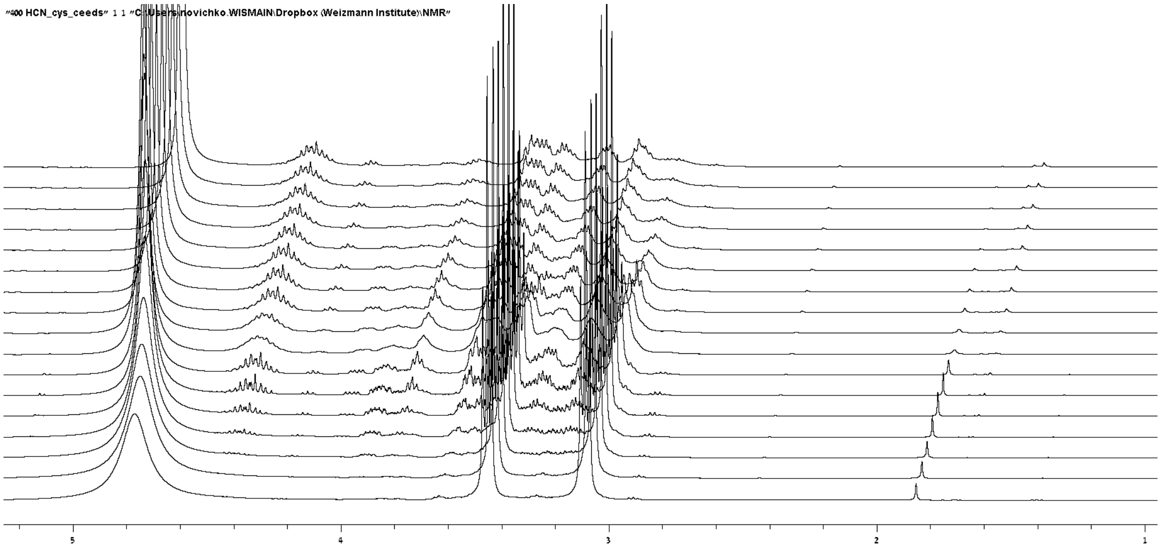

Figure 3.2. NMR spectra of the standard addition experiment (5% seeds), 2M HCN + 2M cysteamine•HCl, at 40°C, with 10-minute intervals. The characteristic triplets of cysteamine between 3 and 3.5 ppm were used for integration and for constructing the kinetic concentration curve of cysteamine (Figure 1B).

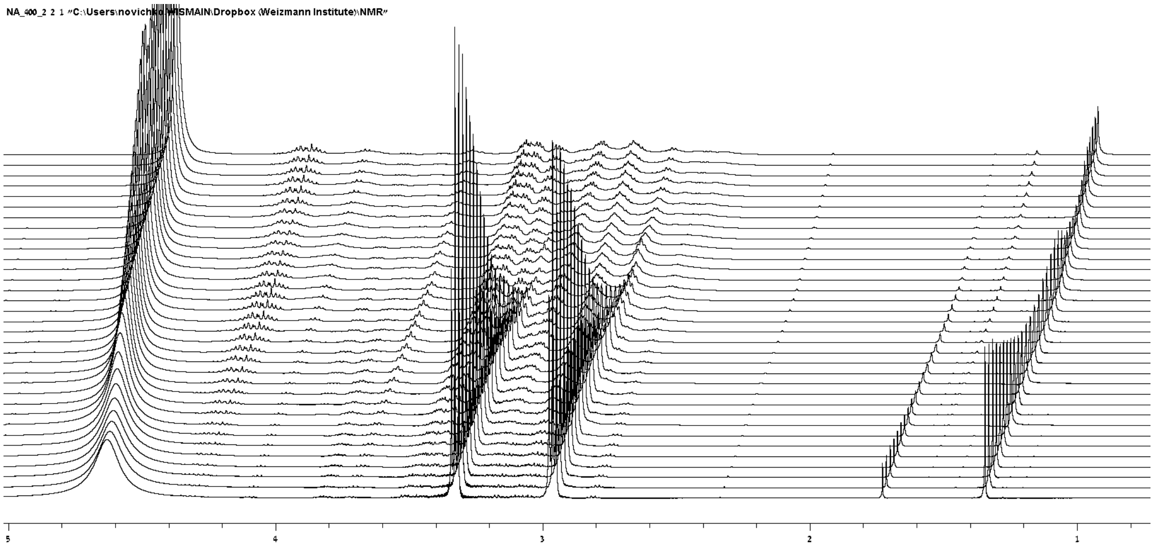

Figure 3.3. NMR spectra of the standard addition experiment (10% seeds), 2M HCN + 2M cysteamine•HCl, at 40°C, with 5-minute intervals. The characteristic triplets of cysteamine between 2.8 and 3.4 ppm were used for integration, and an internal standard of t-BuOH was included. These spectra were utilized to construct the kinetic concentration curve of cysteamine (Figure 1B).

b) NMR experiment with benzene (Fig. 4A, B), 2M HCN + 2M cysteamine*HCl, 40°C

For the NMR experiment involving benzene, deuterated water was mixed with a small amount of benzene to prepare a benzene-in-water solution. Liquid HCN was then added to the pre-cooled D_2O-benzene solution, followed by dilution to achieve a 2M concentration of HCN. A measured amount of cysteamine hydrochloride was subsequently added just before the start of the experiment to achieve a final concentration of 2M for both HCN and cysteamine. The initial concentration of benzene was calculated using the intensity of the cysteamine signal.

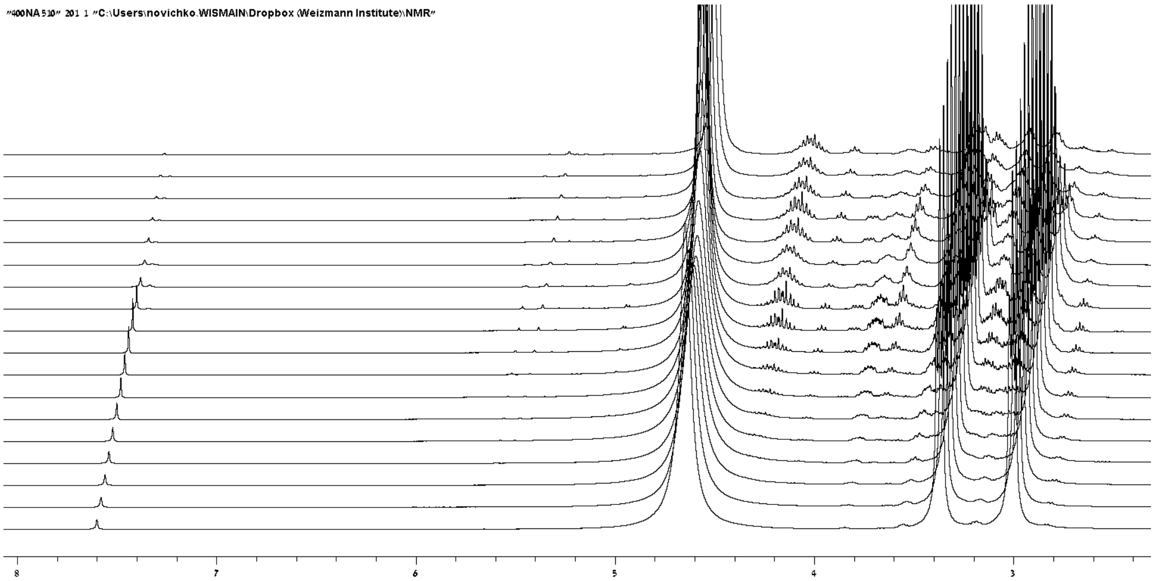

Figure 3.4. NMR spectra of 2M HCN + 2M cysteamine•HCl with benzene at 40°C, recorded at 10-minute intervals. The characteristic triplets of cysteamine between 2.9 and 3.4 ppm, along with the benzene signal at 7.6 ppm, were used for integration and to construct the kinetic concentration curves of cysteamine and benzene in solution (Figure 4A).

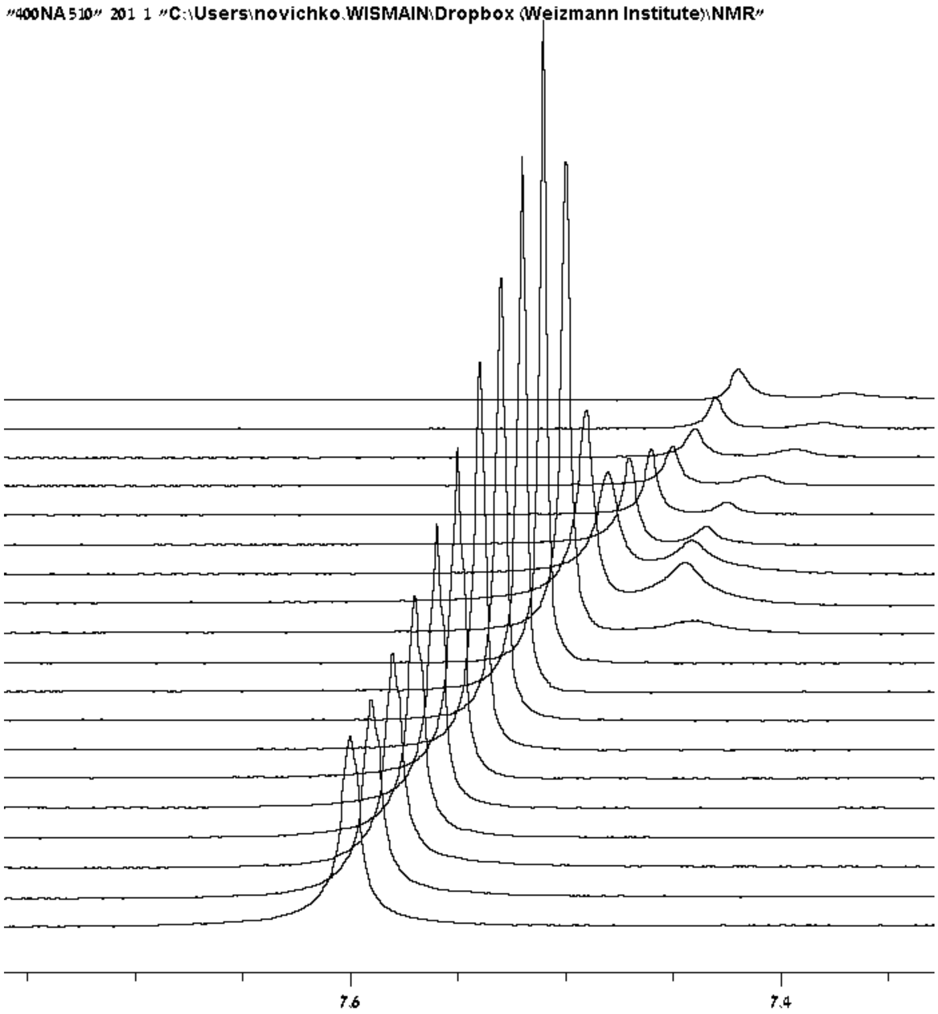

Figure 3.5. ^{1}H NMR spectra showing a zoomed-in view of the benzene region at 7.6 ppm, highlighting the emergence of a broadened twin signal (benz s2), which coincides with the initiation of phase separation in the system.

c) NMR experiment with triton X-100 (Fig. 2 C), 2M HCN + 2M cysteamine*HCl, 40°C

The standard preparation involved pre-cooling D_2O, adding liquid hydrogen cyanide, and diluting to achieve a 2M concentration of HCN. A measured amount of cysteamine hydrochloride was then added just before the start of the experiment to reach a final concentration of 2M for both HCN and cysteamine. Immediately before the experiment, 10 µL of Triton X-100 (2-[4-(2,4,4-trimethylpentan-2-yl)phenoxy]ethanol) was added to 1 mL of the prepared mixture in an NMR tube.

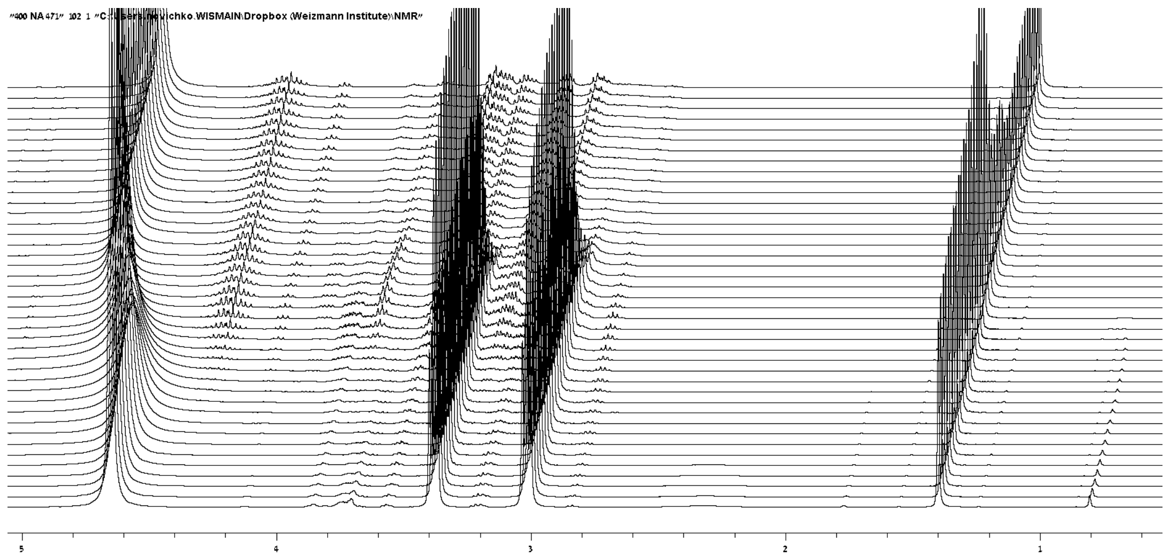

Figure 3.6. NMR spectra of the experiment with Triton X-100, 2M HCN + 2M cysteamine•HCl, at 40°C, recorded at 10-minute intervals. The characteristic triplets of cysteamine between 3.0 and 3.4 ppm and the signal of Triton X-100 at 0.8 ppm were used for constructing their respective kinetic concentration curves. The tert-Butanol internal standard at 1.4 ppm was used for reference.

d) NMR experiment with isopentenyl alcohol (Fig. 4 C), 2M HCN + 2M cysteamine*HCl, 40°C

The standard preparation involved pre-cooling D_2O, adding liquid hydrogen cyanide, and diluting to achieve a 2M concentration of HCN. A measured amount of cysteamine hydrochloride was then added just before the start of the experiment to reach a final concentration of 2M for both HCN and cysteamine. Immediately before the experiment, 10 µL of isopentenyl alcohol (3-methyl-2-buten-1-ol) was added to 1 mL of the prepared mixture in the NMR tube.

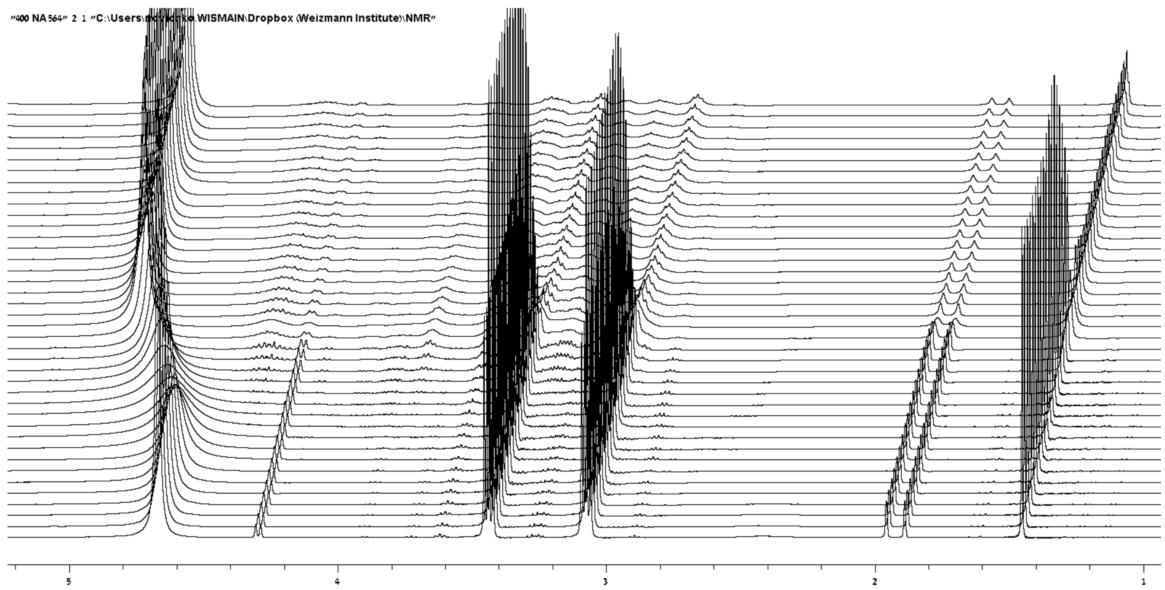

Figure 3.7. NMR spectra of the experiment with isopentenyl alcohol (3-Methyl-2-buten-1-ol) (Figure 4C), 2M HCN + 2M MEA*HCl, at 40°C, recorded at 10-minute intervals. The characteristic triplets of cysteamine between 3.0 and 3.5 ppm and the two singlets of isopentenyl alcohol around 1.9 ppm were used for constructing their respective kinetic concentration curves. The tert-butanol internal standard at 1.45 ppm was used for reference.

e) NMR experiment with cyclohexanol (figure 4 D), 2M HCN + 2M cysteamine*HCl, 40°C

The preparation began with pre-cooling D_2O, followed by the addition of liquid hydrogen cyanide and subsequent dilution to achieve a 2M concentration of HCN. Cysteamine hydrochloride was added immediately before the experiment to reach a final concentration of 2M for both HCN and cysteamine. Just before starting the experiment, 20 μL of cyclohexanol was added to 1 mL of the prepared mixture in NMR tube.

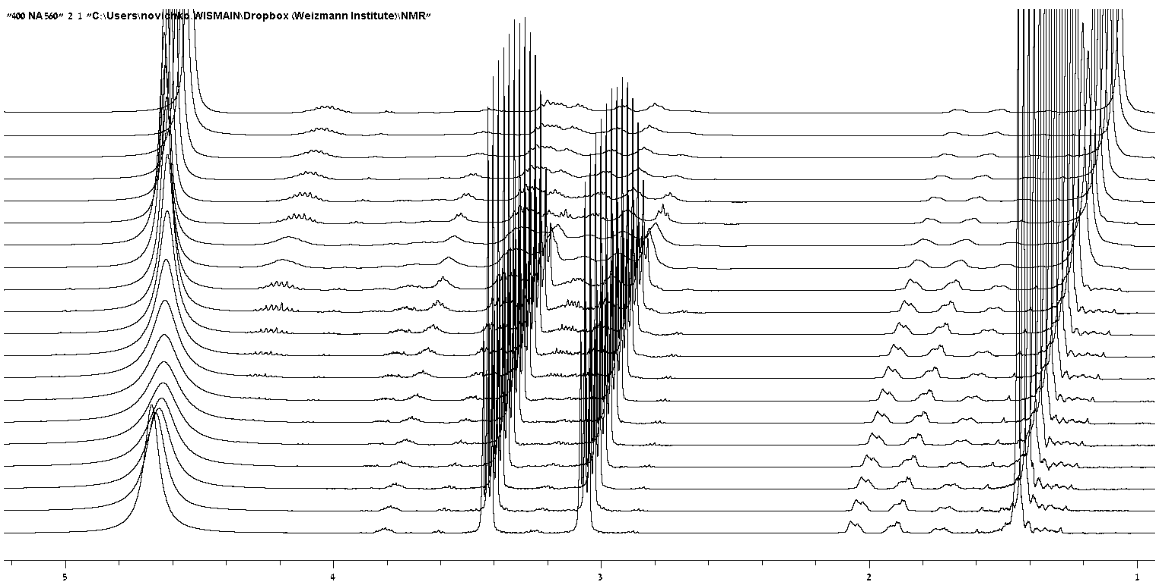

Figure 3.8. NMR spectra of the experiment with cyclohexanol (Figure 4D), 2M HCN + 2M MEA*HCl, at 40°C, recorded at 10-minute intervals. The characteristic triplets of cysteamine between 3.0 and 3.5 ppm and the multiplets of cyclohexanol between 1.8 and 2.1 ppm were used for constructing their respective kinetic concentration curves. The tert-butanol internal standard at 1.45 ppm was used for reference.

f) NMR experiment with adenine, 2M HCN + 2M cysteamine*HCl, 40°C

The preparation involved pre-cooling D_2O, adding liquid hydrogen cyanide, and diluting to achieve a 2M concentration of HCN. Cysteamine hydrochloride was added just before the experiment to reach a final concentration of 2M for both HCN and cysteamine. Immediately before starting the experiment, 13.5 mg of adenine was added to 1 mL of the prepared mixture in NMR tube.

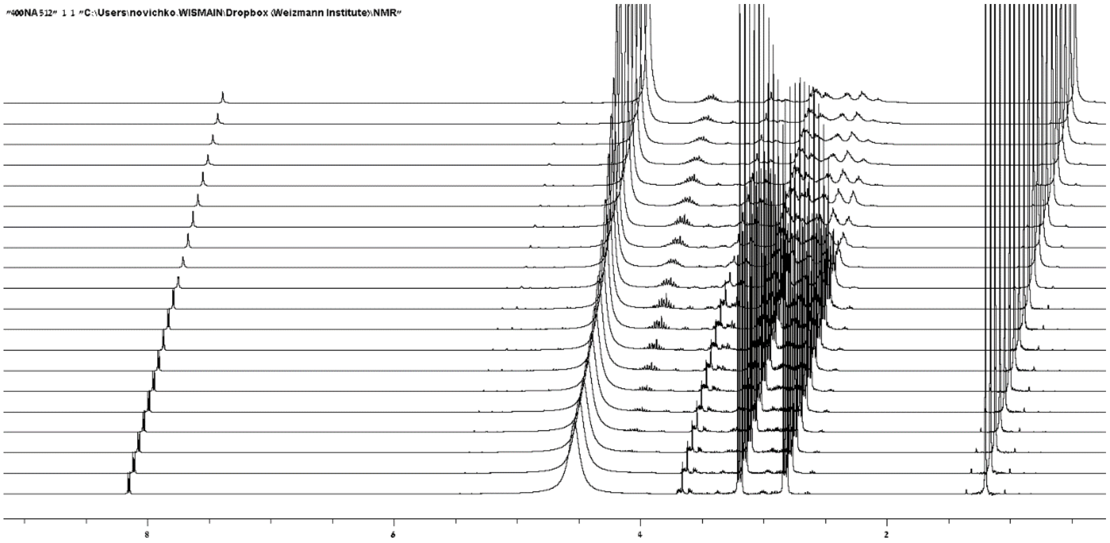

Figure 3.9. NMR spectra of the experiment with adenine, 2M HCN + 2M cysteamine•HCl, at 40°C, recorded at 10-minute intervals. The characteristic triplets of cysteamine between 2.9 and 3.3 ppm and the signal of adenine at 8.2 ppm were used for constructing their respective kinetic concentration curves. The tert-butanol internal standard at 1.3 ppm was used for reference.

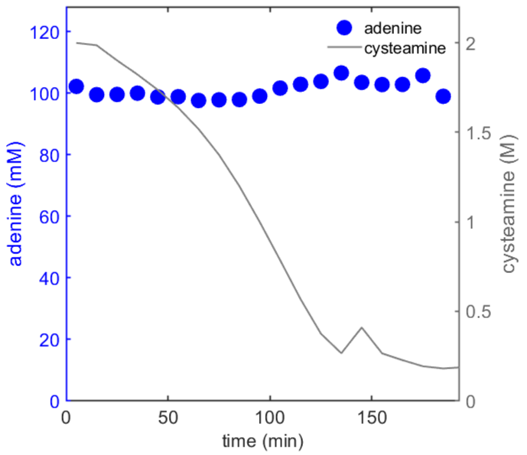

Figure 3.10. Kinetic concentration curves of adenine and cysteamine derived from ^{1}H NMR spectra. The concentration of adenine (blue circles) remains stable over time, indicating that it is not incorporated into the forming second phase.

g) NMR studies of m174

Proposed structure of m174 in solution:

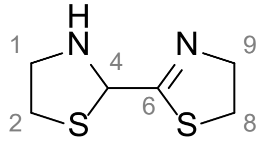

2-(thiazolidin-2-yl)-4,5-dihydrothiazole

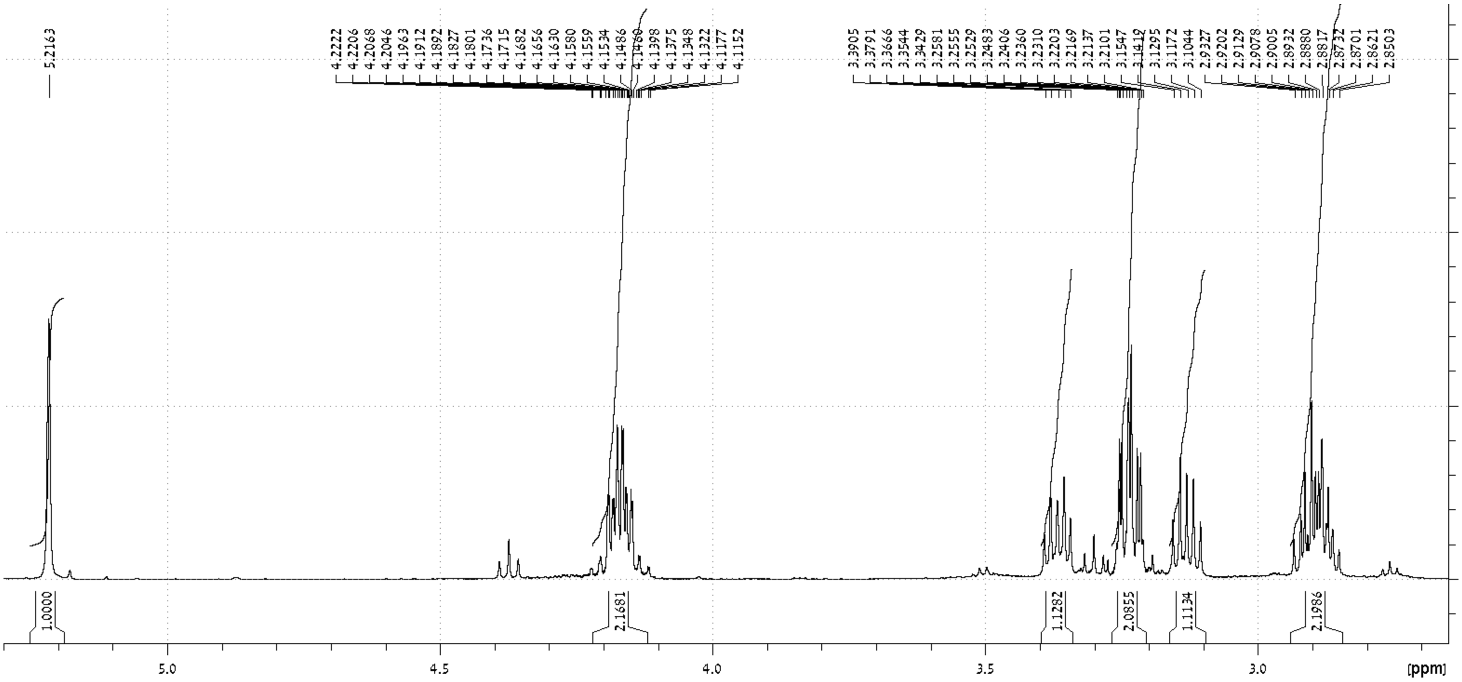

Figure 3.11. ^1H NMR spectrum of m174 in $CDCl_3$

^1H NMR (500 MHz, $CDCl_3$, 25 °C): δ 5.22 (s, 1 H, C(4)H), 4.22–4.12 (m, 2 H, C(9)H_2), 3.39–3.34 (m, 1 H, C(1)H), 3.25–3.22 (m, 2 H, C(8)H_2), 3.15–3.10 (m, 1 H, C(1)H), 2.94–2.89 (m, 2 H, C(2)H_2); integrals 1:2:1:2:1:2.

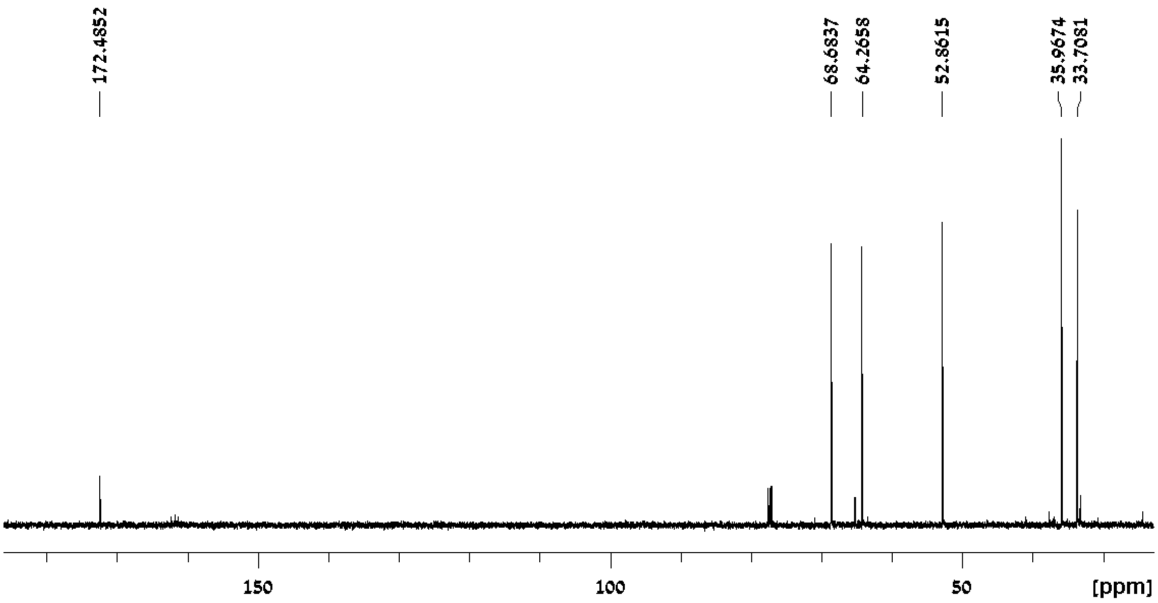

Figure 3.12. ^{13}C NMR spectrum of m174 in $CDCl_3$

$^{13}C\{^1H\}$ NMR (126 MHz, $CDCl_3$, 25 °C): δ 172.5 (C(6)), 68.7 (C(4)H), 64.3 (C(9)H_2), 52.9 (C(1)H_2), 35.9 (C(2)H_2), 33.7 (C(8)H_2).

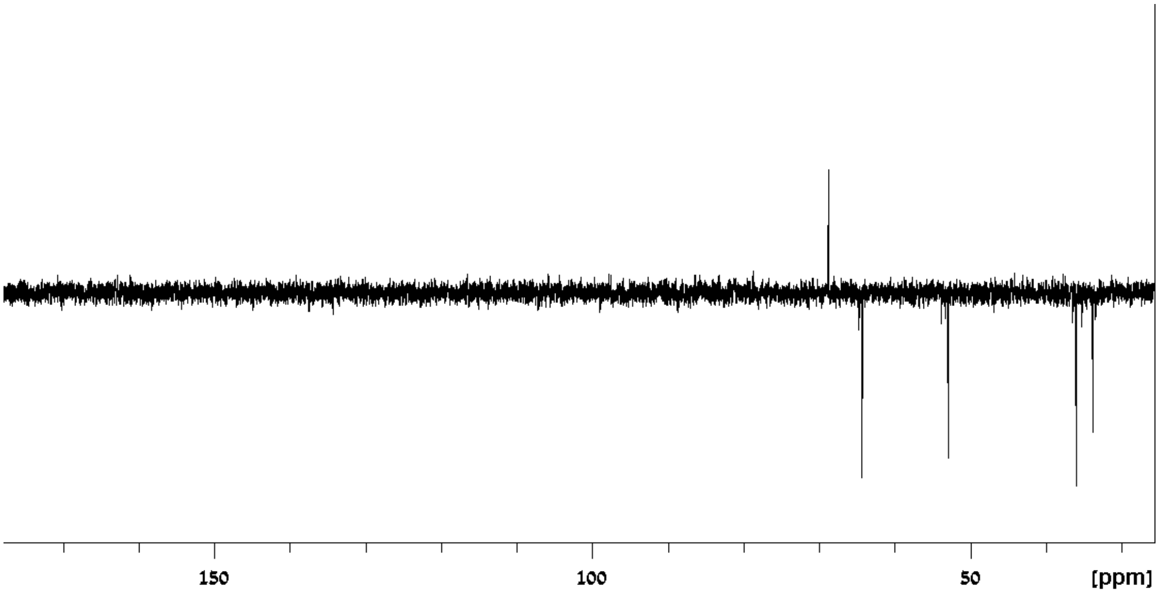

Figure 3.13. ^{13}C DEPT NMR spectrum of m174 in $CDCl_3$

DEPT-135 confirms the presence of five CH_2 signals (64.3, 52.9, 35.9, 33.7 ppm), and one CH signal at 68.7 ppm; the quaternary imine carbon at 172.5 ppm is absent, as expected.

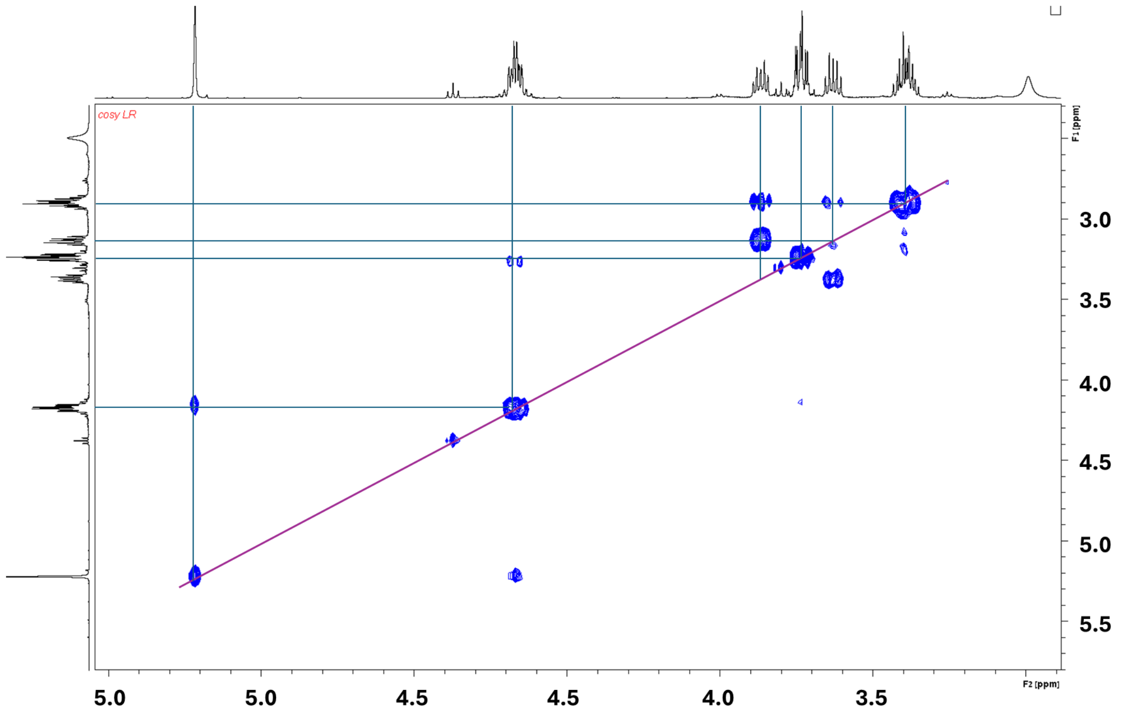

Figure 3.14. COSY NMR spectrum of m174 in $CDCl_3$

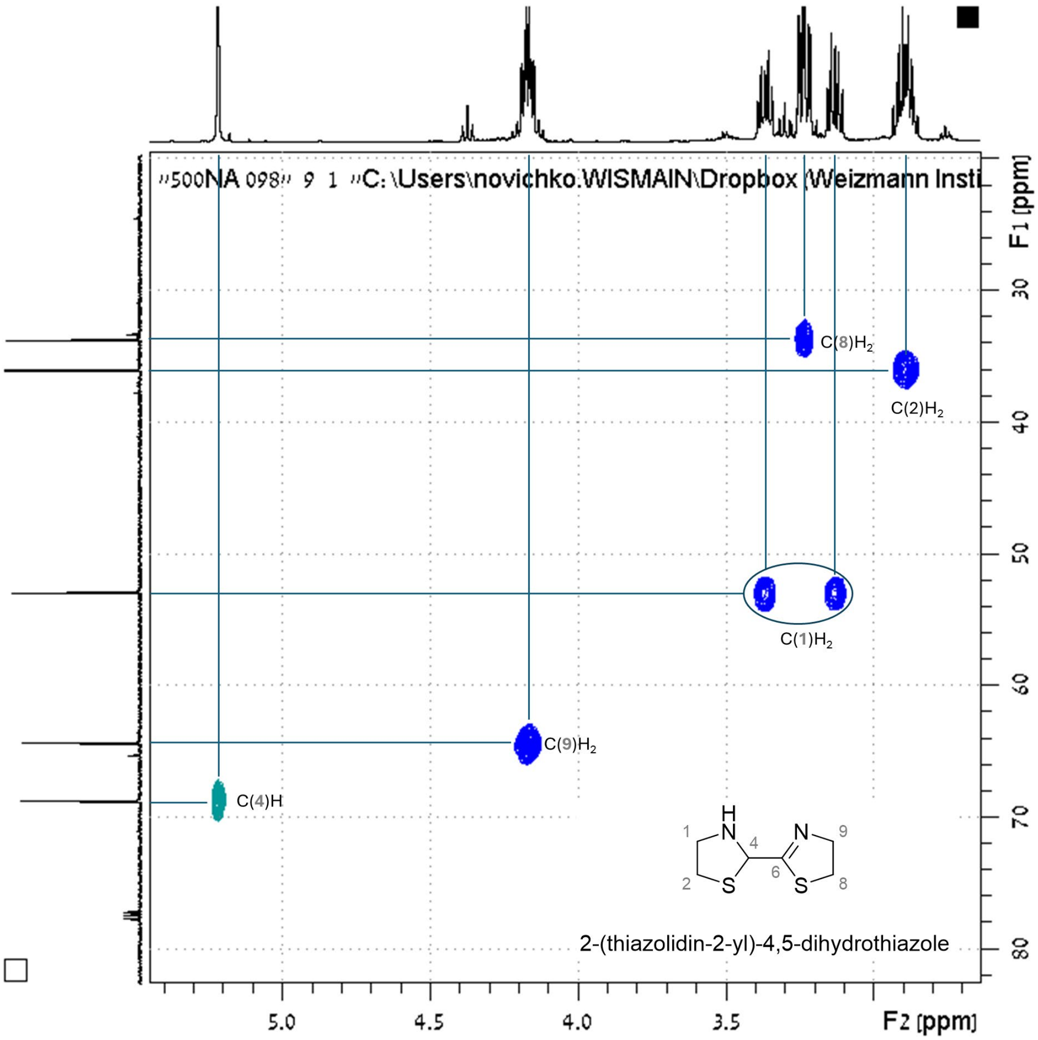

Figure 3.15. HSQC NMR spectrum of m174 in $CDCl_3$

h) NMR experiment $K^{13}CN$ with cysteamine under ^{13}C NMR control

Experimental Procedure

To gain molecular-level insight into the initial reaction steps, $K^{13}CN$ (1 mmol, 66 mg), KH_2PO_4 (1 mmol, 136 mg), and cysteamine hydrochloride (MEA*HCl, 1 mmol, 113 mg) were dissolved in D_2O, adjusting the total reaction volume to 1 ml. The reaction was conducted at 25 °C and monitored by ^{13}C NMR spectroscopy on a Bruker Avance spectrometer (125 MHz for ^{13}C). A series of ten consecutive ^{13}C {^{1}H} DEPTQ spectra (8 scans each, approx. 6 min acquisition per spectrum) were collected over the initial hour.

Subsequently, a more detailed ^{13}C {^1H} spectrum (20 scans) was recorded to enhance signal-to-noise and allow detailed spectral interpretation. Another spectrum (80 scans) was obtained after 6 hours. On the following day, the newly formed second liquid phase was carefully separated via pipette, dissolved in 150 µL TFA, diluted with 350 µL CD_2Cl_2, and analyzed by ^{13}C {^1H} NMR spectroscopy.

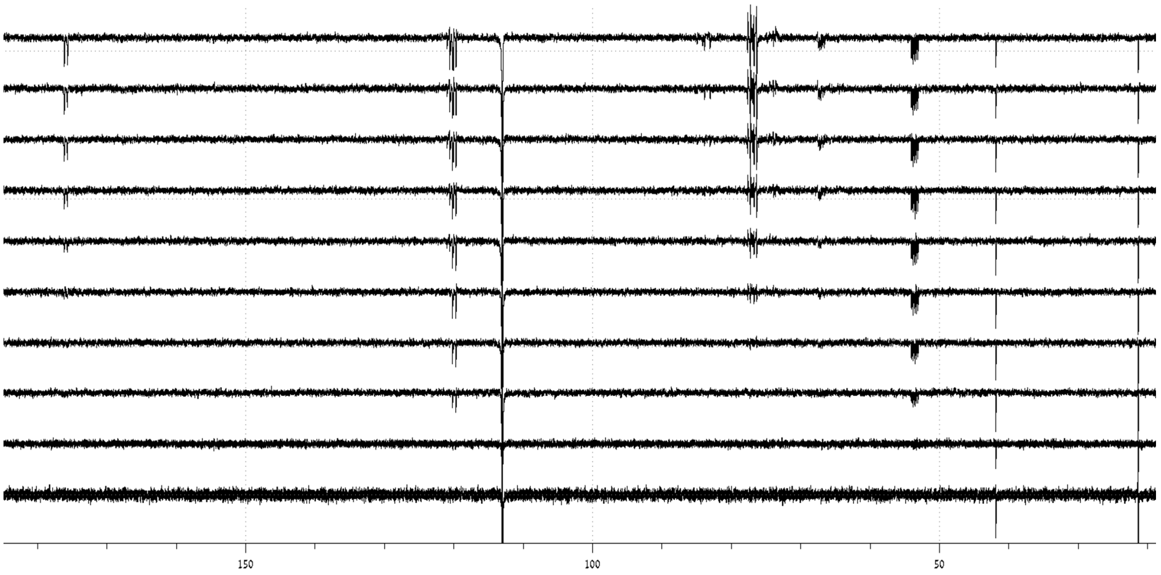

Figure 3.16. Stacked ^{13}C {^1H} DEPTQ spectra (500 MHz, D_2O, 25 °C) of the reaction mixture of $K^{13}CN$, KH_2PO_4, and MEA·HCl recorded during the first hour of the reaction. Signals of 2-cyanothiazolidine are at 119.9 ppm and 53.5 ppm.

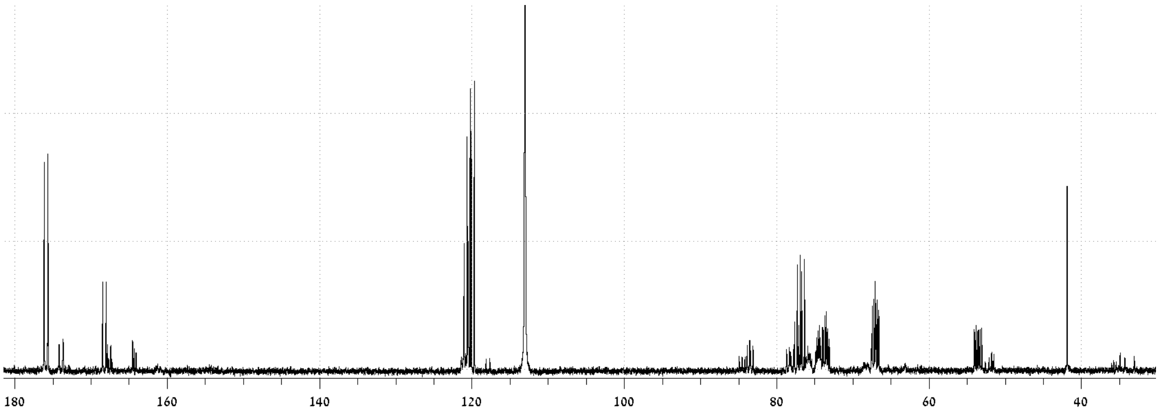

Figure 3.17. ^{13}C {^1H} NMR spectrum (500 MHz, D_2O, 25 °C) of the reaction mixture 1 hour after the start of the reaction.

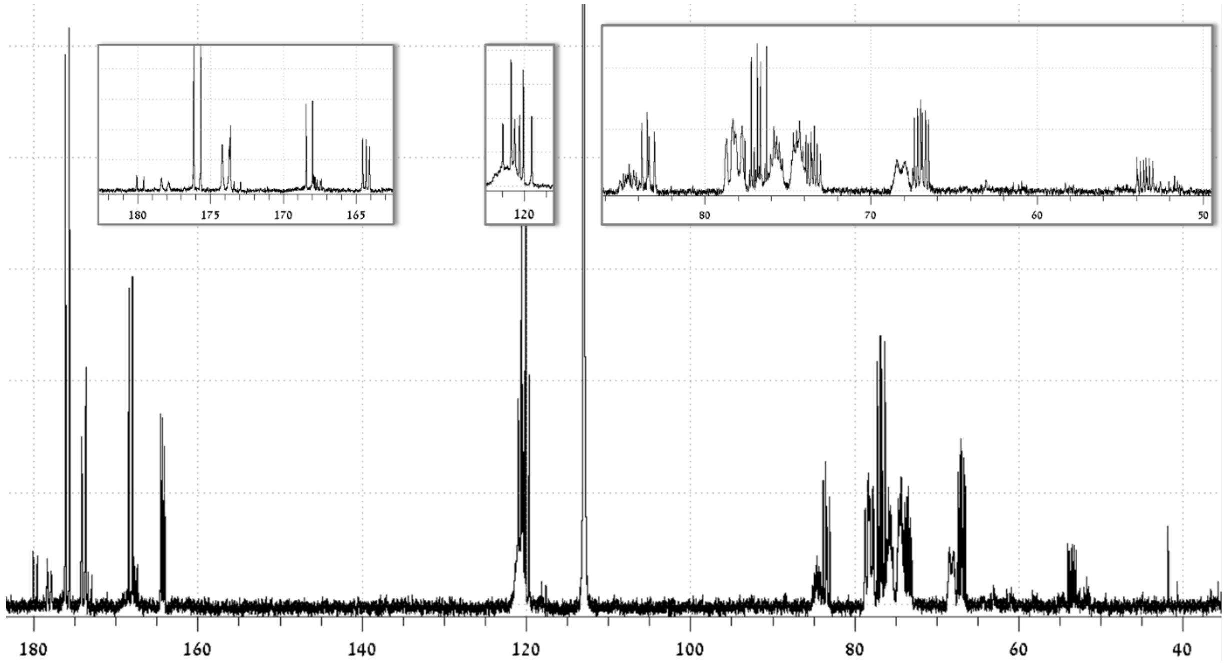

Figure 3.18. ^{13}C {^{1}H} NMR spectrum (500 MHz, D_2O, 25 °C) of the reaction mixture 6 hours after the start of the reaction. Insets show expanded regions of the spectrum.

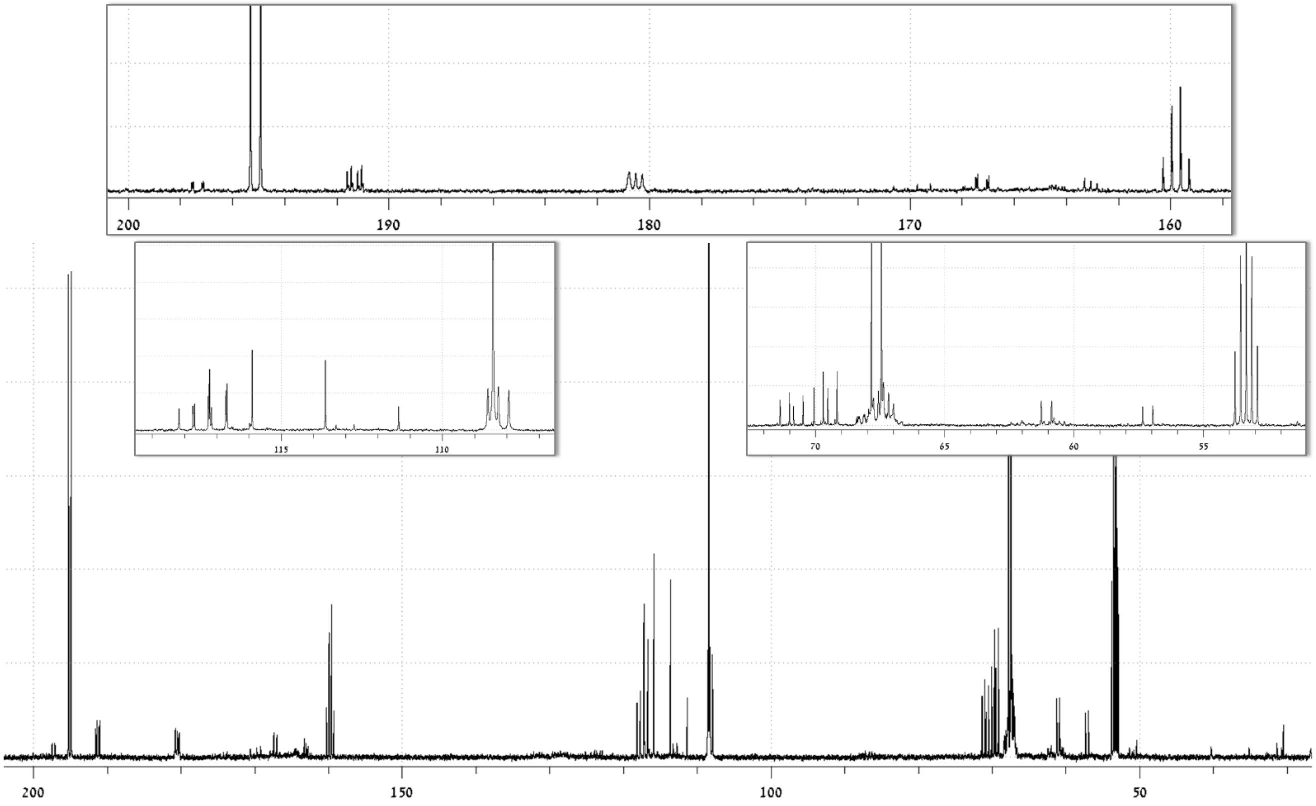

Figure 3.19. ^{13}C {^{1}H}NMR spectrum (500 MHz, CD_2Cl_2, 25 °C) of the second liquid phase separated from the reaction mixture on the following day. Insets show expanded regions of the spectrum.

i) ^{1}H NMR kinetic study of HCN- cysteamine reaction initiated by KOH

To investigate the effect of direct basification on the reaction initiation, a kinetic ^{1}H NMR experiment was performed. Cysteamine hydrochloride (227 mg, 2.00 mmol) and KOH (5.6 mg, 0.1 mmol, to achieve a final concentration of 0.1 M or 5 mol% relative to cysteamine) were dissolved together in D_2O to make a 0.5 mL solution. Subsequently, 6 µL of *tert*-butanol (internal standard) was added. The reaction was initiated by adding 0.5 mL of a freshly prepared 4 M HCN stock solution in D_2O to the 0.5 mL cysteamine/KOH solution in an NMR tube. This resulted in final concentrations of 2 M HCN and 2 M cysteamine hydrochloride in the 1 mL total reaction volume. The reaction was monitored by ^{1}H NMR spectroscopy at 40 °C, with spectra recorded at 5-minute intervals. The reaction started instantly, confirming that basification alone is sufficient to initiate the process.

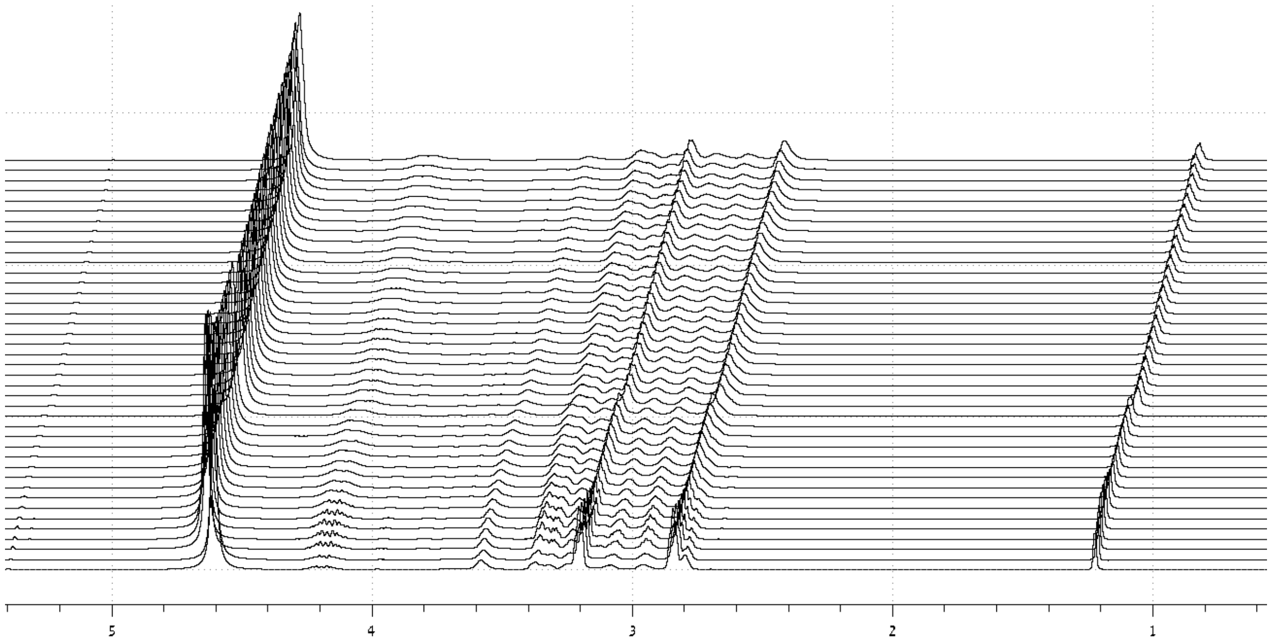

Figure 3.20: Stacked ^{1}H NMR spectra (400 MHz, D_2O, 40 °C) for the reaction of HCN (2 M) with cysteamine hydrochloride (2 M) initiated by KOH (0.1 M final concentration). Spectra were recorded at 5-minute intervals. The decay of cysteamine signals (e.g., triplets around δ 3.0-3.5 ppm) and the signals of *tert*-butanol (internal standard, ~δ 1.2-1.3 ppm) are visible.

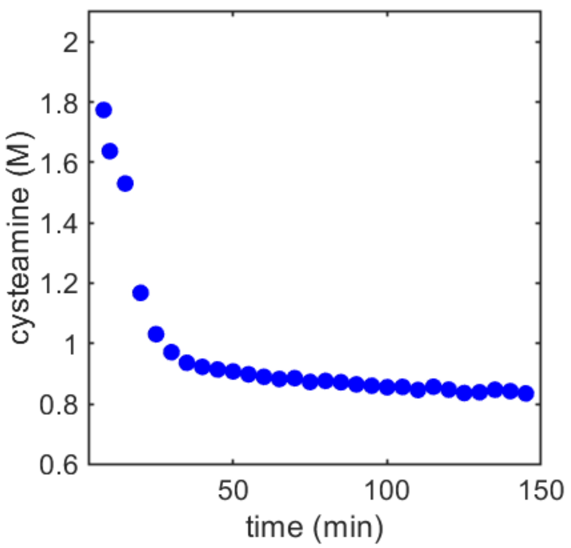

Figure 3.21: Kinetic curve for the consumption of cysteamine hydrochloride (2 M) in the reaction with HCN (2 M) at 40 °C in D_2O, initiated by KOH (0.1 M). Concentrations were determined by ^{1}H NMR spectroscopy using *tert*-butanol as an internal standard. The reaction commenced rapidly upon mixing.

j) ^{1}H NMR kinetic study of HCN-cysteamine reaction in phosphate buffer (pH 6.5)

To study the reaction kinetics under buffered conditions mimicking a pH value observed during the exponential phase of the unbuffered reaction, a ^{1}H NMR experiment was conducted in phosphate buffer. Cysteamine hydrochloride (227 mg, 2.00 mmol) was dissolved in 0.5 mL of a freshly prepared 2 M potassium phosphate buffer in D_2O (pH 6.5). It was noted that the solids did not fully dissolve at this stage. To this suspension/solution, 6 µL of *tert*-butanol (internal standard) was added. The reaction was initiated by adding 0.5 mL of a freshly prepared 4 M HCN stock solution in D_2O. Upon addition of the HCN solution, the remaining solids dissolved, resulting in a clear solution. This led to final concentrations of 2 M HCN and 2 M cysteamine hydrochloride in 1 M phosphate buffer (pH 6.5) in a total reaction volume of 1 mL. The reaction was monitored by ^{1}H NMR

spectroscopy at 40 °C, with spectra recorded at 5-minute intervals. The reaction started almost immediately, although a small lag phase could be discerned.

A 2 M potassium phosphate buffer solution (pH 6.5) in D_2O was prepared by dissolving 0.227 g of KH_2PO_4 and 0.058 g of K_2HPO_4 in D_2O and adjusting the total volume to 1 mL.

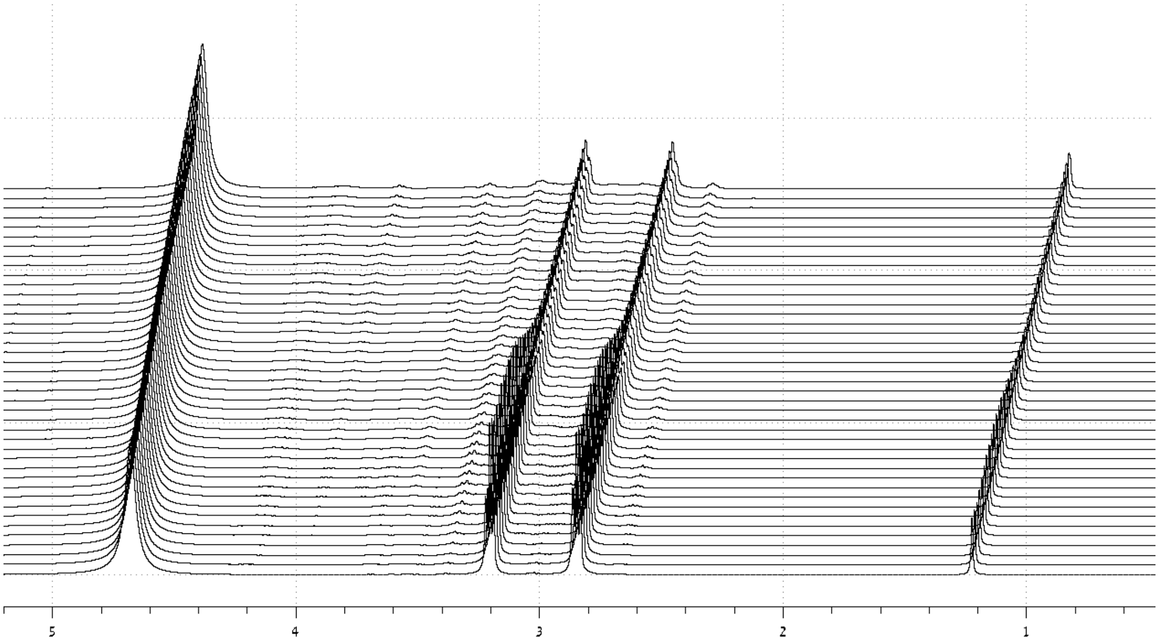

Figure 3.22: Stacked ^{1}H NMR spectra (500 MHz, D_2O, 40 °C) for the reaction of HCN (2 M) with cysteamine hydrochloride (2 M) in 1 M potassium phosphate buffer (pH 6.5). Spectra were recorded at 5-minute intervals. The decay of cysteamine signals and the signals of *tert*-butanol (internal standard) are visible.

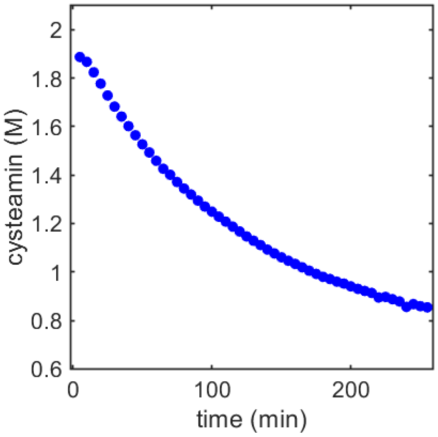

Figure 3.23: Kinetic curve for the consumption of cysteamine hydrochloride (2 M) in the reaction with HCN (2 M) at 40 °C in D_2O, buffered with 1 M potassium phosphate (pH 6.5). Concentrations

were determined by ^{1}H NMR spectroscopy using *tert*-butanol as an internal standard. A short lag phase was observed before rapid consumption.

k) Seeding experiments with m261

Cysteamine hydrochloride (454 mg, 4.00 mmol) was dissolved in 2 mL of HCN solution in D_2O (~1M, significant amount of HCN was lost from original 2M solution due to evaporation; given concentration is estimated based on the cysteamine consumption in the reaction). The solution was divided into two portions, 630 mL each. The first portion was transferred to NMR tube and used as control. The second portion was mixed with grained crystals of m261 (2.2 mg), and the suspension was transferred to another NMR tube. The reaction was monitored by ^{1}H NMR spectroscopy at 30 °C, with spectra recorded at 10-minute intervals. After 15 hours, additional ^{1}H and ^{13}C NMR spectra of the final mixture we recorded (Figures 3.27-28). At this stage, the sample seeded with m261 contained pale-yellow solution with white crystals on the bottom of the tube, while control sample contained pale-yellow solution and dark-yellow liquid on the bottom and walls of the tube. Next, the aqueous solutions from both samples were removed by decantation, the residues were dissolved in DMSO-d^6, and ^{1}H and ^{13}C NMR spectra were recorded for both samples (Figures 3.25-26). Identification of m261 in these spectra was done using ^{1}H and ^{13}C NMR spectra (Figure 3.24) recorded from the same batch of crystals that were used for seeding and analyzed by single crystal X-ray.

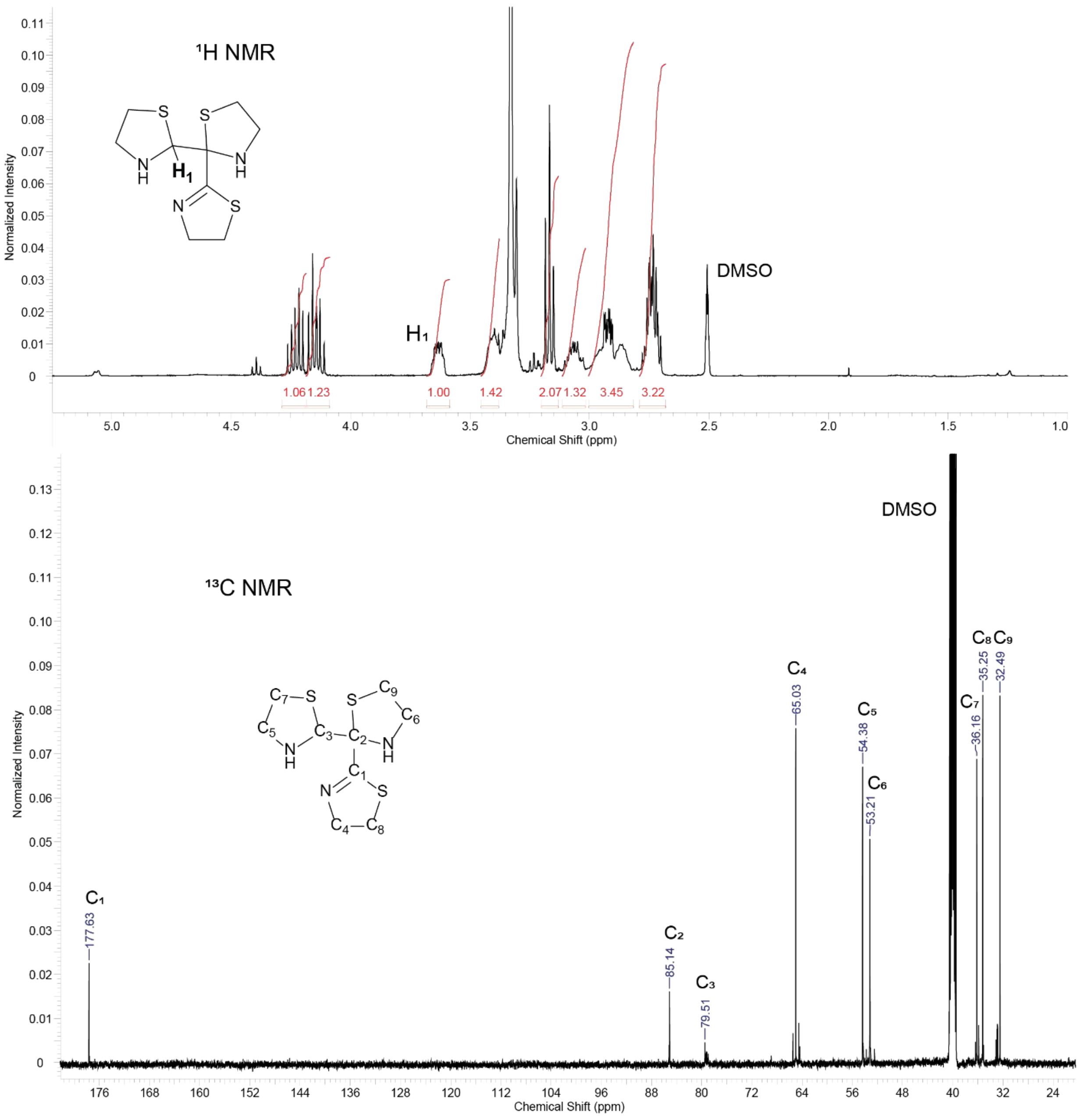

Figure 3.24. ^{1}H and ^{13}C NMR of m261 crystals that were used for seeding and analyzed by single crystal X-ray. The assignment of the picks in ^{13}C spectrum is based on the chemical shift predicted by *ChemDraw* software.

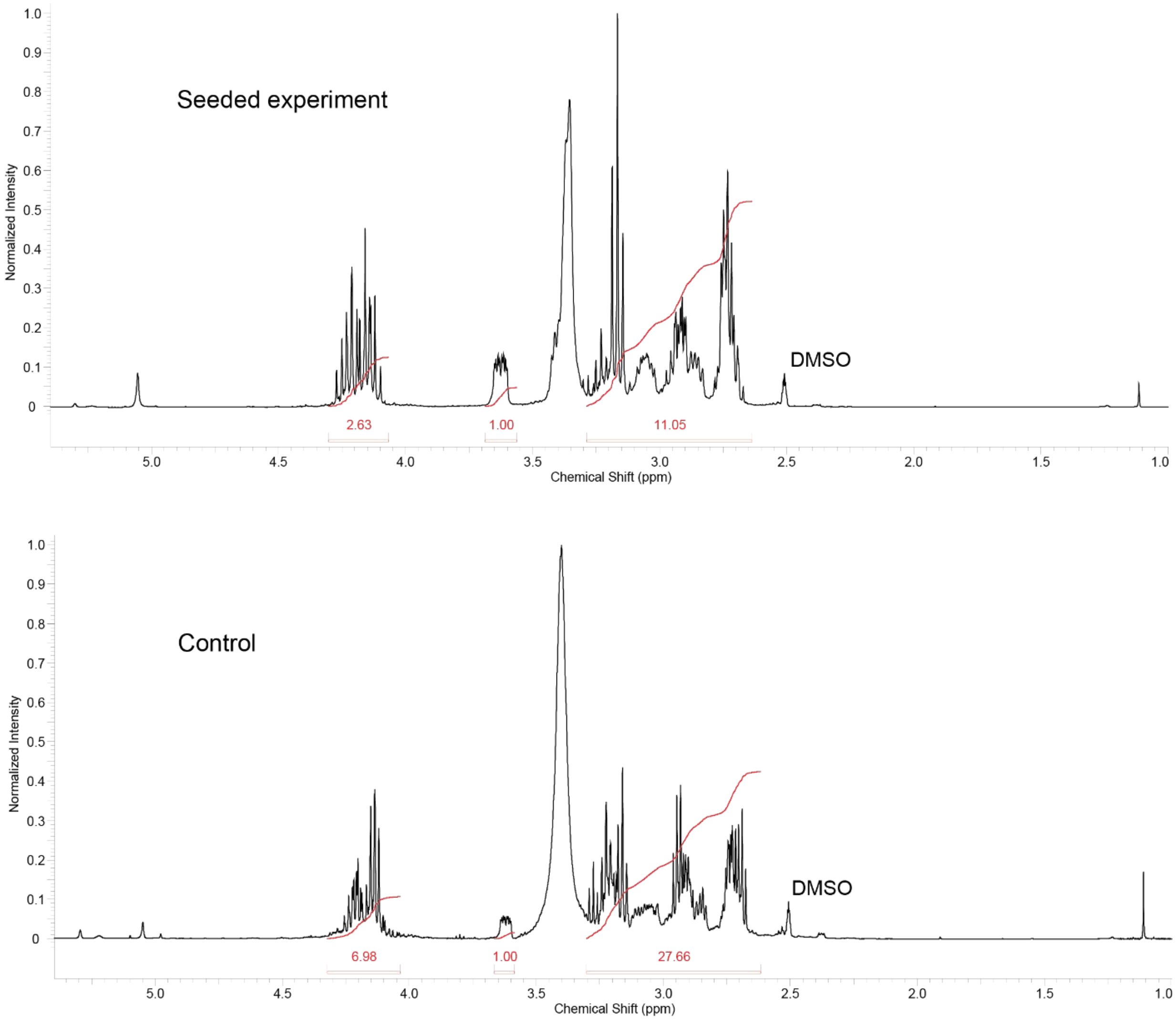

Figure 3.25. Comparison of ^{1}H NMR spectra in DMSO-d^{6} of the precipitates (liquid and solid) from the experiments seeded with m261 and identical control experiment. Taking integral of C*H* in m261 as 1, the sum for all integrals of CH_2 in m261 should be 11 (considering on signal hiding under water signal based on Figure 3.24). Based on the integrals shown here, the content of m261 in the seeded sample is about 80%, while the content of m261 in control sample is about 30 %.

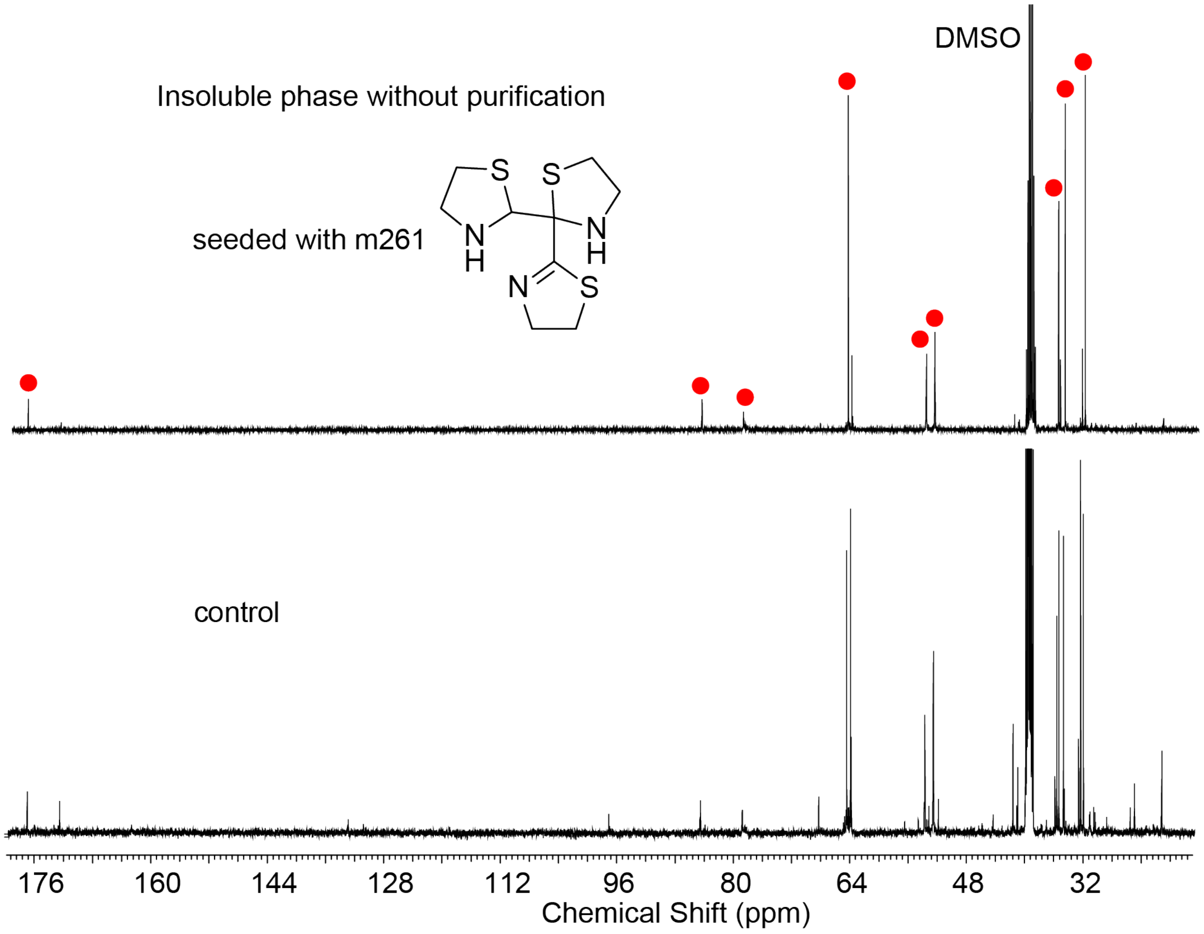

Figure 3.26. Comparison of ^{13}C NMR spectra in DMSO-d^6 of the precipitates (liquid and solid) from the experiments seeded with m261 and identical control experiment. Red cycles indicate signals belonging to m261.

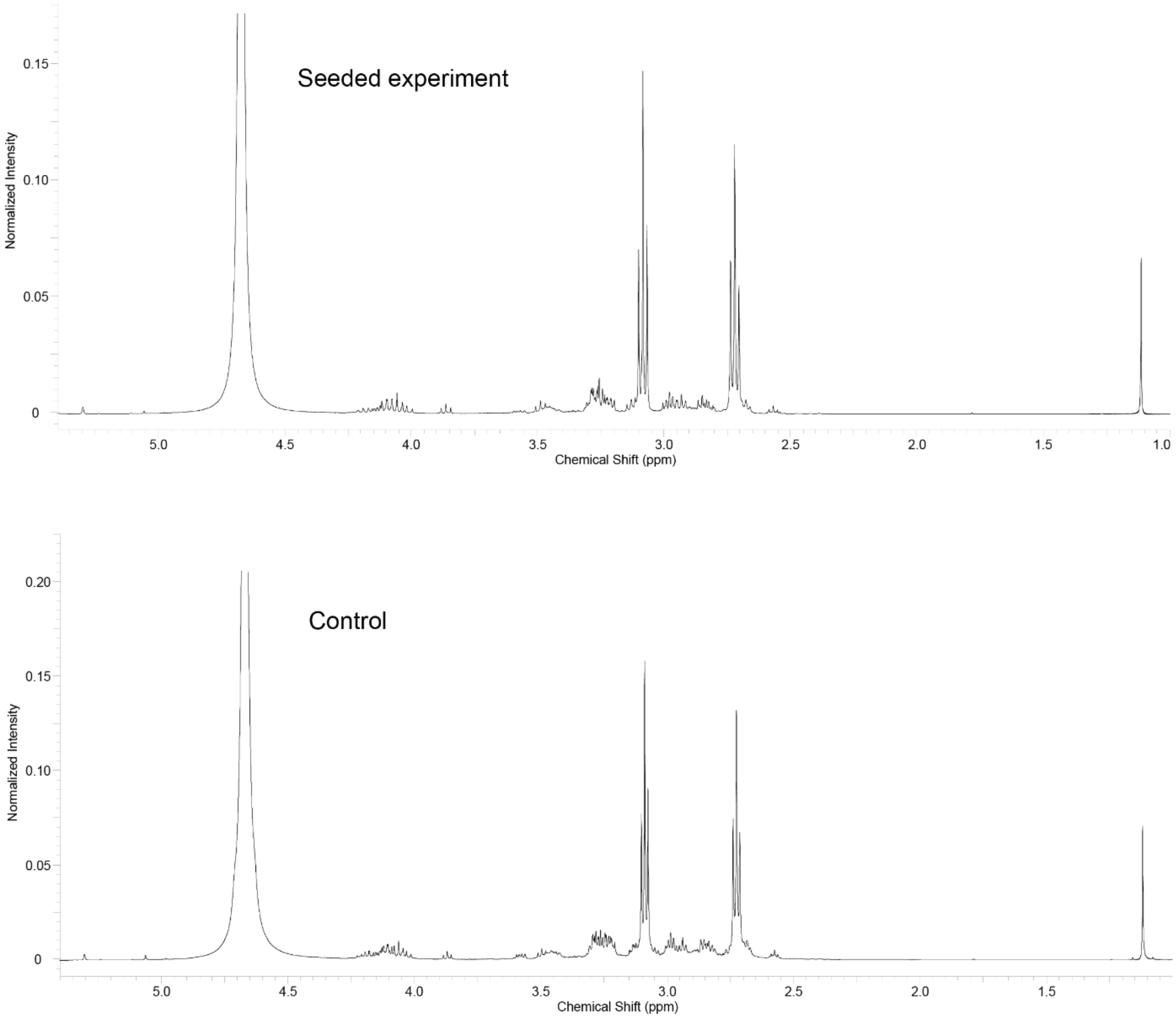

Figure 3.27. Comparison of ^{1}H NMR spectra of the aqueous phases from the experiments seeded with m261 and identical control experiment.

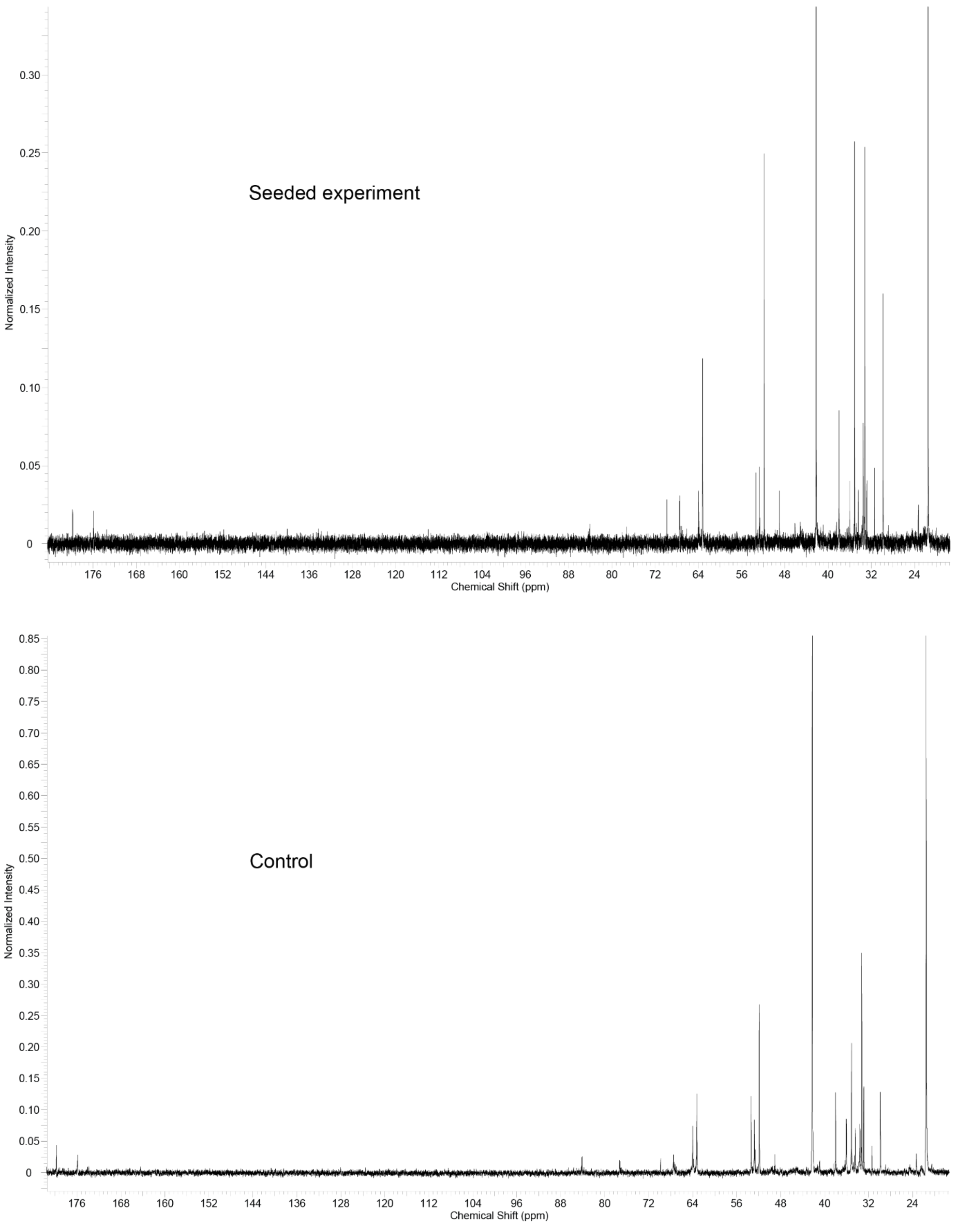

Figure 3.27. Comparison of ^{13}C NMR spectra of the aqueous phases from the experiments seeded with m261 and identical control experiment.

4. Isolation and structural analysis of products by X-ray crystallography

a) Single-crystal X-ray diffraction analysis of compounds.

X-ray data collection and structure refinement. Single crystals of m261benzoylated, m174benzoylated, m192benzoylated, m261, m192 and m102 suitable for X-ray diffraction were coated in Paratone oil (Hampton Research, CA USA), mounted on a MiTeGen loop and flash frozen in liquid nitrogen. m261benzoylated and m174benzoylated were recorded on a Rigaku Synergy S system equipped with a Dectris Pilatus 300K CdTe detector. Data were measured using Mo-Kα radiation (0.71073 Å) and processed with CrysAlis^{PRO}. (1.171.40.80a Rigaku OD, 2020 or 1.171.41.110a (Rigaku OD, 2021). Diffraction data for m192benzoylated were recorded on a Rigaku Synergy R system equipped with a HyPix-Arc 150 detector . Data were measured using Cu-Kα radiation (1.54184 Å) and processed with CrysAlis^{PRO}. (1.171.42.94a (Rigaku OD, 2023). Data for structures m261, m192 and m102 were collected on a Rigaku Synergy-R diffractometer with HyPix ARC 100º detector, MoKα (λ=0.71073Å) and processed with CrysAlis^{PRO}. (1.171.42.94a (Rigaku OD, 2023). Data collection was performed under LN at 100 K. The structures were solved by direct methods using the SHELXT[2]. All non-hydrogen atoms were further refined by SHELXL[3] with anisotropic displacement coefficients. Hydrogen atoms were assigned isotropic displacement coefficients, U(H) = 1.2U(C) or 1.5U (C-methyl), and their coordinates were allowed to ride on their respective carbons. Refinement was carried out with the OLEX-2[4] GUI. Crystallographic data and refinement parameters are summarized in tables below.

[2] SHELDRICK, G. M. (2015). SHELXT – INTEGRATED SPACE-GROUP AND CRYSTAL-STRUCTURE DETERMINATION *ACTA CRYSTALLOGR.* SECT. A 71, 3-8

[3] SHELDRICK, G. M. (2015). CRYSTAL STRUCTURE REFINEMENT WITH SHELXL. *ACTA CRYSTALLOGR*. SECT. C 71, 3-8

[4] DOLOMANOV, O. V., BOURHIS, L. J., GILDEA, R. J., HOWARD, J. A. K., AND PUSCHMANN, H. (2009). OLEX2: A COMPLETE STRUCTURE SOLUTION, REFINEMENT AND ANALYSIS PROGRAM. *J. APPL. CRYSTALLOGR.* 42, 339–341.

Complex	m261benzoylated	m174benzoylated	m192benzoylated
**CCDC No.**	**2388963**	**2388965**	**2388966**
**Formula**	$C_{23} H_{23} N_3 O_2 S_3$	$C_{20} H_{18} N_2 O_2 S_2$	$C_{20} H_{20} N_2 O_3 S_2$
**Formula weight**	469.62	382.48	400.50
**Crystal system**<br>**Space group**	monoclinic<br>*P* 2_1	triclinic<br>*P* -1	monoclinic<br>*P* 2_1/c
**Crystal size**	0.23 x0.145 x0.094	0.28 x0.06 x0.04	0.20 x0.06x0.12
**Crystal color and shape**	Colorless plate	Yellow plate	Colorless plate
**Temperature (K)**	100	100	100
**wavelength (Å)**	0.71073	0.71073	1.54178
**a, (Å)**	6.6026(2)	9.3798(4)	17.5837(2)
**b, (Å)**	18.1812(6)	9.4728(3)	11.5509(1)
**c, (Å)**	9.1114(3)	11.8306(3)	9.4610(1)
**α, (°)**	90	103.699(2)	90
**β, (°)**	93.339(3)	96.970(3)	102.61(13)
**γ, (°)**	90	114.430(3)	90
**Volume ($Å^3$)**	1091.90(6)	900.81(6)	1875.26(3)
**Z**	2	2	4
**$\rho_{calcd,}$(g cm^{-1})**	1.428	1.410	1.419
**μ, mm^{-1}**	0.366	0.313	2.775
**F(000)**	492	400	840
**Reflections collected (unique)**	13892(8015)	34998(8015)	35498(3833)
**R_{int}**	0.0374	0.0329	0.0334
**Theta range for data collection (°)**	2.24-27.48	1.83-36.29	4.62-74.94
**Completeness to θ (%)**	91.5	99.9	99.5
**data / restraints / parameters**	4572/1/280	8015/0/235	3833/0/248
**goodness-of-fit on F^2**	1.032	1.055	1.059
**Final R_1 and wR_2 indices [$I > 2\sigma(I)$]**	0.0282,0.0688	0.0358,0.0897	0.0315,0.0839
**R_1 and wR_2 indices (all data)**	0.0294,0.0699	0.0482,0.0940	0.0344,0.0855
**Largest diff peak and hole**	0.281, -0.176	0.549, -0.238	0.351, -0.361

Table 4.1. X-ray crystallographic data for benzoylated complexes m261, m174, and m192.

CIF file name	m261	m192	m102
CCDC	2389626	2389627	2389628
Crystal description	Pink Prism	Orange needle	Colorless plate
Crystal size	0.13 x 0.06 x 0.04	0.42 x 0.04 x 0.03	0.12 x 0.10 x 0.07
Empirical formula	$C_9 H_{15} N_3 S_3$	$C_6 H_{14} N_2 O S_2$ + 2 Cl O_4	$C_3 H_7 N_2$ S + $C_2 F_3 O_2$
Formula weight (g/mol)	261.42	393.21	216.19
Temperature (K)	100(2))	102(3)	100(2)
Wavelength (Å)	0.71073	0.71073	0.71073
Crystal system	triclinic	triclinic	triclinic
Space group	*P*-1	*P*-1	*P*-1
a (Å)	5.7571(2)	5.53060(10)	6.41561(16)
b (Å)	7.5221(2)	10.14030 (10)	8.0795(2)
c (Å)	13.6102(4)	12.7240 (2)	8.7308(2)
α°	91.560(2)	88.2120(10)	64.341(3)
β°	90.824(2)	83.6760(10)	87.657(2)
γ°	100.337(2)	77.7800(10)	88.635(2)
Volume (Å^3)	579.50(3)	693.152(18)	407.58(2)
Z	2	2	2
Density calculated (Mg/m^3)	1.498	1.884	1.762
Absorption coefficient (mm^{-1})	0.610	0.816	0.418
F(000)	276	404	220
Theta range for data collection (°)	2.76 to 31.65	2.60 to 33.27	2.59 to 30.51
Reflection collected (Unique)	22309(3430)	9069(9069)	19816(2487)
R int	0.0310		0.0387
Completeness %	100.0	99.4	99.9
Data\restraints\parameters	3430 /0/ 144	9069/0/ 192	2487/0/118
Goodness-of-fit on F^2	1.047	1.060	1.040
Final R [I>2σ(I)]	R1=0.0294 wR2= 0.0722	R1=0.0348 wR2= 0.0973	R1=0.0332 wR2= 0.0861
R (all data)	R1=0.0378 wR2=0.0754	R1=0.0456 wR2=0.1018	R1=0.0423 wR2=0.0902
Largest diff. peak and hole (e·Å^{-3})	0.555 and -0.200	0.687 and -0.446	0.550 and -0.466

Table 4.2. X-ray crystallographic data for complexes m261, m192, m102

b) Formation of m261 in experiment with isopentenyl alcohol

The m261 crystals were serendipitously obtained from the NMR experiment conducted to investigate the effect of isopentenyl alcohol on the HCN-cysteamine reaction (initially related to main text Fig 5C), the detailed setup of which is described in Section 3. d) of this Supporting Information.

Briefly, the experiment was performed on a 1 ml scale in an NMR tube. A solution was prepared in D_2O containing cysteamine hydrochloride (227 mg, 2 mmol) and HCN (target concentration 2 M, 2 mmol). To this 1 ml mixture, 10 µL of isopentenyl alcohol (3-methylbut-2-en-1-ol) was added. The ampoule was sealed and monitored by ^1H NMR spectroscopy at 40°C for approximately 4 hours. After the NMR experiment, the tube was removed from the spectrometer and kept undisturbed at room temperature for 20 hours. During this period, crystals of m261 formed on the surface of the second liquid phase that had developed within the NMR tube. These crystals were then manually collected for X-ray diffraction analysis.

c) Isolation m192 using $HClO_4$ treatment of the second liquid phase

In this experiment, a freshly prepared second liquid phase obtained from an experiment involving dihydrogen phosphate was used. To prepare this phase, 260 mg of potassium cyanide (4 mmole) was dissolved in 2 mL of water and then 450 mg of cysteamine hydrochloride (4 mmole) and 540 mg of potassium dihydrogen phosphate (4 mmole) were added. To control the exothermic nature of the reaction and prevent excessive heating during its initial stages, the reaction mixture was cooled in an ice water bath for 15 minutes. Monitoring the mixture, the formation of a second liquid phase was indicated by the onset of solution turbidity. Once these initial signs were observed, the reaction vial was removed from the ice bath and allowed to equilibrate at room temperature for an additional 30 minutes.

The saturated solution resulted in the formation of a second liquid phase at the surface, attributed to the high density imparted by dissolved salts. After standing at room

temperature for 30 minutes, the second phase exhibited a color change from colorless to light brown while maintaining its transparency.

Subsequently, approximately 100 mg of the second liquid phase was dissolved in 1 mL of 30% perchloric acid ($HClO_4$). The acidified solution was left to evaporate under ambient conditions for two weeks, during which crystals of m192*$HClO_4$ formed as square plates.

d) Benzoylation of the second liquid phase: formation and isolation of m192benzoylated, m174benzoylated, and m261benzoylated.

Benzoylated products m192benzoylated, m174benzoylated, and m261benzoylated were obtained through several experiments following a unified protocol.

Typical protocol for synthesis of the second liquid phase:

Hydrogen cyanide (HCN, 380 mg, 14 mmol) was dissolved in water (7 mL), followed by the addition of cysteamine hydrochloride (0.8 g, 7 mmol). The molar ratio of HCN to cysteamine was maintained at 2:1 to increase the likelihood of forming heavier products in which the ratio of HCN units to cysteamine fragments is close to 2:1, as well as to compensate for potential HCN losses.

The reaction mixture was left at room temperature for 36 hours until a darkly colored second phase formed. The mixture was then extracted with dichloromethane (5 × 10 mL). The combined organic extracts were dried over anhydrous sodium sulfate (Na_2SO_4), filtered, and concentrated under reduced pressure to yield 300 mg of a transparent dark-red oil.

Typical protocol for benzoylation of the second liquid phase:

The resulting crude second liquid phase was dissolved in dichloromethane (10 mL) and benzoylated by adding benzoyl chloride (300 mg, 2.13 mmol) and pyridine (175 mg, 2.2 mmol). The reaction mixture was stirred at room temperature for 24 hours. After completion, the mixture was washed with water (3 ×) and saturated sodium bicarbonate

solution (3 ×), dried over anhydrous Na_2SO_4, filtered, and concentrated under reduced pressure to obtain 500 mg of dark oil.

Purification was performed using column chromatography on silica gel (30 g, 230–400 mesh) with a gradient mixture of dichloromethane and acetonitrile as the eluent, ranging from 1:0 to 1:2. All fractions were collected and assessed for purity by HPLC-MS. The purest fractions were used to attempt crystallization from a two-phase dichloromethane-pentane system. Using this method, crystals of m192benzoylated, m174benzoylated, and m261benzoylated were successfully obtained.

e) Boc protection, chromatography, and deprotection of the second phase to isolate m102

Product m102 was obtained through Boc protection of the second liquid phase, followed by purification and deprotection, as detailed below.

Freshly obtained second liquid phase (300 mg), prepared as described above, was dissolved in dichloromethane (10 mL). To this solution, di-tert-butyl dicarbonate (Boc_2O, 1.2 g, 2.87 mmol) and pyridine (250 mg, 3.16 mmol) were added. The reaction mixture was stirred at room temperature for 24 hours. After completion of the reaction, the mixture was washed with water (3 × 10 mL), dried over anhydrous sodium sulfate (Na_2SO_4), filtered, and concentrated under reduced pressure to yield a light-yellow oil. The crude product was purified by column chromatography on silica gel using a gradient elution of ethyl acetate and hexane (from 1:1 to 1:0). All fractions were collected and concentrated under reduced pressure.

Each fraction was redissolved in dichloromethane (1 mL), and trifluoroacetic acid (TFA, 500 μL) was added. The mixtures were left at room temperature for 2 hours. After evaporation under reduced pressure, each fraction was dissolved in approximately 1 mL of 70% ethanol and allowed to evaporate at room temperature. Crystals of m102 formed in one of the first fractions eluted from the column.

5. Mass spectrometry analysis of HCN-cysteamine reactions: techniques and methods

a) Selected experiments with isotopically labeled cyanides

The main goal of using labeled cyanides ($K^{13}CN$ and $KC^{15}N$) alongside regular cyanide was to compare mass spectra and identify distinct signals exhibiting isotopic shifts, thereby reliably identifying and characterizing the compounds formed during the reaction between HCN and cysteamine. However, due to the high volatility and potential loss of hydrogen cyanide over extended reaction times – especially at milligram scales – these experiments presented significant challenges. To mitigate this issue, the reactions were conducted under elevated pH conditions, effectively minimizing hydrogen cyanide losses and stabilizing reagent concentrations. Preliminary experiments with natural isotope abundance KCN and HCN confirmed that this pH adjustment allowed the reaction to proceed within a reasonable timeframe while preserving the major compound peaks in the mass spectra.

The experiments were carried out in 1 mL volumes under the following conditions:

- 1 M KCN + 0.5 M HCl + 1 M cysteamine
- 1 M KCN + 0.5 M cysteamine + 2 M KH_2PO_4
- 1 M KCN + 1 M cysteamine + 2 M KH_2PO_4
- 1 M KCN + 0.5 M cysteamine

Aliquots were taken at 30, 60, 120, and 300 minutes. These aliquots were dissolved in methanol and analyzed using both direct injection and HPLC-MS with column separation. For each set of three aliquots, mass spectra were obtained to compare and identify signals. In the case of direct injection, ES+ spectra were used directly for analysis. However, when using the HPLC column, chromatograms representing intensity over time were generated. Identical signals regions corresponding to retention times across all three spectra were selected for comparison and analysis. Notably, no compounds exhibiting isotopic shifts were detected in the ES- spectra. To enhance reliability, samples were reanalyzed at

intervals of 4 hours, 1 day, 2 days, and 4 days, which helped assess the stability of the signals and eliminate the influence of random noise. The collected data allowed for a more reliable distinction between signals demonstrating isotopic shifts and those resembling random noise.

b) Signal recognition and triplet identification

This section presents selected spectra with identified signals and accompanying comments. The examples illustrate the method used for selecting and identifying signals, including isotopic shifts. A summary table with the results of all identified signals is provided at the end of the section.

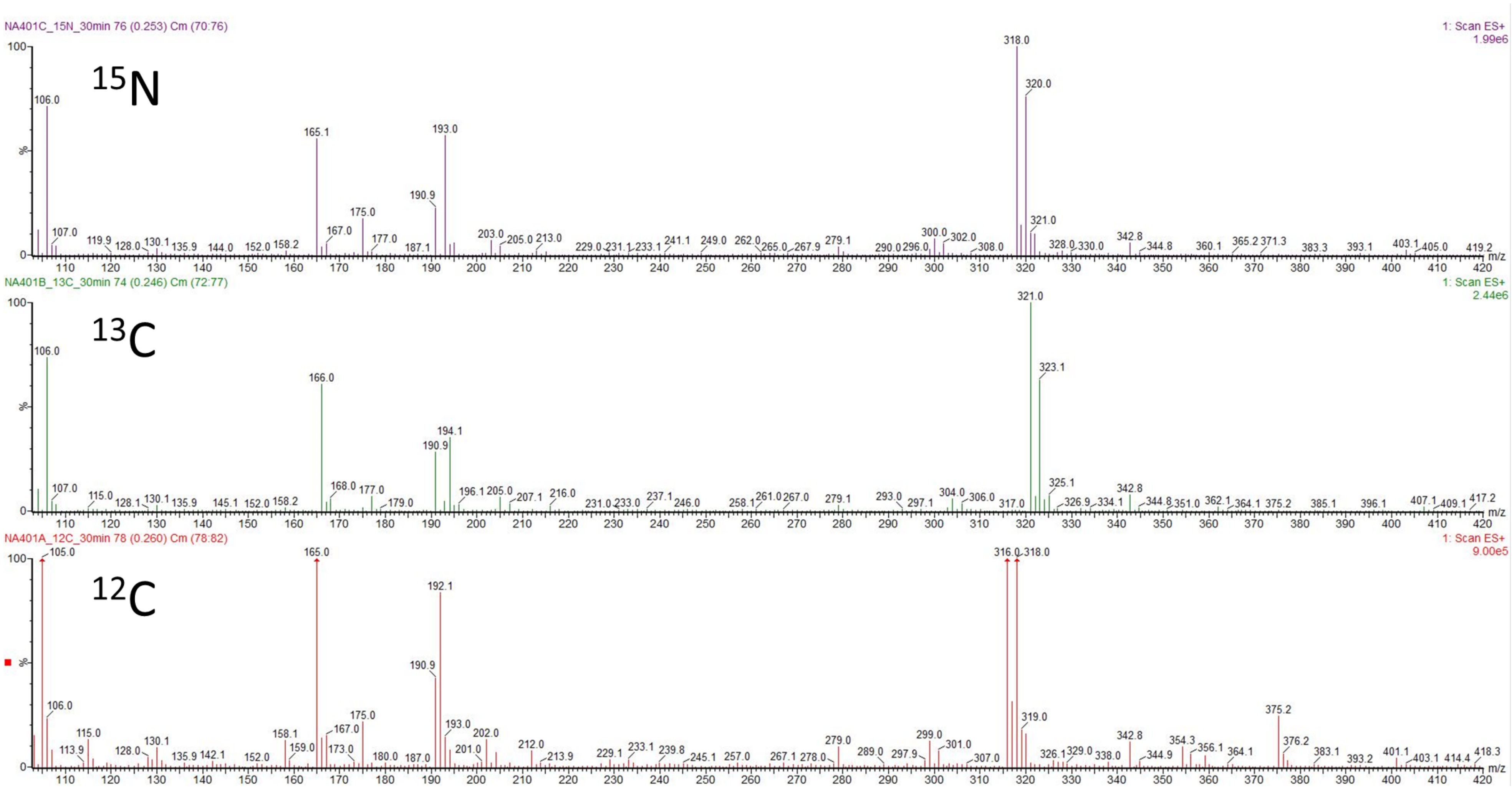

Figure 5.1. ESI-MS (+) spectra from direct injection of aliquots taken 30 minutes after initiation of parallel reactions. Conditions: 1 M KCN, 0.5 M HCl, 1 M cysteamine. *Bottom:* natural abundance KCN. *Middle:* $K^{13}CN$. *Top:* $KC^{15}N$. Isotopic shifts are used to determine $n(C_{HCN})$ and $n(N_{HCN})$

Analysis of Triplet Signals: The primary approach to identifying signals related to our system involves finding signals in the ^{12}C spectrum that shift several mass units to the right in the ^{13}C spectrum. This behavior is due to the incorporation of carbon atoms from the HCN molecule. Subsequently, the corresponding third component of the triplet is identified from the ^{15}N spectrum. These triplets are then extracted for further mathematical analysis.

In the current spectra, which are not zoomed in but still informative, the following triplets can be noted:

- **316-321-318** paired with its twin **318-323-320**, which dominate many spectra. Such pairs with a mass difference of two units are labeled as 'pairs' in the chemical reaction network due to their frequent occurrence.
- Their typical companions are **299-304-300** and **301-306-302**.

Over time, the heavier pair decreases in intensity, while the lighter one increases, suggesting that these pairs are related and transform into one another due to ammonia loss. It is highly likely that cyclization to a 5- or 6-membered ring occurs during this transformation, providing clues for constructing structural formulas.

In the unzoomed spectrum, additional triplets can also be identified:

- **105-106-105**
- **165-166-165**
- **175-177-175**

There are also other triplets that become more apparent upon zooming in on the spectra.

In the next figure provided, the mass spectra represent three parallel experiments similar to the previous set, but with aliquots taken at a different time point (90 minutes)

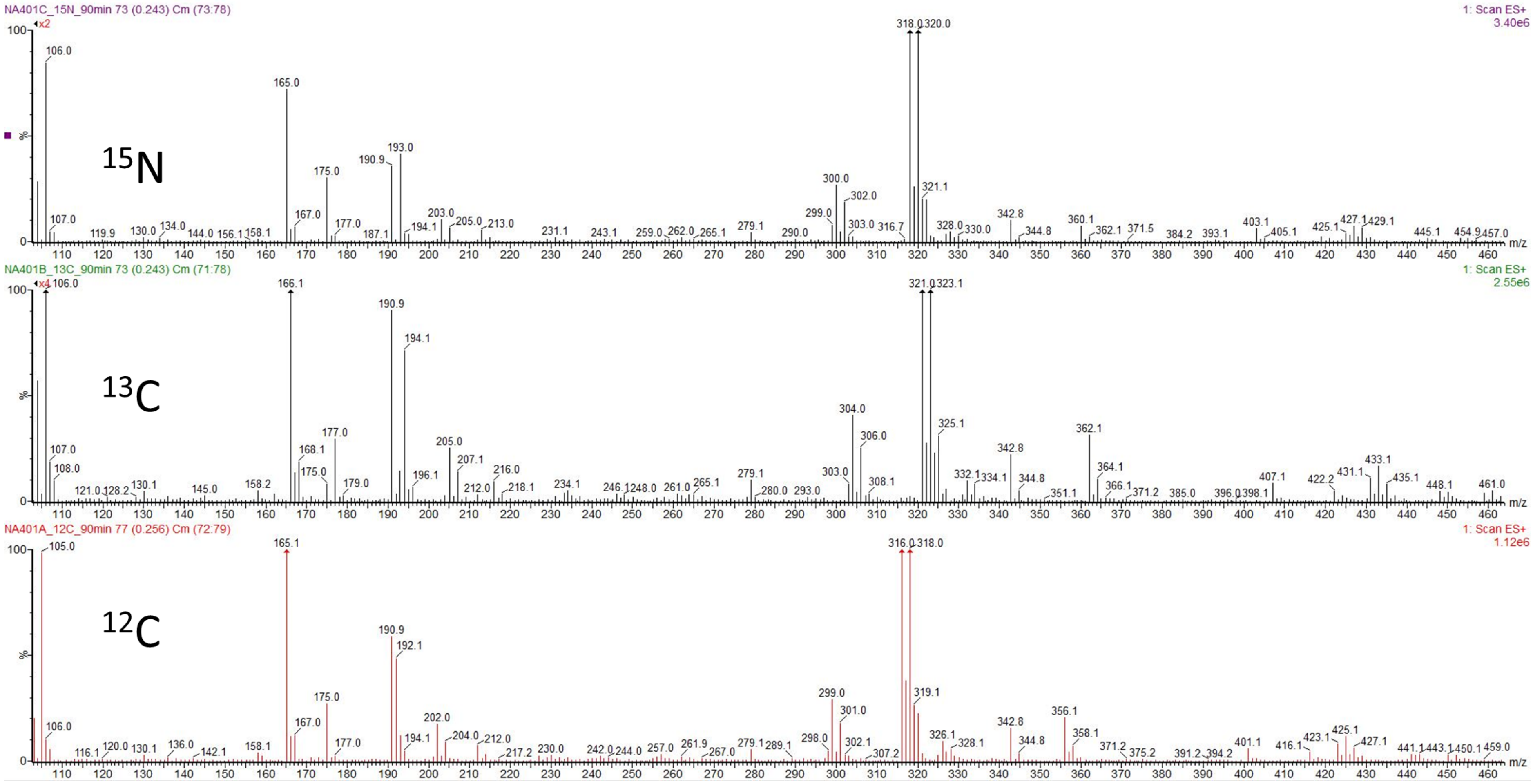

Figure 5.2. ESI-MS (+) spectra from direct injection of aliquots taken 90 minutes after initiation of parallel reactions. Conditions: 1 M KCN, 0.5 M HCl, 1 M cysteamine. *Bottom:* natural abundance KCN. *Middle:* $K^{13}CN$. *Top:* $KC^{15}N$.

- Pair of triplets such as **316-321-318** and **318-323-320** continue to dominate, suggesting consistent behavior over time.
- Companion triplets **299-304-300** and **301-306-302** are also observed, with similar trends in intensity change.
- Additional triplets such as **165-166-165** and **175-177-175** remain present, indicating their stability throughout the reaction process.

In the heavier products, three new triplets appear with the same step of 2 mass units:

- **423-431-425**
- **425-433-427**
- **427-435-429**

Additionally, a new pair is observed:

- **202-205-203**
- **204-207-205**

In our scheme, this slower formation is associated with the slower cyclization of a 6-membered ring.

Also, without zooming, another typical pair for our system is barely visible:

- **212-216-213**
- **214-218-215**

Overall, the evolution of the system is evident, with the accumulation of signals and the appearance of heavier signals containing 8 carbon atoms from HCN in their structure, as well as the emergence of new typical pairs of oxidized/reduced compounds.

Analysis of chromatograms For the next stage of the analysis, we utilized a chromatographic column (model: Jupiter 5 μm C4 300 Å, LC Column 50 x 4.6 mm) with a typical representative chromatogram shown below.

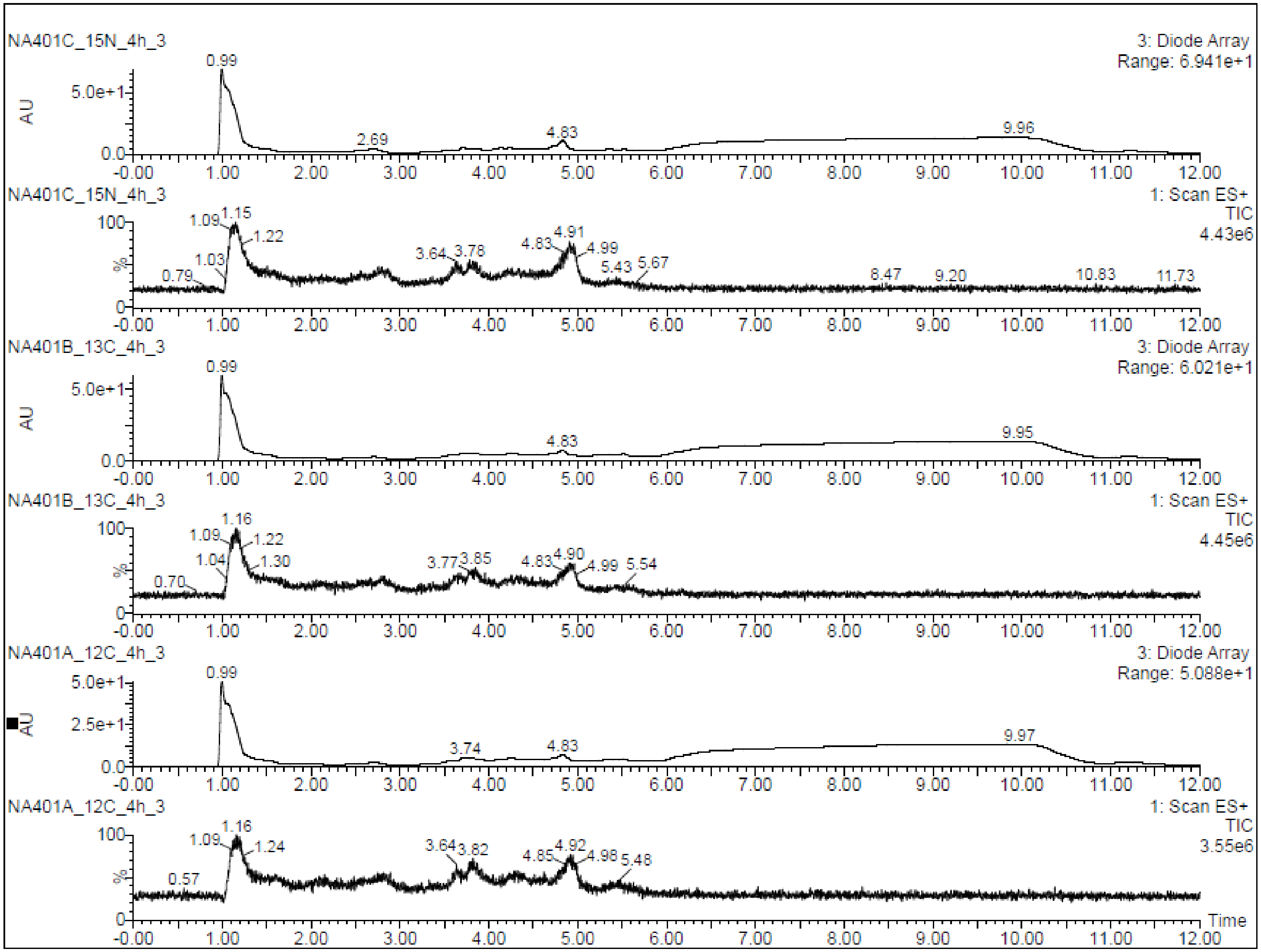

Figure 5.3. Representative HPLC-ESI-MS chromatograms (UV-Vis and ES+ Total Ion Current, TIC) of aliquots from parallel reactions, taken 4 hours after initiation. Conditions: 1 M KCN, 0.5 M HCl, 1 M cysteamine. LC separation: Jupiter 5 µm C4 300 Å column (50 x 4.6 mm). *Bottom pair:* natural abundance KCN. *Middle pair:* $K^{13}CN$. *Top pair:* $KC^{15}N$.

Analysis of similar chromatograms was performed by separately analyzing visible substance peaks on the chromatograms and isolating triplets from the specified regions. A breakdown of one of the regions of the presented chromatogram is provided below.

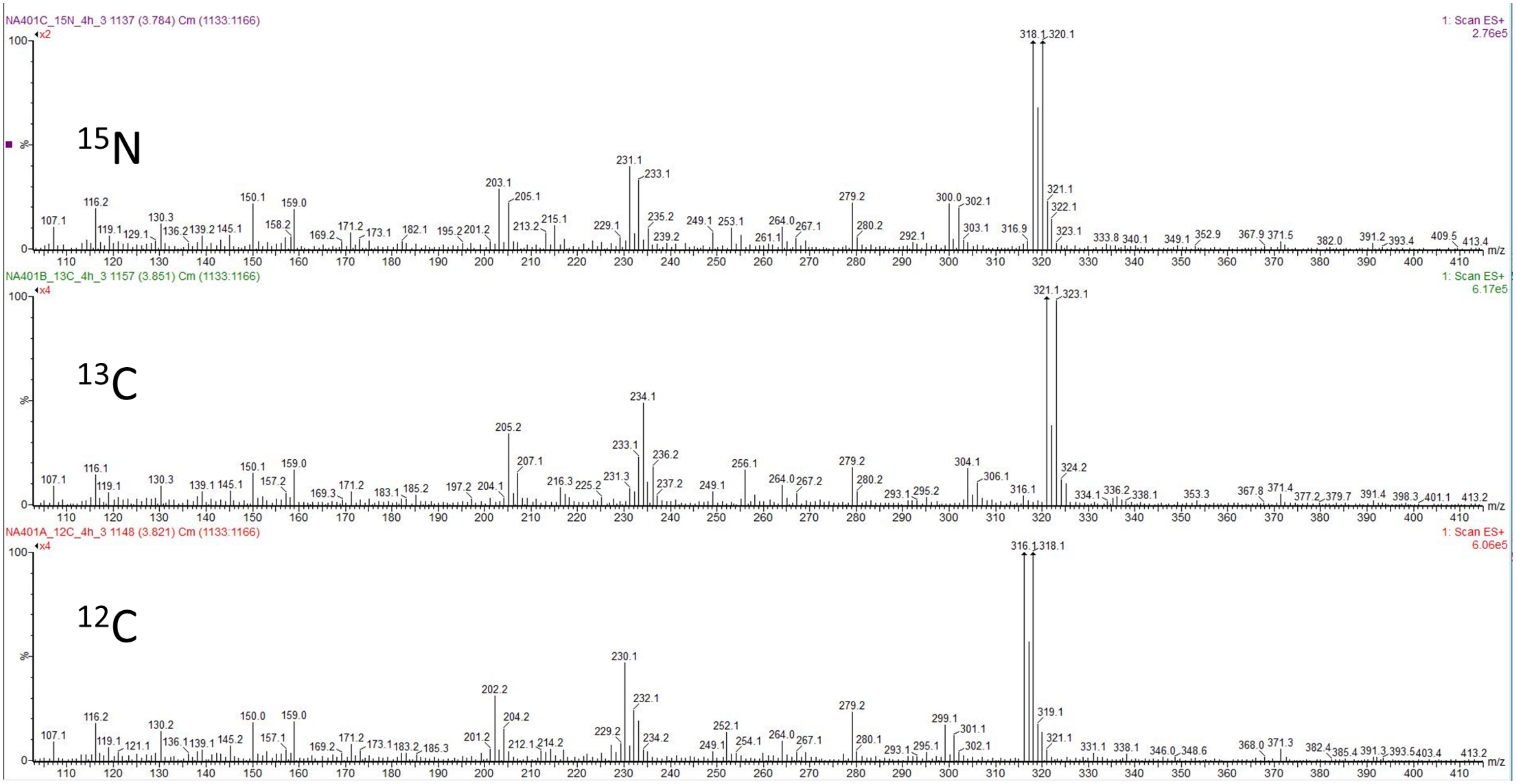

Figure 5.4. ESI-MS (+) spectra for the chromatographic peak eluting at ~3.78 minutes from the HPLC-MS analysis shown in Figure S5.3. Spectra correspond to parallel reactions with: *Bottom:* natural abundance KCN. *Middle:* $K^{13}CN$. *Top:* $KC^{15}N$. These data illustrate the selection of isotopic triplets for further computational analysis.

From the provided spectrum, it can be seen that this signal is predominantly represented by the pair **316-321-318** and **318-323-320**, with the presence of **299-304-300** and **301-306-302**. At the same time, recognizable pairs of triplets are:

- **202-205-203**
- **204-207-205**

Meanwhile, signals such as:

- **230-234-231**
- **232-236-233**

and minor signals like:

- **253-256-253**

were identified but did not pass the statistical validation and model decomposition tests, and therefore were not included in the final version of the reaction scheme.

c) Mathematical framework for data analysis

Our analysis is grounded in the simplicity of the chemical reaction space within the system, proposing that all complex transformations can be explained using just six fundamental reactions. While the exact structures of the end products may vary, this hypothesis provides valuable insights into the temporal evolution of the molecular composition.

This simplicity arises from the limited set of initial reactants: hydrogen cyanide (HCN), cysteamine, and water (H_2O). Cysteamine contains two nucleophilic centers—sulfur and nitrogen—but under the given reaction conditions, the breaking of C–N, C–C, S–C, or C–H bonds is unlikely. Therefore, cysteamine primarily reacts through its thiol (–SH) and amine (–NH_2) groups.

In contrast, HCN is more versatile. Its H–C and C≡N bonds can undergo various transformations, including polymerization and hydrolysis of the –CN group, leading to

ammonia elimination. HCN can also be attacked by nucleophiles like the sulfur or nitrogen atoms in cysteamine or the oxygen in water. Additionally, oxidation and reduction reactions are possible, with thiols acting as reducing agents and oxidants such as disulfides or atmospheric oxygen facilitating oxidation.

Based on these observations, we have formulated six core transformations that effectively capture the fundamental reactions occurring in the system (Figure 4 in main text):

1. **Addition of HCN**: Increases the molecular mass by 27 units and results in a +1 isotopic shift in both the ^{13}C and ^{15}N spectra.
2. **Addition of cysteamine**: Increases the molecular mass by 77 units without altering isotopic shifts.
3. **Addition of H_2O**: Increases the molecular mass by 18 units without changing isotopic shifts.
4. **Ammonia elimination (–NH_3)**: Decreases the molecular mass by 17 units and reduces the isotopic shift in the ^{15}N spectrum by 1.
5. **Oxidation**: Decreases the molecular mass by 2 units.
6. **Reduction**: Increases the molecular mass by 2 units.

While complex reactions such as isomerization or cyclization are not explicitly included, this model provides a practical framework for analyzing the system's chemical transformations, particularly when assuming that the cysteamine backbone (–S–CH_2–CH_2–N–) remains intact.

Using this simplified approach, we can deconstruct more complex molecules formed in the system by representing them as combinations of the six fundamental transformations. If a detected compound with mass **X** results from a reaction between substances **Y** and **Z**, each can be described by the sequence of transformations that led to their formation. Consequently, compound **X** can be represented as the cumulative sum of these reactions.

Our method leverages the unique impact of each transformation on both molecular mass and isotopic shifts, facilitating the identification of transformation pathways within the system. For example, frequently observed triplets in mass spectra—such as 175–177–175,

corresponding to a compound with a mass of 174—can be explained using this model. The isotopic shifts for the ^{13}C and ^{15}N experiments are +2 and 0, respectively. This pattern can be interpreted as the result of six transformations: two additions of HCN, two additions of cysteamine, and two ammonia eliminations, starting from an initial state with no molecules. Finding an alternative combination of basic reactions to explain such a signal is challenging. Moreover, the model's proposed pathway aligns with experimental evidence, such as the X-ray crystal analysis of m174-benz.

To simplify the analysis of compounds detected via mass spectrometry using isotopic shifts, we developed the **IVO algorithm**. This algorithm systematically searches for the simplest combination of the six proposed reactions that accounts for the observed molecular mass and isotopic shifts. By ensuring that the transformation pathways are optimal and consistent with the detected isotopic data, the IVO algorithm provides reliable insights into the chemical transformations occurring within the system.

d) Data processing and analysis using MATLAB and IVO algorithm

We developed a MATLAB script to analyze triplets of ion masses obtained from experimental ^{12}C, ^{13}C, and ^{15}N mass spectrometry spectra. The script calculates the combination of six fundamental chemical reactions that lead to compounds with the observed masses and isotopic shifts. When a new compound is identified, its data are automatically added to the data_names table. Additionally, a comprehensive summary table is created, which includes ^{13}C and ^{15}N shifts, the number of cysteamine fragments (N(cys)), and the frequency of observation, along with the reaction breakdown.

Additionally, to avoid working with sets of numbers, each compound was assigned a simple name consisting of three syllables and one digit. This naming scheme made it easier to reference compounds, search through tables, and work on constructing the chemical reaction network. These compounds and their corresponding names are presented in the tables below.

The collected data from all decompositions are presented in the tables below. The first table includes comprehensive data for each compound analyzed by the script.

- **x1**: Number of reactions involving the addition of HCN
- **x2**: Number of reactions involving the addition of cysteamine
- **x3**: Number of reactions involving the addition of H_2O
- **x4**: Number of reactions involving the elimination of NH_3
- **x5**: Number of oxidation reactions
- **x6**: Number of reduction reactions

Name	x1	x2	x3	x4	x5	x6	Name	x1	x2	x3	x4	x5	x6
lumeru1	1	1	0	0	1	0	memoro4	5	3	0	4	1	0
berana0	1	1	0	1	0	0	belemi2	5	3	0	4	0	0
beruna1	1	1	0	1	0	1	bumiro8	5	3	0	4	0	1
lareru8	1	1	0	0	0	0	mimuri1	5	3	0	4	0	2
bulelu7	1	1	1	1	0	0	bilemi8	5	3	0	3	1	0
mamero4	1	1	1	1	0	3	lemora2	5	3	0	3	0	0
bumeru8	1	2	0	0	34	0	bureri8	5	3	1	4	0	0
leluma6	1	2	0	1	31	0	lemori0	5	3	0	3	0	1
lumare4	1	1	2	1	0	2	buleli9	5	3	1	4	0	1
limari8	2	1	0	0	1	0	lalime1	5	3	0	3	0	2
bolimu5	2	1	1	1	0	0	lamere1	6	2	4	4	0	2
bomori4	1	1	3	1	2	0	bilora5	6	3	0	4	0	0
bomemi2	3	1	0	1	0	0	boburu0	6	3	2	6	0	0
limere4	3	1	2	3	0	0	belore4	6	3	0	4	0	1
mimora0	4	1	0	2	3	0	lilome8	6	3	0	4	0	2
bilolu6	1	2	0	1	9	0	laluru3	5	3	0	0	18	0
laliru5	1	2	0	1	0	0	bulire9	6	3	1	4	3	0
buroru2	3	1	2	2	0	3	bamura8	5	3	2	4	0	2
rariro2	1	2	0	1	0	1	mamoro0	6	3	1	4	3	0
liluro3	2	2	0	2	1	0	lemame3	6	3	1	4	2	0
lereru8	2	2	0	2	0	0	bobule9	5	3	2	4	0	3
bolomo1	3	2	0	2	13	0	bumemu2	7	3	0	4	0	1
bibole2	1	2	0	0	3	0	laluma0	6	3	0	2	2	0
mamora1	2	2	0	2	0	1	mamuru9	7	3	0	4	0	2
moreru8	1	2	0	0	1	0	bolere4	6	3	0	2	1	0
limara6	2	2	1	2	3	0	bamaru6	5	3	2	2	0	5
bimuro7	2	2	1	2	3	0	bolalo3	5	3	3	2	3	0
lomemu7	3	1	3	1	2	0	bumora2	2	2	0	1	0	0
bulumi9	3	2	0	2	1	0	loluro6	5	4	0	3	1	0
bilare6	1	2	1	0	0	1	bolemi8	5	3	3	2	0	3
babibo0	3	2	0	2	0	0	buliro4	6	4	0	4	1	0
bomoru6	3	2	0	2	0	1	lumara4	5	4	1	4	0	5
bilame0	1	2	1	0	0	2	loreru3	6	4	2	6	0	0

belori5	4	2	0	3	0	0	|	bamira7	6	4	0	4	0	1
bomore6	4	2	0	3	0	1	|	mamoru3	4	4	1	2	0	2
baboli3	4	2	0	3	0	1	|	bebole0	6	4	0	4	0	2
boloma9	4	2	0	3	0	1	|	bilara2	4	4	1	2	0	3
bilalo6	2	2	1	1	0	2	|	balaru6	6	5	0	4	0	2
muroru9	4	2	0	3	0	2	|	bomiri8	7	4	0	5	0	1
liraru1	2	2	1	1	0	3	|	bolumu0	8	4	0	5	12	0
belimo3	4	2	0	2	1	0	|	limemi7	6	4	2	5	3	0
bomure6	3	2	1	2	0	4	|	lilumu8	6	4	0	3	2	0
bulime7	4	2	0	2	0	0	|	lumeri1	7	4	0	5	0	2
lamara1	4	2	0	1	8	0	|	lerero8	6	4	0	3	1	0
baluru5	4	2	1	3	0	0	|	bolume9	6	4	1	4	1	0
lireru1	4	2	0	1	7	0	|	liriru6	6	4	1	4	0	0
lemami9	3	2	0	0	2	0	|	belemu9	8	4	0	6	0	0
biliru8	4	2	1	3	0	3	|	lumoru9	8	4	0	4	16	0
lumame8	5	2	0	3	0	0	|	lelolu4	8	4	0	4	16	0
lomuro4	4	2	2	3	3	0	|	belale7	7	4	1	5	3	0
bomomu9	4	2	2	3	2	0	|	lilomo8	8	4	0	6	0	1
lumera1	4	2	0	1	1	0	|	meriro6	8	4	0	4	15	0
belima7	4	3	0	2	30	0	|	bolema6	8	4	0	6	0	2
bimoro3	4	2	2	3	1	0	|	bibume1	7	4	1	5	2	0
leroru9	4	2	0	1	0	0	|	lelumi1	8	4	2	8	0	2
lalure6	4	2	2	3	0	2	|	laleme1	8	4	0	6	0	3
lemara3	4	2	2	3	0	3	|	biraro8	7	4	1	5	0	5
balera7	5	2	0	2	0	0	|	biburu5	8	4	1	6	0	0
bebule4	4	2	1	2	0	5	|	maroru0	7	4	0	3	3	0
bebimu2	5	2	1	3	0	0	|	bumuro1	7	4	2	5	3	0
babilu1	5	2	0	2	0	1	|	bumuri0	8	4	1	6	0	1
lilura7	5	2	1	3	0	1	|	bamemu0	9	4	0	6	0	0
lelura7	5	3	0	2	37	0	|	biboru5	9	4	0	6	0	1
belure2	3	3	0	3	0	0	|	lalire8	7	4	1	4	0	3
balume1	5	3	0	4	17	0	|	leloro0	8	4	1	5	0	5
boliri9	5	1	7	4	3	0	|	bulumo1	8	4	0	3	2	0
bibolu9	6	3	0	4	29	0	|	moraro7	6	5	0	3	3	0
bemara0	5	2	3	4	2	0	|	bulara7	6	5	3	5	0	0
bararo8	5	2	3	4	1	0	|	bebula8	6	5	3	5	0	1
bemuru8	3	3	0	2	1	0	|	bililo8	7	5	1	4	3	0
bumera0	5	2	2	3	0	2	|	bibulu7	4	7	0	3	32	0
mariru2	3	3	0	2	0	0	|	momuru3	6	7	1	5	0	4
balemu2	5	2	2	3	0	3	|	murero2	5	7	2	4	0	1
balare6	4	3	0	3	0	0	|	babimi9	5	8	0	5	9	0
lamure5	4	3	0	2	7	0	|	lorare8	10	8	1	8	1	0
bamiri7	3	3	0	1	2	0	|	memoru6	10	8	1	8	0	0
mareri0	4	3	0	2	6	0	|	lalumi0	10	8	1	8	0	1
bumiri1	3	3	0	1	1	0	|	lamuro8	10	8	1	8	0	2

Table 5.1. compound names with reaction breakdown from MATLAB analysis, showing occurrences of HCN addition, cysteamine addition, H_2O addition, NH_3 elimination, oxidation, and reduction.

The second table is a filtered selection with additional data and frequency of observation, focusing on compounds observed more than three times and excluding those with implausible reaction counts (e.g., an unrealistically high number of oxidation/reduction reactions). This curated dataset highlights the most relevant compounds for our analysis.

- **OF** - frequency of observation
- **13C** – isotope shift when $K^{13}CN$ used
- **15N** – isotope shift when $KC^{15}N$ used
- **cys** – calculated number of cysteamin fragment
- **name** – generated name for practical use
- **mass** – molecular mass of compound
- **+HCN –** number of reactions involving the addition of HCN
- **+cys** – number of reactions involving the addition of cysteamine
- **+H2O** – number of reactions involving the addition of H_2O
- **-NH3** – number of reactions involving the elimination of NH_3
- **-2H** – number of oxidation reactions
- **+2H** – number of reduction reactions

OF	13C	15N	cys	name	mass	+HCN	+cys	+H2O	-NH3	-2H	+2H	Total
9	1	0	1	berana0	87	1	1	0	1	0	0	3
9	1	0	1	beruna1	89	1	1	0	1	0	1	4
10	1	1	1	lumeru1	102	1	1	0	0	1	0	3
15	1	1	1	lareru8	104	1	1	0	0	0	0	2
8	1	0	1	bulelu7	105	1	1	1	1	0	0	4
4	1	0	1	lumare4	127	1	1	2	1	0	2	7
7	2	1	1	bolimu5	132	2	1	1	1	0	0	5
6	3	2	1	bomemi2	141	3	1	0	1	0	0	5
15	1	0	2	laliru5	164	1	2	0	1	0	0	4
13	2	0	2	liluro3	172	2	2	0	2	1	0	7
28	2	0	2	lereru8	174	2	2	0	2	0	0	6
3	1	1	2	bibole2	175	1	2	0	0	3	0	6
9	1	1	2	moreru8	179	1	2	0	0	1	0	4

15	2	1	2	bumora2	191	2	2	0	1	0	0	5
5	3	2	2	lomemu7	191	3	1	3	1	2	0	10
31	3	1	2	babibo0	201	3	2	0	2	0	0	7
24	3	1	2	bomoru6	203	3	2	0	2	0	1	8
46	4	1	2	belori5	211	4	2	0	3	0	0	9
23	2	1	2	bilalo6	213	2	2	1	1	0	2	8
13	4	1	2	bomore6	213	4	2	0	3	0	1	10
4	4	1	2	bomore6	213	4	2	0	3	0	1	10
28	4	2	2	belimo3	226	4	2	0	2	1	0	9
7	4	2	2	bulime7	228	4	2	0	2	0	0	8
4	4	1	2	baluru5	229	4	2	1	3	0	0	10
7	3	3	2	lemami9	231	3	2	0	0	2	0	7
10	4	3	2	lumera1	243	4	2	0	1	1	0	8
4	5	3	2	balera7	255	5	2	0	2	0	0	9
14	5	2	2	bebimu2	256	5	2	1	3	0	0	11
9	5	2	2	lilura7	258	5	2	1	3	0	1	12
7	3	0	3	belure2	261	3	3	0	3	0	0	9
5	5	1	1	boliri9	264	5	1	7	4	3	0	20
5	5	1	2	bemara0	271	5	2	3	4	2	0	16
4	3	1	3	bemuru8	276	3	3	0	2	1	0	9
4	3	1	3	mariru2	278	3	3	0	2	0	0	8
6	4	1	3	balare6	288	4	3	0	3	0	0	10
5	3	2	3	bamiri7	291	3	3	0	1	2	0	9
4	3	2	3	bumiri1	293	3	3	0	1	1	0	8
43	5	1	3	belemi2	298	5	3	0	4	0	0	12
43	5	1	3	bumiro8	300	5	3	0	4	0	1	13
5	5	1	3	mimuri1	302	5	3	0	4	0	2	14
21	5	2	3	lemora2	315	5	3	0	3	0	0	11
5	5	1	3	bureri8	316	5	3	1	4	0	0	13
23	5	2	3	lemori0	317	5	3	0	3	0	1	12
4	5	2	3	lalime1	319	5	3	0	3	0	2	13
15	6	2	3	bilora5	325	6	3	0	4	0	0	13
12	6	2	3	belore4	327	6	3	0	4	0	1	14
4	6	2	3	lilome8	329	6	3	0	4	0	2	15
19	6	4	3	laluma0	355	6	3	0	2	2	0	13
8	6	4	3	bolere4	357	6	3	0	2	1	0	12
6	6	3	4	lilumu8	415	6	4	0	3	2	0	15
12	8	2	4	lilomo8	424	8	4	0	6	0	1	19
12	8	2	4	bolema6	426	8	4	0	6	0	2	20
6	9	3	4	bamemu0	449	9	4	0	6	0	0	19
9	9	3	4	biboru5	451	9	4	0	6	0	1	20

Table 5.2. Filtered compound data with observation frequency, isotope shifts, cysteamine count, and reaction breakdown for relevant compounds.

The analysis of the decomposed mass spectrometry data allowed us to identify possible relationships between substances within the system. Our primary approach involved constructing transformation pathways, comprising sequences of the six fundamental

reactions, to link substances that differ by a single unit in one of the reaction counts. This method enabled us to infer likely transitions and structural similarities between molecules.

For example, consider the substance pairs with masses 191 and 174, 315 and 298, and 317 and 300. Each pair differs by one unit in the count of ammonia elimination reactions. Specifically:

- Mass 191 corresponds to reaction counts (2, 2, 0, 1, 0, 0), while mass 174 corresponds to (2, 2, 0, 2, 0, 0).
- Mass 315 corresponds to (5, 3, 0, 3, 0, 0), while mass 298 corresponds to (5, 3, 0, 4, 0, 0).
- Mass 317 corresponds to (5, 3, 0, 3, 0, 1), while mass 300 corresponds to (5, 3, 0, 4, 0, 1).

In each case, the difference between the substances is the addition of one ammonia elimination reaction. This suggests that these pairs are related by the same transformational step – the elimination of ammonia and implies structural similarity between the molecules within each pair.

Similarly, we identified a relationship between a substance with a known structure of mass 261 (reaction counts: 3, 3, 0, 3, 0, 0) and a substance of mass 278 (3, 3, 0, 2, 0, 0). By analyzing the number of fragments involved in the reactions and observing general patterns, we constructed characteristic structures for the system. However, it is important to note that while this approach provides valuable insights, it does not allow for definitive conclusions about the exact structures of heavier compounds. This limitation arises from the lack of direct observations and evidence for molecules containing more than three HCN fragments in their core. Additionally, the model does not account for significant transformations such as rearrangements.

Our assumption regarding the formation of six-membered rings in heavier molecules is based on the observed change in the ratio of cyanide to cysteamine fragments. For heavier molecules, this ratio shifts from the 1:1 ratio common in molecules with five-membered rings to a 2:1 ratio, which corresponds to the formation of six-membered rings. Despite

this hypothesis, the mechanisms underlying the reduction and oxidation processes in the system remain unclear. It is likely that these processes occur in multiple stages, potentially involving transitions such as $2HS^- \leftrightarrow S–S$ or more complex mechanisms associated with the decarboxylation of intermediate products.

In conclusion, despite its limitations, the proposed model of six fundamental reactions serves as a powerful tool for analyzing this complex system. It allows us to make well-grounded assumptions and forms the basis for proposing transformations and structural formulas, providing valuable insights into the chemical evolution occurring within the system.

6. UV-Vis experiments

a) Hydrolysis rate of PNPA in the presence of the second liquid phase

Preparation of phosphate buffer solution (200 mM, pH 7.0): A fresh phosphate buffer was prepared by dissolving 0.933 g of KH_2PO_4 (0.00686 mol) and 2.290 g of K_2HPO_4 (0.01314 mol) in deionized water. The solution was transferred to a 100 mL volumetric flask and diluted to the mark with deionized water.

Preparation of 0.025 M PNPA solution in acetonitrile: A 0.025 M solution of *p*-nitrophenyl acetate (PNPA) was prepared by dissolving 8.2 mg of PNPA in 2 mL of acetonitrile. This stock solution was used for all subsequent reactions.

Synthesis of the second liquid phase: The second liquid phase was synthesized by reacting 2 M hydrogen cyanide with 2 M cysteamine hydrochloride at 40 °C for 4 hours. The resulting product was a moderately viscous, reddish-brown liquid, indicating successful formation of the desired phase.

Measurement of PNPA hydrolysis rate: To measure the hydrolysis rate of PNPA, 100 mg of the second liquid phase was added to vials containing 10 mL of the phosphate buffer solution (200 mM, pH 7.0) and 200 μL of the 0.025 M PNPA solution. This setup yielded a final PNPA concentration of 5×10^{-4} M in the reaction mixture. The mixtures

were stirred continuously using magnetic stirrers at room temperature. The experiment was conducted in triplicate, using three separate samples.

Sampling and analysis: At time intervals of 5, 20, 35, 65, and 95 minutes, 1 mL aliquots were withdrawn from the reaction mixtures. Each aliquot was immediately diluted by adding 1 mL of the phosphate buffer solution, resulting in a two-fold dilution. The diluted samples were filtered through a 0.22 µm syringe filter and transferred to a 10 mm pathlength cuvette. Absorbance at 405 nm was measured using a UV-Vis spectrophotometer.

Control experiments: 3 control experiments were conducted under identical conditions without the addition of the 100 mg of the second liquid phase. Absorbance measurements for both experimental and control samples were recorded and are presented in the accompanying table.

	**5 min**	**20 min**	**35 min**	**65 min**	**95 min**
Sample 1	*0.167*	*0.380*	*0.557*	*0.703*	*0.747*
Sample 2	*0.313*	*0.547*	*0.670*	*0.798*	*0.847*
Sample 3	*0.260*	*0.516*	*0.651*	*0.806*	*0.860*
Blank 1	*0.0320*	*0.0755*	*0.120*	*0.212*	*0.308*
Blank 2	*0.0295*	*0.0760*	*0.123*	*0.210*	*0.301*
Blank 3	*0.0298*	*0.0756*	*0.121*	*0.209*	*0.293*

Table 6.1. Absorbance measurements for PNPA hydrolysis in the presence of the second liquid phase and control samples at 405 nm.

Data analysis: PNP concentrations were determined using the Beer–Lambert law, accounting for the two-fold dilution of samples:

$$C_{PNP} = \left(\frac{A_{405}}{\varepsilon * l}\right) * 2$$

where:

- A is the average absorbance at 405 nm,

- ε is the molar absorptivity coefficient of PNP (9160 L mol^{-1} cm^{-1} for pH=7.0[5]),
- *l* is the pathlength of the cuvette (1 cm),
- multiply by 2 to account for the two-fold dilution of the aliquots before measuring absorbance

b) Continuous pH monitoring and thiol concentration measurement in HCN-cysteamine reaction

Hydrogen cyanide (400 mg, 14 mmol) was dissolved in 7 g of deionized water in a vial equipped with a lid allowing for the insertion of a pH meter electrode (e.g., Metrohm 913 pH meter). Cysteamine hydrochloride (1600 mg, 14 mmol) was then added to the HCN solution. The vial was placed in a water bath maintained at 40°C, and the pH electrode was installed for continuous pH monitoring.

At the start of the reaction, a 10 µL aliquot of the reaction mixture was withdrawn, and the vial was resealed hermetically. Sampling was repeated every 15 minutes throughout the reaction duration. Each 10 µL aliquot was diluted into 1 mL of deionized water. Subsequently, 15 µL of the diluted sample was transferred into a 2 mL cuvette containing 0.5 mM Ellman's reagent in 100 mM phosphate buffer at pH 7.0. The mixture was thoroughly mixed to ensure complete reaction, and the absorbance of the solution was measured at 412 nm using a UV-Vis spectrophotometer.

The recorded absorbance data was converted to thiol concentrations using the formula for thiol concentration calculation,

$$C = \frac{A_{412}}{\varepsilon * l} * D$$

where A_{412} is the absorbance at 412 nm, ε is the molar extinction coefficient of the TNB^{2-} ion (14,150 M^{-1} cm^{-1}), l is the path length of the cuvette (1 cm), and D is the dilution factor (D=1000*2000/(10*15)=13333).

[5] ***Trans. Faraday Soc.***, 1954,**50**, 800-802, https://pubs.rsc.org/en/content/articlepdf/1954/tf/tf9545000800

Resulting data is:

time	A_{412}	concentration
0	2.34	2.204947
15	2.2	2.073027
30	2.2	2.073027
45	2.15	2.025913
60	2.21	2.08245
75	1.94	1.828033
90	1.68	1.583039
105	1.08	1.017668
120	0.628	0.591755
135	0.383	0.360895
150	0.245	0.23086
165	0.225	0.212014
180	0.2	0.188457

Table 6.2. Absorbance and calculated thiol concentrations over time for HCN-cysteamine reaction with continuous pH monitoring.

The pH of the reaction mixture was continuously monitored (one record per minute) using a pH meter electrode inserted into the vial, and the resulting data from the instrument were directly plotted on a graph.

7. DLS and microscopy characterization of the second liquid phase

a) DLS studies of the formation of the second liquid phase.

To study possible molecular aggregation processes preceding the formation macroscopic droplets we performed *in situ* DLS studies of the HCN/cysteamine hydrochloride reaction. Cysteamine hydrochloride (341 mg, 3.00 mmol) was dissolved in 1.5 mL of a freshly prepared 2 M HCN water solution. The reaction mixture was filtered through 0.2 µm syringe filter and placed into DLS instrument. The mixture was thermo-equilibrated for 10 minutes and scans were performed every 15 minutes (Figure 7.1).

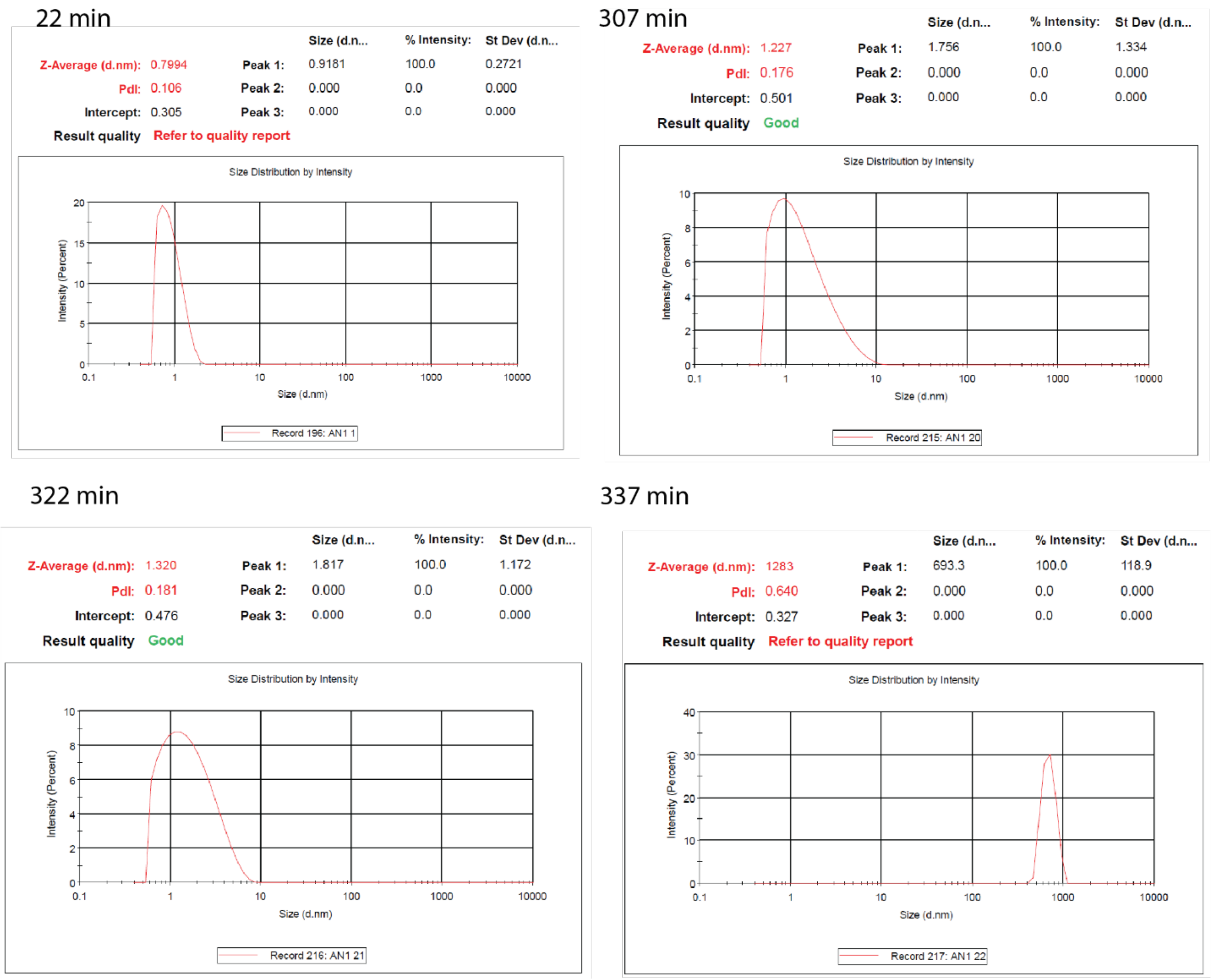

Figure 7.1. Selected DLS spectra for the reaction between HCN (2M) and cysteamine hydrochloride (2M) in water at 25 °C. Spectra from 22 to 292 minutes look similar to the spectrum at 22 minutes and indicate absence of large than molecular size objects.

Dynamic light scattering (DLS) measurements indicate that no aggregation was detected up to 292 minutes. Between 307 and 322 minutes, small aggregates approximately 1 nm in size may have begun to form; however, their dimensions are insufficient to suggest the formation of supramolecular structures such as micelles or supramolecular polymers. Between 322 and 337 minutes, phase separation occurs, resulting in the formation of micron-sized droplets that subsequently sediment.

b) Light microscopy studies of the second liquid phase.

To probe whether the second liquid phase contains any solid particles and crystallization accompanies the phase separation, we observed the suspension of the second liquid phase immediately after its formation by inverted light microscope (Nikon Ti2E) (Figure 7.2). Cysteamine hydrochloride (341 mg, 3.00 mmol) was dissolved in 1.5 mL of a freshly prepared 2 M HCN water solution. The mixture was visually observed. As soon as cloudiness of the solution was visually detected, the drop of the suspension was transferred to the glass slide and observed under a microscope using bright-field and DIC-contrast modes. The droplets looked and behaved as regular liquid, no anomalies were detected.

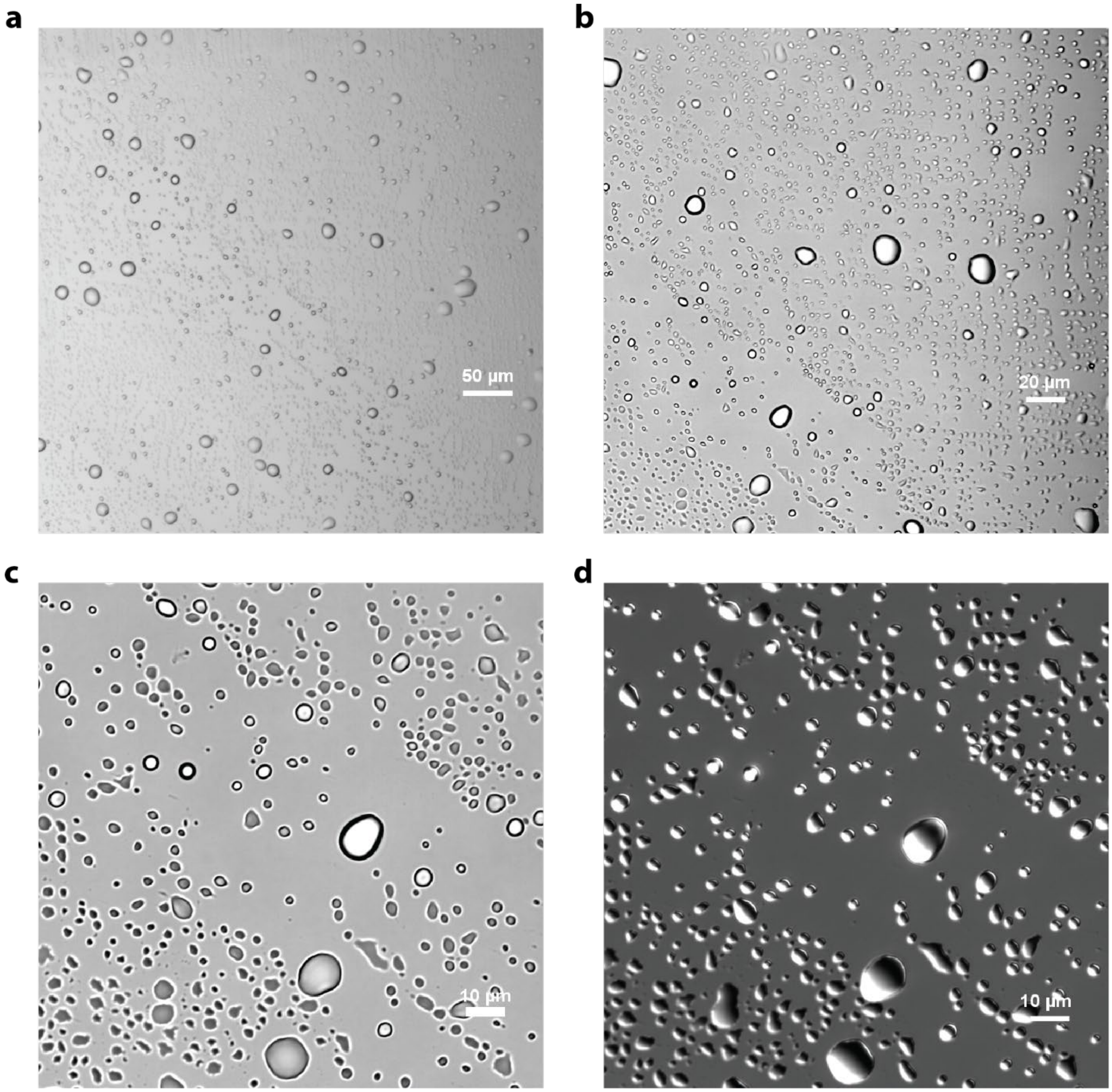

Figure 7.2. Bright-field (**a-c**) and differential interference contrast (DIC) (**d**) images of the droplets formed in the reaction between HCN (2M) and cysteamine hydrochloride (2M) in water at 25 °C. Images **a-c** differ in magnification.

8. Computational Deconstruction

The chemistry-inspired graph rewrite software mød[6] was used to find computational plausible reaction pathways from the initial molecules HCN and Cystamine to the molecules depicted in Fig. 4. First a set of graph-rewrite rules were written, which formalize the major reaction mechanisms supposed to operate under the experimental conditions. A set of reaction rules allows for the computational construction of the chemical reaction space (in the form of a reaction network) via iterative application of the reaction rules to the growing set of generated compounds, starting from the initial molecules. Once the chemical reaction space is constructed, finding a pathway between chemical compounds in the reaction network can be reformulated as an integer hyperflow problem on the reaction network.[7] To get chemically meaningful pathways, the number of reactions within a pathway and the amount of flow through the reactions in the pathway is minimized during the solving of the integer hyperflow problem. Finally, the causal order of reactions along the pathway is determined by computing a realizability certificate.[8] An iterative forward construction of the chemical reaction space and subsequent path search, turned out to be computationally infeasible, due to the combinatorial complex nature of the HCN polymerization chemistry. Therefore, instead of a forward construction of the full chemical space to all compounds in Fig. 4, a deconstruction space was calculated for each compound in Fig. 4 individually. In essence the reaction operators were applied iteratively in reverse direction to a compound of interest until the initial compounds HCN and Cystamine were reached and no further deconstructions were possible. The deconstruction pathway is, as in the forward approach, found by solving the integer hyperflow problem from the compound of interest to the initial compounds in the deconstruction space. The deconstruction pathways of all the compounds, except for the two largest ones, can be

[6] J. L. Andersen, C. Flamm, D. Merkle, P. F. Stadler, "A Software Package for Chemically Inspired Graph Transformation." *LNCS,* **2016**, 9761, 73.
[7] J. L. Andersen, C. Flamm, D. Merkle, P. F. Stadler, "Chemical Transformation Motifs — Modelling Pathways as Integer Hyperflows." *IEEE/ACM Trans Comp Biol Bioinf,* **2019**, 16(2), 510.
[8] J. L. Andersen, S. Banke, R. Fagerberg, C. Flamm, D. Merkle, P. F. Stadler, "On the Realisability of Chemical Pathways." *LNBI* **2023**, 14348, 409.

found in the below. The analysis of the deconstruction pathways reveals a consistent mechanism in the construction of the reduced compounds in the molecular pairs in Fig. 4.

Overview of the Reaction Rules

The reaction rules are modeled as double pushout (DPO) graph transformation rules (see Figure 1 for an example) encoding the necessary bond and atom patterns that must be present before the reaction (L), as well as the bond and atom patterns introduced by the reaction (R).

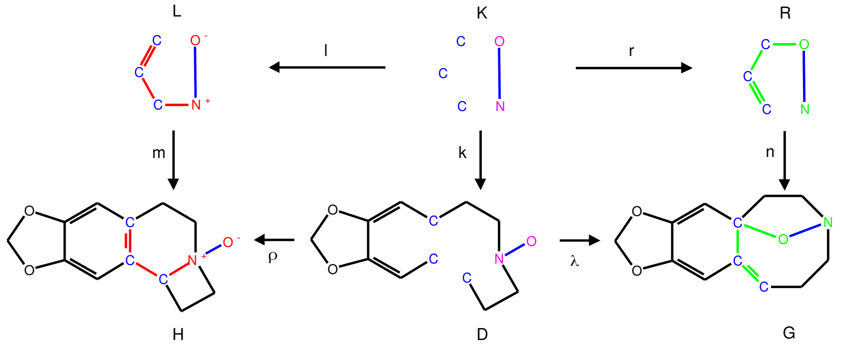

Figure 1: **Double pushout (DPO) rule of the Meisenheimer rearrangement.** The top row shows the graph transformation rule, specified in 3 graphs the left graph L, encoding the bond and atom patterns, that must be present before the reaction (highlighted in red), the context graph K (highlighted in blue), capturing the constant part of the reaction center, and the right graph R, specifying the bond and atom pattern present after the reaction (highlighted in green). The bottom row shows the application of the rewrite rule to an educt molecule H, yielding the product molecule G. The mechanism of rewriting first finds L in H, followed by erasing the read high lighted parts of L from H producing the virtual intermediate graph D, and finally inserting the green highlighted parts from R into D to yield G.

On the following pages the 37 graph transformation rules which were used for the computational deconstruction of the molecules are listed as DPO rules. The atom label '*' matches any atom in the periodic table. Atom labels which are uppercase letters prefixed with an underscore, e.g. _X, match only a subset of the atoms of the periodic table. The set of atoms which is matched by these lables is specified below the DPO rule. The numbers in the angular brackets specify the atom to atom map. The layout of the DPO rules was generated automatically and is therefor not always intersection free.

0.1 DPO Rule(s)

0.1.1 n20: (N#C)2CS* + NH3-> (N#C)2CNH2 + HS*

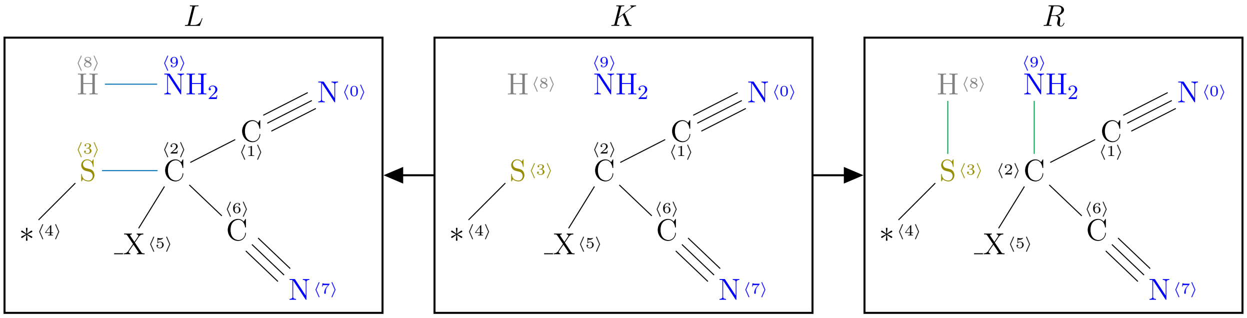

Files: out/001_r_29_11300110_{L, K, R}

_X ∈ {'H', 'C'}

0.1.2 20b: N#CC(*)(*)S* + NH3 -> N#CC(*)(*)NH2 + HS*

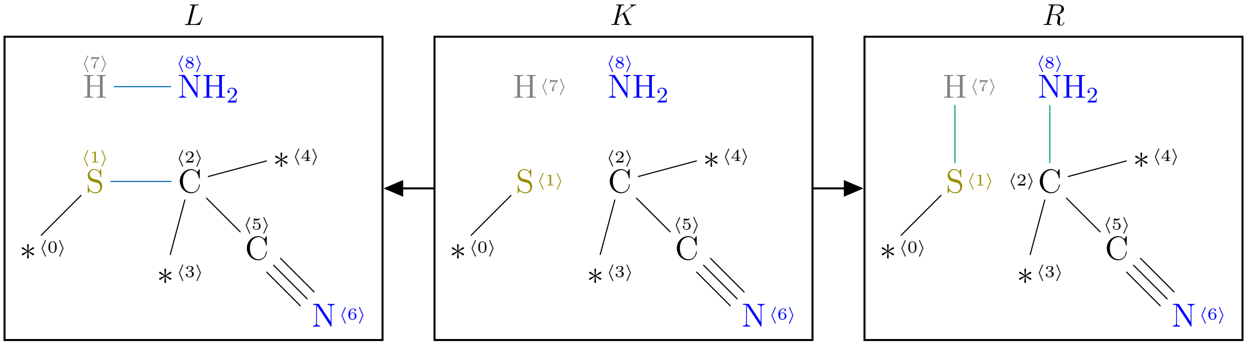

Files: out/004_r_30_11300110_{L, K, R}

0.1.3 n01: break 5-cyc with N=C

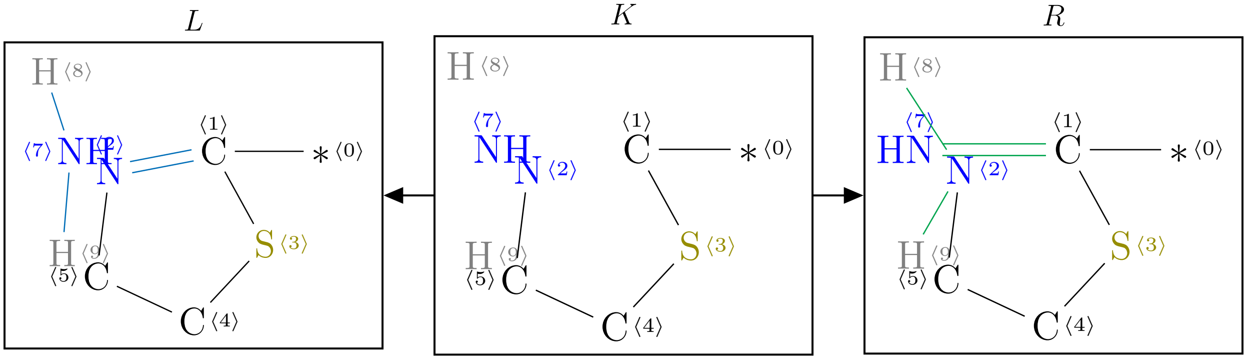

Files: out/007_r_0_11300110_{L, K, R}

0.1.4 n01y: break 5-cyc of pyrrole context

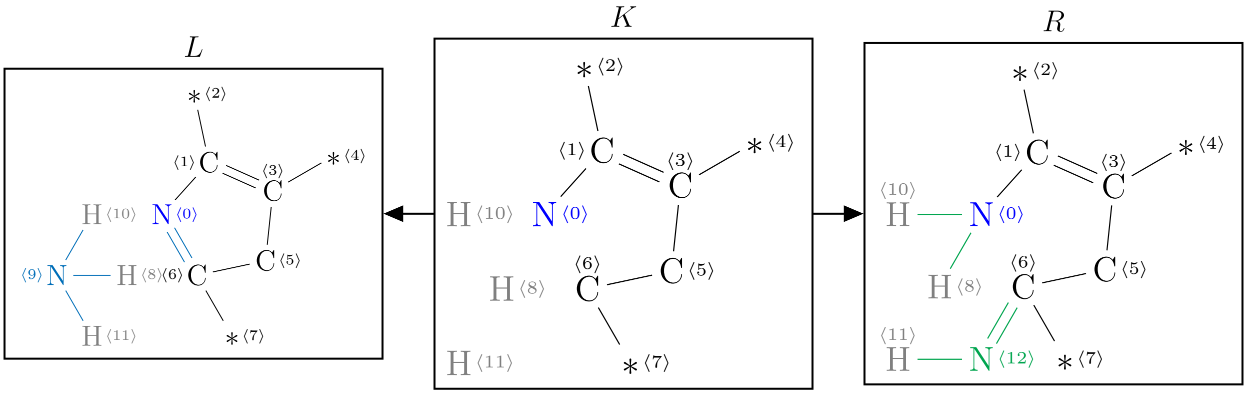

Files: out/010_r_1_11300110_{L, K, R}

0.1.5 n01z: break 5-cyc with HN-C-NH2 context

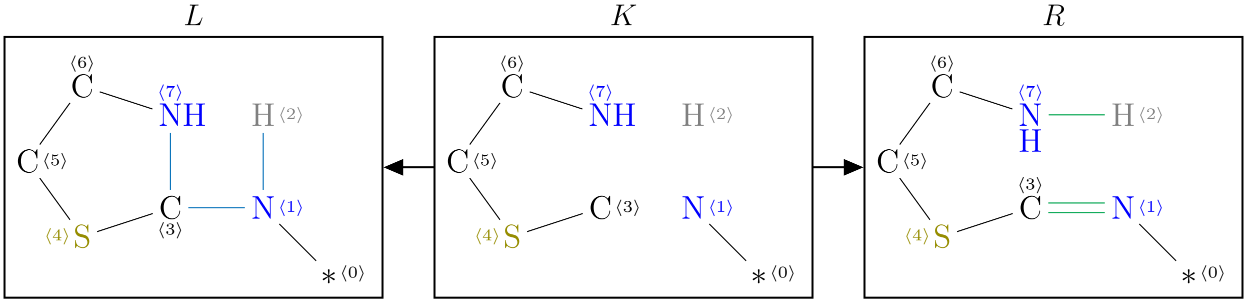

Files: out/013_r_2_11300110_{L, K, R}

0.1.6 n01a: break 5-cyc with HN-C

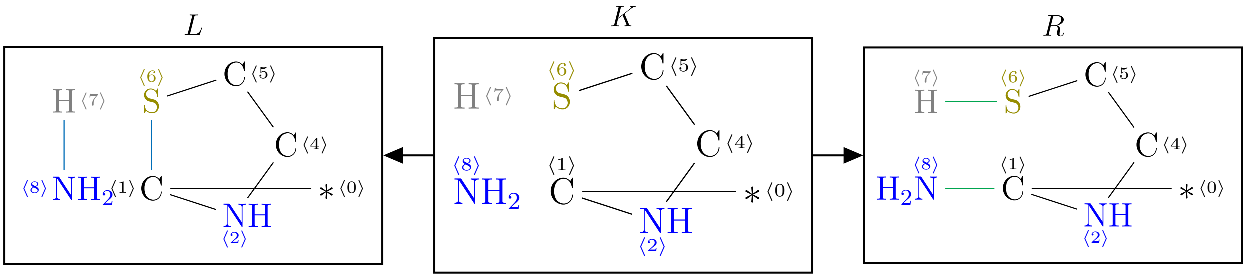

Files: out/016_r_3_11300110_{L, K, R}

0.1.7 n01b: break 5-cyc with exo C=N

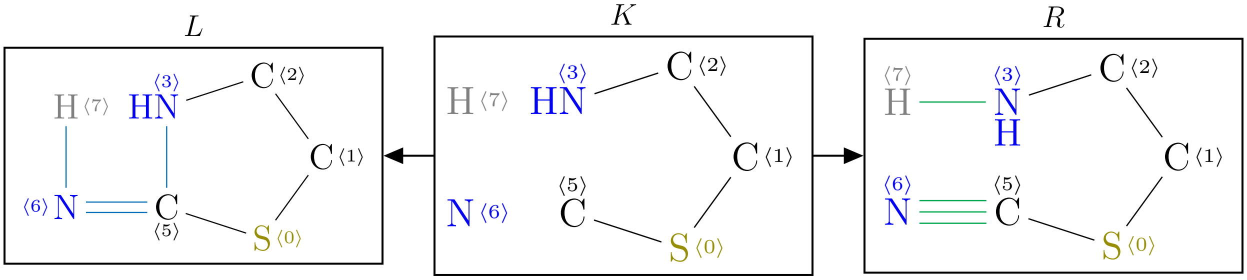

Files: out/019_r_4_11300110_{L, K, R}

0.1.8 n01c: break 6-cyc with exo C=N

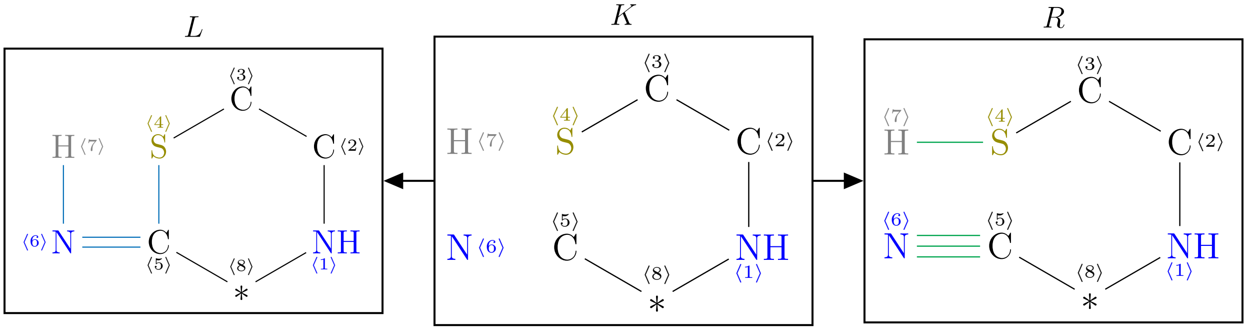

Files: out/022_r_5_11300110_{L, K, R}

0.1.9 n01d: break 6-cyc with exo C=N + C=N in cycle

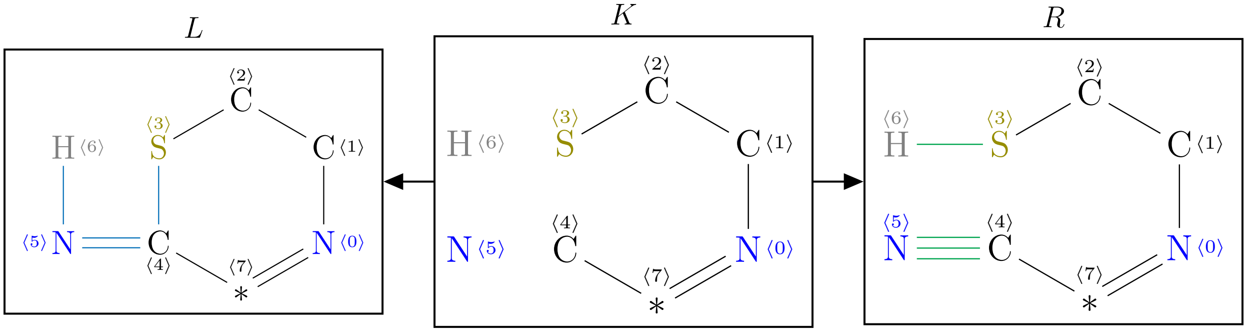

Files: out/025_r_6_11300110_{L, K, R}

0.1.10 n01e: break 6-cyc with exo C=N + C=N in cycle

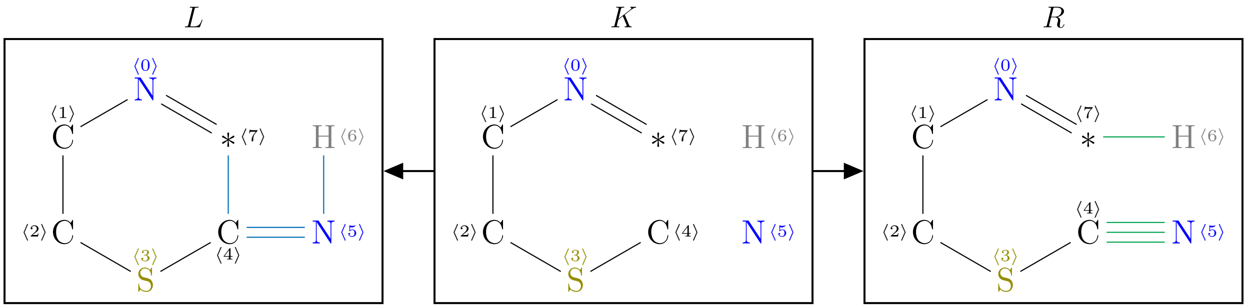

Files: out/028_r_7_11300110_{L, K, R}

0.1.11 n01f: break 6-cyc with C=N in cycle 1/3

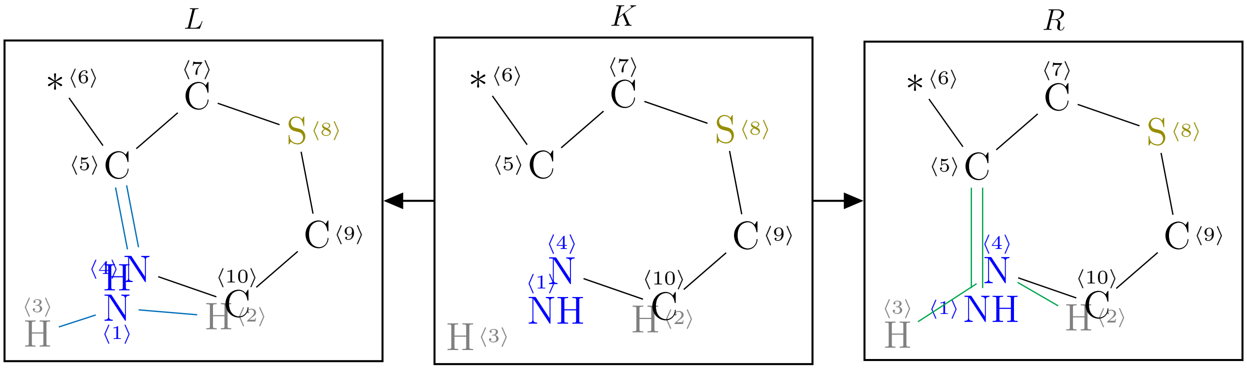

Files: out/031_r_8_11300110_{L, K, R}

0.1.12 n01ff: breacing 6-cyc with C=N in cycle 2/3

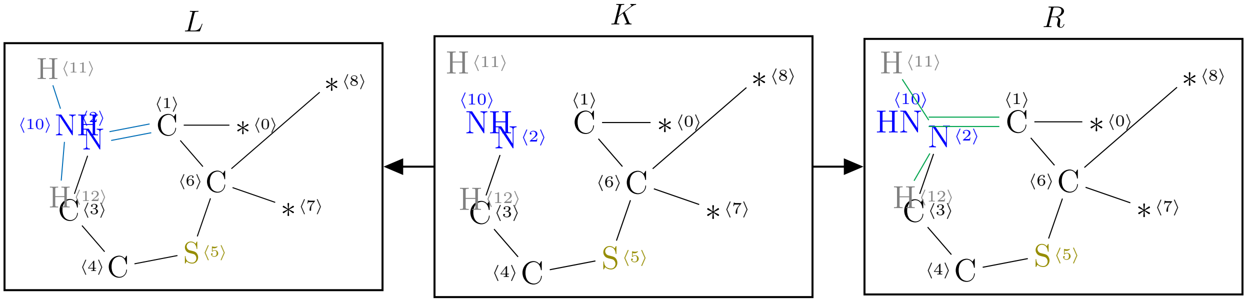

Files: out/034_r_9_11300110_{L, K, R}

0.1.13 n01g: *-SC(=NH)* -> SH + N#C-*

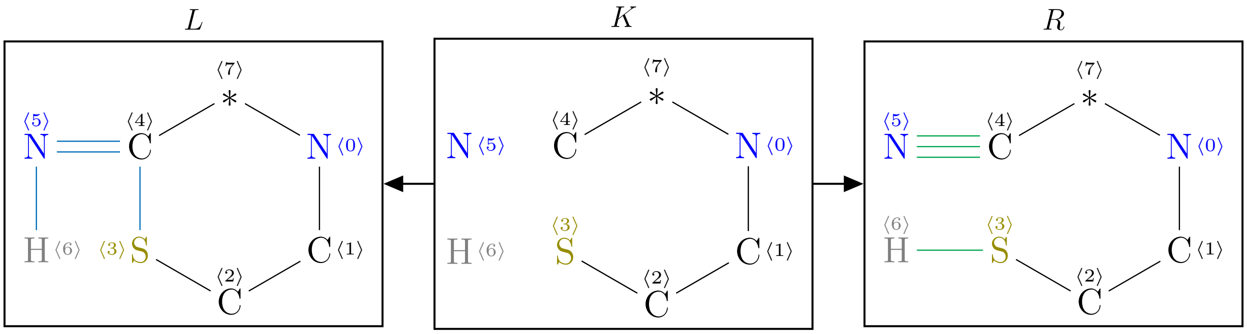

Files: out/037_r_10_11300110_{L, K, R}

0.1.14 n02: C=NH -> C=O + NH3

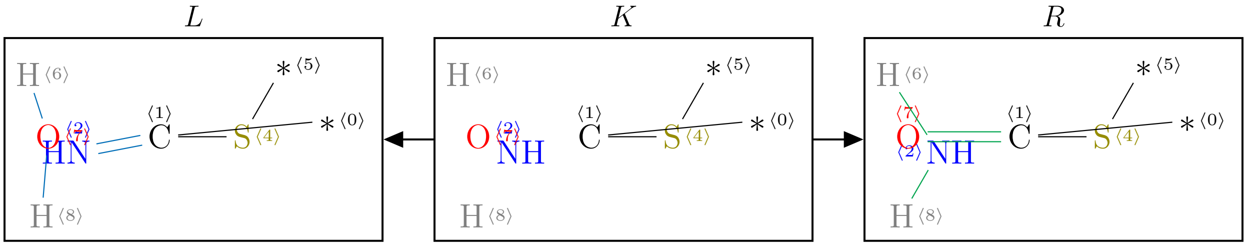

Files: out/040_r_11_11300110_{L, K, R}

0.1.15 n02rev: C=O + NH3 -> C=NH + H2O

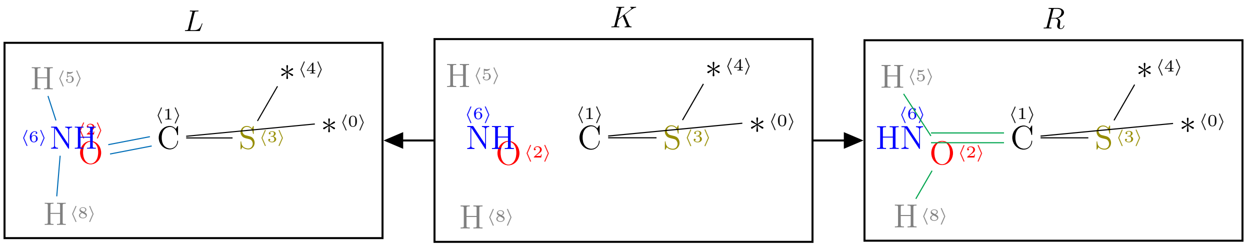

Files: out/043_r_12_11300110_{L, K, R}

0.1.16 n03: remove cys *N(H)-C(H)-NH2 context

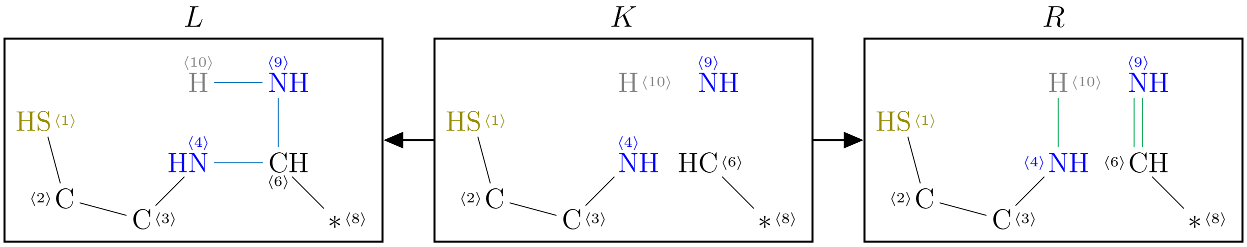

Files: out/046_r_13_11300110_{L, K, R}

0.1.17 n03a: remove cys from *S-C(=NH)* context

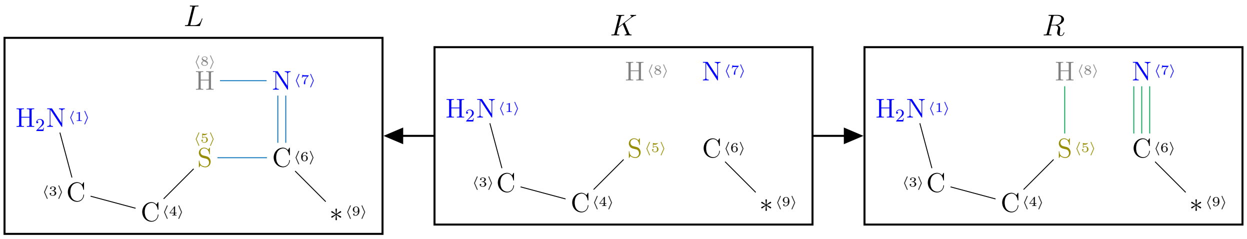

Files: out/049_r_14_11300110_{L, K, R}

0.1.18 n03b: remove cys from *NH-C(=NH)* context

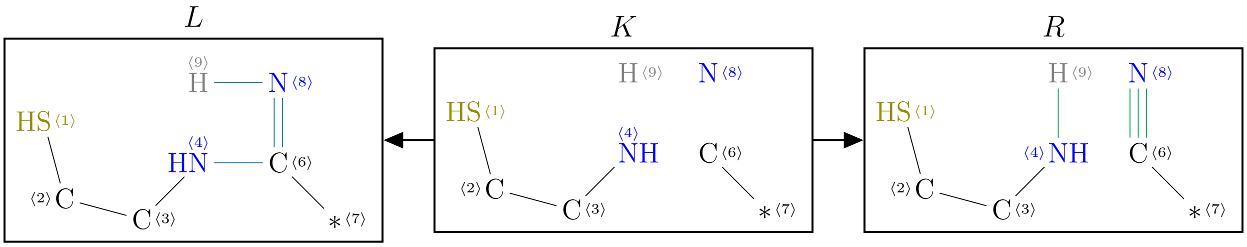

Files: out/052_r_15_11300110_{L, K, R}

0.1.19 n03c: remove cys from SCCN=C via NH3

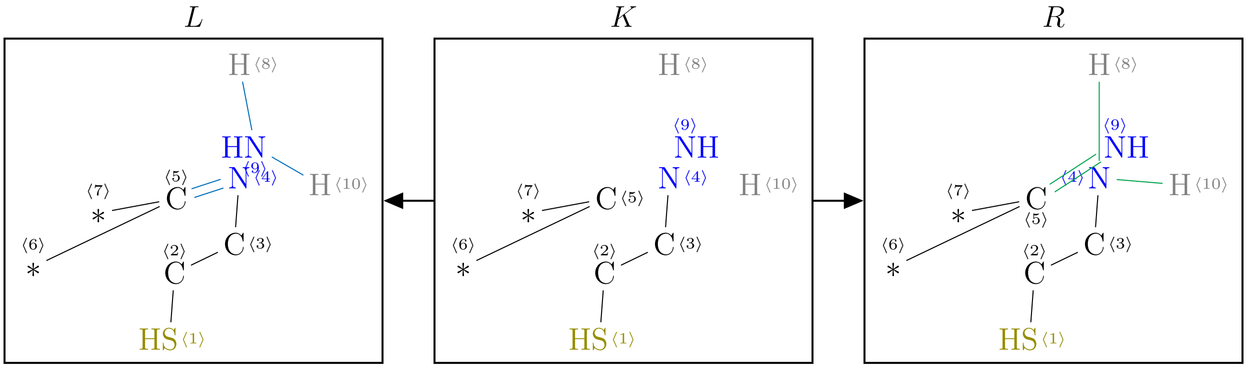

Files: out/055_r_16_11300110_{L, K, R}

0.1.20 n03d: removinc cys from (HCN)3 via NH2 substitution

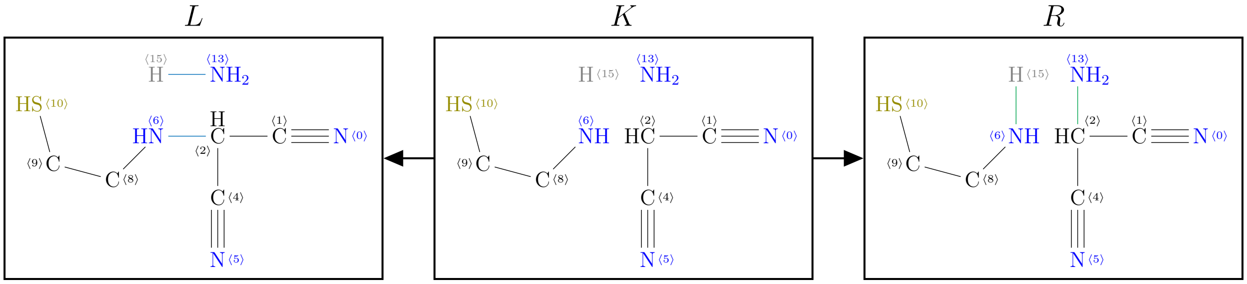

Files: out/058_r_17_11300110_{L, K, R}

0.1.21 n04: N#CC(=NH)[H,C,S] -> HCN + [H,C,S]CN

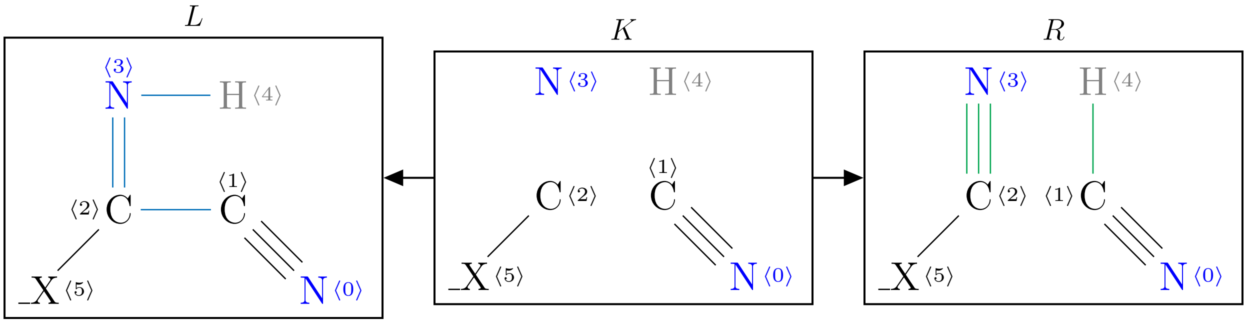

Files: out/061_r_19_11300110_{L, K, R}

_X ∈ {'H', 'S', 'C'}

0.1.22 n05: N#C-CYS + CYS -> HCN + (CYS)2

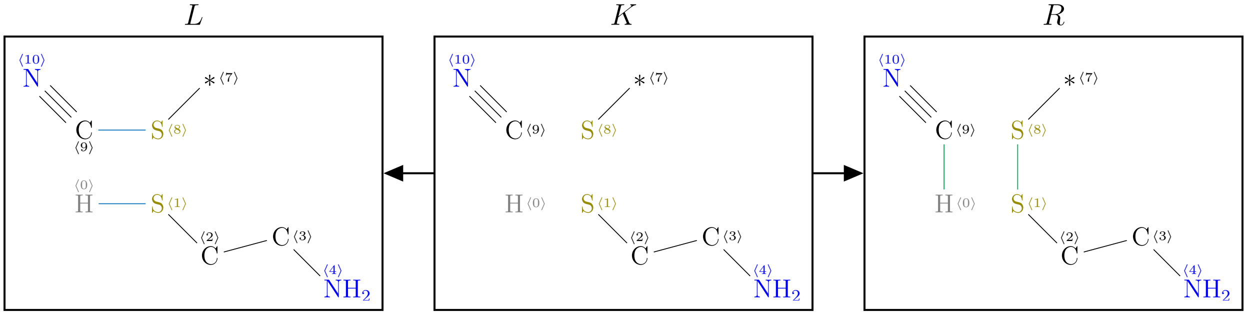

Files: out/064_r_20_11300110_{L, K, R}

0.1.23 n06: *-S-S-* + H2 -> 2 *-S-H

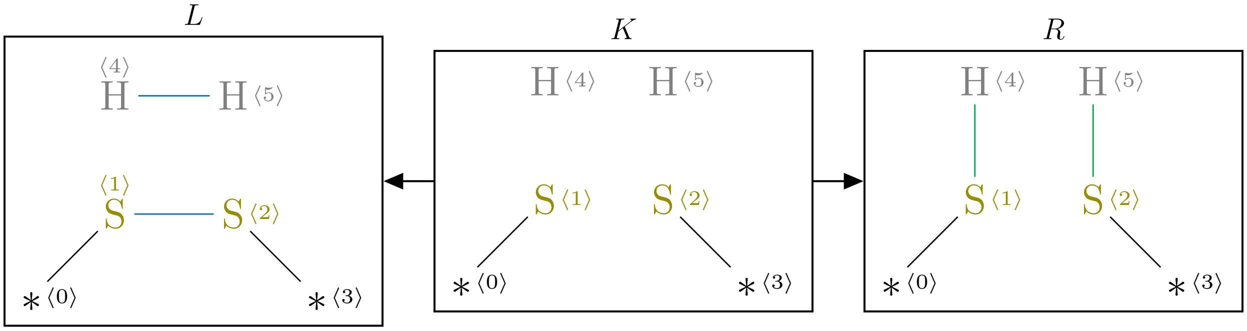

Files: out/067_r_21_11300110_{L, K, R}

0.1.24 n07: (HCN)3 -> HCN + (HCN)2

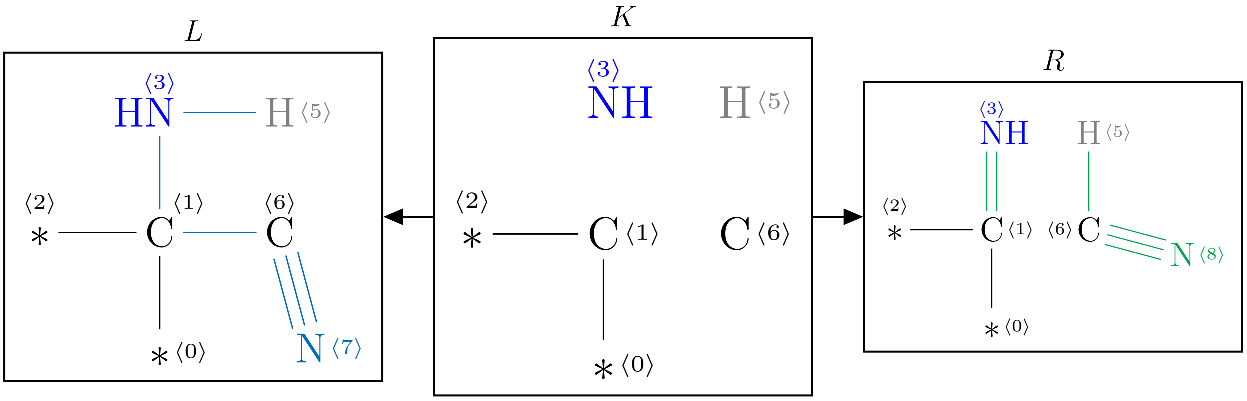

Files: out/070_r_22_11300110_{L, K, R}

0.1.25 n08: (HCN)4 tautomerism

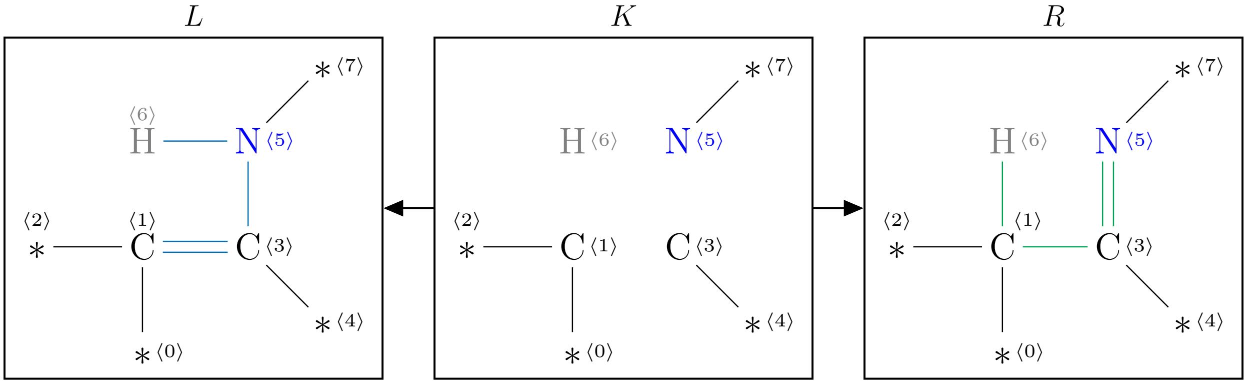

Files: out/073_r_23_11300110_{L, K, R}

0.1.26 n08b: (HCN)4 tautomerism

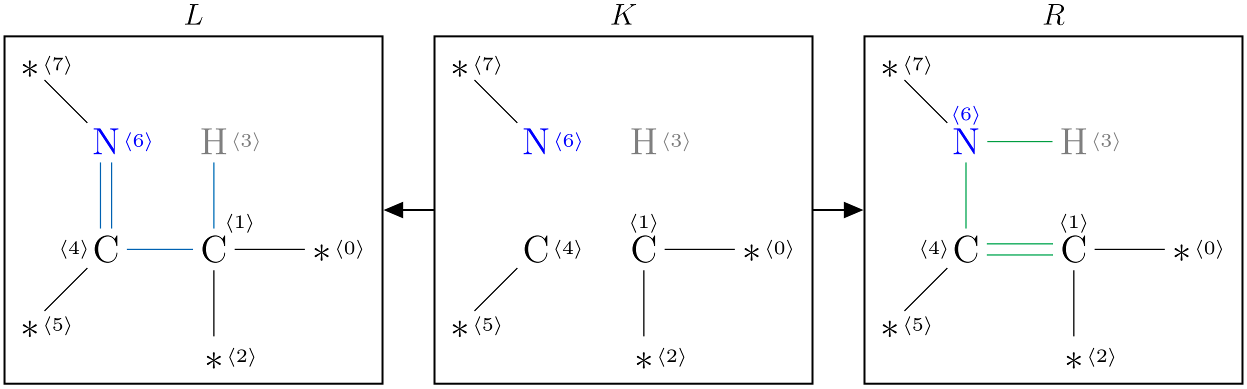

Files: out/076_r_24_11300110_{L, K, R}

0.1.27 n08c: N#C-CH2-NH2 + CYS -> N#C-CYS + NH3

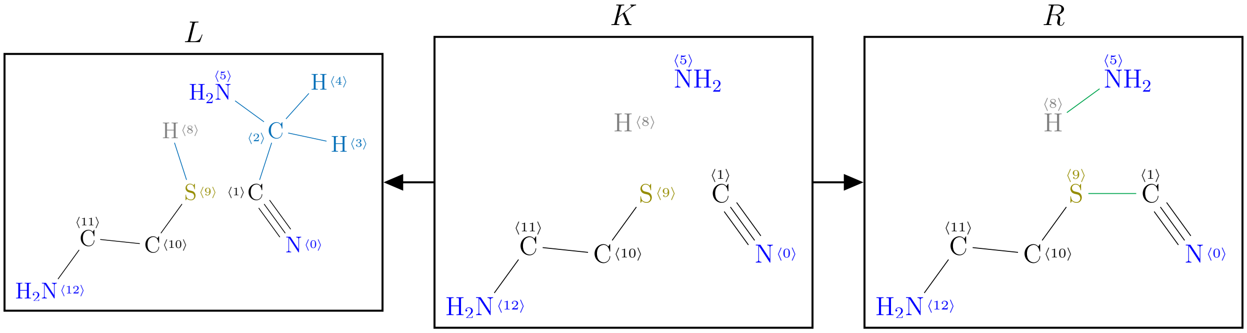

Files: out/079_r_25_11300110_{L, K, R}

0.1.28 n09: amino imino tauromerism

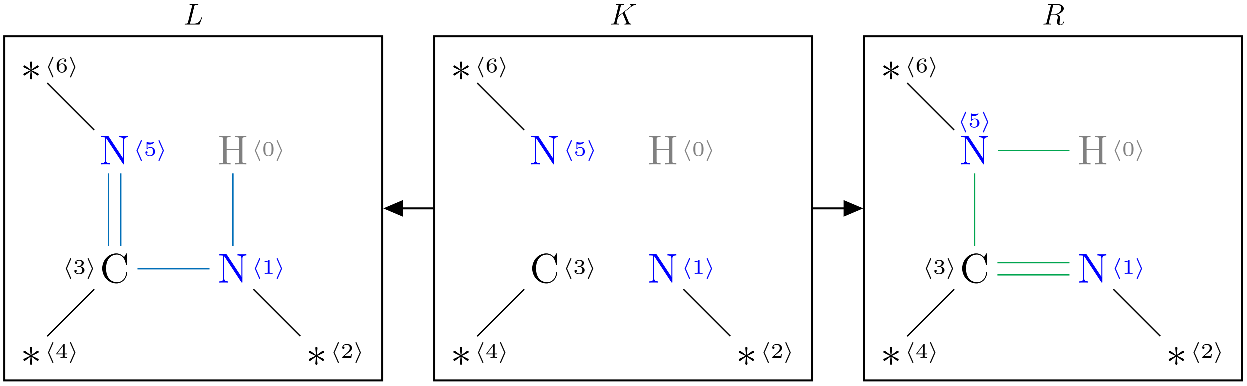

Files: out/082_r_26_11300110_{L, K, R}

0.1.29 n09b: amino imino tauromerism

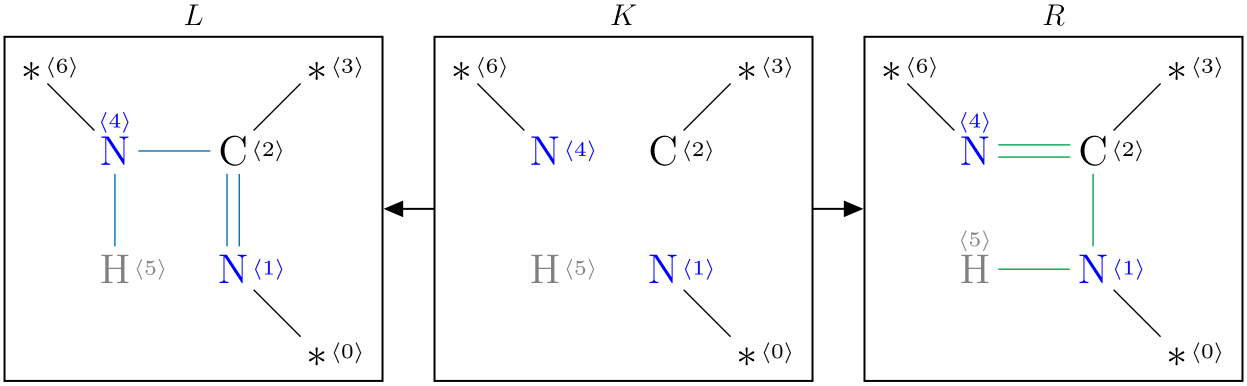

Files: out/085_r_27_11300110_{L, K, R}

0.1.30 n30: *-CONH2 -> *-C#N + H2O

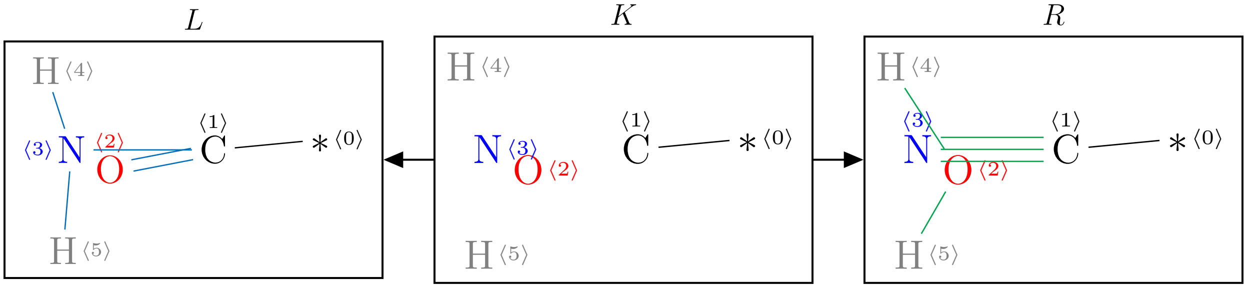

Files: out/088_r_31_11300110_{L, K, R}

0.1.31 n30: *-C#N + H2O -> *-CONH2

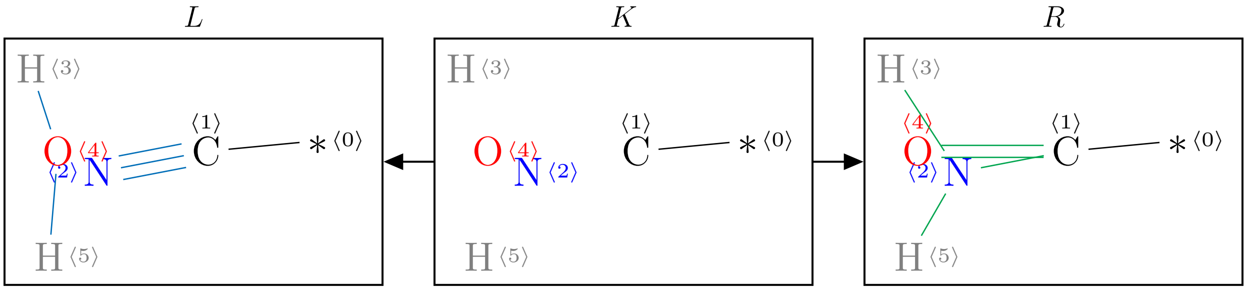

Files: out/091_r_32_11300110_{L, K, R}

0.1.32 n31: *-C(=O)NH2 + H2O -> *-CO2H + NH3

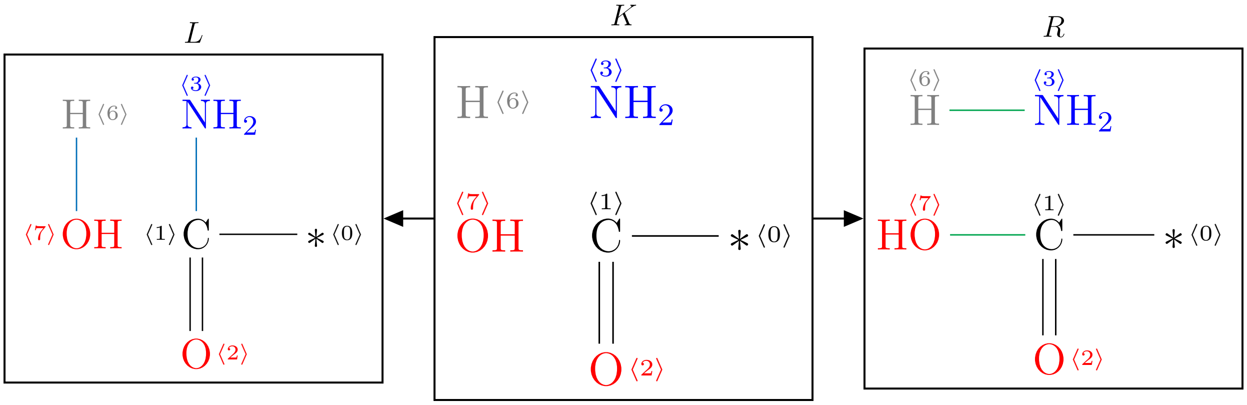

Files: out/094_r_33_11300110_{L, K, R}

0.1.33 n31: *-C(=O)NH2 + H2O -> *-CO2H + NH3

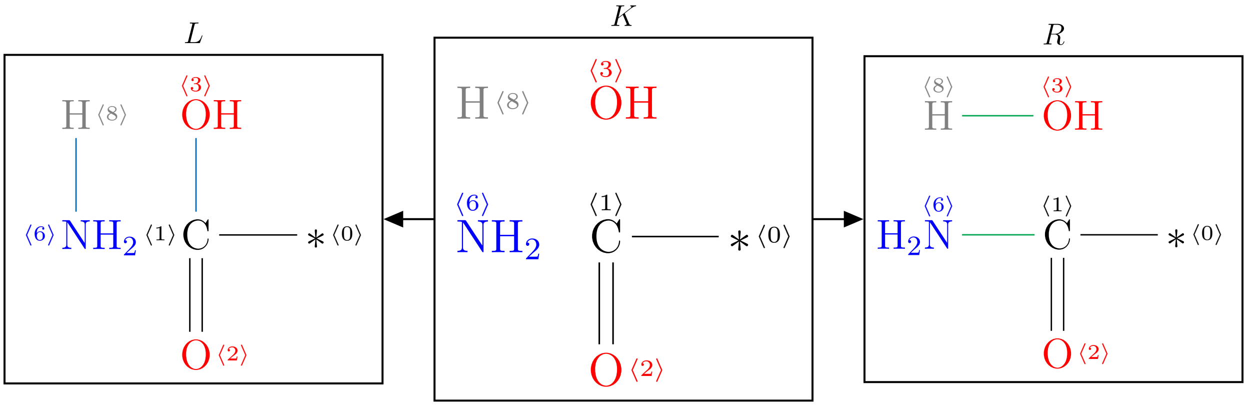

Files: out/097_r_34_11300110_{L, K, R}

0.1.34 n32: *-CO2H + NH3 -> *-C#N + 2 H2O

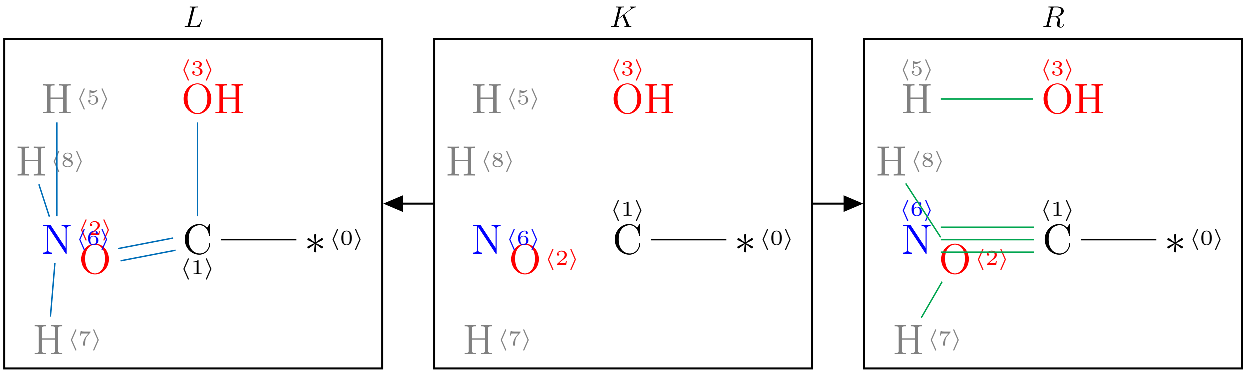

Files: out/100_r_35_11300110_{L, K, R}

0.1.35 n40: SC(H)(CO2H)N -> SC(H2)N + CO2

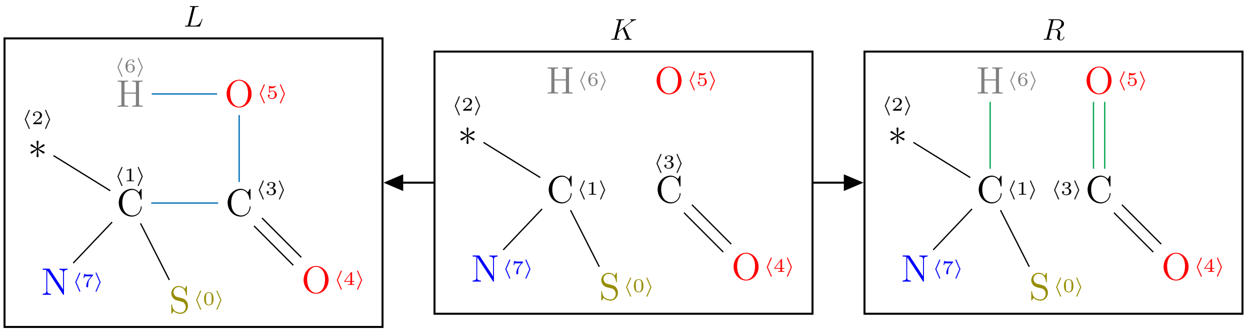

Files: out/103_r_36_11300110_{L, K, R}

0.1.36 n40rev: SC(H2)N + CO2 -> SC(H)(CO2H)N

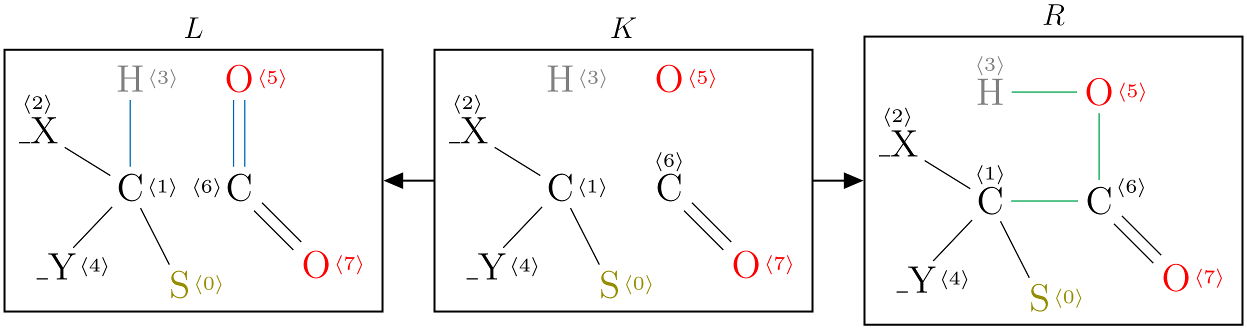

Files: out/106_r_38_11300110_{L, K, R}

_X ∈ {'C', 'N'}
_Y ∈ {'C', 'N'}

0.1.37 n50: split off cys

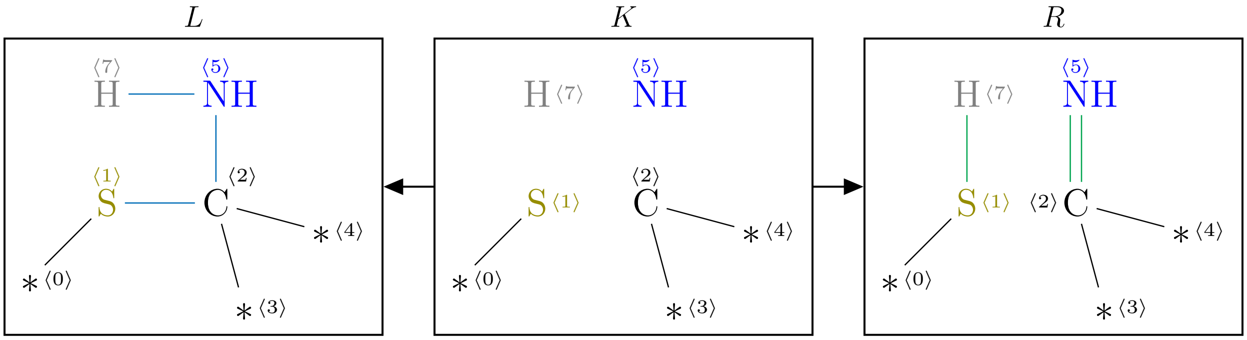

Files: out/109_r_40_11300110_{L, K, R}

9. Appendix S1: Raw NMR integral data for kinetic experiments

This appendix provides the raw ¹H NMR integral data used for the kinetic analysis described in Section 3 (NMR Experiments) of the Supporting Information and for constructing the kinetic plots presented in the main manuscript

(ppm)	1	2	3	4	5	6	7	8	9	10	11	12	13	14	15	16	17	18	19	20	21	22
[1.65 .. 1.92]	2.65E-03	3.18E-03	3.57E-03	4.26E-03	4.20E-03	4.08E-03	4.16E-03	4.36E-03	3.84E-03	3.73E-03	3.37E-03	2.62E-03	2.58E-03	2.82E-03	2.96E-03	2.83E-03	2.92E-03	2.97E-03	2.89E-03	2.96E-03	2.94E-03	3.15E-03
[3.01 .. 3.14]	2.45E-01	2.35E-01	2.34E-01	2.28E-01	2.18E-01	2.07E-01	1.85E-01	1.62E-01	1.29E-01	9.69E-02	8.21E-02	6.95E-02	5.97E-02	5.31E-02	4.83E-02	4.51E-02	4.26E-02	4.05E-02	3.91E-02	3.84E-02	3.74E-02	3.64E-02
[3.39 .. 3.51]	2.51E-01	2.42E-01	2.42E-01	2.36E-01	2.29E-01	2.19E-01	2.00E-01	1.76E-01	1.43E-01	1.11E-01	8.95E-02	7.38E-02	6.38E-02	5.80E-02	5.42E-02	5.15E-02	4.98E-02	4.85E-02	4.74E-02	4.68E-02	4.64E-02	4.61E-02
[4.50 .. 5.09]	4.42E-01	4.25E-01	4.20E-01	4.13E-01	4.19E-01	4.31E-01	4.30E-01	4.31E-01	4.29E-01	4.24E-01	4.72E-01	5.05E-01	5.26E-01	5.41E-01	5.53E-01	5.65E-01	5.74E-01	5.81E-01	5.87E-01	5.92E-01	5.95E-01	5.95E-01

Table S1.1. Integral data for the standard addition experiment: 2M HCN + 2M MEA*HCl at 40°C, no seeds. Spectra recorded at 10-minute intervals. Cysteamine triplets (δ 3.01-3.14 ppm) and sodium acetate internal standard (δ 1.65-1.92 ppm) and water as additional internal standard (δ 4.50-5.09 ppm) integrated. *Corresponds to SI Fig. 3.1 and Main Text Fig. 1B (red triangles).*

(ppm)	1	2	3	4	5	6	7	8	9	10	11	12	13	14	15	16	17	18	19	20	21	22
[1.66 .. 1.89]	3.14E-03	4.68E-03	4.60E-03	4.69E-03	4.53E-03	4.36E-03	4.14E-03	3.84E-03	3.70E-03	3.61E-03	3.53E-03	3.49E-03	3.48E-03	3.49E-03	3.57E-03	3.61E-03	3.63E-03	3.68E-03	3.69E-03	3.73E-03	3.70E-03	3.72E-03
[3.03 .. 3.13]	3.36E-01	2.15E-01	2.06E-01	1.98E-01	1.80E-01	1.54E-01	1.22E-01	8.92E-02	7.09E-02	5.66E-02	4.49E-02	3.70E-02	3.24E-02	2.95E-02	2.77E-02	2.60E-02	2.50E-02	2.41E-02	2.30E-02	2.17E-02	2.14E-02	2.10E-02
[3.40 .. 3.50]	3.40E-01	2.17E-01	2.09E-01	2.04E-01	1.88E-01	1.61E-01	1.27E-01	9.51E-02	7.26E-02	5.73E-02	4.71E-02	4.20E-02	4.09E-02	4.09E-02	4.06E-02	3.99E-02	4.03E-02	3.92E-02	3.83E-02	3.72E-02	3.71E-02	3.69E-02

Table S1.2. Integral data for the standard addition experiment: 2M HCN + 2M MEA*HCl at 40°C, with 5% seeds. Spectra recorded at 10-minute intervals. Cysteamine triplets (δ 3.03-3.13 ppm) and sodium acetate internal standard (δ 1.66-1.89 ppm) integrated. *Corresponds to SI Fig. 3.1 and Main Text Fig. 1B (blue circles).*

(ppm)	1	2	3	4	5	6	7	8	9	10	11	12	13	14	15	16	17	18	19	20
[1.31 .. 1.35]	2.11E-02	2.91E-02	2.86E-02	2.87E-02	2.87E-02	2.85E-02	2.81E-02	2.89E-02	2.83E-02	2.74E-02	2.76E-02	2.80E-02	2.67E-02	2.63E-02	2.60E-02	2.54E-02	2.48E-02	2.42E-02	2.25E-02	2.24E-02
[2.92 .. 2.97]	1.30E-01	1.81E-01	1.77E-01	1.74E-01	1.69E-01	1.64E-01	1.57E-01	1.52E-01	1.44E-01	1.34E-01	1.25E-01	1.16E-01	1.03E-01	9.21E-02	8.21E-02	7.15E-02	6.19E-02	5.34E-02	4.43E-02	3.76E-02
[3.29 .. 3.34]	1.33E-01	1.84E-01	1.80E-01	1.75E-01	1.71E-01	1.65E-01	1.58E-01	1.50E-01	1.43E-01	1.32E-01	1.22E-01	1.10E-01	9.75E-02	8.54E-02	7.38E-02	6.17E-02	5.11E-02	4.19E-02	3.32E-02	2.58E-02

21	22	23	24	25	26	27	28	29	30	31	32	33	34	35	36	37	38	39	40	41
2.17E-02	2.13E-02	2.06E-02	2.06E-02	2.10E-02	2.19E-02	2.26E-02	2.26E-02	1.76E-02	1.77E-02	1.74E-02	1.78E-02	1.76E-02	1.77E-02	2.22E-02	2.21E-02	2.19E-02	2.15E-02	2.15E-02	2.15E-02	2.15E-02
3.20E-02	3.00E-02	3.36E-02	3.22E-02	2.73E-02	2.31E-02	1.99E-02	1.75E-02	1.31E-02	1.20E-02	1.15E-02	1.12E-02	1.07E-02	1.02E-02	1.22E-02	1.18E-02	1.14E-02	1.11E-02	1.09E-02	1.06E-02	1.04E-02
2.08E-02	1.95E-02	2.28E-02	2.14E-02	1.70E-02	1.35E-02	1.15E-02	1.09E-02	8.34E-03	8.05E-03	7.86E-03	8.06E-03	7.96E-03	8.01E-03	1.05E-02	1.07E-02	1.07E-02	1.08E-02	1.08E-02	1.09E-02	1.10E-02

Table S1.3. Integral data for the standard addition experiment: 2M HCN + 2M MEA*HCl at 40°C, with 10% seeds. Spectra recorded at 5-minute intervals. Cysteamine triplets (δ 2.92-2.97 ppm) and t-BuOH internal standard (δ 1.31-1.35ppm) integrated. *Corresponds to SI Fig. 3.3 and Main Text Fig. 1B (black crosses).*

(ppm)	1	2	3	4	5	6	7	8	9	10	11	12	13	14	15	16	17	18	19	20	21
[2.97 .. 3.05]	1.91E-03	1.89E-03	1.91E-03	1.95E-03	1.99E-03	1.99E-03	1.95E-03	1.85E-03	1.70E-03	1.51E-03	1.27E-03	9.76E-04	6.97E-04	4.82E-04	2.82E-04	1.75E-04	1.37E-04	1.17E-04	1.29E-04	1.38E-04	1.37E-04
[3.34 .. 3.41]	1.92E-03	1.90E-03	1.92E-03	1.96E-03	2.01E-03	2.02E-03	1.99E-03	1.89E-03	1.74E-03	1.55E-03	1.28E-03	9.66E-04	6.80E-04	4.34E-04	2.49E-04	1.81E-04	1.75E-04	1.67E-04	1.71E-04	2.05E-04	2.01E-04
[4.54 .. 4.90]	4.76E-03	4.58E-03	4.47E-03	4.40E-03	4.46E-03	4.48E-03	4.59E-03	4.70E-03	4.82E-03	4.98E-03	5.00E-03	5.08E-03	5.35E-03	5.80E-03	6.16E-03	6.43E-03	6.70E-03	6.92E-03	6.97E-03	6.82E-03	6.68E-03
[7.52 .. 7.58]	1.32E-06	2.18E-06	2.50E-06	1.30E-06	6.79E-07	4.90E-07	1.75E-07	3.53E-07	4.91E-08	1.06E-07	3.62E-06	9.72E-06	8.40E-06	3.15E-06	2.74E-06	2.78E-06	2.97E-06	2.05E-06	1.33E-06	4.98E-06	4.21E-06
[7.59 .. 7.62]	2.19E-05	2.20E-05	2.27E-05	2.40E-05	2.55E-05	2.68E-05	2.74E-05	2.80E-05	2.87E-05	2.91E-05	2.80E-05	2.06E-05	1.40E-05	9.82E-06	7.01E-06	5.33E-06	4.60E-06	4.23E-06	4.68E-06	8.17E-06	7.69E-06

Table S1.4. Integral data for the NMR experiment with benzene: 2M HCN + 2M MEA*HCl with benzene at 40°C. Spectra recorded at 10-minute intervals. Cysteamine triplets (δ 2.97-3.5 ppm), benzene signal S1 (δ 7.59-7.62 ppm) and benzene signal S2 (δ 7.52-7.58 ppm) integrated. As internal standard water signal at (δ 4.54-4.90 ppm) was used. Initial benzene concentration calculated relative to cysteamine. *Corresponds to SI Fig. 3.4, 3.5 and Main Text Fig. 5A, B.*

(ppm)	1	2	3	4	5	6	7	8	9	10	11	12	13	14	15	16	17	18	19	20
[0.79 .. 0.83]	6.45E-03	4.11E-03	3.12E-03	2.64E-03	2.31E-03	2.01E-03	1.86E-03	1.58E-03	1.40E-03	1.24E-03	1.18E-03	1.08E-03	9.73E-04	8.72E-04	8.47E-04	7.66E-04	6.43E-04	4.02E-04	1.01E-05	3.03E-04
[1.37 .. 1.42]	5.80E-02	5.84E-02	5.88E-02	5.92E-02	6.06E-02	6.12E-02	6.13E-02	6.19E-02	6.19E-02	6.13E-02	6.09E-02	6.06E-02	6.06E-02	6.02E-02	6.00E-02	5.97E-02	5.86E-02	5.86E-02	5.82E-02	5.78E-02
[2.96 .. 3.07]	1.93E-01	1.98E-01	2.01E-01	2.03E-01	2.08E-01	2.11E-01	2.11E-01	2.15E-01	2.15E-01	2.12E-01	2.09E-01	2.06E-01	2.04E-01	2.00E-01	1.94E-01	1.86E-01	1.75E-01	1.65E-01	1.50E-01	1.31E-01
[3.34 .. 3.42]	1.94E-01	2.00E-01	2.04E-01	2.07E-01	2.12E-01	2.15E-01	2.15E-01	2.18E-01	2.18E-01	2.15E-01	2.11E-01	2.09E-01	2.06E-01	2.03E-01	1.97E-01	1.89E-01	1.79E-01	1.68E-01	1.52E-01	1.31E-01

(ppm)	21	22	23	24	25	26	27	28	29	30	31	32	33	34	35	36	37	38	39	40
[0.79 .. 0.83]	2.37E-04	5.38E-05	6.80E-05	1.59E-05	0.00E+00	0.00E+00	0.00E+00	8.14E-05	0.00E+00	0.00E+00	0.00E+00	1.34E-05	0.00E+00	0.00E+00	0.00E+00	0.00E+00	0.00E+00	0.00E+00	0.00E+00	0.00E+00
[1.37 .. 1.42]	5.60E-02	5.46E-02	5.25E-02	5.32E-02	5.42E-02	5.46E-02	5.39E-02	5.33E-02	5.41E-02	5.55E-02	5.49E-02	5.51E-02	5.61E-02	5.64E-02	5.68E-02	5.69E-02	5.72E-02	5.74E-02	5.74E-02	5.75E-02
[2.96 .. 3.07]	1.07E-01	8.45E-02	6.54E-02	5.27E-02	4.06E-02	3.22E-02	2.67E-02	2.48E-02	2.16E-02	2.00E-02	1.94E-02	1.88E-02	1.77E-02	1.72E-02	1.68E-02	1.64E-02	1.61E-02	1.57E-02	1.56E-02	1.53E-02
[3.34 .. 3.42]	1.06E-01	8.21E-02	6.25E-02	4.98E-02	3.88E-02	3.17E-02	2.93E-02	2.90E-02	2.80E-02	2.73E-02	2.70E-02	2.66E-02	2.63E-02	2.60E-02	2.57E-02	2.55E-02	2.53E-02	2.50E-02	2.49E-02	2.47E-02

Table S1.5. Integral data for the NMR experiment with Triton X-100: 2M HCN + 2M MEAHCl with Triton X-100 (15 mM) at 40°C. Spectra recorded at 10-minute intervals. Cysteamine triplets (δ 2.96-3.07 ppm), Triton X-100 signal (δ 0.79-0.83 ppm), and t-BuOH internal standard (δ 1.37-1.42 ppm) integrated. *Corresponds to SI Fig. 3.6 and Main Text Fig. 2C.*

(ppm)	1	2	3	4	5	6	7	8	9	10	11	12	13	14	15	16	17	18	19	20
[1.43 .. 1.47]	6.74E-02	5.95E-02	6.06E-02	6.17E-02	6.28E-02	6.33E-02	6.29E-02	6.53E-02	6.17E-02	6.07E-02	5.62E-02	6.12E-02	6.10E-02	6.04E-02	5.98E-02	5.91E-02	5.78E-02	5.57E-02	4.73E-02	3.70E-02
[1.87 .. 1.97]	3.28E-02	2.91E-02	2.97E-02	3.02E-02	3.07E-02	3.09E-02	3.09E-02	3.24E-02	2.99E-02	2.93E-02	2.77E-02	2.93E-02	2.92E-02	2.90E-02	2.87E-02	2.83E-02	2.72E-02	2.59E-02	2.18E-02	1.63E-02
[3.04 .. 3.10]	2.20E-01	1.94E-01	1.97E-01	2.00E-01	2.04E-01	2.06E-01	2.05E-01	2.14E-01	2.01E-01	1.96E-01	1.82E-01	1.93E-01	1.89E-01	1.81E-01	1.70E-01	1.55E-01	1.33E-01	1.06E-01	7.70E-02	5.48E-02
[3.40 .. 3.47]	2.31E-01	2.04E-01	2.08E-01	2.11E-01	2.15E-01	2.17E-01	2.15E-01	2.21E-01	2.09E-01	2.05E-01	1.86E-01	2.02E-01	1.97E-01	1.89E-01	1.78E-01	1.62E-01	1.39E-01	1.10E-01	7.90E-02	5.53E-02

21	22	23	24	25	26	27	28	29	30	31	32	33
4.98E-02	5.05E-02	5.02E-02	5.12E-02	5.22E-02	5.28E-02	5.33E-02	5.39E-02	5.38E-02	5.40E-02	5.41E-02	5.44E-02	6.17E-02
1.95E-02	1.89E-02	1.80E-02	1.76E-02	1.75E-02	1.73E-02	1.71E-02	1.70E-02	1.67E-02	1.65E-02	1.64E-02	1.63E-02	1.84E-02
5.22E-02	4.44E-02	3.94E-02	3.61E-02	3.33E-02	3.16E-02	3.02E-02	2.88E-02	2.79E-02	2.71E-02	2.63E-02	2.55E-02	2.84E-02
4.76E-02	4.01E-02	3.57E-02	3.32E-02	3.15E-02	3.08E-02	3.02E-02	2.95E-02	2.91E-02	2.86E-02	2.81E-02	2.76E-02	3.03E-02

Table S1.6. Integral data for the NMR experiment with isopentenyl alcohol: 2M HCN + 2M MEA*HCl with isopentenyl alcohol (100 mM) at 40°C. Spectra recorded at 10-minute intervals. Cysteamine triplets (δ 3.04-3.10 ppm), isopentenyl alcohol singlets (δ 1.87-1.97 ppm), and t-BuOH internal standard (δ 1.43-1.47 ppm) integrated. *Corresponds to SI Fig. 3.7 and Main Text Fig. 5C.*

(ppm)	1	2	3	4	5	6	7	8	9	10	11	12	13	14	15	16	17	18	19	20	21	22	23
[1.40 .. 1.48]	2.33E-01	2.29E-01	2.24E-01	2.24E-01	2.26E-01	2.26E-01	2.25E-01	2.24E-01	2.22E-01	2.21E-01	2.18E-01	2.13E-01	2.00E-01	1.94E-01	2.15E-01	2.18E-01	2.17E-01	2.16E-01	2.07E-01	2.19E-01	2.21E-01	2.21E-01	2.22E-01
[1.67 .. 2.10]	3.87E-02	3.46E-02	3.99E-02	3.97E-02	4.02E-02	3.94E-02	3.95E-02	3.95E-02	3.92E-02	3.91E-02	3.91E-02	3.96E-02	4.01E-02	3.66E-02	3.15E-02	3.05E-02	2.99E-02	2.93E-02	2.63E-02	2.88E-02	2.86E-02	2.84E-02	2.83E-02
[3.01 .. 3.10]	1.69E-01	1.66E-01	1.61E-01	1.59E-01	1.57E-01	1.54E-01	1.49E-01	1.41E-01	1.31E-01	1.18E-01	1.01E-01	8.00E-02	5.71E-02	4.03E-02	2.74E-02	1.71E-02	1.21E-02	1.02E-02	1.12E-02	8.22E-03	7.60E-03	7.24E-03	6.93E-03
[3.39 .. 3.46]	1.71E-01	1.67E-01	1.63E-01	1.61E-01	1.61E-01	1.57E-01	1.52E-01	1.45E-01	1.35E-01	1.22E-01	1.04E-01	8.15E-02	5.79E-02	3.99E-02	2.57E-02	1.69E-02	1.50E-02	1.51E-02	1.60E-02	1.48E-02	1.47E-02	1.48E-02	1.47E-02

Table S1.7. Integral data for the NMR experiment with cyclohexanol: 2M HCN + 2M MEA*HCl with cyclohexanol (200 mM) at 40°C. Spectra recorded at 10-minute intervals. Cysteamine triplets (δ 3.01-3.10 ppm), cyclohexanol multiplets (δ 1.67-2.10 ppm), and t-BuOH internal standard (δ 1.40-1.48 ppm) integrated. *Corresponds to SI Fig. 3.8 and Main Text Fig. 5D.*

(ppm)	1	2	3	4	5	6	7	8	9	10	11	12	13	14	15	16	17	18	19	20	21	22
[1.19 .. 1.26]	1.79E-01	1.80E-01	1.82E-01	1.83E-01	1.82E-01	1.81E-01	1.81E-01	1.79E-01	1.79E-01	1.77E-01	1.67E-01	1.62E-01	1.71E-01	1.76E-01	1.68E-01	1.71E-01	1.68E-01	1.69E-01	1.69E-01	1.90E-01	1.81E-01	1.85E-01
[2.80 .. 2.86]	1.60E-01	1.59E-01	1.54E-01	1.48E-01	1.40E-01	1.31E-01	1.20E-01	1.08E-01	9.23E-02	7.50E-02	5.48E-02	3.74E-02	2.52E-02	1.77E-02	2.68E-02	1.64E-02	1.34E-02	1.11E-02	1.03E-02	1.19E-02	1.04E-02	1.02E-02

[3.17 .. 3.22]	1.61E-01	1.61E-01	1.58E-01	1.52E-01	1.44E-01	1.35E-01	1.24E-01	1.11E-01	9.45E-02	7.55E-02	5.30E-02	3.40E-02	2.10E-02	1.40E-02	2.37E-02	1.43E-02	1.29E-02	1.23E-02	1.22E-02	1.42E-02	1.34E-02	1.40E-02
[8.14 .. 8.17]	7.58E-03	7.66E-03	7.49E-03	7.49E-03	7.49E-03	7.52E-03	7.52E-03	7.53E-03	7.54E-03	7.53E-03	7.02E-03	6.62E-03	7.13E-03	7.37E-03	6.86E-03	6.82E-03	6.44E-03	6.35E-03	6.29E-03	6.78E-03	6.54E-03	6.42E-03

Table S1.8. Integral data for the NMR experiment with adenine: 2M HCN + 2M MEA*HCl with adenine (~0.1 M) at 40°C. Spectra recorded at 10-minute intervals. Cysteamine triplets (δ 2.80-2.86 ppm), adenine signal (δ 8.14-8.17 ppm), and t-BuOH internal standard (δ 1.19-1.26 ppm) integrated. *Corresponds to SI Fig. 3.9 and SI Fig. 3.10 (kinetic plot for adenine).*

(ppm)	1	2	3	4	5	6	7	8	9	10	11	12	13	14	15	16	17	18	19	20	21
[1.18 .. 1.26]	4.31E-02	5.00E-02	6.34E-02	4.22E-02	4.16E-02	4.16E-02	4.11E-02	4.07E-02	4.01E-02	4.00E-02	3.99E-02	3.99E-02	3.99E-02	3.92E-02	3.93E-02	3.91E-02	3.90E-02	3.99E-02	3.98E-02	3.97E-02	3.98E-02
[2.78 .. 2.87]	1.68E-01	1.80E-01	2.13E-01	1.08E-01	9.43E-02	8.89E-02	8.47E-02	8.26E-02	8.06E-02	7.99E-02	7.88E-02	7.82E-02	7.76E-02	7.63E-02	7.55E-02	7.55E-02	7.49E-02	7.59E-02	7.54E-02	7.48E-02	7.50E-02
[3.14 .. 3.23]	1.49E-01	1.59E-01	1.86E-01	9.13E-02	7.69E-02	7.11E-02	6.83E-02	6.70E-02	6.69E-02	6.72E-02	6.83E-02	6.98E-02	7.08E-02	7.06E-02	7.18E-02	7.21E-02	7.25E-02	7.44E-02	7.46E-02	7.49E-02	7.50E-02

(ppm)	22	23	24	25	26	27	28	29	30	31	32	33	34	35	36	37	38	39	40	41	42
[1.18 .. 1.26]	3.90E-02	3.89E-02	3.98E-02	3.96E-02	4.00E-02	4.01E-02	4.00E-02	3.90E-02	3.99E-02	4.00E-02	3.99E-02	3.99E-02	3.92E-02	3.92E-02	3.92E-02	3.95E-02	3.92E-02	3.91E-02	3.99E-02	4.36E-02	3.92E-02
[2.78 .. 2.87]	7.25E-02	7.34E-02	7.42E-02	7.29E-02	7.38E-02	7.46E-02	7.42E-02	7.17E-02	7.39E-02	7.40E-02	7.30E-02	7.33E-02	7.28E-02	7.16E-02	7.09E-02	7.13E-02	7.08E-02	7.01E-02	7.20E-02	8.03E-02	7.06E-02
[3.14 .. 3.23]	7.45E-02	7.41E-02	7.62E-02	7.49E-02	7.58E-02	7.53E-02	7.57E-02	7.44E-02	7.56E-02	7.55E-02	7.61E-02	7.62E-02	7.50E-02	7.58E-02	7.53E-02	7.59E-02	7.56E-02	7.49E-02	7.61E-02	8.10E-02	7.50E-02

Table S1.9. Integral data for the kinetic study of the HCN (2 M) and cysteamine hydrochloride (2 M) reaction initiated by KOH (0.1 M) at 40 °C in D_2O. Spectra recorded at 5-minute intervals. Signals integrated: cysteamine $-CH_2S-$ triplets (δ 2.78-2.87ppm) and *tert*-butanol internal standard (δ 1.18-1.26 ppm). *Corresponds to SI Fig. 3.21*

(ppm)	1	2	3	4	5	6	7	8	9	10	11	12	13	14	15	16	17	18	19	20	21
[1.20 .. 1.24]	3.52E-02	3.34E-02	3.32E-02	3.35E-02	3.40E-02	3.47E-02	3.52E-02	3.54E-02	3.56E-02	3.57E-02	3.57E-02	3.59E-02	3.59E-02	3.58E-02	3.54E-02	3.52E-02	3.50E-02	3.47E-02	3.43E-02	3.39E-02	3.36E-02

[2.81 .. 2.88]	1.87E-01	1.78E-01	1.74E-01	1.71E-01	1.69E-01	1.68E-01	1.66E-01	1.63E-01	1.60E-01	1.56E-01	1.53E-01	1.50E-01	1.47E-01	1.43E-01	1.39E-01	1.35E-01	1.32E-01	1.28E-01	1.24E-01	1.20E-01	1.17E-01
[3.17 .. 3.24]	1.92E-01	1.82E-01	1.79E-01	1.76E-01	1.74E-01	1.73E-01	1.71E-01	1.68E-01	1.64E-01	1.61E-01	1.58E-01	1.54E-01	1.51E-01	1.47E-01	1.43E-01	1.40E-01	1.36E-01	1.33E-01	1.29E-01	1.26E-01	1.23E-01

22	23	24	25	26	27	28	29	30	31	32	33	34	35	36	37	38	39	40	41	42	43	44
3.32E-02	3.30E-02	3.28E-02	3.27E-02	3.27E-02	3.28E-02	3.31E-02	3.34E-02	3.35E-02	3.36E-02	3.38E-02	3.38E-02	3.39E-02	3.39E-02	3.39E-02	3.39E-02	3.39E-02	3.39E-02	3.39E-02	3.38E-02	3.38E-02	3.38E-02	3.39E-02
1.14E-01	1.12E-01	1.09E-01	1.07E-01	1.05E-01	1.04E-01	1.03E-01	1.02E-01	1.01E-01	9.99E-02	9.91E-02	9.79E-02	9.67E-02	9.55E-02	9.42E-02	9.29E-02	9.23E-02	9.11E-02	9.04E-02	8.90E-02	8.80E-02	8.72E-02	8.68E-02
1.20E-01	1.18E-01	1.15E-01	1.13E-01	1.11E-01	1.10E-01	1.09E-01	1.08E-01	1.06E-01	1.05E-01	1.04E-01	1.03E-01	1.02E-01	1.01E-01	9.96E-02	9.85E-02	9.76E-02	9.66E-02	9.57E-02	9.47E-02	9.39E-02	9.30E-02	9.26E-02

Table S1.9. Integral data for the kinetic study of the HCN (2 M) and cysteamine hydrochloride (2 M) reaction in 1 M potassium phosphate buffer (pH 6.5) at 40 °C in D_2O. Spectra recorded at 5-minute intervals. Signals integrated: cysteamine $-CH_2S-$ triplets (δ 2.81-2.88 ppm) and *tert*-butanol internal standard (typically δ 1.20-1.24 ppm). *Corresponds to SI Fig. 3.23*

10. Appendix S2: Matlab scripts and framework for mass spectrometry data analysis and compound identification

This section outlines the methodology and computational tools used for the identification and characterization of compounds formed in the reaction between HCN and cysteamine, based on mass spectrometry (MS) data obtained with ^{12}C, ^{13}C, and ^{15}N isotopically labeled HCN. The approach relies on an Integer Vector Optimization (IVO) algorithm, implemented in MATLAB, to deduce the combination of fundamental chemical transformations leading to each observed species.

1. Core principles of the analytical approach

The complexity of the reaction mixture necessitates a systematic approach to elucidate the composition of its components. Our method is based on the following core principles:

Isotopic Labeling: The use of $K^{13}CN$ and $KC^{15}N$ alongside natural abundance KCN allows for the precise determination of the number of carbon ($n(C_{HCN})$) and nitrogen ($n(N_{HCN})$) atoms originating from HCN in each detected ion. This is achieved by observing the characteristic mass shifts in the MS spectra of the labeled compounds relative to the unlabeled ones.

Fundamental Reactions: The vast number of potential reactions is simplified by considering a set of six fundamental chemical transformations that a precursor molecule can undergo to form a given product. These include:

- Addition of HCN (+HCN)
- Addition of cysteamine (+Cys)
- Addition of H_2O (+H_2O)
- Elimination of NH_3 (-NH_3)
- Oxidation (-2H)
- Reduction (+2H)

Integer Vector Optimization (IVO): For each triplet of experimentally observed m/z values (corresponding to the ^{12}C, ^{13}C, and ^{15}N isotopologues of a presumed $[M+H]^+$ ion), the algorithm determines the most plausible combination of the six fundamental reactions (i.e., the number of times each reaction occurred) that would result

in the observed m/z and isotopic shifts. This is framed as an integer linear programming problem, aiming to find the shortest sequence of these fundamental reactions.

2. Workflow for data analysis

The analytical workflow comprises several sequential steps:

Manual Peak Triplet Identification: Initial analysis of the raw MS data (from experiments with $K^{12}CN$, $K^{13}CN$, and $KC^{15}N$) is performed manually. The operator visually inspects the spectra (either printed or on-screen) to identify potential "triplets" – sets of peaks where a signal in the ^{13}C spectrum is shifted to a higher m/z relative to the ^{12}C spectrum, and a corresponding signal is sought in the ^{15}N spectrum. These preliminarily identified m/z values for each triplet are recorded. *(This manual step is crucial due to the complexity and potential for noise in the raw MS data, making full automation of peak picking and triplet matching challenging with the tools available at the time of the original work.)*

Database of Reaction Combinations (data_names.xls): A reference database (data_names.xls) is maintained. This database, initially generated by the names_for_comp.m script (see Appendix S2.3.1), contains a comprehensive list of theoretically possible combinations of the six fundamental reaction counts, with each unique combination assigned a unique identifier (name).

Computational Deconstruction via check_for_comp.m:

The operator uses the check_for_comp.m script (see Appendix S2.3.2), running in MATLAB.

For each manually identified triplet of m/z values, the operator inputs these values into the script.

The script calculates the target parameters: the number of HCN-derived carbon atoms ($n(C_{HCN})$), the number of HCN-derived nitrogen atoms ($n(N_{HCN})$), an estimated range for the number of cysteamine units (n(Cys)), and the nominal mass of the compound.

Using the intlinprog function, the script solves for the integer vector representing the counts of each of the six fundamental reactions that best explain the input data. The script iterates through a small range of possible n(Cys) values to find a consistent solution.

The resulting reaction count vector is then compared against the data_names.xls database.

If a match is found, the script outputs the pre-assigned name for that compound (i.e., for that specific combination of reaction counts).

If the derived reaction count vector represents a new, previously uncataloged combination, the script indicates this. This new combination and its corresponding experimental data would then be manually considered for addition to an updated version of the data_names.xls database or a working list.

Data Consolidation: Parallel to the computational analysis, a separate table is manually maintained to log the identified compounds (based on their generated names or reaction vectors) for each specific experiment or experimental condition.

3. MATLAB Scripts

```
% names_for_comp.m
% Author: Alexandr Novichkov
% Date: 12/2023
% Description:
% This script generates a table ('data_names.xls') that maps unique compound
% identifiers (names) to specific combinations of six fundamental reaction counts.
% These reaction counts represent the number of times each fundamental
% reaction (HCN addition, Cysteamine addition, H2O addition, NH3 elimination,
% Oxidation, Reduction) is involved in the formation of a compound.
% The generated table is used by 'check_for_comp.m' for identifying
% compounds based on their reaction count vectors.

% --- User-defined matrix of reaction count combinations ---
% Each row represents a unique compound (or a potential compound)
% Each column corresponds to one of the six fundamental reactions:
% Col 1: Number of HCN additions (+HCN)
% Col 2: Number of Cysteamine additions (+Cys)
% Col 3: Number of H2O additions (+H2O)
% Col 4: Number of NH3 eliminations (-NH3)
% Col 5: Number of Oxidations (-2H)
% Col 6: Number of Reductions (+2H)

% Example: matrix = [1 1 0 0 1 0; 1 1 0 0 0 0; ...];


matrix = [
    1 1 0 0 1 0;
    1 1 0 0 0 0;
    % ... (other compounds vectors) ...
    10 8 1 8 0 2
];

% --- Name generation settings ---
numCompounds = size(matrix, 1); % Number of compounds/combinations
% Mapping for generating part of the compound name.
% Using letters to represent the count of each reaction type (0 -> A, 1 -> B, etc.)
% This is just one way to create distinguishable names; the core is the reaction vector.
digitToCharMapping = 'ABCDEFGHIJK'; % Supports reaction counts from 0 to 10, letters cold be chosen in any way

% Preallocate cell array for storing names and reaction counts
% Each row: {Name, x1, x2, x3, x4, x5, x6}
compoundDataTable = cell(numCompounds, 7);

% --- Generate names and populate the data table ---
fprintf('Generating names for %d compound reaction vectors...\n', numCompounds);
for i = 1:numCompounds
    reactionCounts = matrix(i, :); % Get the current reaction count vector

    % Generate a unique name based on reaction counts
```

```
    % Example: R_B_B_A_A_B_A for counts [1 1 0 0 1 0]
        mappedChars = arrayfun(@(x) digitToCharMapping(x + 1), reactionCounts, 'UniformOutput', false);
    compoundName = ['R', mappedChars{:}];

    % Store the name and the original reaction counts
    compoundDataTable(i, :) = {compoundName, reactionCounts(1), reactionCounts(2), ...
                          reactionCounts(3), reactionCounts(4), reactionCounts(5), ...
                          reactionCounts(6)};
end

% --- Convert to table and save to Excel ---
% Define variable names for the output table
variableNames = {'Name', 'num_HCN_add', 'num_Cys_add', 'num_H2O_add', ...
                 'num_NH3_elim', 'num_Oxidations', 'num_Reductions'};
T_compound_names = cell2table(compoundDataTable, 'VariableNames', variableNames);

% Define the output file name
outputFileName = 'data_names_generated.xls';
try
    writetable(T_compound_names, outputFileName);
    fprintf('Successfully generated and saved table to "%s".\n', outputFileName);
catch ME
    fprintf('Error writing table to Excel file: %s\n', ME.message);
    fprintf('Please ensure you have write permissions and Excel is not locking the file.\n');
end

disp('Script "names_for_comp.m" finished.');
```

```
% check_for_comp.m
% Author: Alexandr Novichkov
% Date: 12/2023
% Description:
% This script implements the Integer Vector Optimization (IVO) algorithm
% to determine the combination of six fundamental chemical reactions that
% corresponds to a given set of mass spectrometry (MS) data (m/z values for
% 12C, 13C, and 15N isotopologues). It then attempts to identify the
% compound by comparing the derived reaction count vector with a pre-generated
% database of known compounds and their reaction counts ('data_names_generated.xls').

% --- Load pre-generated compound database ---
databaseFileName = 'data_names_generated.xls';
try
    compoundDatabase = readtable(databaseFileName);
    fprintf('Successfully loaded compound database from "%s".\n', databaseFileName);
catch ME
    fprintf('Error loading compound database "%s": %s\n', databaseFileName, ME.message);
    fprintf('Please ensure the file exists in the current directory or provide the full path.\n');
    fprintf('You can generate it using "names_for_comp.m".\n');
    return; % Exit if database cannot be loaded
end

% --- Define fundamental reaction vectors ---
% Each row represents a reaction vector:
% [d_n(C)_HCN, d_n(N)_HCN, d_n(Cys), d_mass]
% where:
%   d_n(C)_HCN: Change in the number of Carbon atoms from HCN
%   d_n(N)_HCN: Change in the number of Nitrogen atoms from HCN
%   d_n(Cys):   Change in the number of Cysteamine units (not directly encoded here, but calculated)
%   d_mass:     Change in mass
% The script solves for x_i, where x_i is the number of times reaction_i occurs.
% Aeq*x = beq, where Aeq is reactions_matrix' and beq is target_vector.

% Columns in reactions_matrix:
% 1: Change in count of HCN-derived C atoms (for target_13C)
% 2: Change in count of HCN-derived N atoms (for target_15N)
% 3: Change in count of Cysteamine molecules (for target_cys)
% 4: Change in overall mass (for target_mass)
reactions_matrix = [
% Reaction        d_n(C)_HCN  d_n(N)_HCN  d_n(Cys)  d_Mass
  1,          1,          0,        27;  % 1. HCN addition
  0,          0,          1,        77;  % 2. Cysteamine addition
  0,          0,          0,        18;  % 3. H2O addition
  0,         -1,          0,       -17;  % 4. NH3 elimination
  0,          0,          0,        -2;  % 5. Oxidation (-2H)
  0,          0,          0,         2   % 6. Reduction (+2H)
];
% Note: The d_n(Cys) column here is for consistency in the matrix structure.
% The actual number of Cys units (target_cys) is handled as part of the 'beq' vector.

% --- Integer Linear Programming (ILP) setup ---
numReactionTypes = size(reactions_matrix, 1);
objectiveFunction = ones(numReactionTypes, 1); % Minimize sum of reaction counts (shortest path)
```

```
% Lower and upper bounds for reaction counts (x_i >= 0)
lowerBounds = zeros(numReactionTypes, 1);
upperBounds = Inf(numReactionTypes, 1); % No upper limit on how many times a reaction can occur

% Define all variables as integers
integerConstraints = 1:numReactionTypes;

% --- User Interaction ---
num_iterations_str = input('Enter the number of compounds to analyze (iterations): ', 's');
num_iterations = str2double(num_iterations_str);
if isnan(num_iterations) || num_iterations < 1
    disp('Invalid number of iterations. Exiting.');
    return;
end

fprintf('\n--- Starting Compound Analysis ---\n');
for iteration = 1:num_iterations
    fprintf('\n--- Analyzing Compound %d of %d ---\n', iteration, num_iterations);

    % Input experimental MS data (m/z values)
    mass_12C_str = input('Enter m/z for 12C isotopologue (e.g., M+H)+: ', 's');
    mass_13C_str = input('Enter m/z for 13C-labeled isotopologue: ', 's');
    mass_15N_str = input('Enter m/z for 15N-labeled isotopologue: ', 's');

    mass_12C = str2double(mass_12C_str);
    mass_13C = str2double(mass_13C_str);
    mass_15N = str2double(mass_15N_str);

    if isnan(mass_12C) || isnan(mass_13C) || isnan(mass_15N)
        disp('Invalid mass input. Skipping this compound.');
        continue;
    end

    % --- Transform MS data into target variables for ILP ---
    % Target mass is calculated assuming [M+H]+, so nominal mass M = mass_12C - 1
    target_nominal_mass = mass_12C - 1;
    % Target number of HCN-derived Carbon atoms
    target_num_HCN_C = mass_13C - mass_12C;
    % Target number of HCN-derived Nitrogen atoms
    target_num_HCN_N = mass_15N - mass_12C;

    if target_num_HCN_C < 0 || target_num_HCN_N < 0
        disp('Warning: Calculated 13C or 15N shift is negative. Check input masses.');
        % Potentially skip or allow user to re-enter
    end

    % Estimate the number of Cysteamine units (n(Cys))
    % mass_M = n(C)_HCN * mass_C_HCN + n(N)_HCN * mass_N_HCN + n(Cys) * mass_Cys_unit + ...
    % Simplified estimation:
    % remaining_mass = target_nominal_mass - (target_num_HCN_C * 12) - (target_num_HCN_N * 14)
    % Assumes C and N from HCN are the main contributors besides Cys.
    % This is a heuristic; the ILP will confirm the n(Cys).
    % Molecular weight of Cys unit (C2H7NS) = 77.15. Using 77 for integer calculations.
```

```
    estimated_cys_start = floor((target_nominal_mass - target_num_HCN_C * 27 - (target_num_HCN_N - target_num_HCN_C) * 14) / 77);
    % The (target_num_HCN_N - target_num_HCN_C) term estimates N atoms not part of a CN from HCN.
    % A simpler approximation from your original code:
    estimated_cys_start_orig = floor((target_nominal_mass - target_num_HCN_C*12 - target_num_HCN_N*14)/77);

    % Ensure non-negative and provide a small search window
    estimated_cys_start_final = max(0, estimated_cys_start_orig - 1); % Allow a slightly lower start
    estimated_cys_end_final   = max(1, estimated_cys_start_orig + 2); % Allow a slightly higher end

    fprintf('Estimated n(Cys) search range: %d to %d\n', estimated_cys_start_final, estimated_cys_end_final);

    solutionFoundForCompound = false;
    % Iterate over the estimated range of Cysteamine units
    for current_target_cys = estimated_cys_start_final:estimated_cys_end_final
        % Target vector for ILP: [target_num_HCN_C; target_num_HCN_N; current_target_cys; target_nominal_mass]
        % This defines the system Aeq * x = beq
        target_vector_beq = [
            target_num_HCN_C;    % Sum of x_i * reactions_matrix(i,1) should equal this
            target_num_HCN_N;    % Sum of x_i * reactions_matrix(i,2) should equal this
            current_target_cys;  % Sum of x_i * reactions_matrix(i,3) should equal this
            target_nominal_mass  % Sum of x_i * reactions_matrix(i,4) should equal this
        ];

        % Equality constraints matrix (Aeq)
        Aeq_matrix = reactions_matrix'; % Transpose to match dimensions for Aeq*x = beq

        % Solve the Integer Linear Programming problem
        % options = optimoptions('intlinprog','Display','off'); % Suppress solver messages
        % [x_solution, fval, exitflag] = intlinprog(objectiveFunction, integerConstraints, ...
        %                                 [], [], Aeq_matrix, target_vector_beq, ...
        %                                 lowerBounds, upperBounds, [], options);
        [x_solution, ~, exitflag] = intlinprog(objectiveFunction, integerConstraints, ...
                                    [], [], Aeq_matrix, target_vector_beq, ...
                                    lowerBounds, upperBounds);


        % Check if a valid solution was found
        if exitflag == 1 % 1 indicates optimal solution found
            reaction_counts_vector = round(x_solution); % Ensure integer counts

            % Check if this solution exists in the database
            isKnownCompound = false;
            compoundNameInDb = 'N/A';
            for i = 1:size(compoundDatabase, 1)
                db_reaction_counts = table2array(compoundDatabase(i, 2:end)); % Assuming cols 2-7 are reaction counts
                if isequal(reaction_counts_vector', db_reaction_counts)
                    compoundNameInDb = compoundDatabase.Name{i};
                    isKnownCompound = true;
                    break;
                end
            end

            % Display results
```

```
        fprintf('  Solution found with n(Cys) = %d:\n', current_target_cys);
        fprintf('    Reaction Counts (HCN, Cys, H2O, -NH3, -Ox, +Red): [%s]\n', sprintf('%d ', reaction_counts_vector));
        % fprintf('    Target Vector (nC_HCN, nN_HCN, nCys, Mass_M): [%s]\n', sprintf('%g ', target_vector_beq)); % For
debugging

        if isKnownCompound
          fprintf('    Compound IDENTIFIED as: %s\n', compoundNameInDb);
        else
          fprintf('    This is a NEW combination of reactions! (Not found in database).\n');
        end
        solutionFoundForCompound = true;
        break; % Exit cys_loop once a solution is found for this compound
      end
    end % End of loop over estimated_cys_count

    if ~solutionFoundForCompound
      fprintf('  No valid combination of reactions found for the input masses and estimated Cys range.\n');
      fprintf('  Consider broadening the n(Cys) search range or re-checking input MS data.\n');
      fprintf('    Target (nC_HCN, nN_HCN, Mass_M): [%g, %g, %g]\n', target_num_HCN_C, target_num_HCN_N,
target_nominal_mass);
    end
end % End of loop over iterations/compounds

disp('Script "check_for_comp.m" finished.');
```

4. Note on workflow optimization

The described workflow, particularly the manual steps of triplet identification, iterative input into check_for_comp.m, and manual database updating, was developed based on the computational tools and programming expertise available at the time of the original research. With current advancements in programming assistance, for instance, through interaction with AI language models, several aspects of this workflow could potentially be streamlined. This includes more automated batch processing of potential peak triplets, dynamic updating of the compound database within a single, more integrated script, and improved data input/output management, thereby enhancing the efficiency of the overall analytical process. However, the core IVO logic implemented in check_for_comp.m remains a robust and valid approach for the deconstruction of complex MS data in this chemical system.

10. Appendix S3: Computational deconstruction pathways for selected compounds

This appendix presents illustrative deconstruction pathways for a selection of compounds identified in the HCN-cysteamine reaction mixture. These pathways were computationally derived as described in ESI, Section 7 ("Computational Deconstruction"), utilizing a set of predefined graph transformation rules (detailed in ESI, Section 7). Each scheme visualizes a plausible sequence of reaction steps transforming simpler precursors into the target molecule. The labels used within these schemes (e.g., Mxxx, Px,x, rxx, dg_0) are internal identifiers generated by the computational software (mød) and correspond to specific molecular species or reaction rules applied. These deconstructions aid in understanding the potential origin and relationships between different components of the complex reaction network.

0.3.2 DG Hyper, dg_0

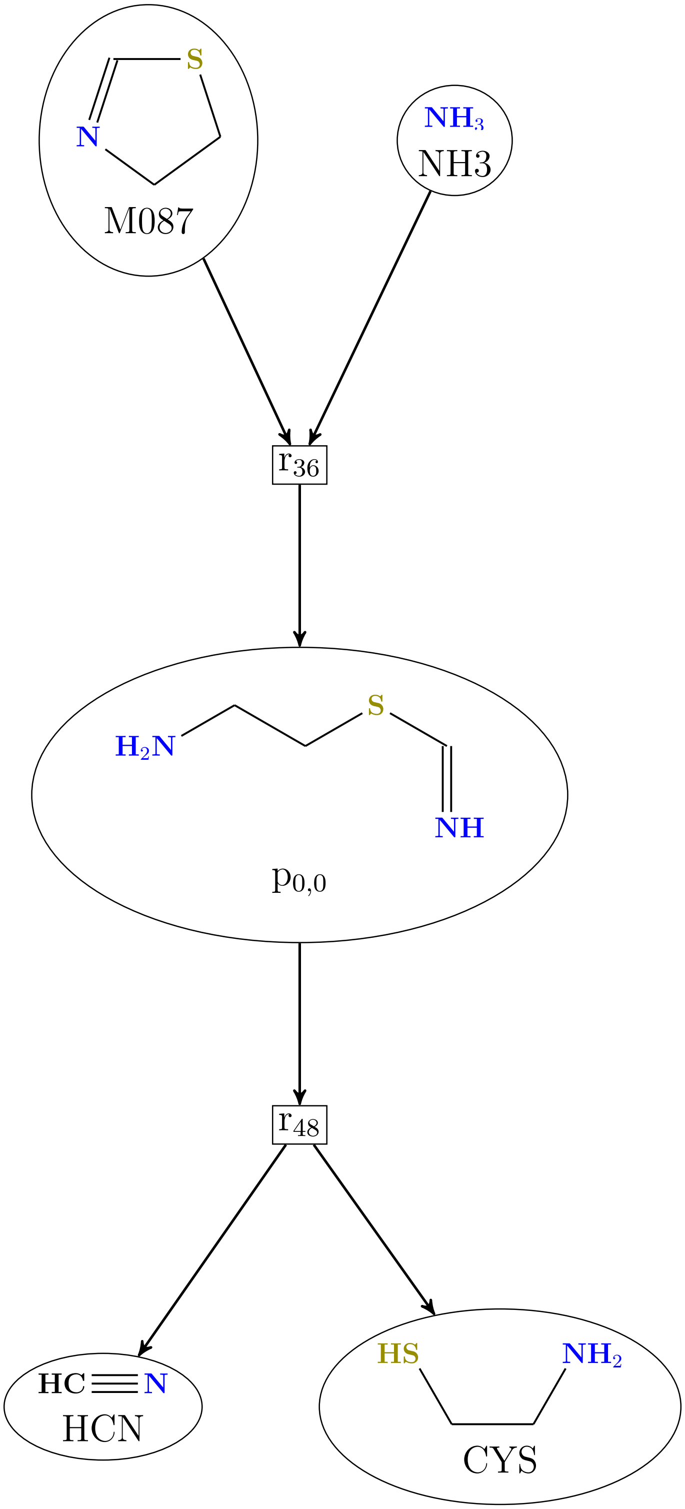

File: out/134_dg_0_11100

0.3.2 DG Hyper, dg_0

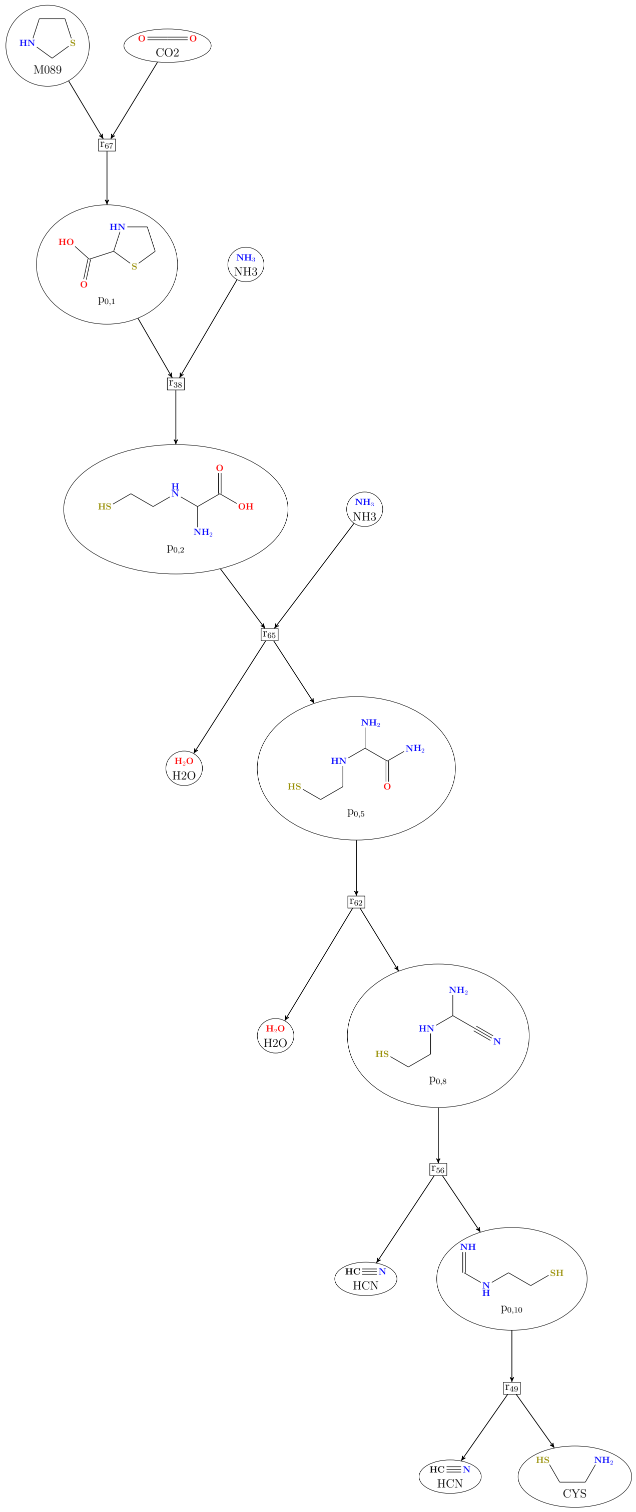

File: out/155_dg_0_11100

0.5 Catalyst p_{0,6}

0.5.1 DG Hyper, dg_0

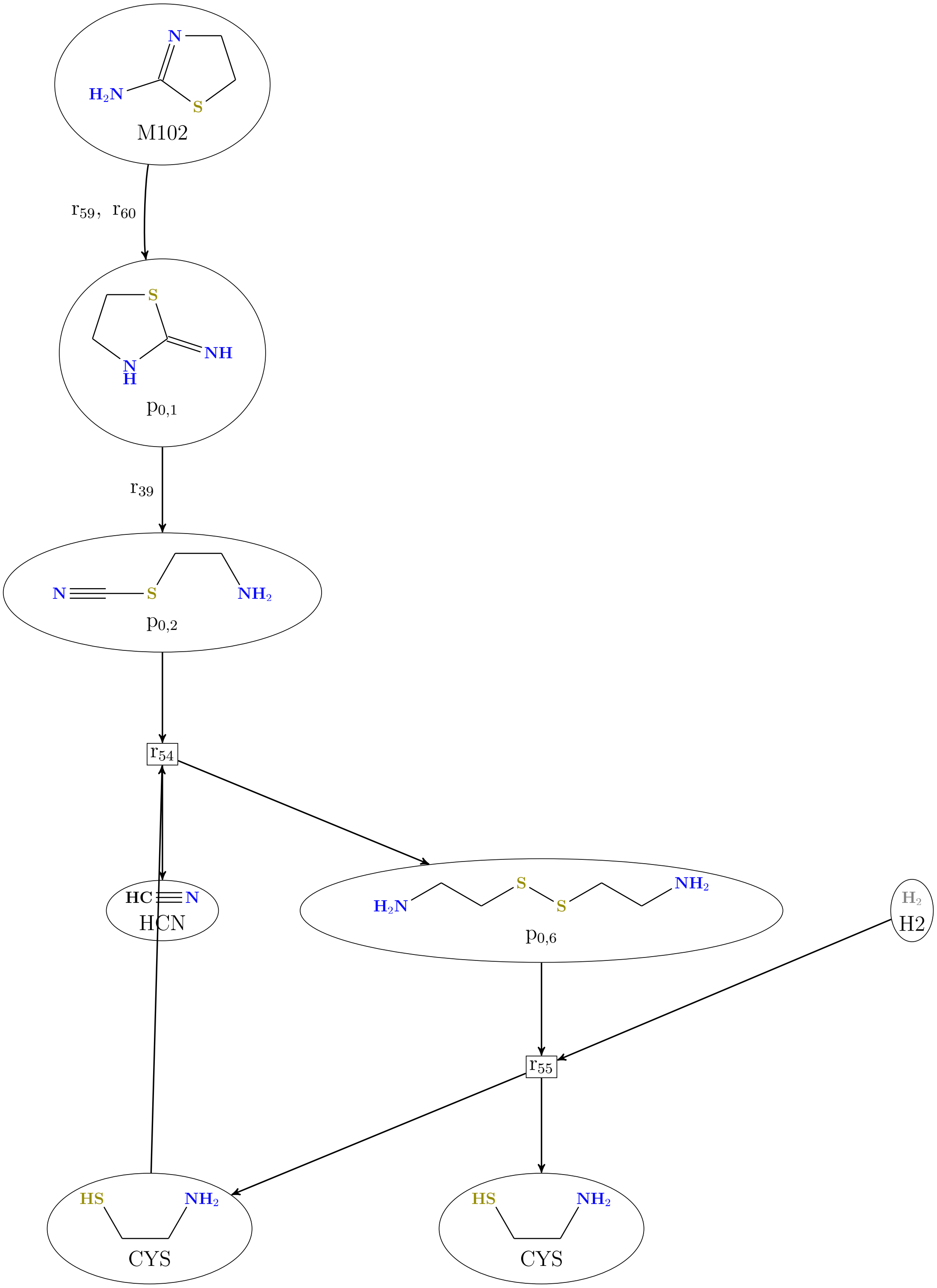

File: out/131_dg_0_11100

0.3.2 DG Hyper, dg_0

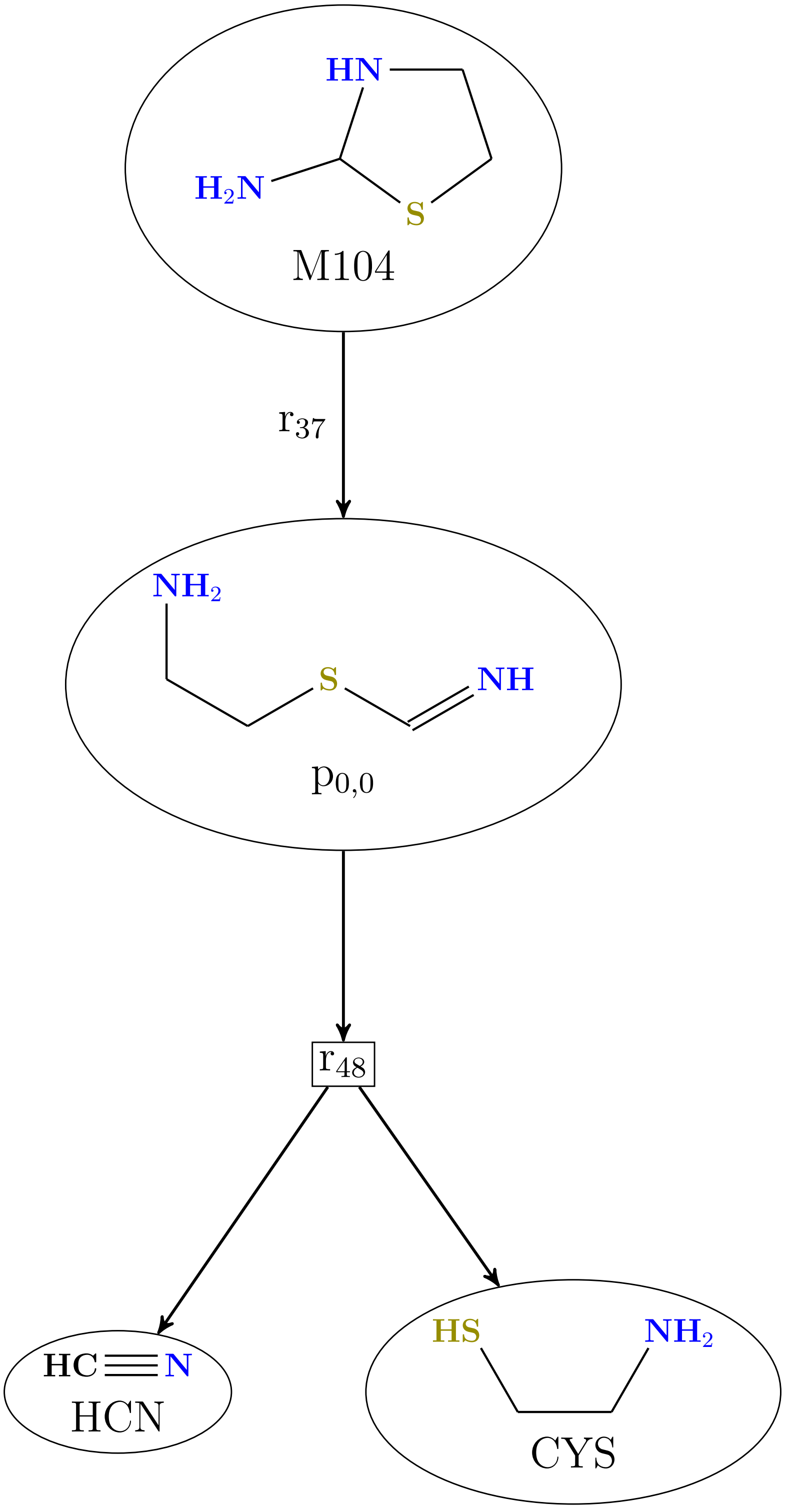

File: out/118_dg_0_11100

0.3.2 DG Hyper, dg_0

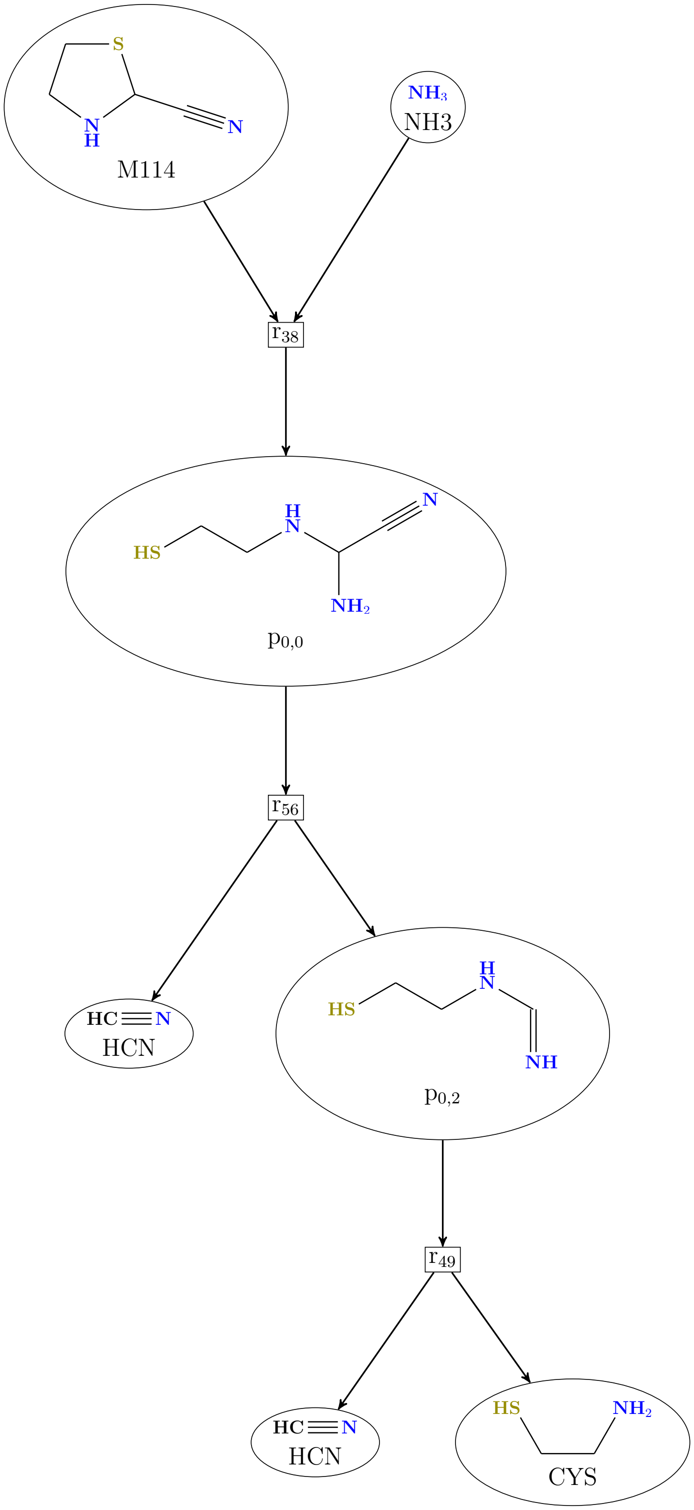

File: out/155_dg_0_11100

0.3.2 DG Hyper, `dg_0`

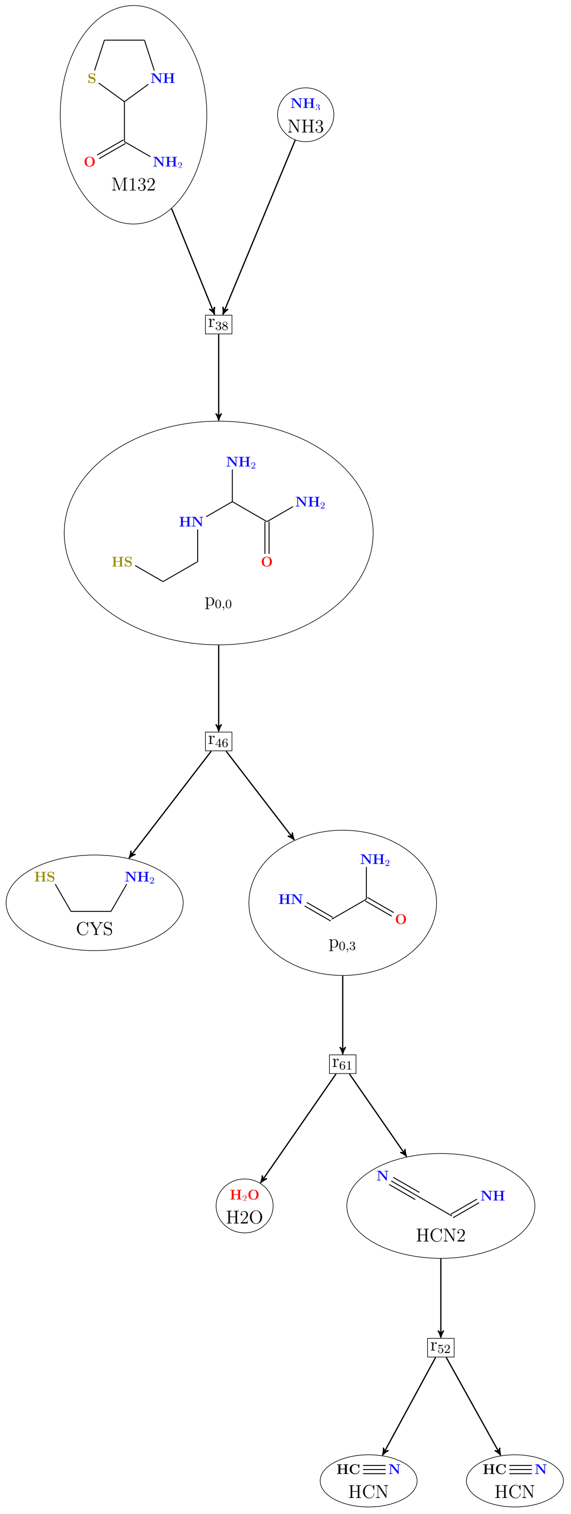

File: `out/123_dg_0_11100`

0.3.2 DG Hyper, dg_0

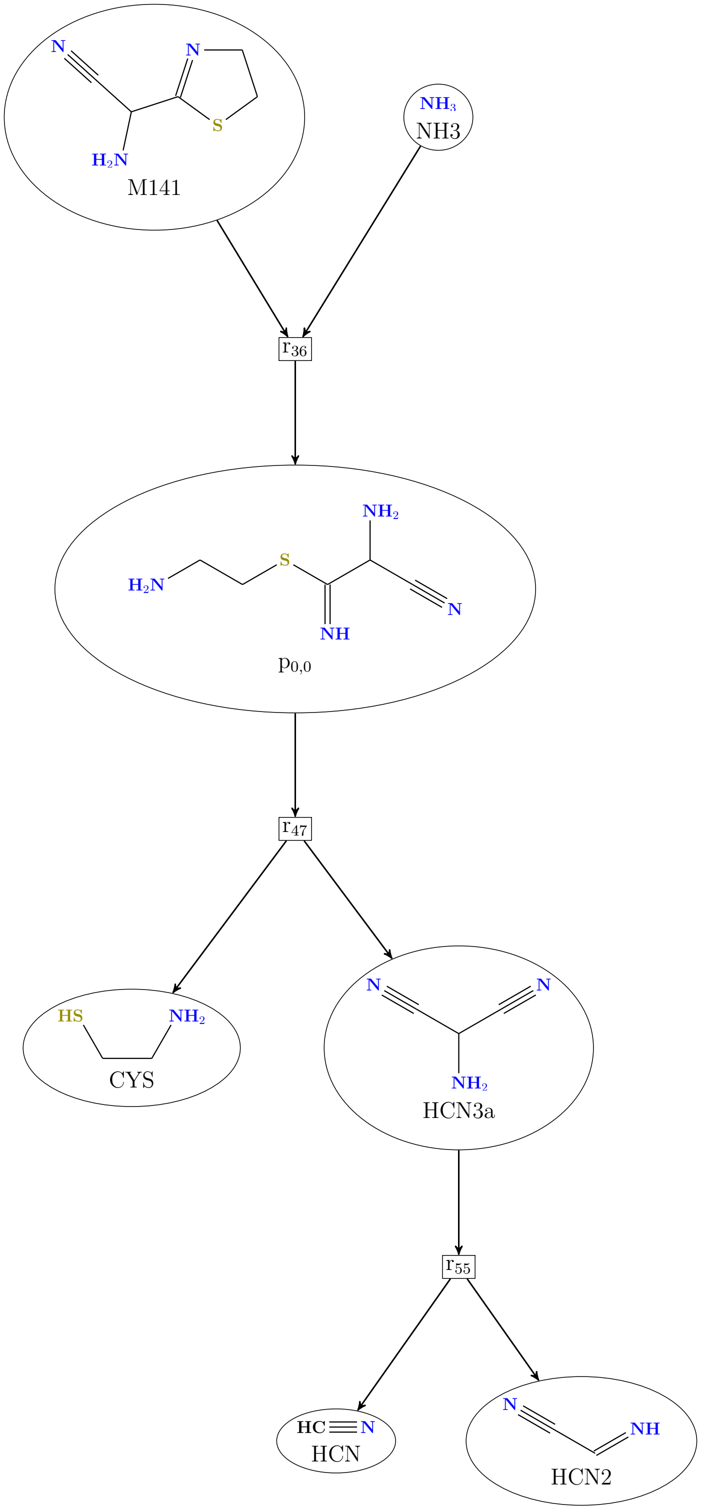

File: out/207_dg_0_11100

0.3.2 DG Hyper, dg_0

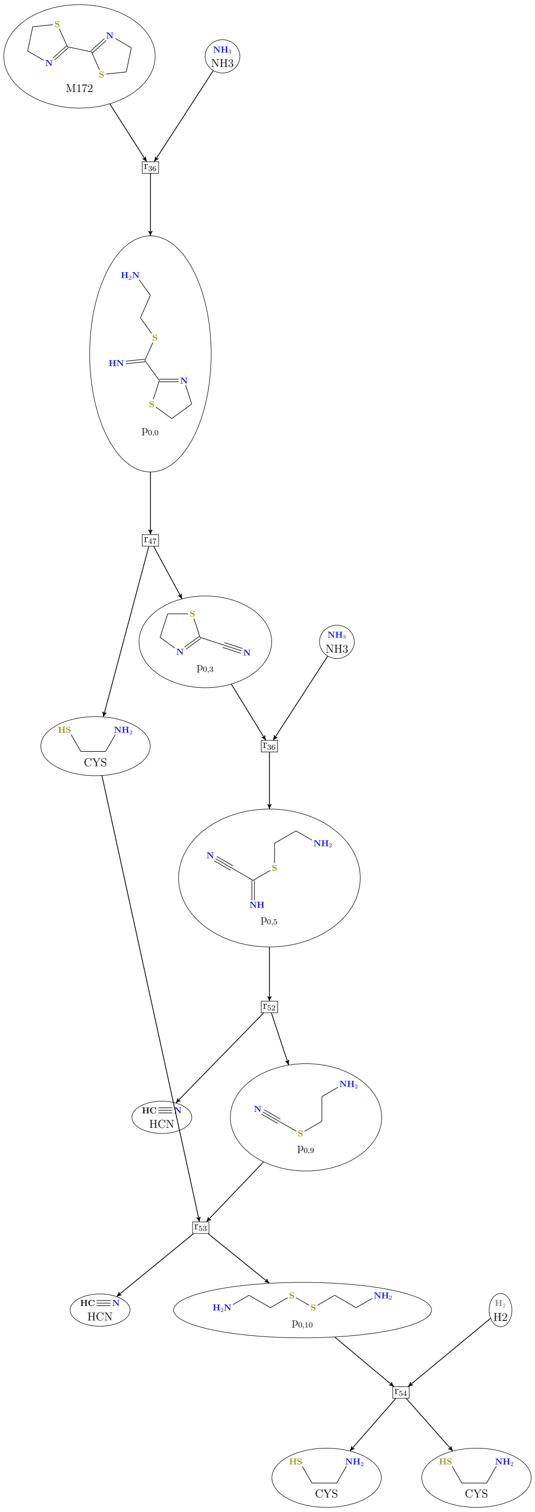

File: out/121_dg_0_11100

0.3.2 DG Hyper, dg_0

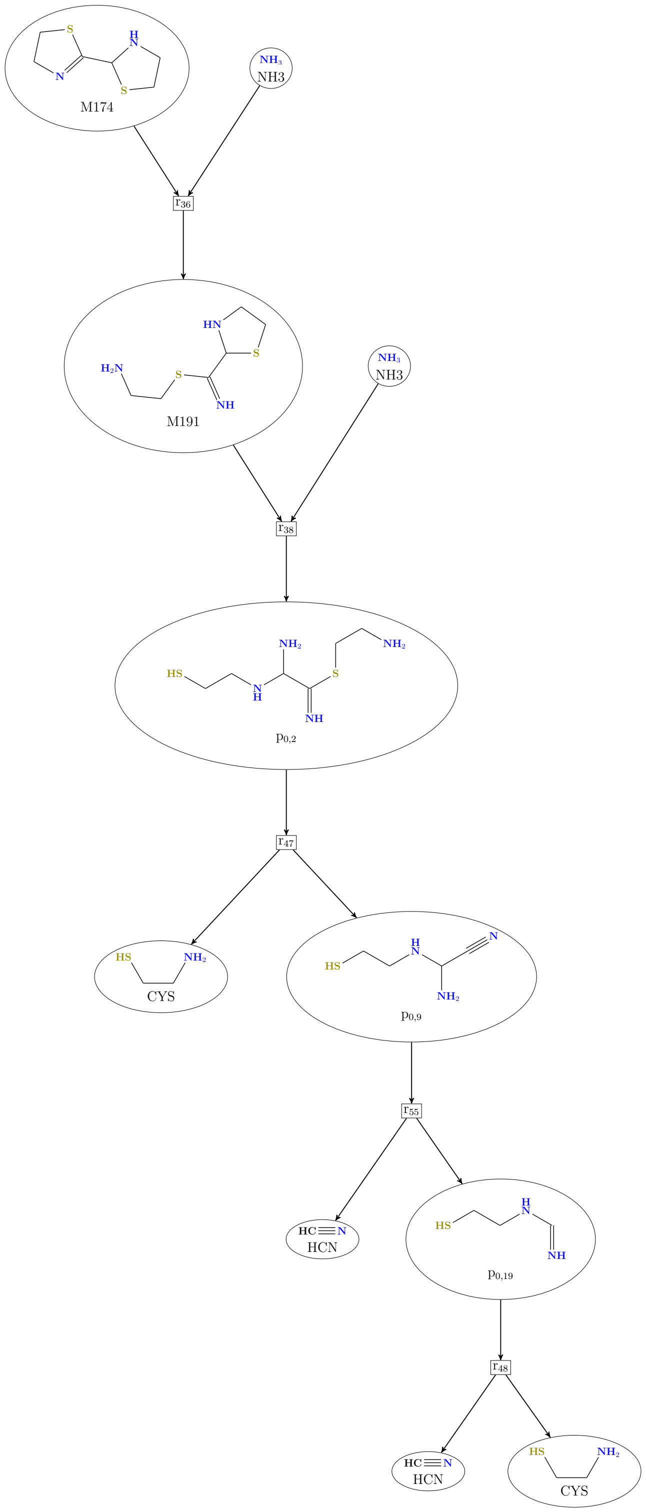

File: out/195_dg_0_11100

0.3.2 DG Hyper, dg_0

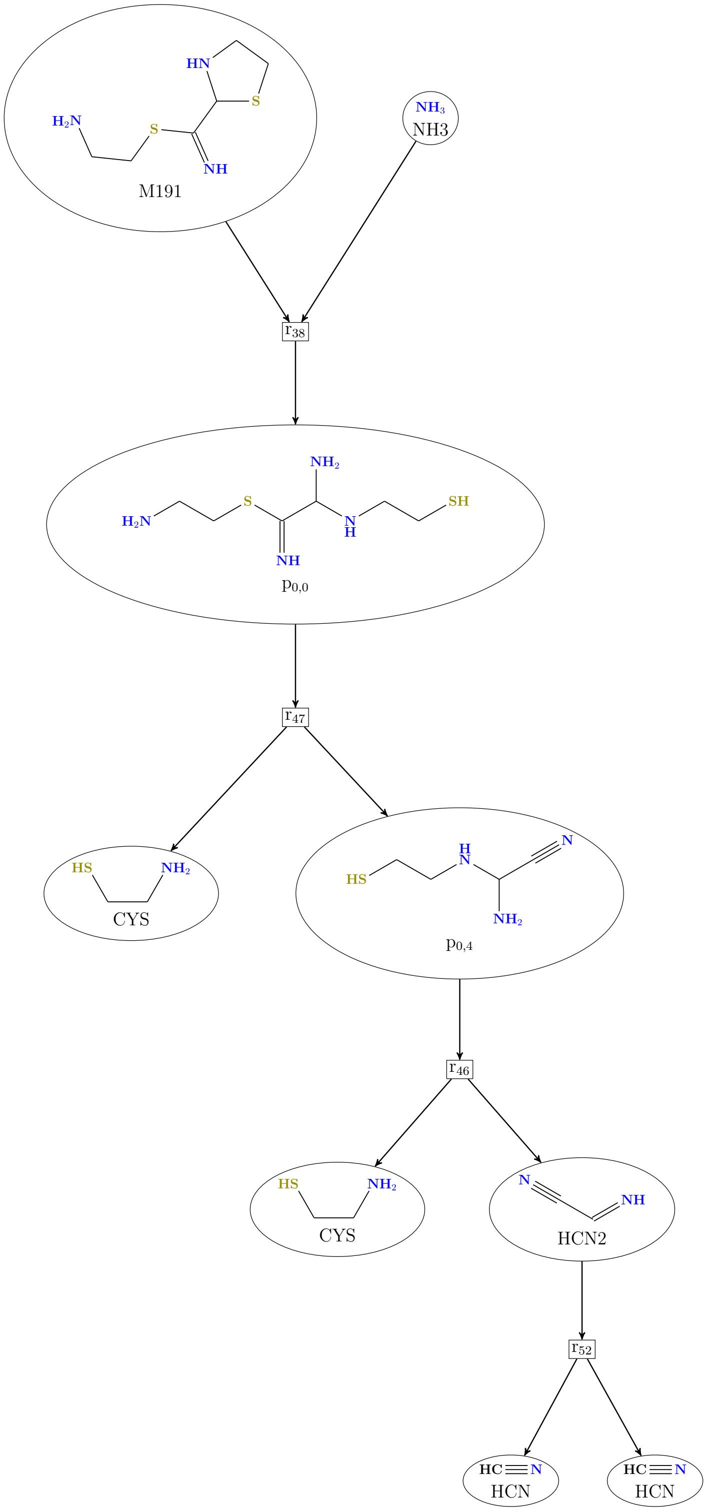

File: out/153_dg_0_11100

0.3.2 DG Hyper, dg_0

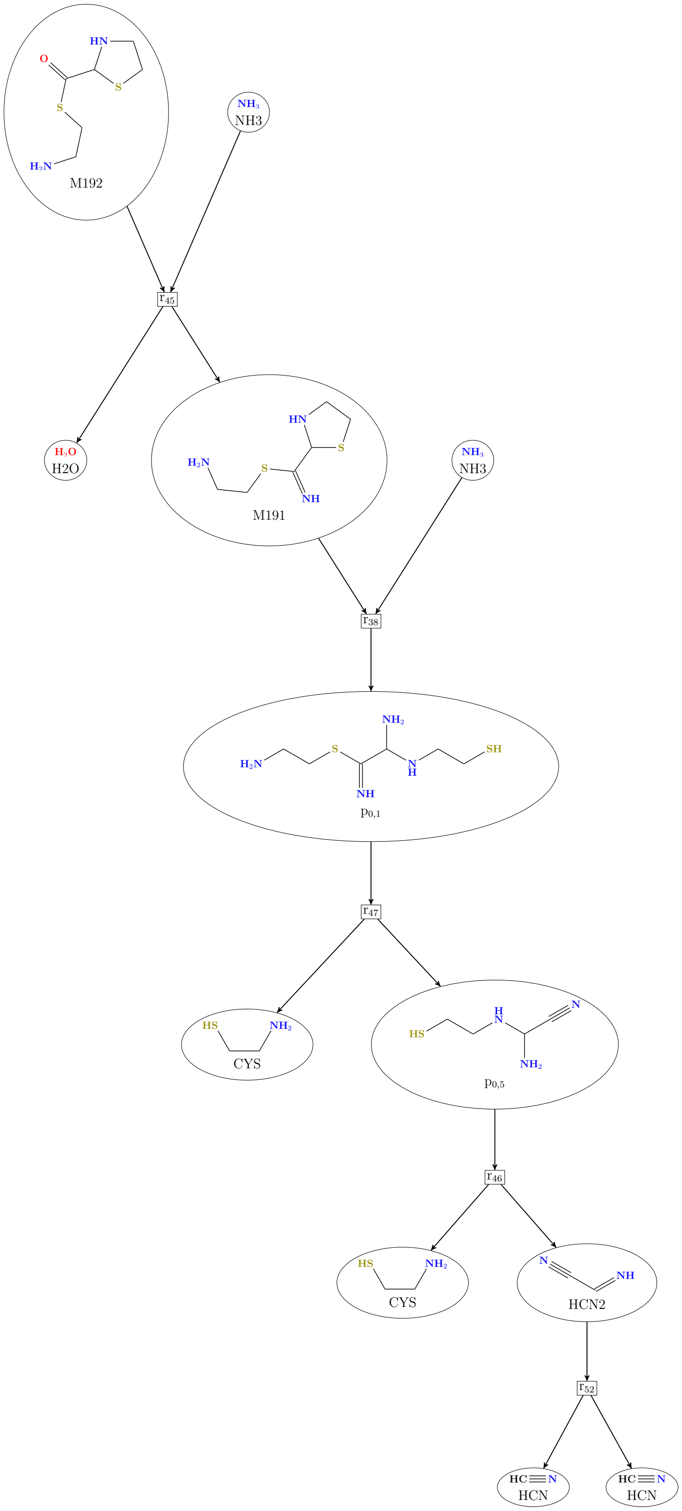

File: out/153_dg_0_11100

0.3.2 DG Hyper, dg_0

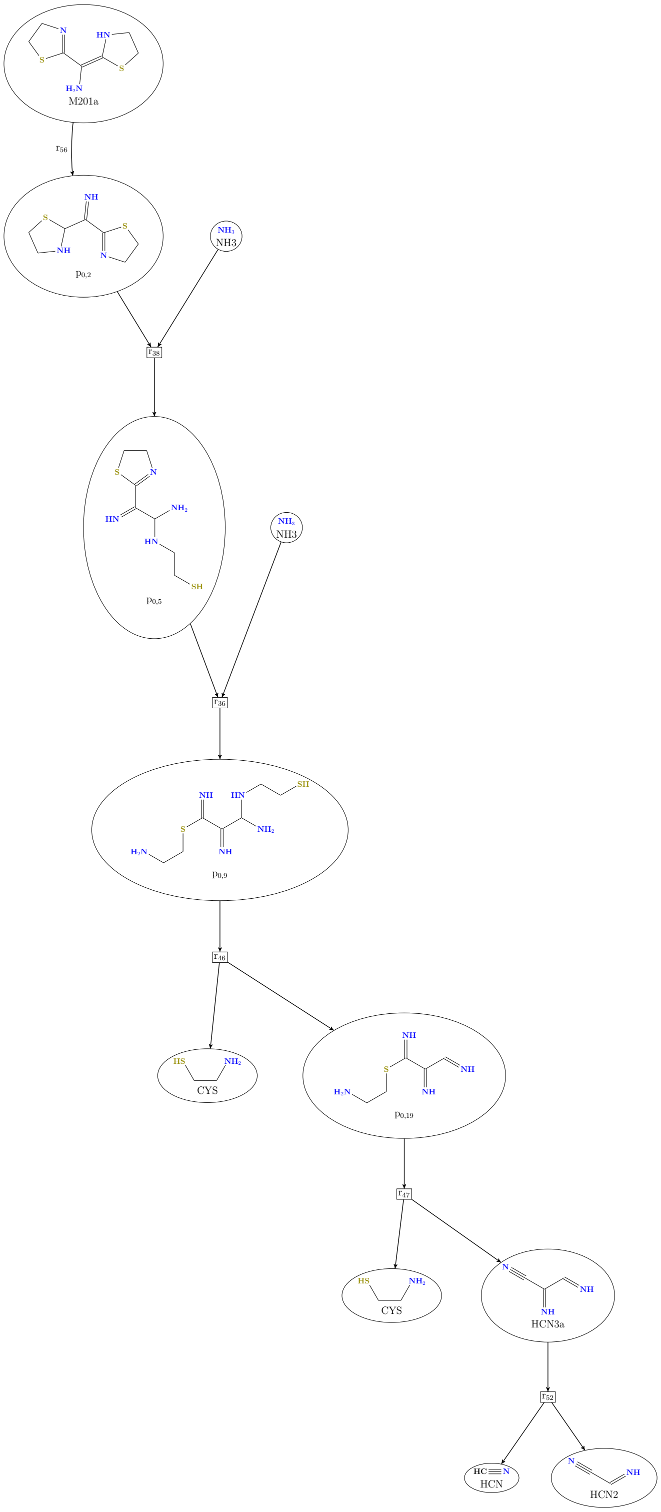

File: out/321_dg_0_11100

0.3.2 DG Hyper, dg_0

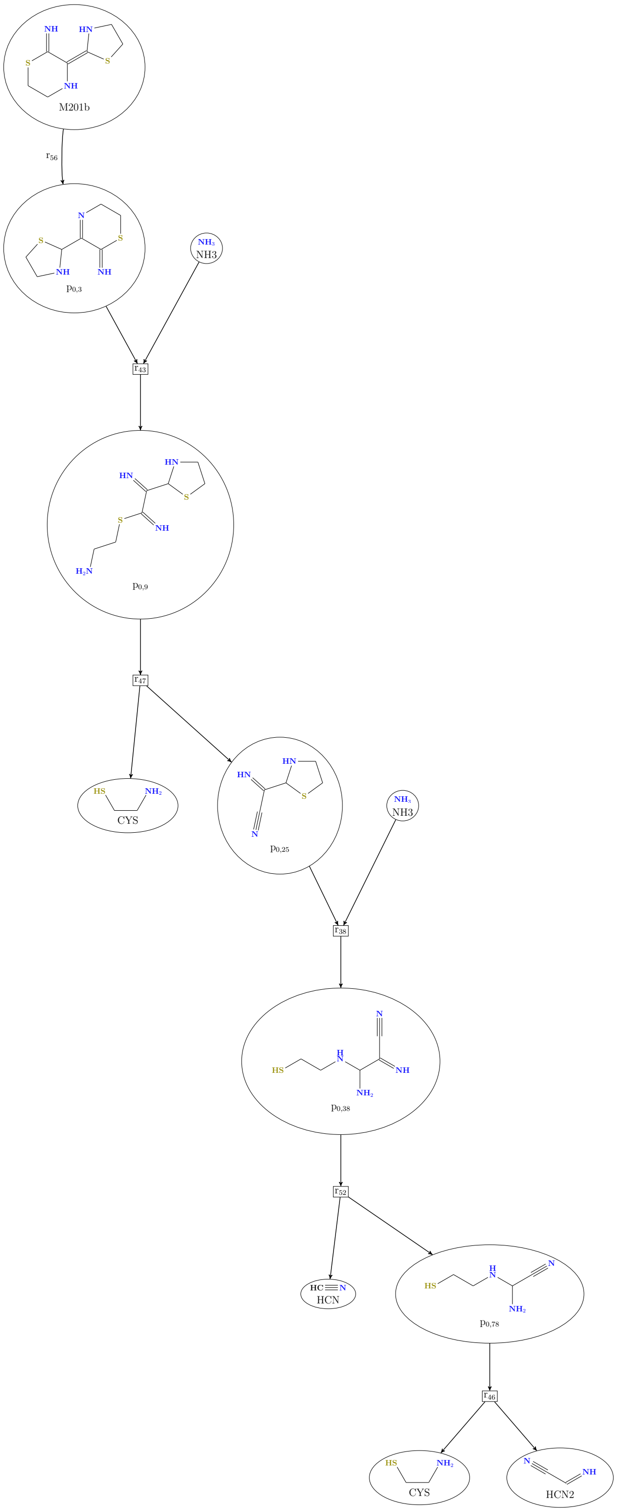

File: out/611_dg_0_11100

0.11 Catalyst p_{0,70}

0.11.1 DG Hyper, dg_0

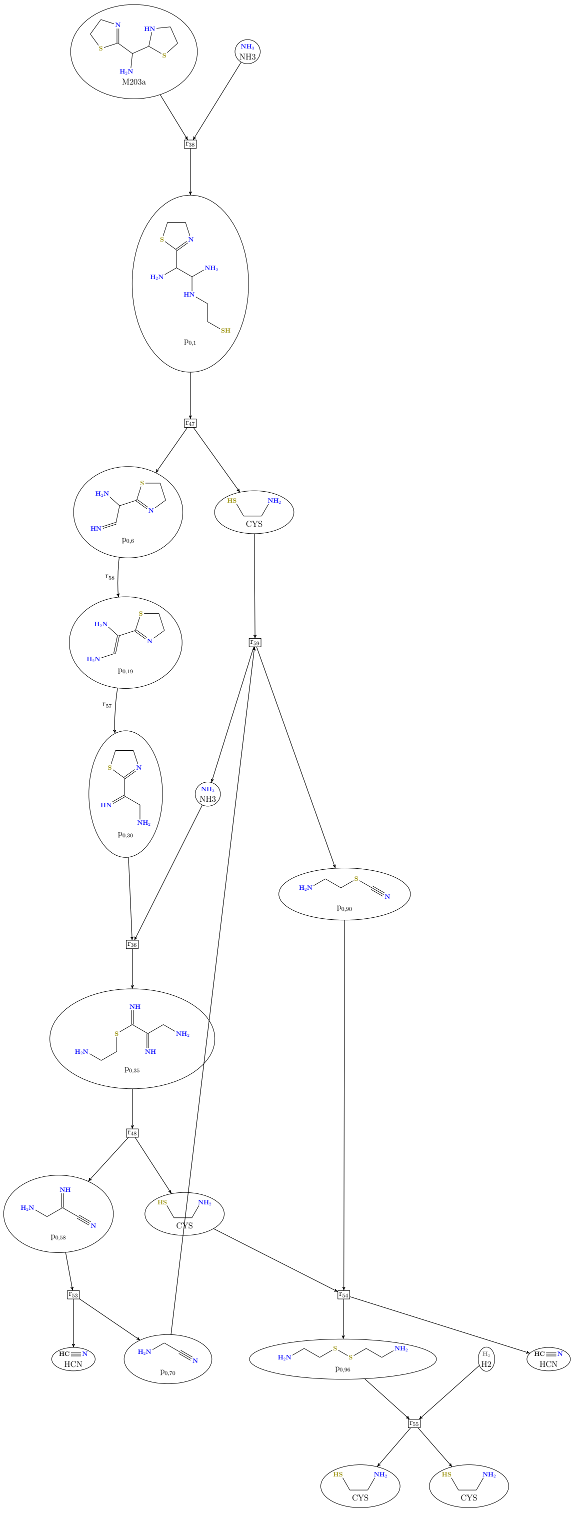

File: out/419_dg_0_11100

0.12 Catalyst p_{0,96}

0.12.1 DG Hyper, `dg_0`

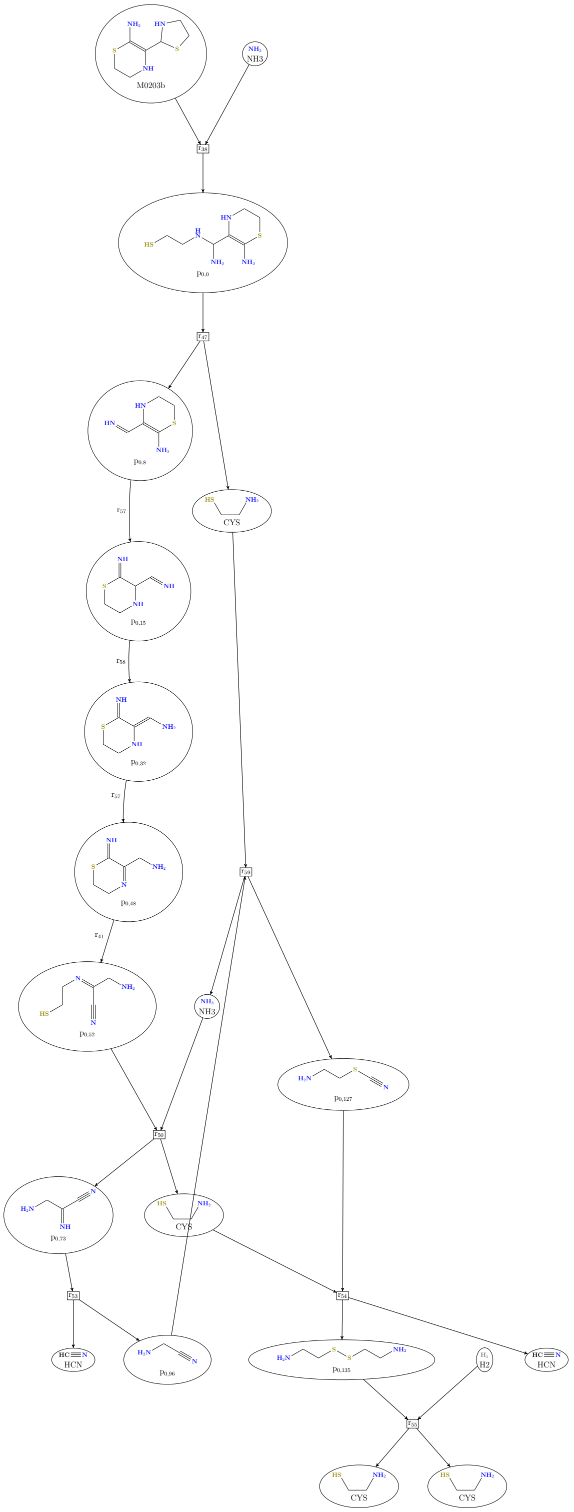

File: `out/492_dg_0_11100`

0.3.2 DG Hyper, dg_0

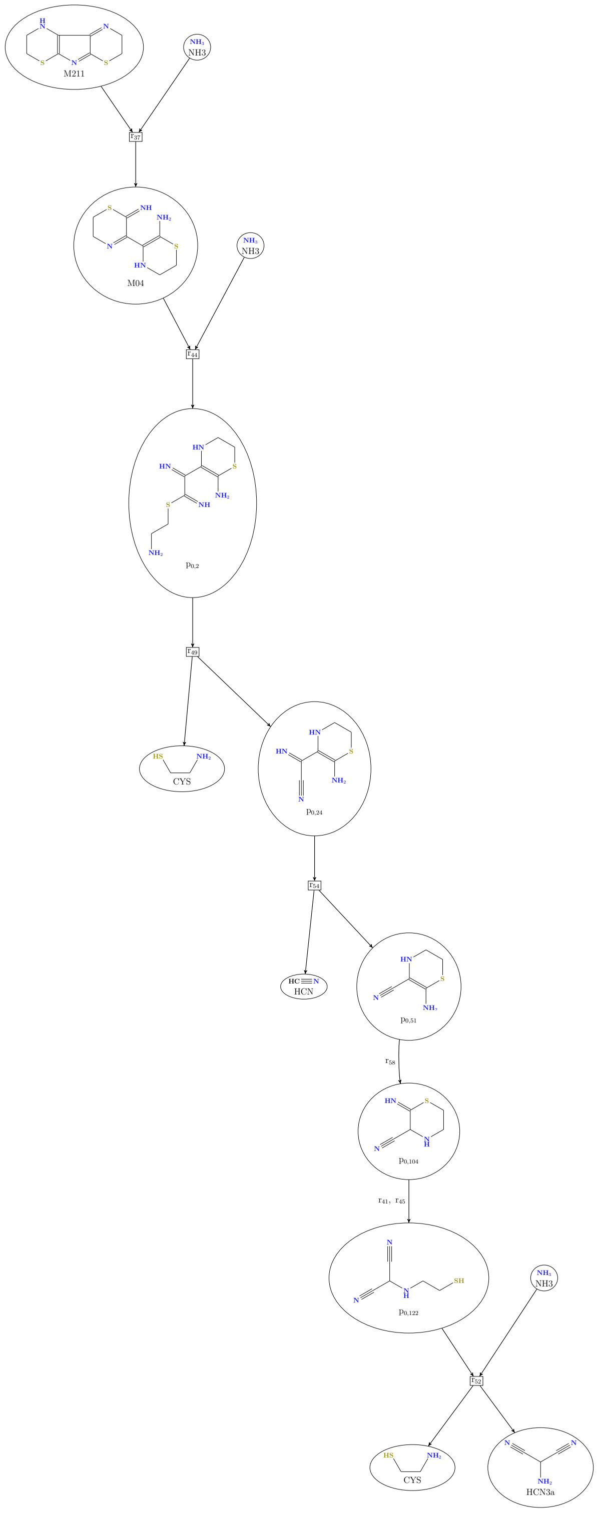

File: out/1479_dg_0_11100

0.15 Catalyst p_{0,273}

0.15.1 DG Hyper, `dg_0`

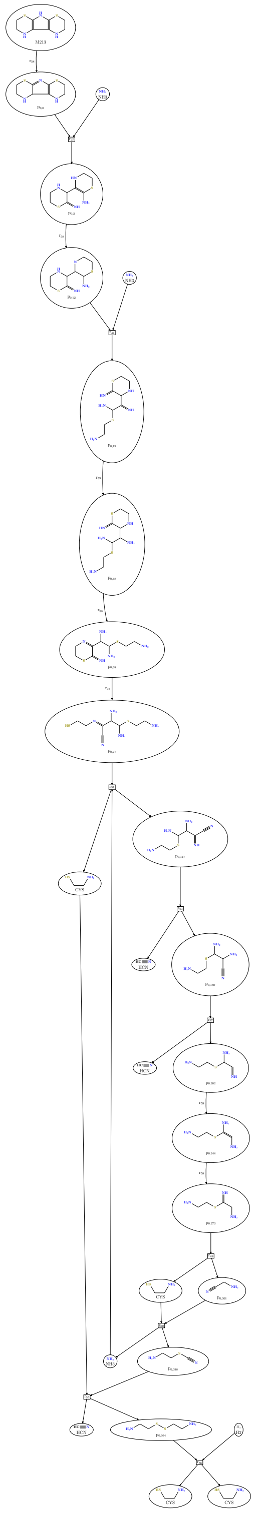

File: `out/966_dg_0_11100`

0.3.2 DG Hyper, dg_0

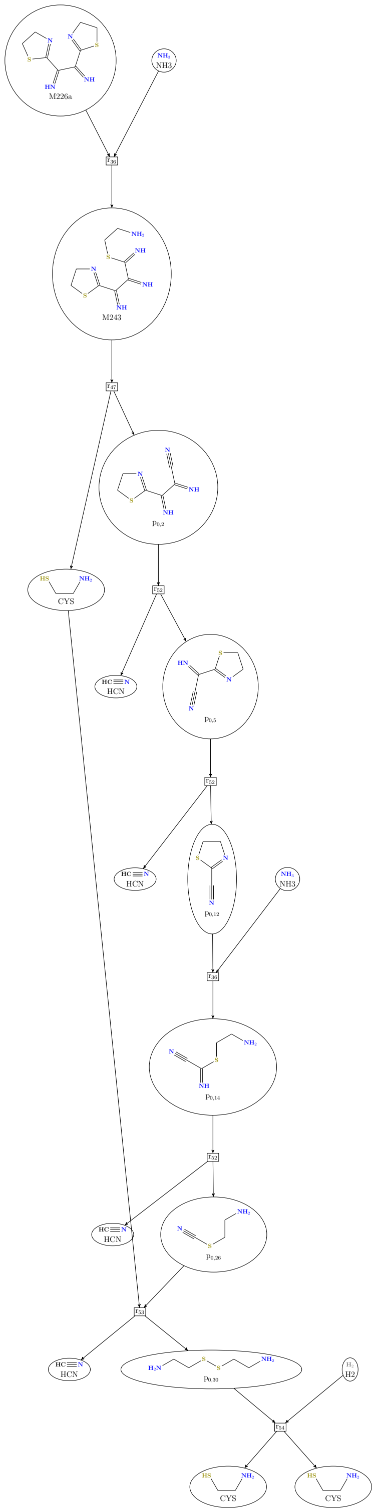

File: out/183_dg_0_11100

0.8 Catalyst p_{0,84}

0.8.1 DG Hyper, `dg_0`

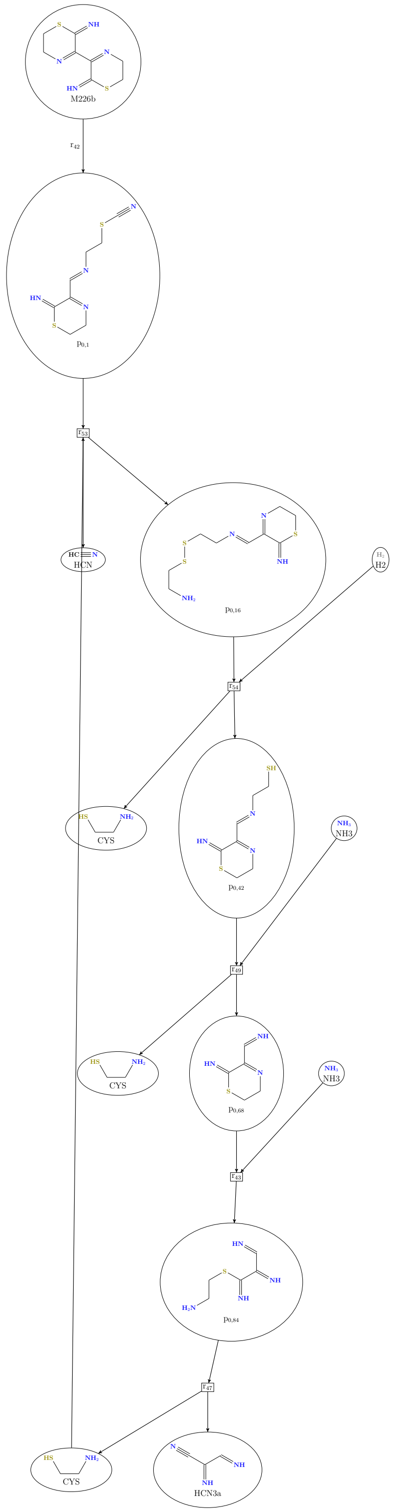

File: `out/413_dg_0_11100`

0.3.2 DG Hyper, dg_0

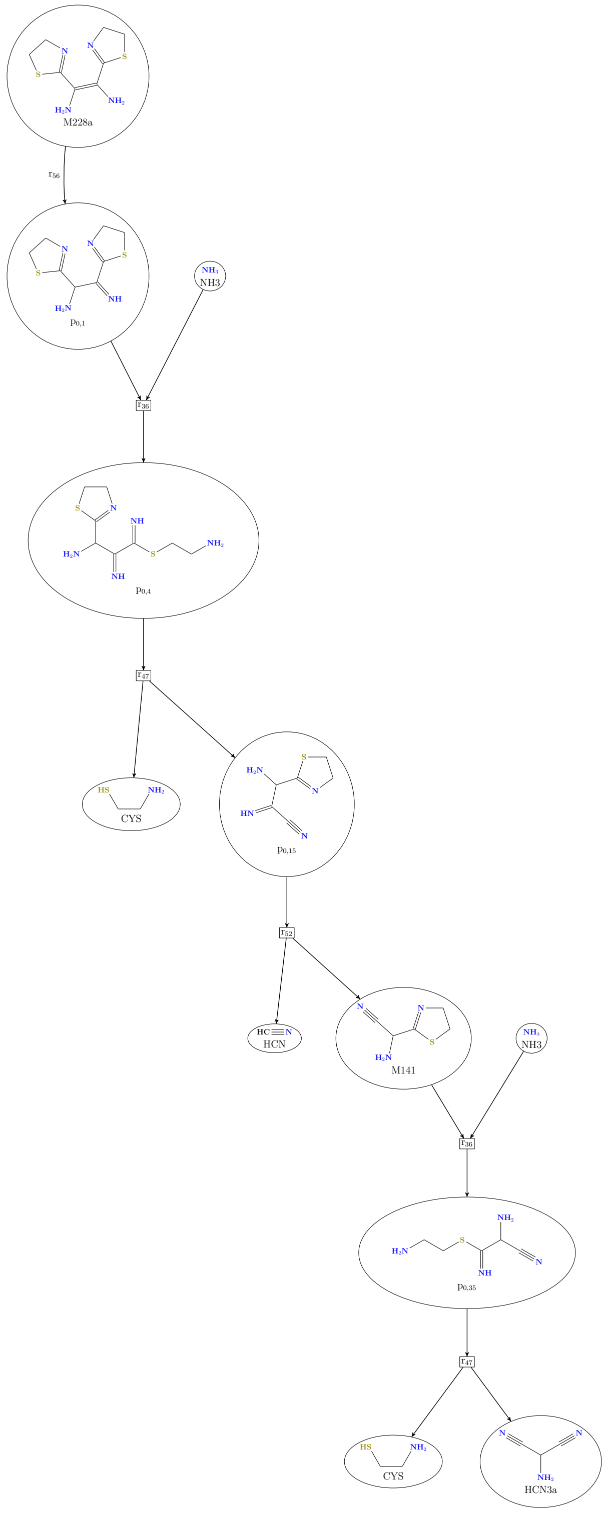

File: out/549_dg_0_11100

0.3.2 DG Hyper, dg_0

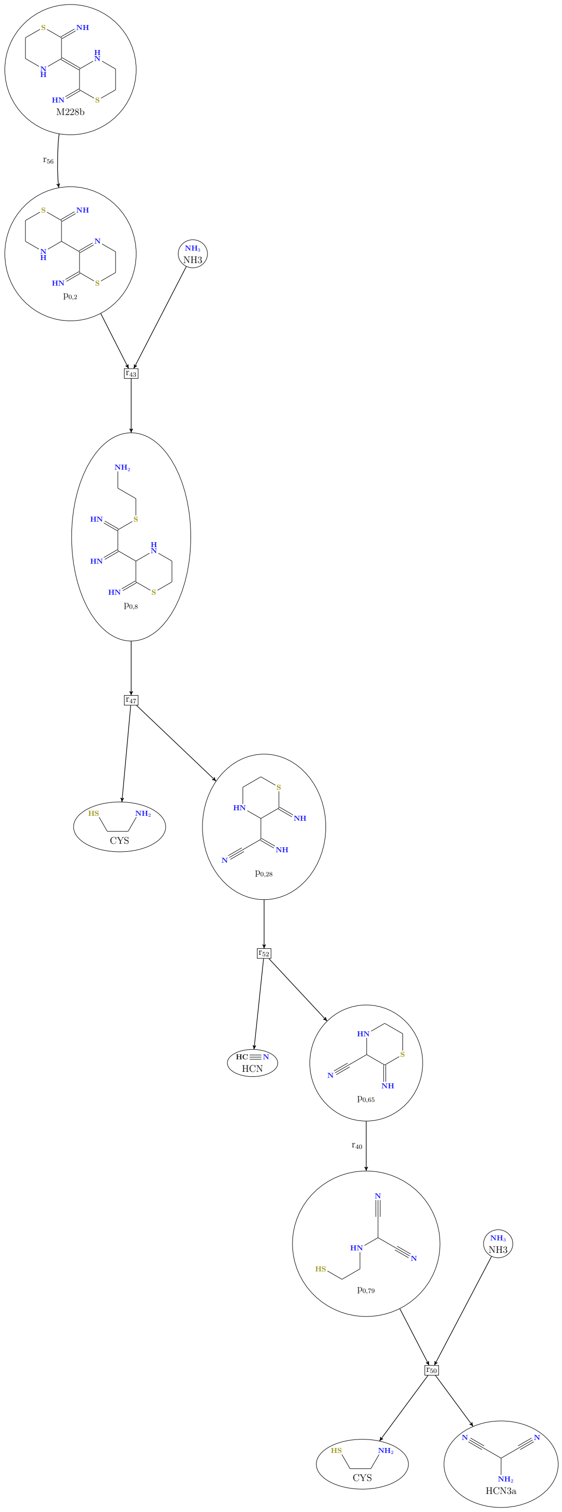

File: out/933_dg_0_11100

0.3.2 DG Hyper, dg_0

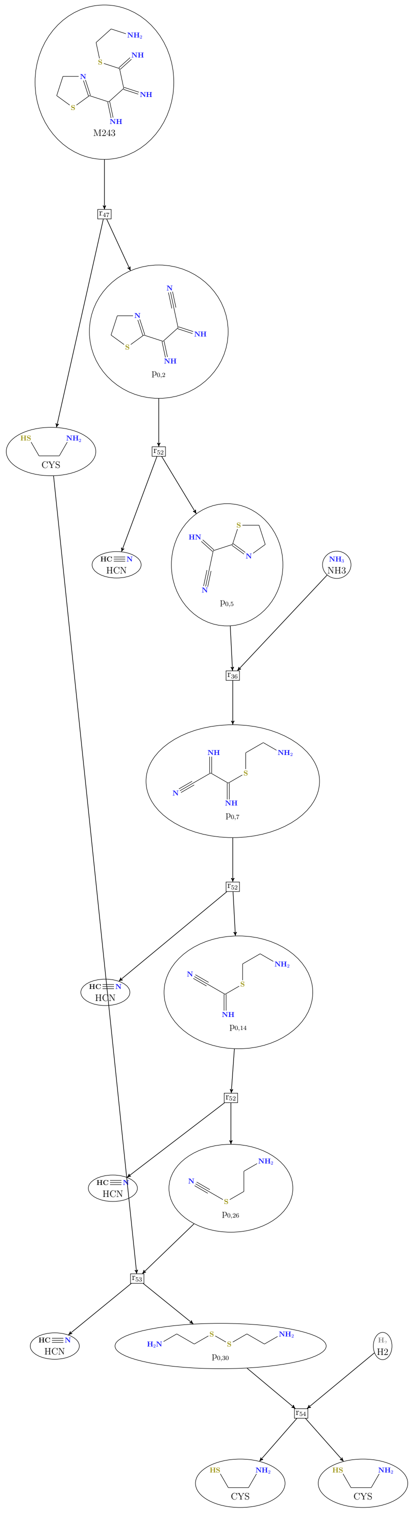

File: out/181_dg_0_11100

0.3.2 DG Hyper, dg_0

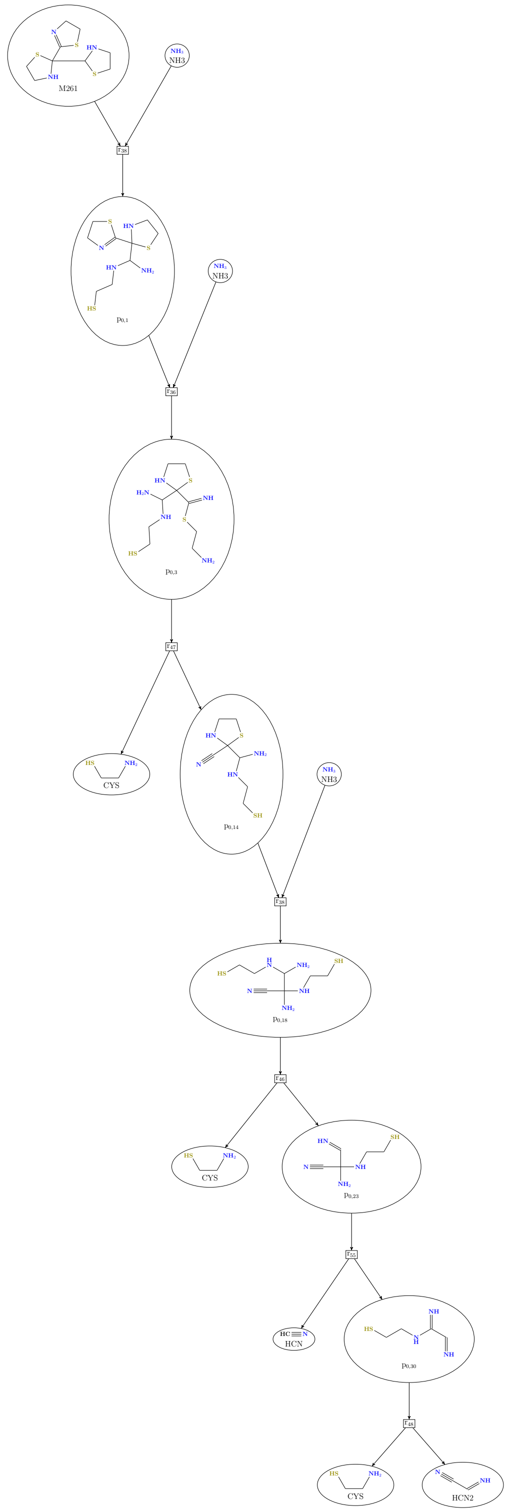

File: out/195_dg_0_11100

0.3.2 DG Hyper, `dg_0`

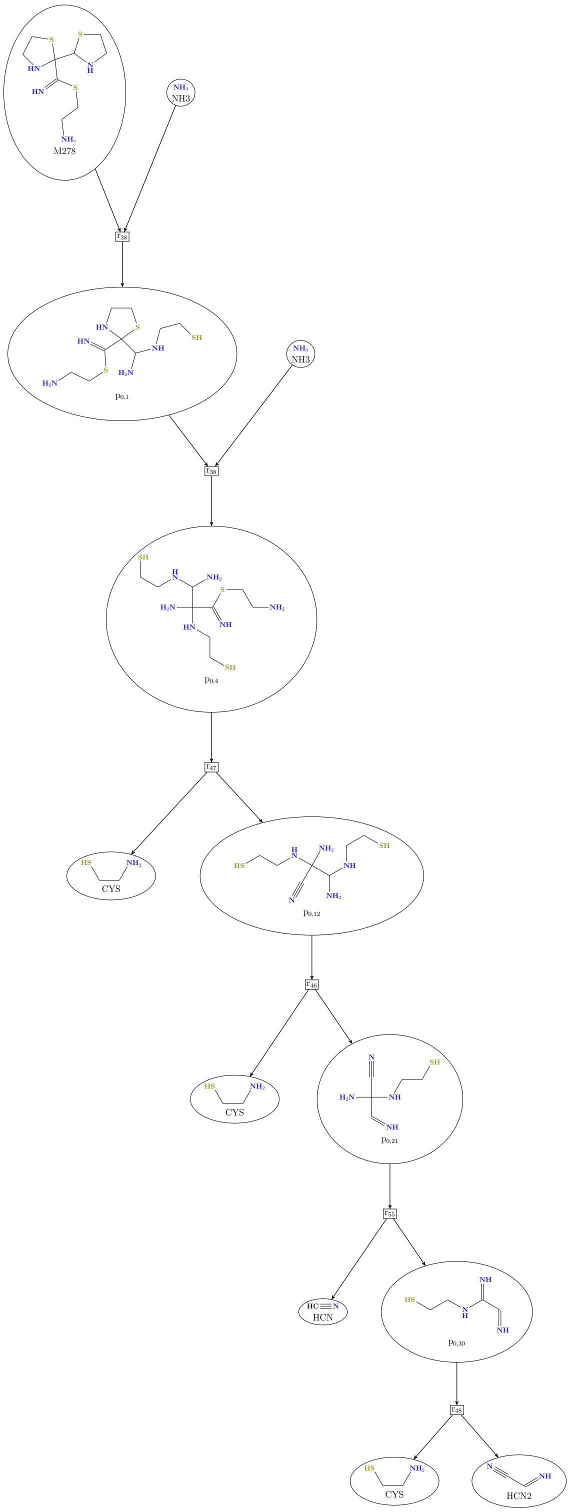

File: `out/223_dg_0_11100`

0.3.2 DG Hyper, dg_0

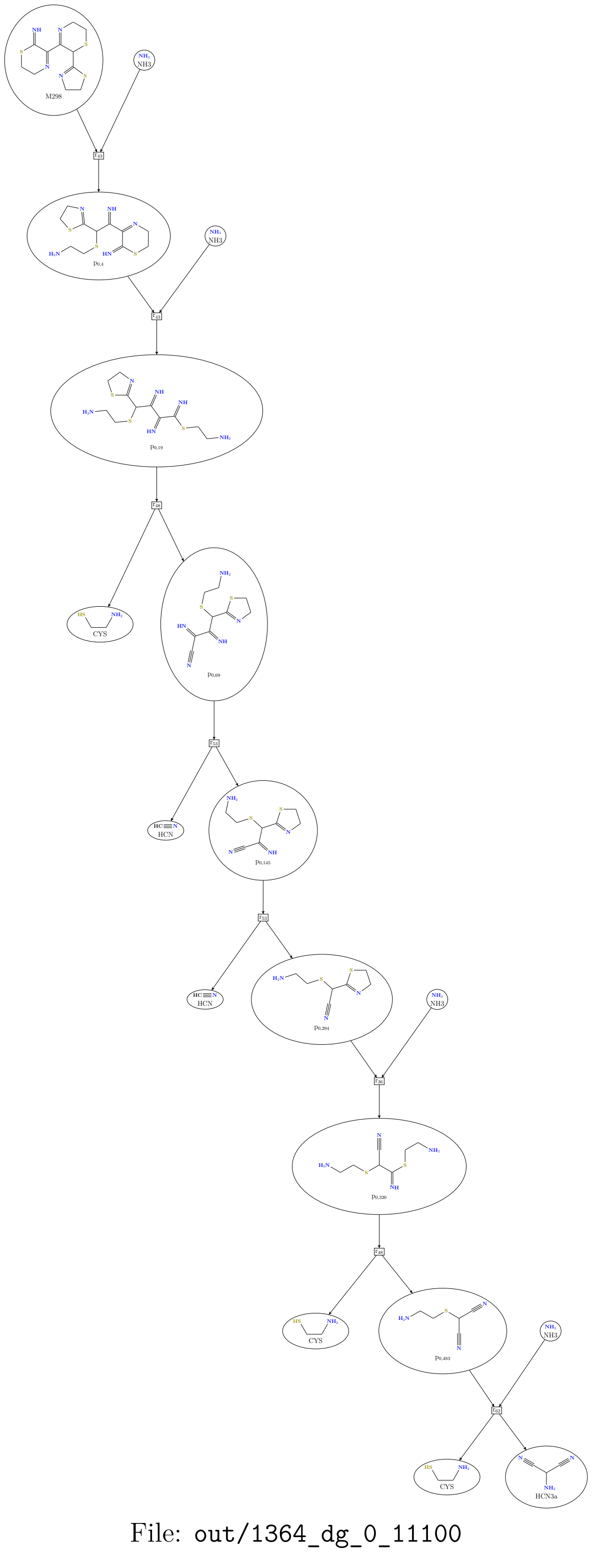

File: out/1364_dg_0_11100

0.3.2 DG Hyper, `dg_0`

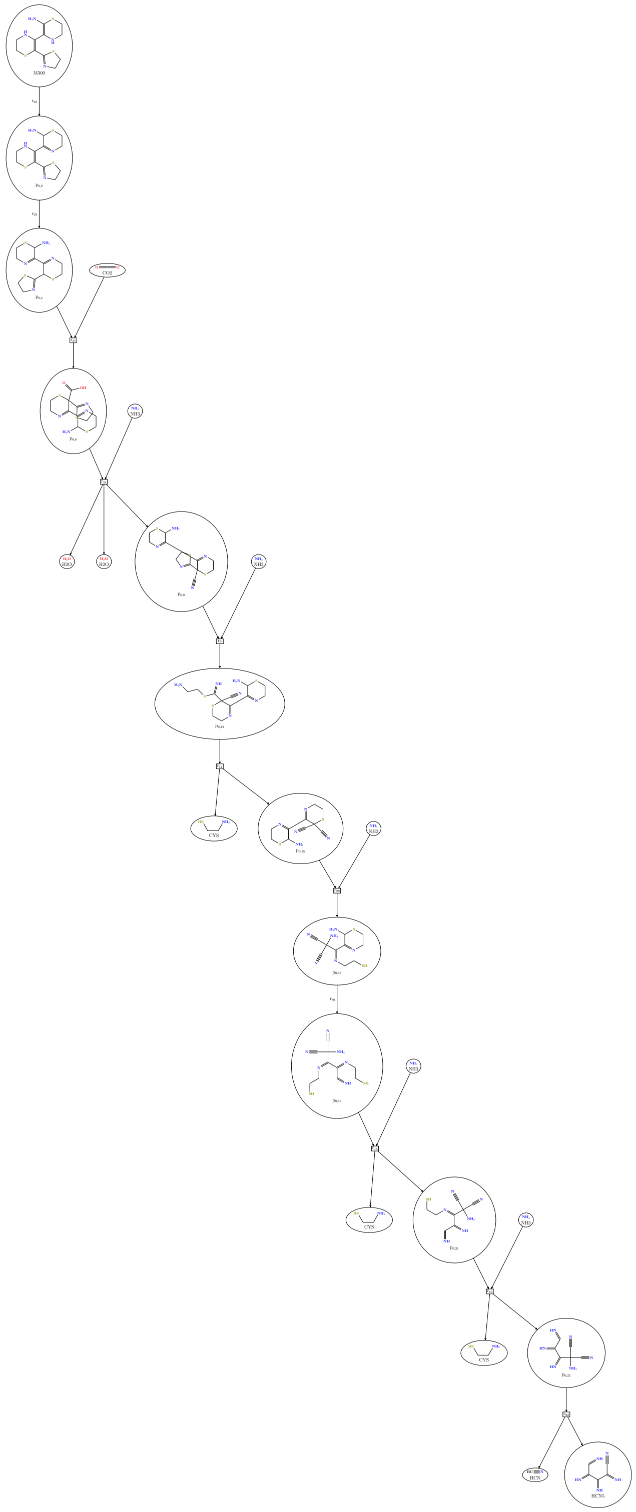

File: `out/192_dg_0_11100`

0.3.2 DG Hyper, dg_0

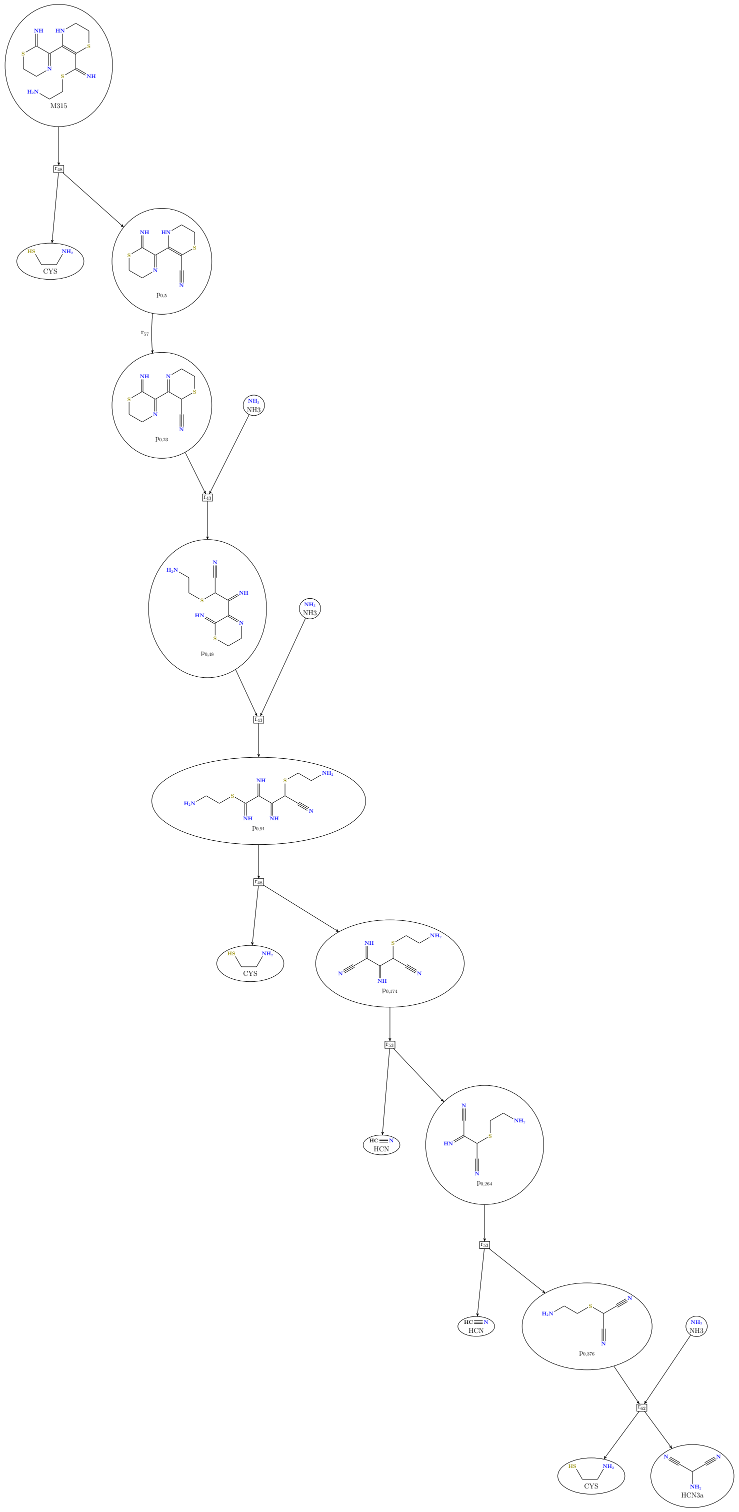

File: out/1100_dg_0_11100

0.3.2 DG Hyper, dg_0

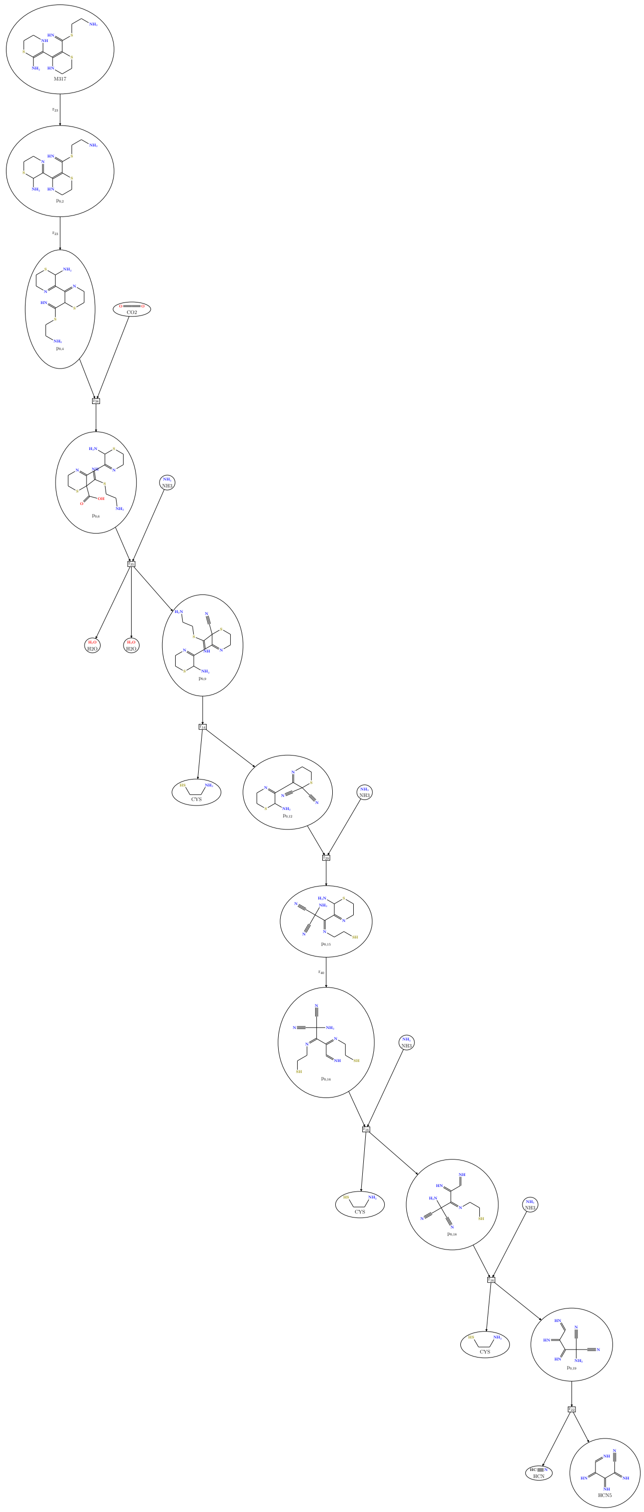

File: out/189_dg_0_11100

0.3.2 DG Hyper, dg_0

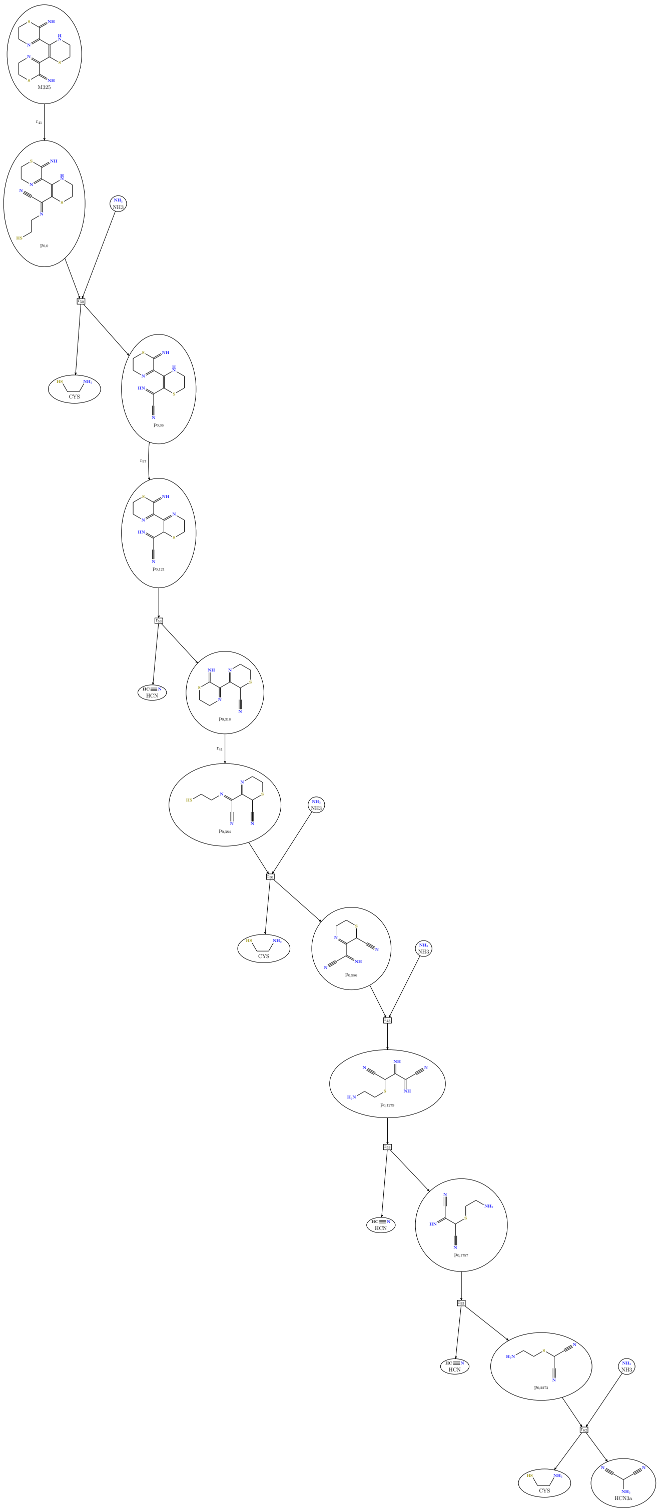

File: out/5354_dg_0_11100

0.3.2 DG Hyper, dg_0

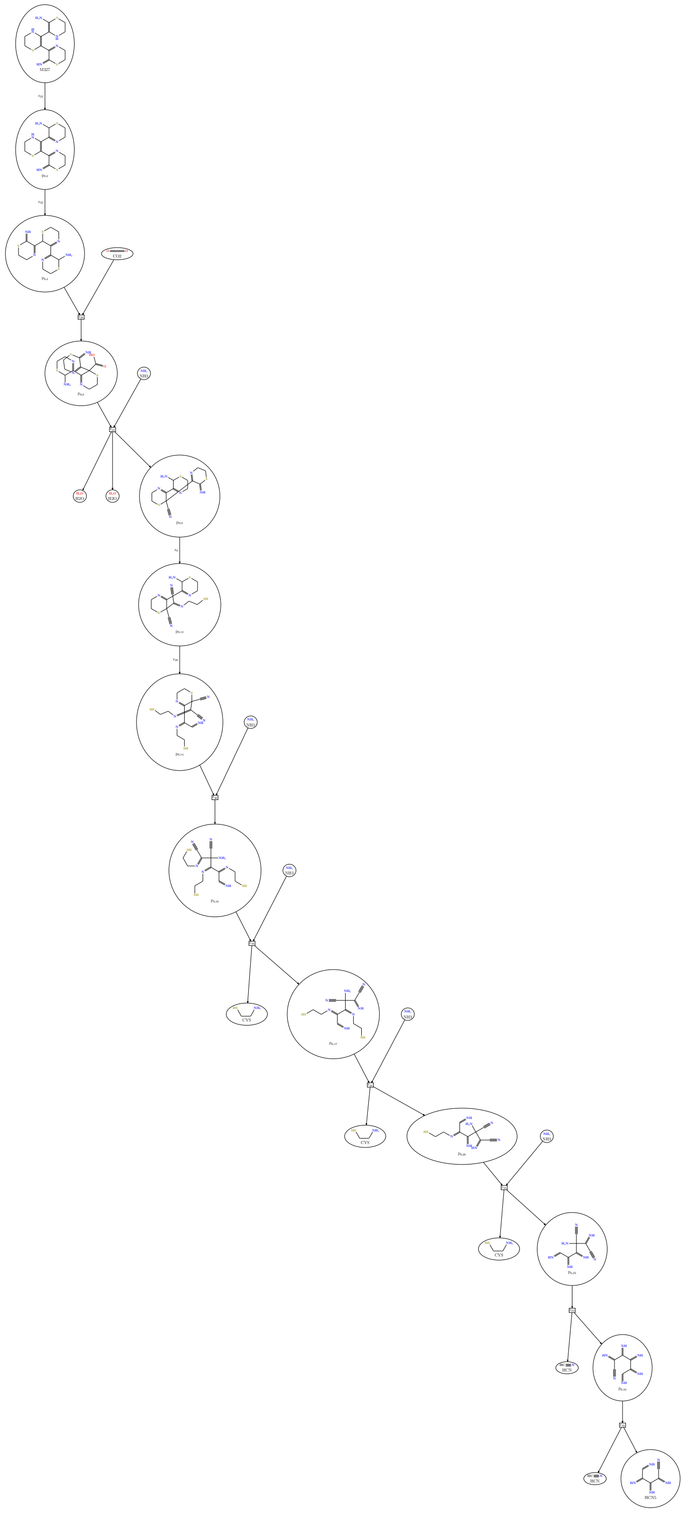

File: out/199_dg_0_11100
